# Genomic analysis of lumpy skin disease virus asian variants and evaluation of its cellular tropism

**DOI:** 10.1038/s41541-024-00846-8

**Published:** 2024-03-21

**Authors:** Shijie Xie, Lianxin Cui, Zhiyi Liao, Junda Zhu, Shuning Ren, Kang Niu, Hua Li, Fei Jiang, Jiajun Wu, Jie Wang, Jian Wu, Baifen Song, Wenxue Wu, Chen Peng

**Affiliations:** 1 National Key Laboratory of Veterinary Public Health and Safety, College of Veterinary Medicine (CVM), China Agricultural University, Beijing, 100193 China; 2 China Animal Disease Control Center, Beijing, 102618 China; 3 Xinjiang Key Laboratory of Animal Infectious Diseases/Institute of Veterinary Medicine, Xinjiang Academy of Animal Sciences, Urumqi, 830013 China

**Keywords:** Pox virus, Viral epidemiology

## Abstract

Lumpy skin disease virus (LSDV) is a poxvirus that mainly affects cattle and can lead to symptoms such as severe reduction in milk production as well as infertility and mortality, which has resulted in dramatic economic loss in affected countries in Africa, Europe, and Asia. In this study, we successfully isolated two strains of LSDV from different geographical regions in China. Comparative genomic analyses were performed by incorporating additional LSDV whole genome sequences reported in other areas of Asia. Our analyses revealed that LSDV exhibited an ‘open’ pan-genome. Phylogenetic analysis unveiled distinct branches of LSDV evolution, signifying the prevalence of multiple lineages of LSDV across various regions in Asia. In addition, a reporter LSDV expressing eGFP directed by a synthetic poxvirus promoter was generated and used to evaluate the cell tropism of LSDV in various mammalian and avian cell lines. Our results demonstrated that LSDV replicated efficiently in several mammalian cell lines, including human A549 cells. In conclusion, our results underscore the necessity for strengthening LSD outbreak control measures and continuous epidemiological surveillance.

## Introduction

Lumpy skin disease (LSD) is a viral disease found primarily in cattle, caused by the lumpy skin disease virus (LSDV). LSDV is a member of the capripoxvirus (CAPV) genus, which also includes sheeppox virus (SPPV) and goatpox virus (GTPV). SPPV and GTPV genomes are very similar to that of LSDV, exhibiting 97% nucleotide identity^1^. While these viruses typically infect different species of animals clinically, LSDV traditionally demonstrates a limited host range, primarily impacting cattle and buffalos. Nevertheless, certain studies have reported its capability to infect other ruminant species, including yaks^2^, giraffes^3^, camels^4^, and gazelles^5^. Like most poxviruses, LSDV exhibits a tropism for epidermal cells, infection of LSDV typically leads to the formation of nodules and skin lesions in infected animals.

In recent years, this disease has significantly proliferated across East, South, and Southeast Asia. Originating from Zambia in 1929, it has circulated for over 50 years in sub-Saharan Africa^6^. The first reported case outside of Africa emerged in Israel in 1989, gradually extending to other Middle Eastern countries^6^. By 2013, it had reached Turkey and further disseminated to Europe, the Balkan Peninsula, and Russia^6^. In 2017, Russia reported the initial novel recombinant viral strain. Subsequently, in 2019, several countries in East and Southeast Asia, such as Mongolia^7^, China^8^, Thailand^9^, and Vietnam^10^, observed strains closely related to the recombinant viral strain found in Russia^6^. Conversely, strains in countries such as India^11^ and Pakistan^12^ exhibited closer genetic ties to the KSGPO-like vaccine strain, indicating distinct viral transmission pathways^13^. Despite early awareness and preventive measures^14^, South Korea reported its first LSD case in October 2023^15^, indicating an ongoing expansion of the virus.

The concept of the ‘pan-genome’ was initially developed for bacterial genomes^16^, but could also be applied to studying viruses^17–20^. Viral pan-genome analysis can provide insights into a viral species or group’s genetic diversity, evolution, and functional variation. The assessment of whether a pan-genome is ‘closed’ or ‘open’ typically involves computational analyses of gene presence or absence across multiple genomes within a species or genus. The pan-genome analysis includes iterative comparisons of core genes (present in all genomes), accessory genes (present in some genomes), and unique genes (found in only one genome). When the number of unique genes in the pan-genome of a species continues to increase as more strains or isolates are included, it is considered an ‘open’ pan-genome. Conversely, if the number of unique genes reaches a plateau and remains relatively stable as more strains are added, it is considered a ‘closed’ pan-genome^16^ Some viruses with an ‘open’ pan-genome have been previously reported, including African swine fever virus with 86 core-gene clusters^19^, Chloroviruses with 126 core-gene clusters^21^, fowlpox virus with 228 core-gene clusters^20^ and Pandoraviruses with 352 core-gene clusters^17^. Identification of pan-genomes is helpful to identify conserved genes critical for viral replication and essential functions, as well as variable genes associated with virulence, host range, and other phenotypic traits such as risk of viral recombination. Understanding the genetic diversity and traits of LSDV proves instrumental in disease surveillance and control, crucial for mitigating the impact of LSD dissemination among cattle.

In this study, two strains of LSDV were successfully isolated in different parts of China and their genomes were fully sequenced. A total of 120 LSDV including two newly sequenced strains, 20 SPPV and 12 GTPV genomes, available in the GenBank database were used for a comprehensive analysis from the pan-genomic level to explore the genetic variability. Moreover, the cell tropism of LSDV was investigated using various mammalian and avian cell lines with a reporter LSDV generated for this study. Our results provide insights into the genetic diversity and complexity of LSDV circulating in China and the rest of Asia, highlighting the risk of virus recombination in affected regions, and may contribute to the preventive strategies for controlling and managing LSD.

## Result

### LSDV genome sequencing, assembly, and re-annotation

Two strains of LSDV were isolated from two cattle farms located in Fujian and Heilongjiang province in dairy cows displaying typical LSD symptoms in 2021 and 2022, respectively. Skin nodules were collected and virus isolation was conducted using MDBK cells. The isolated viruses were plaque purified three times and were confirmed to be LSDV by PCR using primers specific for LSDV ORF126 (Supplementary Fig. 1). Virus genomic DNA was collected from cushion-purified viruses and whole-genome sequencing was performed using the Illumina sequencing platform. The sequencing data for the two LSDV field isolates were designated LSDV/FJ2021 (OP922506) and LSDV/HLJ2022 (OQ555660) and are provided in the supplementary materials (Supplementary Table 1). The De novo assembly process resulted in six or five contigs for LSDV/FJ2021 and LSDV/HLJ2022, with the longest being 145,928 bp and 148,169 bp, respectively. The average genomic coverage was approximately 622.23x and 1,183.89x for LSDV/FJ2021 and LSDV/HLJ2022, respectively, with 149 and 152 putative open reading frames (ORFs) for the two strains. We also obtained a total of 120 different LSDV genome sequences including two isolated strains, 12 GTPV genome sequences, and 20 SPPV genome sequences from NCBI, which were manually verified, particularly about strain virulence. The genome annotation result is listed in Supplementary Table 4. As the LSDV genomes found in the NCBI databank were incompletely annotated, we performed re-annotation and comparison of the genomes using Prokka with AF325528 as the reference genome (Supplementary Table 2 and Supplementary Fig. 2). The annotation of AF325528 was obtained from the two different methods, Prokka and GCG, which exhibited a 1.2% (2/156) difference between the two (Supplementary Table 3). Two genes LSDV069/LSDV106 were not annotated in Prokka, and LSDV00028/LSDV00119 were predicted in Prokka but not in GCG (Supplementary Table 3). LSDV encodes for four complete putative ORFs located in the inverted terminal repeat region (ITR), namely LSDV001/LSDV156, LSDV002/LSDV155, LSDV003/LSDV154, and LSDV004/LSDV153, all of which were duplicated. To ensure data consistency, we re-annotated all 152 genomes using Prokka.

### LSDV contains an ‘open’ pan-genomes

**Fig. 1:**
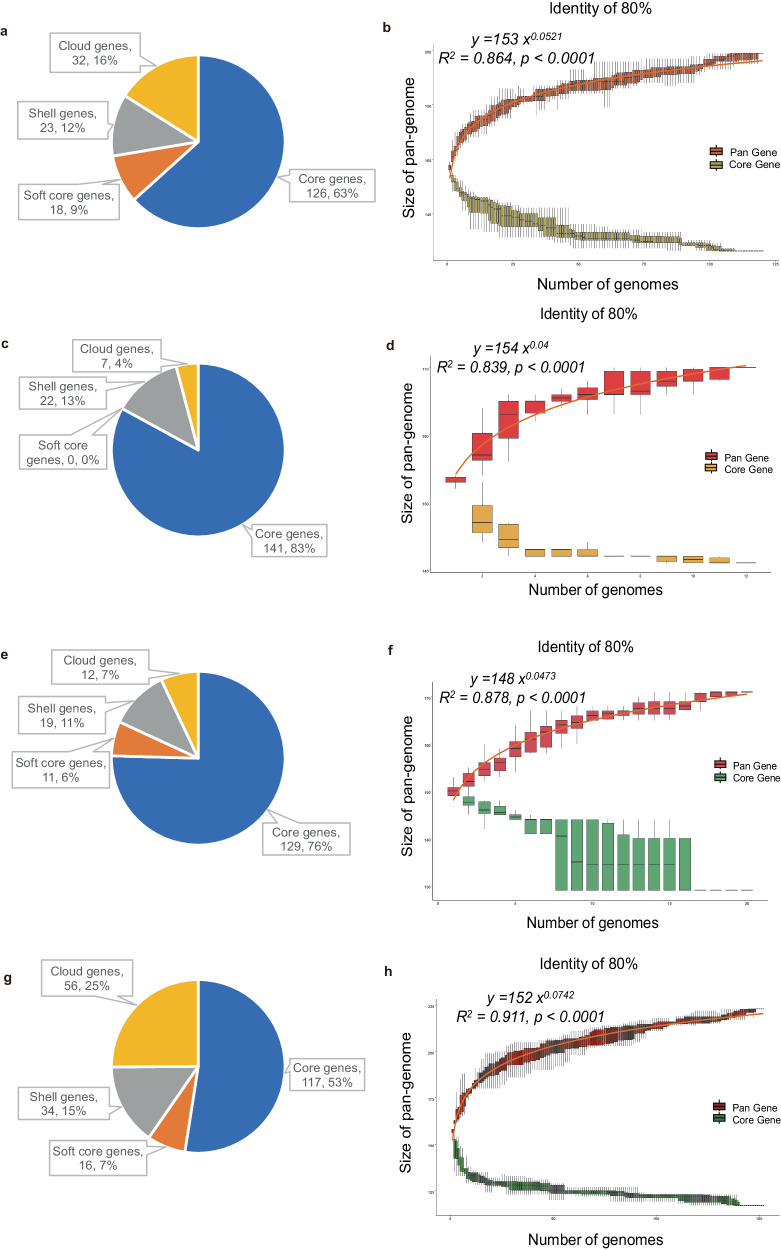
Pan-genome analysis of LSDV, GTPV, SPPV, and CAPV based on protein-coding genes. The quantity and percentage of genes at an 80% sequence identity threshold were assessed for LSDV (**a**), GTPV (**c**), SPPV (**e**), and CAPV (**g**). Core genes (100% <= strains <= 100%), Soft core genes (95% <= strains < 100%), Shell genes(15% <= strains < 95%), Cloud genes(0% <= strains < 15%), Total genes(0% <= strains <= 100%). The number of core genes x-ais plotted as a function of newly added LSDV strains (**b**), GTPV strains (**d**), SPPV strains (**f**), and CAPV strains (**h**). The boxplots illustrate the number of total genes or core genes in the pan-genome. The center line corresponds to the median value of the dataset. The bounds of the box depict the interquartile range (IQR). The whiskers extend from the box, representing the range of the data, and the ends of the whiskers denote the minimum and maximum values in 10 random samples.

Roary was employed for LSDV pan-genome analysis. After assessing the effects of different sequence similarities on the identification of core genes, 80% was chosen as an appropriate parameter for pan-genome estimation (Supplementary Fig. 3). The analysis of 120 LSDV strains unveiled 199 gene clusters, among which 126 were universally shared across all viruses, delineated as core genes (Fig.1a and Supplementary Table 5). However, among the 152 CAPV strains, the pan-genome consisted of 223 gene clusters, with 117 genes categorized as core genes (Fig. 1g and Supplementary Table 6). To estimate the size of the pan-genome of LSDV, we sampled a varied number of strains (from 1 to 120, with increments of 1) from our data set, and for each number of strains, we randomly resampled 10 times. Power regression (y = a⋅x^b^) was conducted to determine the property of the pan-genome of LSDV, using the median number of total genes for each number of strains. The b parameter was estimated as 0.0521 ± 0.000619. The parameters of the fitted curve unveiled a continuous increase in the count of unique genes with the LSDV pan-genome as the number of stains grew. The estimated parameters indicated that the count of unique genes in the LSDV pan-genome continued to rise as more strains were included in the analysis. This observation supports the ‘open’ pan-genome concept, emphasizing that the genomic repertoire of a species or viral population is not fixed but continuously evolves and expands as more genetic data from diverse strains or individuals are incorporated into the analysis^16^.

Additionally, we scrutinized the complete pan-genome data of GTPV, SPPV, and the entire CAPV, employing various cut-off values spanning from 75% to 90% (Supplementary Fig. 4–6). For comparative purposes with LSDV results, the same 80% threshold was chosen as the optimal parameter for pan-genome estimation. For CAPV, the ‘b’ parameter was estimated at 0.0742 ± 0.000626 (Fig. 1g, h). For GTPV, it stood at 0.04 ± 0.00164 (Fig. 1c, d), while for SPPV, the parameter ‘b’ was estimated to be 0.0473 ± 0.00128 (Fig. 1e, f). This indicates that whether it’s GTPV, SPPV, or the entire CAPV genus, all encompass an ‘open’ pan-genome.

### Phylogenetic analysis of capripoxviruses

**Fig. 2:**
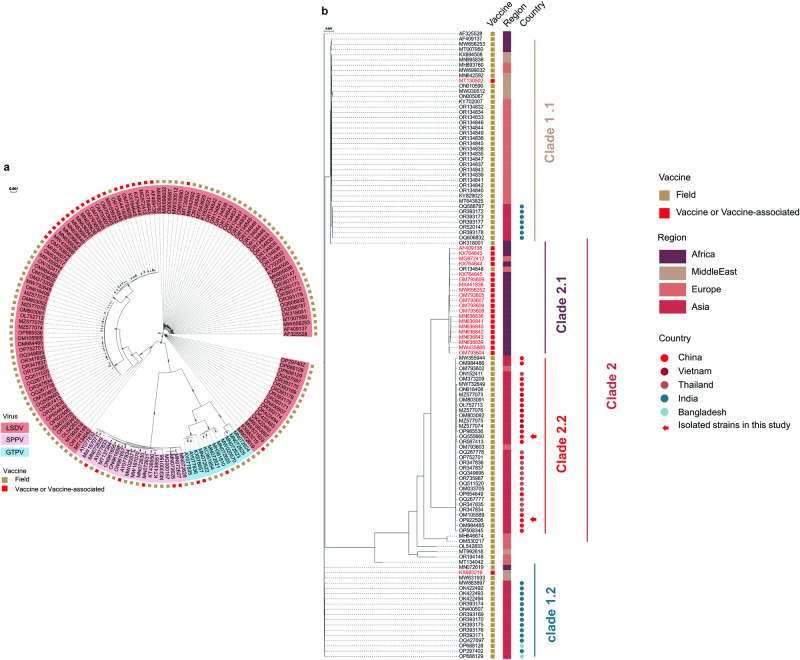
Phylogenetic analysis of LSDV, SPPV, and GTPV. Phylogenetic trees were based on based on core genes of CAPV with model Blosum62+FO (**a**) and LSDV with model TIM + F + I + G4 (**b**). Bootstrapping was performed with 1000 replicates.

Core genes constitute a subset of genes conserved across all or most strains within a species or genus. Typically, these genes encode essential functions and exhibit a lower susceptibility to rapid evolution or horizontal gene transfer. This characteristic makes core genes suitable for deducing evolutionary relationships, contributing to more precise and dependable phylogenetic reconstructions. Therefore, the phylogenetic tree was constructed on 117 core genes for 152 CAPVs (Fig. 2a). We utilized the ModelFinder software to identify the most suitable substitution model based on the Bayesian Information Criterion (BIC). Subsequently, a phylogenetic tree was constructed using the optimal substitution model, with 1,000 replicates for bootstrapping values. The results showed that LSDV, GTPV, and SPPV formed distinct clusters, indicating that this method was appropriate for cluster analysis for CAPVs. However, we noticed that when we included more data on virus virulence, vaccines, or vaccine-related strains, different CAPVs may cluster in the same sister branch (Fig. 2a). To correct this, we performed further analysis on 120 LSDV strains and used ‘IQ-TREE’ to construct phylogenetic trees (Fig. 2b).

All LSDV tested were classified into two major clades (Clade 1, and Clade 2), which were further divided into two sub-clades (namely Clade 1.1/1.2 and Clade 2.1/2.2). Most vaccine and vaccine-associated strains were found within Clade 2.1, with only three exceptions, namely MT130502, KX683219, and OK422494. The Neethling-RIBSP strain (MT130502) was a Kubash/KAZ/16 (MN642592) attenuated strain, isolated by Kubash in Kazakhstan in 2016^22^, which underwent nine passages in lamb testicle cells, followed by five passages in chorioallantoic membranes of embryonated chicken eggs and 33 passages in the MDBK cells. Whether the serial passage affected the virulence of the virus was unknown. The vaccine strain KSGP 0240 (KX683219) was isolated from sheep and has been widely used as a vaccine strain for GTPV and SPPV^23^. However, it did not provide expected protection against LSDV in Israel^24^, Ethiopia^25^, and Morocco^26^. The Lumpi-ProVac^Ind^ strain (OK422494) was a LSDV/2019/RCH(MW883897) attenuated strain^11^, isolated by Naveen Kumar in India in 2019, which underwent fifty passages in Vero cells. The vaccine in cattle was safe and provided protective efficacy against wild-type LSDV^27^.

Outbreaks of LSD have been reported in many countries across different continents. To simplify the analysis, we attempted to visualize the geographic distribution of the viruses based on regions including Africa, the Middle East, Europe, and Asia. Most strains isolated in the Middle East and Africa were clustered in Clade 1.1 and Clade 2.1, while strains from Asia were clustered in Clade 1.2 and Clade 2.2, indicating the relevance of geographic distribution. The first evidence of recombination of the vaccine strains of LSDV with the field strains was reported in Russia^28^. An increasing amount of new evidence is surfacing which suggests a high frequency of recombination of LSDV^29–31^. In light of the recent outbreaks in Asia, we included only strains isolated from Asian countries. Interestingly, distinct variations were observed between the viral strains responsible for the epidemic in South Asia versus Southeast Asia, implying the potential existence of two distinct pathways of transmission (Fig. 2b)^13^. The viruses isolated in China, Vietnam, and Thailand clustered in Clade 2.2, while strains isolated in India and Bangladesh clustered in Clade 1.2, closer to the KSGP 0240 vaccine strain. The virus strains responsible for the epidemic in mainland China, Hong Kong, Taiwan, Vietnam, and Thailand exhibited greater similarities to the recombinant strains in Russia, relative to those found in India, suggesting a unique evolutionary pathway of LSDV in India^13^. Additionally, our observations reveal that several recent isolates from India align with clade 1.1 (Fig. 2b), suggesting the presence of at least three viral subtypes across East, South, and Southeast Asia. From the evolutionary relationships of the recombinant virus strains with the field strains, Clade 2.2 appears to be a result of restructuring events from Clade 1.1 and Clade 2.1(Fig. 2b). Thus far, there have been no reported instances of subsequent recombination between the recombinant Clade 2.2 and other Clades.

### Identification of putative virulence genes in CAPV

**Fig. 3:**
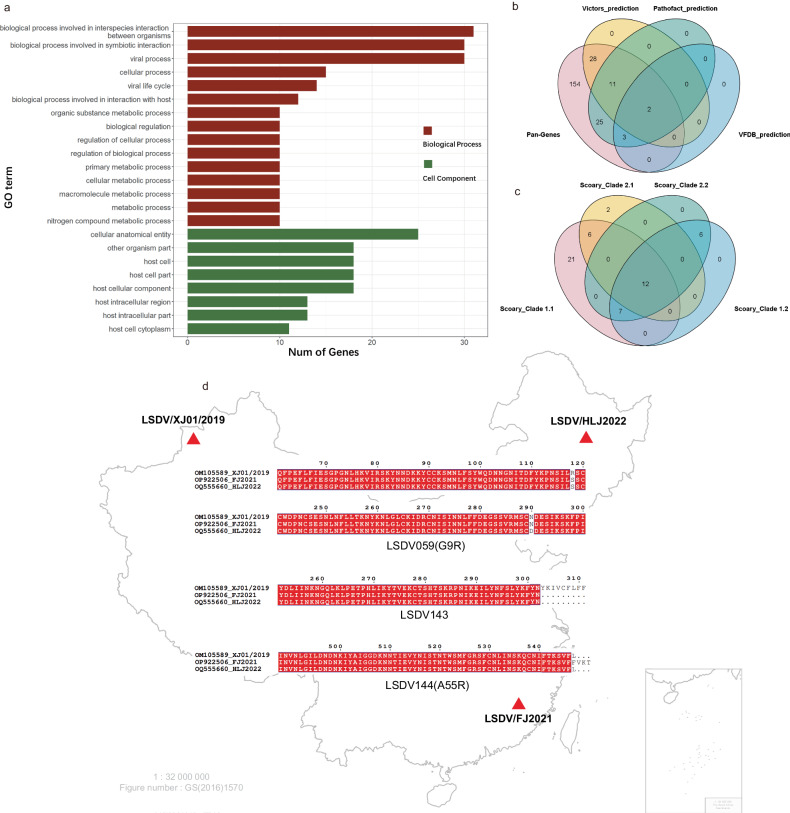
Functional annotations of CAPV genes. The numbers of the functionally annotated CAPV genes enriched on GO terms (**a**). Gene numbers of virulence annotation by VFDB, Victors, and Pathofact (**b**). Genes were associated with LSDV Clade 1.1, Clade 2.1, Clade 2.2, and Clade 1.2 annotation by Scoary (**c**). Geographic distribution of the LSDV/XJ01/2019, LSDV/FJ2021, and LSDV/HLJ2022 and Sequence alignment results of SNP/indel variants(**d**).

As the functions of most genes of LSDV are unclear, we re-annotated the potential functions of the CAPV genes. In total, we annotated the function of 182 out of 223 gene clusters in the CAPV pangenome using eggnog. Detailed GO analysis revealed 15 biological pathways and 8 cellular components that were enriched (Fig. 3a). The limited abundance of enrichment results could be attributed to the unknown functions of poxvirus homologous proteins. From the analysis of the pan-genome, it was revealed that LSDV harbors 126 core genes, whereas CAPV possesses 117 core genes (Fig. 1a, g). Among these, nine genes—such as A52R-like family proteins LSDV009 and LSDV136, LAP/PHD-finger protein LSDV010, G protein-coupled CC chemokine receptor LSDV011, Ankyrin repeat protein LSDV012, IL-1 receptor LSDV013, CD47-like protein LSDV128, LSDV132, and VAR B22R homolog LSDV134—are conserved in LSDV but not in CAPV. Notably, LSDV132 lacks a homologue in SPPV and GTPV. Predictions from VFDB^32^, Victors^33^, and Pathofact^34^ were employed to anticipate virulence factors within the CAPV genomes, identifying a total of 69 proteins as potential virulence contributors (Fig. 3b and Supplementary Table 9). Among these, LSDV131 (Copper/zinc superoxide dismutase, SODC)^35^ and LSDV139 (protein serine/threonine kinase activity) were annotated as virulence factors across all three databases. Furthermore, seven proteins were annotated as toxins, while 38 proteins were labeled as putative virulence factors by Pathofact. Noteworthy are three Kelch-like proteins (LSDV019, LSDV144, and LSDV151), tyrosine phosphatase LSDV072 (H1L), and phospholipase D-like protein LSDV146 (K4L), all annotated as both virulence factors and toxins. The phylogenetic tree (Fig. 2b), constructed based on core genes, delineates distinct Clades of LSDV that have spread extensively across diverse countries and regions. Utilizing Scoary^36^, a pan-genome-wide association study tool, we revealed the association between the presence or absence of pan-genome genes and observed phenotypes across different clades (Fig. 3c and Supplementary Table 9). A52R-like family protein LSDV001/LSDV156, unknown function protein LSDV002/LSDV155, ER-localized apoptosis regulator LSDV003/LSDV154, unknown function protein LSDV004/LSDV153, Kelch-like proteins LSDV019 (F3L) and LSDV144 (A55R), as well as VAR B22R homolog LSDV134, emerged as potential contributors in this process.

The first LSD case in China was reported in Xinjiang Province in 2019^37^. The virus has since spread rapidly and has been detected in more than twenty provinces in China. We isolated two strains from two different locations that are both more than 2000 km away from the location of the original outbreak (Fig. 3d). The differences between the two strains and the Xinjiang strain were further analyzed to assess the evolutionary path of the virus in China (Fig. 3d and Table 1). Single nucleotide polymorphism (SNP) or nucleotide insertion were observed in five genes including the myristoylated protein LSDV059 (G9R), superoxide dismutase-like protein LSDV131(A45R), IFN-α/β binding protein LSDV135 (B19R), tyrosine-protein kinase-like protein LSDV143, Kelch-like protein LSDV144 (A55R), which formed missense, frameshift or a premature stop codon. LSDV059 (G9R) contained a non-synonymous mutation R118S found in both two strains, and another non-synonymous mutation N290D in LSDV/HJL2022. LSDV143 contained a mutation that caused premature translation stops, resulting in a deletion of 9 amino acid residues from the C-terminus in both strains. In addition, insertion in LSDV144 (A55R) resulted in an addition of 3 amino acid residues to the C-terminal end in LSDV/FJ2021. However, it is essential to substantiate the biological consequences resulting from genetic alterations through further assessment.

**Table 1:** The SNP/INSERT between LSDV/XJ01/2019 and LSDV/FJ2021, LSDV/HLJ2022

GBID	POS	TYPE	REF	ALT	EFFECT	LSDV	VACV	PRODUCT	STRAIN
OM105589	53125	snp	A	G	missense	LSDV059	G9R	Myristylated protein	LSDV/HLJ2022
OM105589	52611	snp	G	T	missense	LSDV059	G9R	Myristylated protein	Both
OM105589	119371	snp	C	A	missense	LSDV131	A45R	superoxide dismutase-like protein	Both
OM105589	128695	snp	G	A	missense	LSDV135	B19R	IFN-α/β binding protein, SP	LSDV/HLJ2022
OM105589	135444	snp	C	A	stop	LSDV143		Tyrosine-protein kinase-like protein	Both
OM105589	137245	ins	GTTTTTTTGTA	GTTTTTTTTGTA	frameshift	LSDV144	A55R	Kelch-like protein	LSDV/FJ2021

*GBID* Genebank ID, *POS* Position of the genome, Type Variation type, *REF* Nucleotide sequence in the reference genome, *ALT* mutated nucleotide sequence, *EFFECT* The effect of variation, *LSDV* Gene name in LSDV, *VACV* Gene name in LSDV, *PRODUCT* Gene annotation information, *STRAIN* mutation in strains, LSDV/FJ2021(OP922506), LSDV/HLJ2022 (OQ555660), *Both* in two strains.

### Cellular tropism of LSDV

**Fig. 4:**
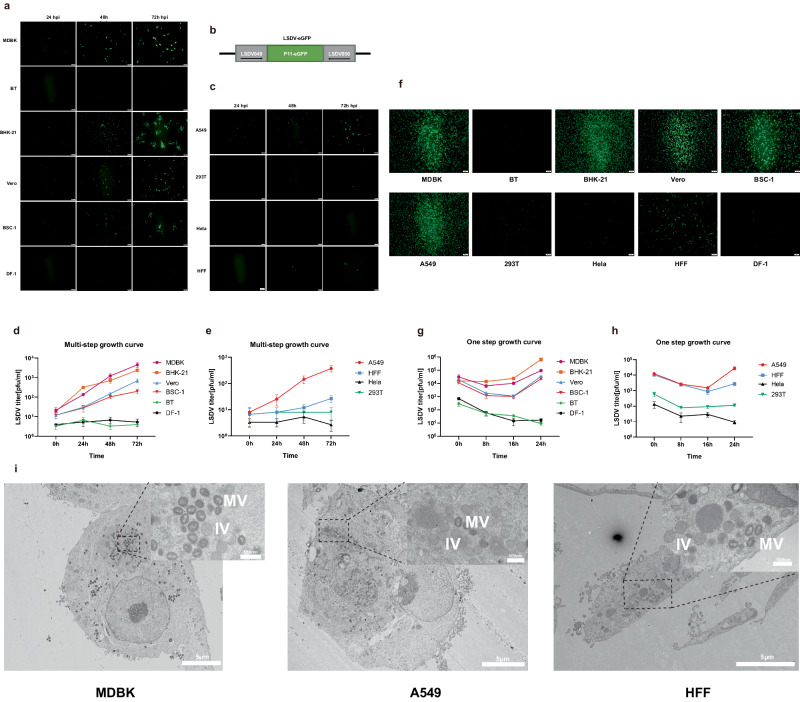
LSDV growth curves and mature virus particles in different cells. LSDV exhibits the ability to replicate within various mammalian cell types, encompassing A549 cells. The configuration utilized for producing the reporter LSDV is depicted in **b**. Fluorescent imagery was obtained at 24 hours post-infection (hpi), 48 hpi, and 72 hpi after infection with LSDV-eGFP at 0.01 MOI (**a** and **c**) or 3 MOI (**f**). Viruses were collected and quantified via plaque assay in MDBK cells, with the growth curves depicted in **d**, **e**, **g**, and **h**. Plaque counting was conducted in triplicate replicates, and the error bars indicate standard deviation. Mature virus particles were visualized using transmission electron microscopy after infecting different cells with LSDV at 0.1 MOI for 48 hours (**i**). The scale bar length for fluorescence images is 200 µm, and for electron microscopy photos of virus particles, it is 5 µm or 500 nm. The scale bar length is marked in the lower right corner of each picture.

Multiple cell lines, including primary and continuous cell lines, have been utilized to assess the growth and replication of LSDV (Supplementary Table 8). Considering the diversity among various clinically isolated strains, we further evaluated the growth capability of LSDV-FJ2021 in different cell lines. We engineered a reporter virus by inserting a cassette with a P11-directed eGFP-ORF into the intergenic region between LSDV049 and LSDV050 using the LSDV/FJ2021 strain (Fig. 4b), which is a suitable insertion site for foreign genes and the insertion does not affect the expression of upstream and downstream genes^38^. To validate the replication capability of LSDV-GFP in Madin-Darby bovine kidney (MDBK) cells and to eliminate any potential confounding effects of GFP in broadening the cell range, we infected MDBK with three virus strains (HLJ2022/FJ2021/FJ2021-GFP) at 0.1 PFU/cell. After 24 hours, we observed the presence of viral protein H3L, an intermediate/late protein used for capripoxvirus detection^39,40^ (Supplementary Fig. 7). Initially, MDBK cells, bovine testis (BT) cells, baby hamster Syrian kidney (BHK-21) cells, African green monkey kidney (Vero) cells, African green monkey kidney (BS-C-1) cells, and chicken embryo fibroblasts (DF-1) cells were used to evaluate virus replication at 0.01 and 3 PFU/cell (Fig. 4a, f). LSDV was able to replicate in MDBK and BHK-21 cells to comparable titers, and the number of progeny virus formed in BHK-21 cells was slightly higher than that in MDBK cells at 24 hours, but slightly lower at 72 hours (Fig. 4a). The replication ability of LSDV in Vero and BS-C-1 cells was lower than that in MDBK and BHK-21 cells. LSDV was barely reproducible in DF-1 cells. We were surprised to find that LSDV was not able to replicate in BT cells, which are used for the replication and production of classical swine fever virus (CSFV)^41^.

Four human cell lines, including cancerous cell lines such as human lung adenocarcinoma (A549), cervical cancer (HeLa), human embryonic kidney (293 T), and non-cancerous cells such as human foreskin fibroblasts (HFF), were also infected with LSDV-eGFP at 0.01 and 3 PFU/cell (Fig. 4c, f). Surprisingly, A549 and HFF cells were infected by LSDV, while HeLa and 293 T cells were unable to support the production of LSDV (Fig. 4c, f). The replication ability of LSDV in A549 cells was comparable to that in Vero and B-SC-1 cells, but lower than in MDBK and BHK cells. LSDV showed limited replication in HFF cells. The infectious dose of LSDV in HFF cells was similar to that in A549, but the replication ability of the virus in HFF seemed to be lower than that in A549 cells. Mature virus particles were observed in MDBK, A549, and HFF cells using transmission electron microscopy, following infection with LSDV at a multiplicity of infection (MOI) of 0.1 for 48 hours (Fig. 4i). Furthermore, the detection of proteins at different time points post-infection provides additional evidence supporting the possibility of LSDV replication in A549 cells (Supplementary Fig. 7).

## Discussion

Poxviruses are large DNA viruses and as a family, can infect a diverse range of animal species. In recent years, mpox virus (MPXV) and LSDV have spread rapidly worldwide in many areas and gained global attention^42^. The estimated economic loss caused by LSDV was up to 1.46 billion dollars globally^43^. Therefore, there is an immediate necessity for increased research endeavors aimed at elucidating the virus’s evolutionary trajectory, as well as its host and cellular tropism. In this study, we aimed to gain a comprehensive understanding of the evolution of LSDV in Asian countries through Comparative Genomics Analysis. We isolated two strains from mainland China and conducted whole genome sequencing, annotation, and phylogenetic analysis. To bolster our investigation, we systematically analyzed 120 LSDV, 20 SPPV, and 12 GTPV genomes using a comparative genomics approach. Our Comparative genomic analysis revealed that LSDV, SPPV, and GTPV possess an ‘open’ pan-genome, and the entire Capripoxvirus genus shares this trait. An ‘open’ pan-genome denotes a dynamic gene pool, echoing the evolutionary patterns observed in poxviruses^44^. These genetic dynamics involve homologous and nonhomologous recombination, gene duplications, gene loss, and the acquisition of new genes through horizontal gene transfer^44^. The core gene counts for LSDV held firm at 126, while for SPPV, it remained at 129, and for GTPV, it remained at 141. In terms of the total gene clusters, LSDV maintained its count at 199, SPPV at 171, and GTPV at 170. There are 49 conserved gene families shared between entomopoxviruses and chordopoxviruses, with an additional 90 genes conserved specifically within chordopoxviruses^45^. These genes constitute the core essential genome, serving as promising targets for drugs, antibodies, vaccines, and detection methodologies^45^. The fowlpox virus encompasses 297 gene clusters, out of which 228 are core genes, exhibiting an “open” pangenome configuration^20^. Similarly, the CAPV genus also demonstrates an “open” pangenome, with a shared set of 117 core genes identified (Supplementary Table 6). These variations in core gene numbers could be attributed to differences in the gene content across various viruses, as well as the scope of the pan-genome analysis, whether conducted at the species, genus, or family level.

The phylogenetic analysis based on core genes revealed distinct branches of LSDV evolution. In 2016, an LSDV outbreak occurred in Kazakhstan^46^, and a mass vaccination campaign was launched using the Neethling-based Lumpivax^TM^ vaccine^47,48^. The Neethling-based vaccine has been extensively used in many regions globally and has demonstrated effectiveness in preventing LSDV infections in cattle^6^. However, the Lumpivax^TM^ vaccine is not a pure Neethling-based LSDV vaccine but a complex mixture of several CaPVs^47^. The emergence of LSDV recombination was reported in Russia in 2018^28,49^. Recombinant strains resembling vaccines were found in multiple East and Southeast Asian countries^50,51^. The spread of these recombinant viruses was attributed to a contaminated vaccine^47^. Since the initial outbreak of LSDV in 2019, China has utilized a GTPV attenuated live vaccine for emergency immunization, which has somewhat curbed the epidemic’s spread. However, it hasn’t entirely prevented the virus’s dissemination. In East and Southeast Asian countries like China, Vietnam, and Thailand, LSDV strains belong to Clade 2.2, which is a recombinant virus between Clade 1.1 and Clade 2.1(Fig. 2b). These strains showed a high correlation with recombinant strains found in Russia and Middle Eastern countries, suggesting a possible transmission route from the Middle East to Asia. However, the phylogenetic analysis indicates that the prevalent viral strains in India belong to two distinct branches, Clade 1.1 and Clade 1.2, rather than the recombinant viral Clade 2.2. The pathway through which LSDV spread to South Asian countries remains unknown^13^. This indicates the existence of multiple lineages or strains of LSDV circulating in different regions of Asia. This geographical distribution suggested that LSDV strains within these clades had undergone local adaptation and potentially developed unique genetic signatures specific to their respective regions. LSD exhibited a significant impact on animal populations, infecting over 2 million animals and leading to approximately 100,000 deaths in India^52^, along with 190,000 cases and more than 7,500 deaths in Pakistan^12^. However, in China, the total morbidity and mortality in the eight outbreaks were 19.5% (156/801) and 0.9% (7/801), respectively^31^. While differences in virulence among prevalent viral strains may contribute to these variations, it is essential to acknowledge that the diversity in pathogenicity is likely influenced by a multitude of factors. These factors include, but are not limited to, overall animal health, vector density, animal density, and the level of herd immunity. Therefore, it is crucial to consider these multifactorial aspects when evaluating the impact of LSDV in different regions. Most Asian countries haven’t introduced the Neethling vaccine strain but have implemented various alternative control measures against LSD. India developed a new vaccine based on isolated strains, while China conducted emergency vaccinations using a weakened GTPV vaccine^53^. In 2023, South Korea and Libya reported their first LSD cases, highlighting the widening extent of LSDV transmission^15^.

LSDV primarily affects cattle and other ruminant animals, demonstrating a remarkably limited host range^54^. In addition, LSDV acts as a limited replication vaccine vector across various animal models such as mice, rabbits, hamsters, and Macaques (Supplementary Table 7). We conducted the inaugural evaluation of LSDV growth capabilities in BSC-1, DF-1, and HFF cells. Our study marks the first confirmation that LSDV can replicate within BSC-1, A549, and HFF cells. This observation contradicts the outcomes reported by Chunling Ma^55^. We repeated our experiments using A549 cell lines preserved in three different laboratories and consistently observed the same result. We observed mature virus particles within A549 and HFF cells (Fig. 4i) and we are confident with our results. Given the variability in the clinical strains that were isolated, conducting further experimental comparisons may yield intriguing insights. Additionally, although LSDV exhibits replication capability on the chorioallantoic membrane of chicken embryos, it did not replicate successfully in chicken fibroblast DF-1 cells. While LSDV can proliferate in various lamb cells, including Primary Lamb Testis Cells (LT), Lamb Kidney Cells (LK), Lamb Heart Cells, Lamb Skin Cells, OA3.Ts, and ESH-L cell lines (Supplementary Table 8)^56^, it seldom induces infections in sheep and goats under clinical conditions. While we have observed the potential for LSDV replication in A549 cells, it’s important to emphasize that LSDV is not recognized for infecting humans or causing diseases in them. There is no evidence supporting animal-to-human transmission of LSDV.

In conclusion, our findings illuminate the presence of an ‘open’ pan-genome within LSDV, offering invaluable insights into the genetic diversity and intricate evolutionary trajectories of the virus. Phylogenetic analysis unveils distinct virus lineages actively circulating in Asia. Furthermore, cellular tropism studies underscore LSDV’s capability to infect a wide array of mammalian cells, including human A549 cells. Consequently, we underscore the necessity and importance of bolstering LSD control measures and continuous epidemiological surveillance. This knowledge lays the theoretical groundwork for studying the genetic characteristics, phenotypic variations, host range, and virulence of LSDV, thereby contributing to the development of targeted interventions tailored for LSDV outbreaks in specific geographic areas.

## Methods

### Sample collection

Skin samples were collected from cattle affected by LSDV during the 2021 and 2022 outbreaks in Fujian and Heilongjiang provinces in China. Approximately 200 mg of tissue was ground using mortar and quartz sand. The suspended sand was then eliminated by adding 5 ml of PBS containing 10% penicillin-streptomycin. Madin-Darby bovine kidney (MDBK) cells were infected with the tissue supernatants for 2 hours. The supernatants were collected by centrifugation at 2000 g for 10 minutes after three cycles of freeze-thaw. Following a 72-hour culture at 37 °C with CO_2_, both the cells and virus DNA were extracted.

To detect the virus, the primers LSDV126-F (ATGGGAATAGTATCTGTTGTATACG) and LSDV126-R (CGAACCCCTATTTACTTGAGAA) were used with an annealing temperature of 56 °C for 35 cycles^57^. Next-generation sequencing was performed using a Novaseq PE150 (Illumina) platform by Beijing Tsingke Biotech Co., Ltd.

### Genome Sequencing, Assembly, and Annotation

Adapter sequences and low-quality reads were automatically trimmed using Fastp v0.20.0^58^. The reads were mapped to the goat genome by BBmap v38.5185 to remove the host sequence. Two de novo genome assembly tools were used in this study: SPAdes v3.14.1^59^ and SOAPdenovo v2.04^60^ with default parameters. 152 capripoxvirus genome sequences were obtained from NCBI, and the information, particularly regarding virulence, was manually verified for each strain. The strain data can be found in Supplementary Table 4. The genomes were initially incompletely annotated but were re-annotated using Prokka v1.14.6^61^ with AF325528.1 serving as the reference. A comparison between different annotation methods was conducted using Blastp (Supplementary Table 2)^62^, and the results were visualized using Circoletto (Supplementary Fig. 2)^63^. Functional annotation of predicted proteins from capripoxvirus was conducted with eggNOG v5.0^64^, which provides a framework for automated functional annotation of proteins based on their sequence similarity to annotated genes in the database. Gene Ontology (GO) analysis using the R package ClusterProfiler v4.0^65^ to identify enriched biological themes, pathways, and molecular functions. Virulence factors and toxins were predicted by VFDB^32^, Victors^33^, and Pathofact^34^. Scoary^36^ was used for the Pan-genome-wide association study (Pan-GWAS) analysis. It was used to analyze the association between the presence of certain genes and the virulence, regions, and lineages of LSDV.

### Pan-genome inference

Detection of core and accessory genes was performed using the Roary software^66^. Genes present in all analyzed LSDV genomes were classified as core genes, while genes not present in all viral genomes were considered accessory genes. The definition of the core gene set depended on the percentage identity of sequences, and thus the Roary pan-genome pipeline was executed multiple times with different identity thresholds (ranging from 75% to 90% with 5% increments). A comparison of the results revealed no significant difference in the number of core gene clusters between the 75% and 90% identity thresholds, leading to the selection of the 90% identity threshold for further analysis. To estimate the size of the LSDV pan-genome, a variable number of strains (from 1 to 120, with increments of 1) were sampled from the dataset, and this process was repeated randomly for a total of 10 times for each number of strains. The R package “BestFitM” was utilized for curve fitting to determine the properties of the LSDV pan-genome. Result visualization was carried out using the R packages “ggtrendline” and “ggplot2”. The p-value of each regression model determined by the Wald test. For the analysis of 152 capripoxvirus genomes, the same methodology was applied, but a different identity threshold of 80% was utilized for subsequent analysis.

### Phylogenetic analysis

The coding regions of the core gene clusters were extracted from the Roary results. Multiple sequence alignment was performed using Clustal Omega^67^. To determine the best substitution model, ModelFinder^68^ was utilized based on the Bayesian information criterion(BIC). Subsequently, IQ-TREE^69^ was employed to construct a phylogenetic tree using the optimal substitution model, and bootstrapping was performed with 1000 replicates. The resulting phylogenetic tree was visualized using Evolview v3^70^, a platform specifically designed for displaying and analyzing evolutionary trees.

### Virus and recombinant virus construction

The recombinant virus LSDV-eGFP was constructed by inserting the eGFP-ORF into the region between LSDV049 and LSDV050 in the LSDV/FJ2021 strain^38^, under the control of the late promoter p11 (TTTCATTTTGTTTTTTTCTATGCTATAA). Plasmid transfection and virus infection were carried out using BHK cells. After 48 hours, the virus was harvested, and the recombinant virus was purified using MDBK cells. The purification process involved using a DMEM medium(Solarbio, cat#11995) containing 0.5% carboxymethylcellulose sodium(Solarbio, cat#C8621) and 2.5% Donor Equine Serum(Biological Industries, cat#04-004-1 A).

### Cell infection and virus titer determination

MDBK[NBL-1](cat#CL-0153), BHK-21[C-13](cat#CL-0034), Vero(cat#CL-0242), UMNSAH/ DF-1(cat#CL-0279) were kindly provided by Procell Life Science&Technology Co.,Ltd. A549(cat#MZ-0015), HeLa(cat#MZ-3329), 293 T[HEK-293T](cat#MZ-0005), HFF(cat#MZ-1082), BS-C-1(cat#MZ-8048) were purchased from NingboMingZhoubioCO.,Ltd. Bovine testis (BT) cells^41^ and LSDV074 (H3L) antidody^40^ were generously provided by Dr. Chunsheng Yin (China Institute of Veterinary Drug Control, IVDC). Various cell lines were infected with a multiplicity of infection (MOI) of 0.01 or 3, and viruses were harvested at different time intervals. The collected samples underwent three cycles of repeated freezing and thawing. Ultrasound treatment was applied at 80 W for 15 seconds on and 15 seconds off for three cycles. After infecting MDBK cells for 72 hours, the virus titer was determined by calculating the plaque count using LSDV074 (H3L) antibody immunofluorescence. Western blot targeting the H3L gene was utilized to detect LSDV in MDBK and A549 cells at 12 h, 24 h, and 72 h post-infection. All blots or gels originated from the same experiment and were processed in parallel.

### Virion observation

After infecting A549, HFF, and MDBK cells with LSDV at 0.1 MOI and culturing for 48 hours, the samples were fixed using formaldehyde. Fixed samples were embeded in a resin to create a solid block for sectioning. Use an ultramicrotome to cut ultra-thin sections (around 50–100 nm) of the embedded sample. Transfer the ultra-thin sections onto a TEM grid. Apply heavy metal stains (e.g., uranyl acetate, lead citrate) to enhance contrast, making the virus particles more visible. Use the Transmission Electron Microscope (HITACHI HT7800) to visualize the virus particles.

### Reporting summary

Further information on research design is available in the Nature Research Reporting Summary linked to this article.

### Supplementary information

Supplemental Material

REPORTING SUMMARY

## Data Availability

All relevant data are within the manuscript and its Supporting Information files.
