# Supplementary material for: Genomic analysis of lumpy skin disease virus asian variants and evaluation of its cellular tropism
Source: NPJ Vaccines. 2024 Mar 21;9:65. doi: 10.1038/s41541-024-00846-8 (PMC10957905; doi:10.1038/s41541-024-00846-8)
Supplement: Supplementary file 1 — Supplemental Material [file 41541_2024_846_MOESM1_ESM.pdf]

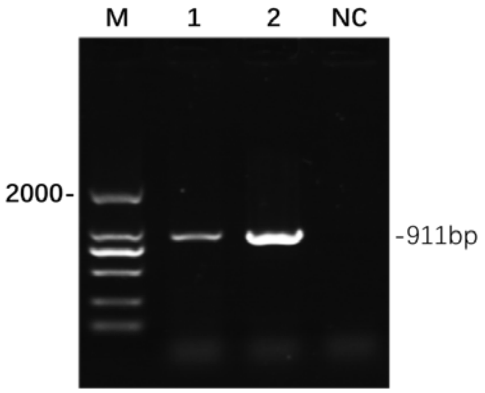

Supplementary Figure 1 PCR detection results for LSDV126 gene. M:2000 marker; 1: LSDV/FJ2021; 2:LSDV/HLJ2022; NC: negative control

Supplementary Table 1 The Illumina sequencing data statistics

Strains	Total_reads	Total_bases	Q20_bases	Q20_rate	Q30_bases	Q30_rate	Clean_reads	rm_rRNA	rm_host	rm_bacteria
**LSDV/FJ2021**	12217532	1832629800	1795186056	0.9796	1725981215	0.9418	6056607(100%)	6044704(99.80%)	1600565(26.43%)	1043374(17.23%)
**LSDV/HLJ2022**	33806732	5104816532	4983896163	0.9763	4763299888	0.9331	16771795(100%)	16731326(99.76%)	4907763(29.26%)	808760(4.82%)

Supplementary Table 2 The result of AF325528 annotation with different methods GCG and prokka

AF325528_prokka	AF325528_GCG	% identity	AF325528_prokka	AF325528_GCG	% identity
LSDV00001	LSDV001	100	LSDV00085	LSDV087	31.325
LSDV00001	LSDV156	100	LSDV00086	LSDV086	31.325
LSDV00002	LSDV002	100	LSDV00086	LSDV087	100
LSDV00002	LSDV155	100	LSDV00087	LSDV088	100
LSDV00003	LSDV003	100	LSDV00088	LSDV089	100
LSDV00003	LSDV154	100	LSDV00089	LSDV090	100
LSDV00004	LSDV005	100	LSDV00090	LSDV091	100
LSDV00005	LSDV006	100	LSDV00091	LSDV092	100
LSDV00005	LSDV013	25.472	LSDV00092	LSDV093	100
LSDV00005	LSDV135	23.762	LSDV00093	LSDV094	100
LSDV00006	LSDV007	100	LSDV00094	LSDV095	100
LSDV00007	LSDV008	100	LSDV00095	LSDV096	100
LSDV00008	LSDV009	100	LSDV00096	LSDV097	100
LSDV00009	LSDV010	100	LSDV00097	LSDV098	100
LSDV00010	LSDV011	100	LSDV00098	LSDV099	100
LSDV00011	LSDV012	100	LSDV00099	LSDV100	100

LSDV00011	LSDV145	25.694	LSDV00100	LSDV101	100
LSDV00011	LSDV147	24.59	LSDV00101	LSDV102	100
LSDV00011	LSDV148	39	LSDV00102	LSDV103	100
LSDV00012	LSDV006	25.472	LSDV00103	LSDV104	100
LSDV00012	LSDV013	100	LSDV00104	LSDV105	100
LSDV00013	LSDV014	100	LSDV00105	LSDV107	100
LSDV00014	LSDV015	100	LSDV00106	LSDV108	100
LSDV00015	LSDV016	100	LSDV00107	LSDV109	100
LSDV00016	LSDV017	100	LSDV00108	LSDV110	100
LSDV00017	LSDV018	100	LSDV00109	LSDV111	100
LSDV00018	LSDV019	100	LSDV00110	LSDV113	100
LSDV00018	LSDV144	29.189	LSDV00111	LSDV112	100
LSDV00018	LSDV144	23.45	LSDV00112	LSDV114	100
LSDV00018	LSDV151	24.217	LSDV00113	LSDV115	100
LSDV00019	LSDV020	100	LSDV00114	LSDV116	100
LSDV00020	LSDV021	100	LSDV00115	LSDV117	100
LSDV00021	LSDV022	100	LSDV00116	LSDV118	100
LSDV00022	LSDV023	100	LSDV00117	LSDV119	100
LSDV00023	LSDV024	100	LSDV00118	LSDV120	100
LSDV00023	LSDV060	29.661	LSDV00120	LSDV121	100
LSDV00024	LSDV025	100	LSDV00121	LSDV122	100
LSDV00025	LSDV026	100	LSDV00122	LSDV123	100
LSDV00026	LSDV027	100	LSDV00123	LSDV124	100
LSDV00027	LSDV028	100	LSDV00124	LSDV125	100
LSDV00027	LSDV146	29.016	LSDV00125	LSDV126	100
LSDV00029	LSDV029	100	LSDV00126	LSDV127	100
LSDV00030	LSDV030	100	LSDV00127	LSDV128	100
LSDV00031	LSDV031	100	LSDV00128	LSDV129	100
LSDV00032	LSDV032	100	LSDV00129	LSDV130	100
LSDV00033	LSDV033	100	LSDV00130	LSDV131	100
LSDV00034	LSDV034	100	LSDV00131	LSDV132	100
LSDV00035	LSDV036	100	LSDV00132	LSDV133	100
LSDV00036	LSDV035	100	LSDV00133	LSDV134	100
LSDV00037	LSDV037	100	LSDV00134	LSDV006	23.762
LSDV00038	LSDV038	100	LSDV00134	LSDV135	100
LSDV00039	LSDV039	100	LSDV00135	LSDV136	100
LSDV00040	LSDV040	100	LSDV00136	LSDV137	100
LSDV00041	LSDV041	100	LSDV00137	LSDV138	100
LSDV00042	LSDV042	100	LSDV00138	LSDV139	100
LSDV00043	LSDV043	100	LSDV00139	LSDV140	100
LSDV00044	LSDV044	100	LSDV00140	LSDV141	100
LSDV00045	LSDV045	100	LSDV00141	LSDV142	100
LSDV00046	LSDV046	100	LSDV00142	LSDV143	100

LSDV00047	LSDV047	100	LSDV00143	LSDV019	29.189
LSDV00048	LSDV048	100	LSDV00143	LSDV144	100
LSDV00049	LSDV049	100	LSDV00143	LSDV151	39.535
LSDV00050	LSDV050	100	LSDV00144	LSDV012	25.694
LSDV00051	LSDV052	100	LSDV00144	LSDV145	100
LSDV00052	LSDV051	100	LSDV00144	LSDV147	29.675
LSDV00053	LSDV053	100	LSDV00144	LSDV147	32.051
LSDV00054	LSDV054	100	LSDV00144	LSDV148	30.5
LSDV00055	LSDV055	100	LSDV00144	LSDV152	26.889
LSDV00056	LSDV056	100	LSDV00145	LSDV028	29.016
LSDV00057	LSDV057	100	LSDV00145	LSDV146	100
LSDV00058	LSDV058	100	LSDV00146	LSDV012	24.59
LSDV00059	LSDV059	100	LSDV00146	LSDV145	29.675
LSDV00060	LSDV024	29.661	LSDV00146	LSDV145	32.051
LSDV00060	LSDV060	100	LSDV00146	LSDV147	100
LSDV00061	LSDV061	100	LSDV00146	LSDV148	26.136
LSDV00062	LSDV062	100	LSDV00146	LSDV152	25.745
LSDV00063	LSDV063	100	LSDV00147	LSDV012	39
LSDV00064	LSDV064	100	LSDV00147	LSDV145	30.5
LSDV00065	LSDV065	100	LSDV00147	LSDV147	26.136
LSDV00066	LSDV066	100	LSDV00147	LSDV148	100
LSDV00067	LSDV067	100	LSDV00147	LSDV152	27.358
LSDV00068	LSDV068	100	LSDV00148	LSDV149	100
LSDV00069	LSDV070	100	LSDV00149	LSDV150	100
LSDV00070	LSDV071	100	LSDV00150	LSDV019	24.217
LSDV00071	LSDV072	100	LSDV00150	LSDV144	40.502
LSDV00072	LSDV073	100	LSDV00150	LSDV151	100
LSDV00073	LSDV074	100	LSDV00151	LSDV012	31.858
LSDV00074	LSDV075	100	LSDV00151	LSDV145	26.444
LSDV00075	LSDV076	100	LSDV00151	LSDV147	25.745
LSDV00076	LSDV077	100	LSDV00151	LSDV148	28.103
LSDV00077	LSDV078	100	LSDV00151	LSDV152	100
LSDV00078	LSDV079	100	LSDV00152	LSDV004	100
LSDV00079	LSDV080	100	LSDV00152	LSDV153	100
LSDV00080	LSDV081	100	LSDV00153	LSDV003	100
LSDV00081	LSDV082	100	LSDV00153	LSDV154	100
LSDV00082	LSDV083	100	LSDV00154	LSDV002	100
LSDV00083	LSDV084	100	LSDV00154	LSDV155	100
LSDV00084	LSDV085	100	LSDV00155	LSDV001	100
LSDV00085	LSDV086	100	LSDV00155	LSDV156	100

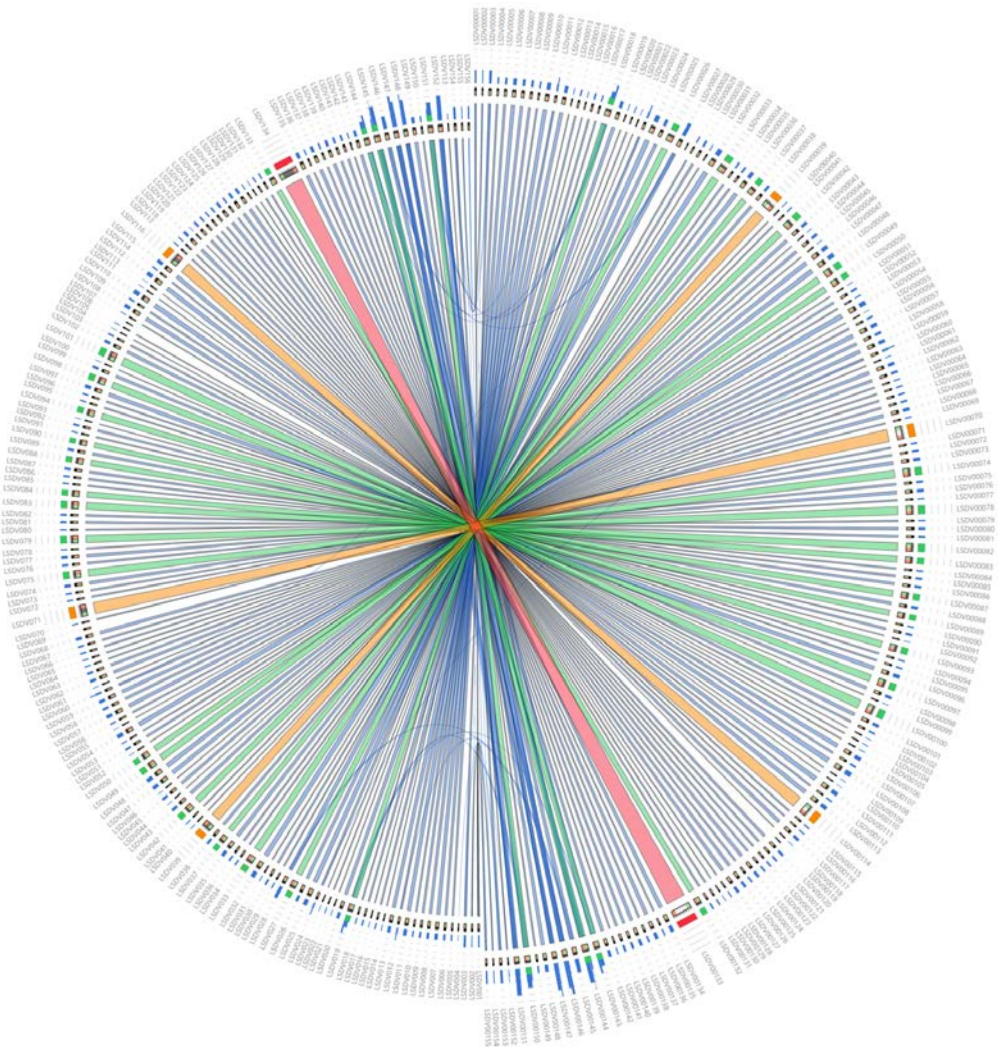

Supplementary Figure 2 The blast result of AF325528 between the different methods Prokka and GCG v.10. Figure produced by Circoletto. The ribbons represent the local alignments produced by BLAST, their width the alignment length, and the colors of the alignment bit scores in four quartiles: blue for the first (i.e. worst) 25% of the maximum bit score, green for the next 25%, orange for the third, and finally red for the top (i.e. best) bit scores of between 75% and 100% of the maximum bit scores.

Supplementary Table 3 The difference in annotation results between Prokka and GCG

	GCG	Prokka	aa	Function
ORFs	156	155		
Different gene	LSDV069		185aa	RNA polymerase subunit
	LSDV106		53aa	putative factor
		LSDV00028	48aa	hypothetical protein
		LSDV00119	41aa	hypothetical protein

Supplementary Table 4 **Capripoxviruses genomes used in this study**

No.	GenBank	Virus	Strain	Field/Vaccine-Associated	Country	Region	Reference
1	KC951854	GTPV	FZ	Field	China	Asia	1
2	MH381810	GTPV	AV41	Vaccine-Associated	China	Asia	
3	MW020570	GTPV	V103	Field	Germany	Europe	2
4	KX576657	GTPV	Gorgan	Vaccine-Associated	Jordan	Middle East	3
5	MN072620	GTPV	India	Field	India	Asia	4
6	AY077835	GTPV	Pellor	Field	Kazakhstan	Middle East	5
7	AY077836	GTPV	G20-LKV	Vaccine-Associated	Kazakhstan	Middle East	5
8	MN072623	GTPV	Oman	Field	Oman	Middle East	4
9	MN072624	GTPV	Sudan	Field	Sudan	Africa	4
10	MN072622	GTPV	Turkey	Field	Turkey	Middle East	4
11	MN072621	GTPV	Vietnam	Field	Vietnam	Asia	4
12	MN072625	GTPV	Yemen	Field	Yemen	Middle East	4
13	KT438550	SPPV	SPPV-GH	Field	China	Asia	
14	KT438551	SPPV	SPPV-GL	Field	China	Asia	
15	MW020571	SPPV	V104	Field	Germany	Europe	2
16	MW167070	SPPV	V123	Field	Germany	Europe	6
17	MW167071	SPPV	V293	Field	Germany	Europe	6
18	MG000156	SPPV	Jaipur	Field	India	Asia	
19	MG000157	SPPV	RomanianFenner	Field	India	Asia	
20	MT137384	SPPV	Srinagarpassage-40vaccine	Vaccine-Associated	India	Asia	
21	MN072626	SPPV	Abu Gharib	Vaccine-Associated	Iraq	Middle East	4
22	AY077833	SPPV	SA	Field	Kazakhstan	Middle East	5
23	AY077834	SPPV	NISKHI	Vaccine-Associated	Kazakhstan	Middle East	5
24	MN072628	SPPV	NIG-SPPV	Field	Nigeria	Africa	4
25	ON961655	SPPV	Moscow	Field	Russia	Europe	7
26	ON961656	SPPV	Moscow	Field	Russia	Europe	7
27	ON961657	SPPV	Tula	Field	Russia	Europe	7
28	MN072627	SPPV	Saudi Arabia vaccine	Vaccine-Associated	Saudi Arabia	Middle East	4
29	MN072630	SPPV	SAU-SPPV	Field	Saudi Arabia	Middle East	4
30	MN072629	SPPV	Pendik SPPV	Vaccine-Associated	Turkey	Middle East	4
31	AY077832	SPPV	TU-V02127	Field	Turkey	Middle East	
32	MN072631	SPPV	Turkey SPPV vaccine	Vaccine-Associated	Turkey	Middle East	4
33	AF325528	LSDV	NI-2490	Field	Kenya	Africa	8
34	AF409137	LSDV	NW-LW	Field	SouthAfrica	Africa	9
35	AF409138	LSDV	LW1959	Vaccine-Associated	SouthAfrica	Africa	9
36	KX683219	LSDV	KSGP0240	Vaccine-Associated	Jordan	Middle East	10
37	KX764643	LSDV	SIS-Lumpyvax	Vaccine-Associated	SouthAfrica	Africa	11
38	KX764644	LSDV	Neethling-Herbivac	Vaccine-Associated	SouthAfrica	Africa	11
39	KX764645	LSDV	Neethling-LSDvaccine-OBP	Vaccine-Associated	SouthAfrica	Africa	11

No.	GenBank	Virus	Strain	Field/Vaccine-Associated	Country	Region	Reference
40	KX894508	LSDV	155920/2012	Field	Israel	Middle East	
41	KY702007	LSDV	SERBIA/Bujanovac/2016	Field	Serbia	Europe	[12]
42	KY829023	LSDV	Evros/GR/15	Field	Greece	Europe	[13]
43	MG972412	LSDV	Cro2016	Vaccine-Associated	Croatia	Europe	[14]
44	MH646674	LSDV	Russia/Saratov/2017	Field	Russia	Europe	[15]
45	MH893760	LSDV	Russia/Dagestan/2015	Field	Russia	Europe	[16]
46	MK441838	LSDV	HerbivacLS	Vaccine-Associated	SouthAfrica	Africa	[17]
47	MN072619	LSDV	Kenya	Field	Kenya	Africa	[4]
48	MN636838	LSDV	LSD-58-LP-RSA-1993	Vaccine-Associated	SouthAfrica	Africa	[18]
49	MN636839	LSDV	LSD-103-GP-RSA-1991	Vaccine-Associated	SouthAfrica	Africa	[18]
50	MN636840	LSDV	LSD-248-NW-RSA-1993	Vaccine-Associated	SouthAfrica	Africa	[18]
51	MN636841	LSDV	LSD-220-1-NW-RSA-1993	Vaccine-Associated	SouthAfrica	Africa	[18]
52	MN636842	LSDV	LSD-220-2-NW-RSA-1993	Vaccine-Associated	SouthAfrica	Africa	[18]
53	MN636843	LSDV	LSD-148-GP-RSA-1997	Vaccine-Associated	SouthAfrica	Africa	[18]
54	MN642592	LSDV	Kubash/KAZ/16	Field	Kazakhstan	Middle East	[19]
55	MN995838	LSDV	pendik	Field	Trukey	Middle East	
56	MT007950	LSDV	Namibia_2016_9F	Field	Namibian	Africa	[20]
57	MT130502	LSDV	Neethling-RIBSP	Vaccine-Associated	Kazakhstan	Middle East	[21]
58	MT134042	LSDV	LSDV/Russia/Udmurtiya/2019	Field	Russia	Europe	[22]
59	MT643825	LSDV	210LSD-249/BUL/16	Field	Bulgaria	Europe	[23]
60	MT992618	LSDV	KZ-Kostanay-2018	Field	Kazakhstan	Middle East	
61	MW030512	LSDV	Neethling-RIBSP(TK-)EGFP	Field	Kazakhstan	Middle East	
62	MW355944	LSDV	China/GD01/2020	Field	China	Asia	[24]
63	MW435866	LSDV	SA-Neethling	Vaccine-Associated	SouthAfrica	Africa	[25]
64	MW631933	LSDV	LSD	Field	Morocco	Middle East	[26]
65	MW656252	LSDV	Haden/RSA/1954	Vaccine-Associated	SouthAfrica	Africa	[27]
66	MW656253	LSDV	280-KZN/RSA/2018	Field	SouthAfrica	Africa	[27]
67	MW699032	LSDV	Russia/Dagestan/2015 75passage	Field	Russia	Europe	[27]
68	MW732649	LSDV	HongKong/2020	Field	China	Asia	[28]
69	MW883897	LSDV	Cattle/India/2019/Ranchi-1	Field	India	Asia	[29]
70	MZ577073	LSDV	20L42_Quyet-Thang/VNM/20	Field	Vietnam	Asia	[30]
71	MZ577074	LSDV	20L43_Ly-Quoc/VNM/20	Field	Vietnam	Asia	[30]
72	MZ577075	LSDV	20L70_Dinh-To/VNM/20	Field	Vietnam	Asia	[30]
73	MZ577076	LSDV	20L81_Bang-Thanh/VNM/20	Field	Vietnam	Asia	[30]
74	OK318001	LSDV	V281	Field	Nigeria	Africa	[31]
75	OK422492	LSDV	Cattle/India/2019/Ranchi-1/P10	Field	India	Asia	[29]
76	OK422493	LSDV	Cattle/India/2019/Ranchi-	Field	India	Asia	[29]

No.	GenBank	Virus	Strain	Field/Vaccine-Associated	Country	Region	Reference
			1/P30				
77	OK422494	LSDV	Cattle/India/2019/Ranchi-1/P50	Field	India	Asia	[29]
78	OL542833	LSDV	Russia/Tyumen/2019	Field	Russia	Europe	[32]
79	OL752713	LSDV	KM/Taiwan/2020	Field	China	Asia	[33]
80	OM033705	LSDV	LSDV/Thailand/YST/2021	Field	Thailand	Asia	[34]
81	OM530217	LSDV	Russia/Saratov/2019	Field	Russia	Europe	[35]
82	OM793602	LSDV	Russia_Tomsk_2020	Field	Russia	Europe	[32]
83	OM793603	LSDV	Russia_Khabarovsk_2020	Field	Russia	Europe	[32]
84	OM984485	LSDV	XJ201901	Field	China	Asia	[36]
85	OM984486	LSDV	FJ2019	Field	China	Asia	[36]
86	ON005067	LSDV	Atyrau-5BJN(IL-18)	Field	Kazakhstan	Middle East	[37]
87	ON010590	LSDV	Neethling-RIBSP/7C	Field	Kazakhstan	Middle East	
88	ON152411	LSDV	LSDV72/PrachuapKhiriKhan/Thailand/2021	Field	Thailand	Asia	[38]
89	ON400507	LSDV	208/PVNRTVU/2020	Field	India	Asia	[39]
90	ON616408	LSDV	NMG/2020	Field	China	Asia	[40]
91	OP297402	LSDV	LSDV-WB/IND/19	Field	India	Asia	[41]
92	OP508345	LSDV	China/Xinjiang/Cattle/Aug-2019	Field	China	Asia	
93	OP688128	LSDV	V392.1	Field	Bangladesh	Asia	[42]
94	OP688129	LSDV	V395.1	Field	Bangladesh	Asia	[42]
95	OP922506	LSDV	FJ2021	Field	China	Asia	This study
96	OM105589	LSDV	XJ01/2019	Field	China	Asia	[43]
97	OM373209	LSDV	LSDV_BH3/CHN/20	Field	China	Asia	
98	OM803091	LSDV	GD02/2020	Field	China	Asia	[44]
99	OM803092	LSDV	China/GX01/2020	Field	China	Asia	
100	OQ555660	LSDV	HLJ2022	Field	China	Asia	This study
101	OM793609	LSDV	LSDV_Vaccine_LW-1959_1988	Vaccine-Associated	SouthAfrica	Africa	
102	OM793608	LSDV	LSDV_Neethling-WC_RSA_1957	Vaccine-Associated	SouthAfrica	Africa	
103	OM793606	LSDV	LSDV_Potter_RSA_1958	Vaccine-Associated	SouthAfrica	Africa	
104	OM793607	LSDV	LSDV_Fourie-FS_RSA_1959	Vaccine-Associated	SouthAfrica	Africa	
105	OM793604	LSDV	LSDV_33-KZN_RSA_1977	Vaccine-Associated	SouthAfrica	Africa	
106	OM793605	LSDV	LSDV_Hoffmeyer_RSA_1958	Vaccine-Associated	SouthAfrica	Africa	
107	OP654649	LSDV	LSDV/China/SiC/2021	Field	China	Asia	
108	OP752701	LSDV	LSDV/FJ/CHA/2021	Field	China	Asia	
109	OP985536	LSDV	LSDV/MZGD/2020/China	Field	China	Asia	
110	OQ267777	LSDV	65F12959	Field	Thailand	Asia	

No.	GenBank	Virus	Strain	Field/Vaccine-Associated	Country	Region	Reference
111	OQ267778	LSDV	119607/64	Field	Thailand	Asia	
112	OQ349695	LSDV	LSDV/Thailand/PraChuapKhiriKhan/2021	Field	Thailand	Asia	
113	OQ427097	LSDV	LSDV_West_Bengal_2022	Field	India	Asia	
114	OQ511520	LSDV	LSDV/Thailand/Trang/2022	Field	Thailand	Asia	
115	OQ588787	LSDV	LSDV/02/KASH/IND/2022	Field	India	Asia	
116	OQ606832	LSDV	LSDV/IND-2022/NIAB-PVNRTVU	Field	India	Asia	
117	OR134832	LSDV	LSDV/Albania/1000/2017	Field	Albania	Europe	
118	OR134833	LSDV	LSDV/Albania/1573/2016	Field	Albania	Europe	
119	OR134834	LSDV	LSDV/Albania/1707/2016	Field	Albania	Europe	
120	OR134835	LSDV	LSDV/Albania/4192/2016	Field	Albania	Europe	
121	OR134836	LSDV	LSDV/Albania/4770/2016	Field	Albania	Europe	
122	OR134837	LSDV	LSDV/Albania/790/2017	Field	Albania	Europe	
123	OR134838	LSDV	LSDV/Greece/314/2016	Field	Greece	Europe	
124	OR134839	LSDV	LSDV/Greece/386-16/2016	Field	Greece	Europe	
125	OR134840	LSDV	LSDV/Greece/478/2016	Field	Greece	Europe	
126	OR134841	LSDV	LSDV/Greece/498A/2016	Field	Greece	Europe	
127	OR134842	LSDV	LSDV/Greece/715/2015	Field	Greece	Europe	
128	OR134843	LSDV	LSDV/North_Macedonia/5000/2016	Field	Macedonia	Europe	
129	OR134844	LSDV	LSDV/North_Macedonia/5011/2016	Field	Macedonia	Europe	
130	OR134845	LSDV	LSDV/Serbia/4592/2016	Field	Serbia	Europe	
131	OR134846	LSDV	LDSV/Serbia/5887/2016	Field	Serbia	Europe	
132	OR134847	LSDV	LSDV/Serbia/6040/2016	Field	Serbia	Europe	
133	OR134848	LSDV	LSDV/Serbia/6402/2016	Field	Serbia	Europe	
134	OR134849	LSDV	LSDV/Serbia/7695/2016	Field	Serbia	Europe	
135	OR194148	LSDV	LSDV/Kurgan/2018	Field	Russia	Europe	
136	OR347834	LSDV	138156/64	Field	Thailand	Asia	
137	OR347835	LSDV	65A01032	Field	Thailand	Asia	
138	OR347836	LSDV	65A09027	Field	Thailand	Asia	
139	OR347837	LSDV	65A09679	Field	Thailand	Asia	
140	OR393169	LSDV	LSDV/2022/Jamnagar/N3	Field	India	Asia	
141	OR393170	LSDV	LSDV/2022/Surat/N6	Field	India	Asia	
142	OR393171	LSDV	LSDV/2022/Surat/N7	Field	India	Asia	
143	OR393172	LSDV	LSDV/2022/Anand/N8	Field	India	Asia	
144	OR393173	LSDV	LSDV/2022/Anand/N9	Field	India	Asia	
145	OR393174	LSDV	LSDV/2019/Ranchi/P50	Field	India	Asia	
146	OR393175	LSDV	LSDV/2021/Banswara	Field	India	Asia	
147	OR393176	LSDV	LSDV/2022/Camel	Field	India	Asia	
148	OR393177	LSDV	LSDV/2022/Jalore	Field	India	Asia	
149	OR393178	LSDV	LSDV/2022/Nohar	Field	India	Asia	

No.	GenBank	Virus	Strain	Field/Vaccine-Associated	Country	Region	Reference
150	OR520147	LSDV	LSD N1 SKUAST	Field	India	Asia	
151	OR567413	LSDV	LSDV/Jiling/2022	Field	China	Asia	
152	OR735987	LSDV	65A14015	Field	Thailand	Asia	

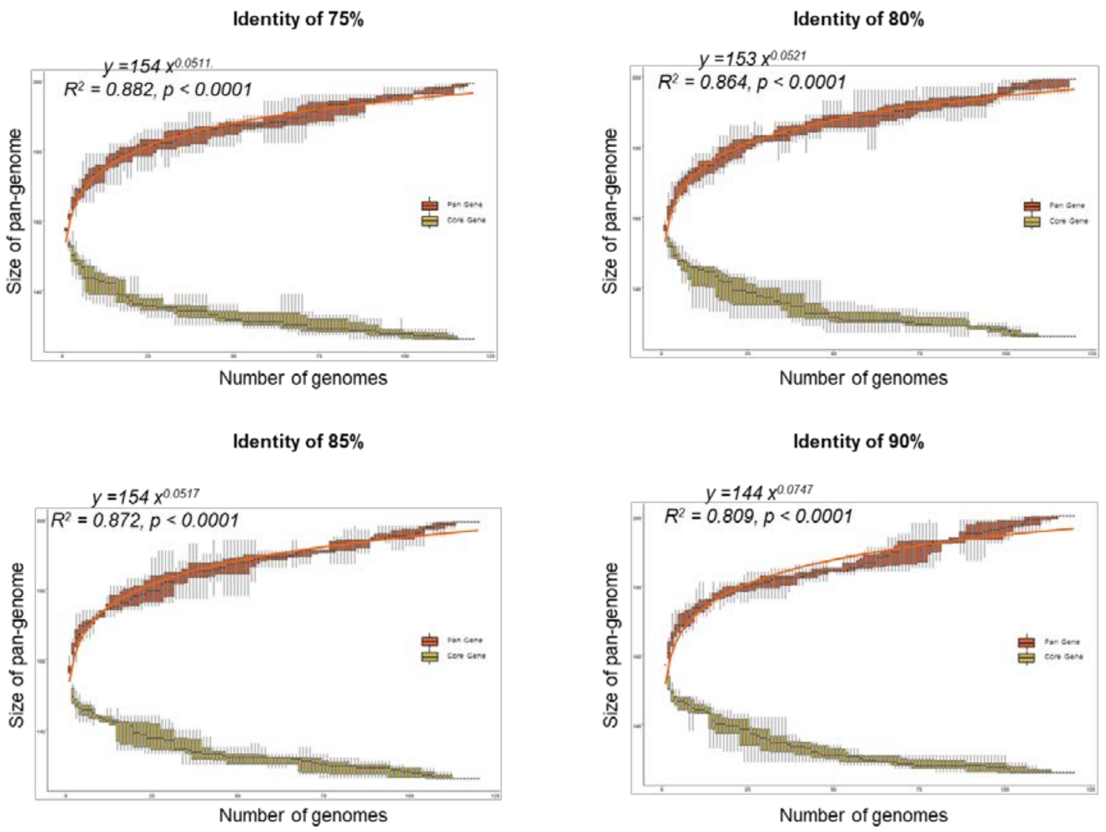

Supplementary Figure 3 Lumpy skin disease virus pan-genome size estimation with different identities from 75% to 90%.

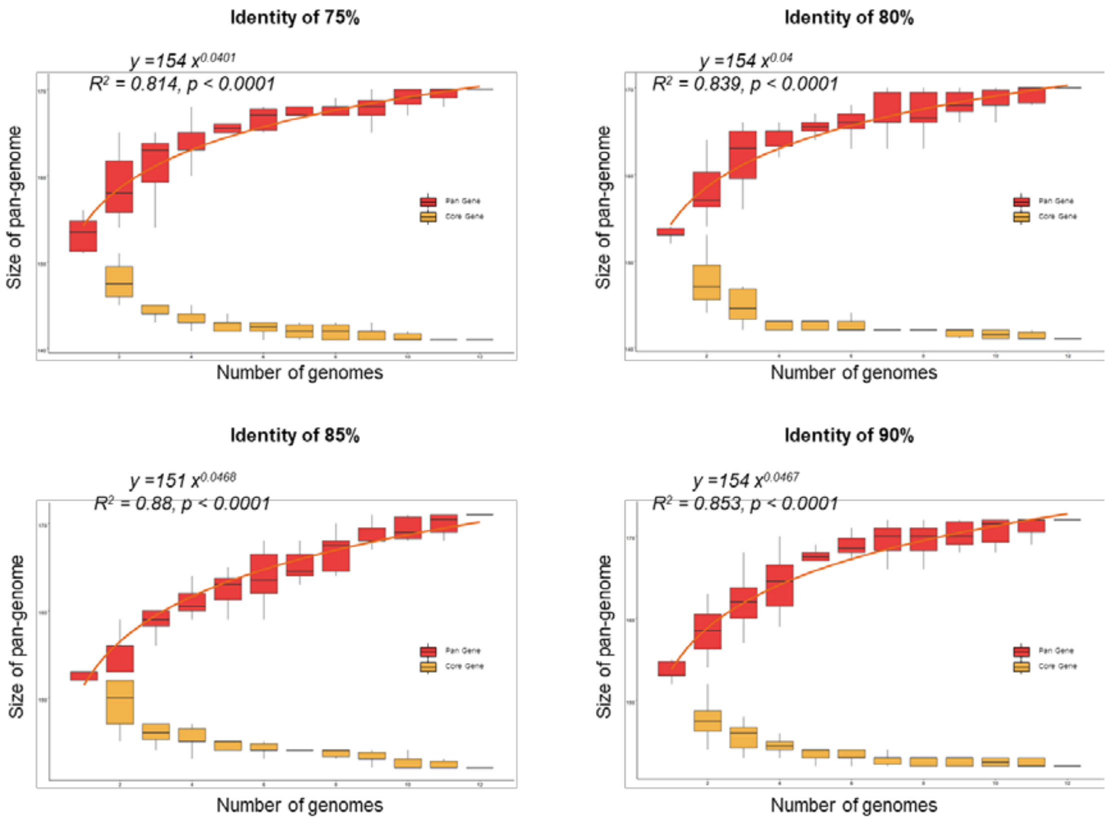

Supplementary Figure 4 Goatpox virus pan-genome size estimation with different identities from 75% to 90%.

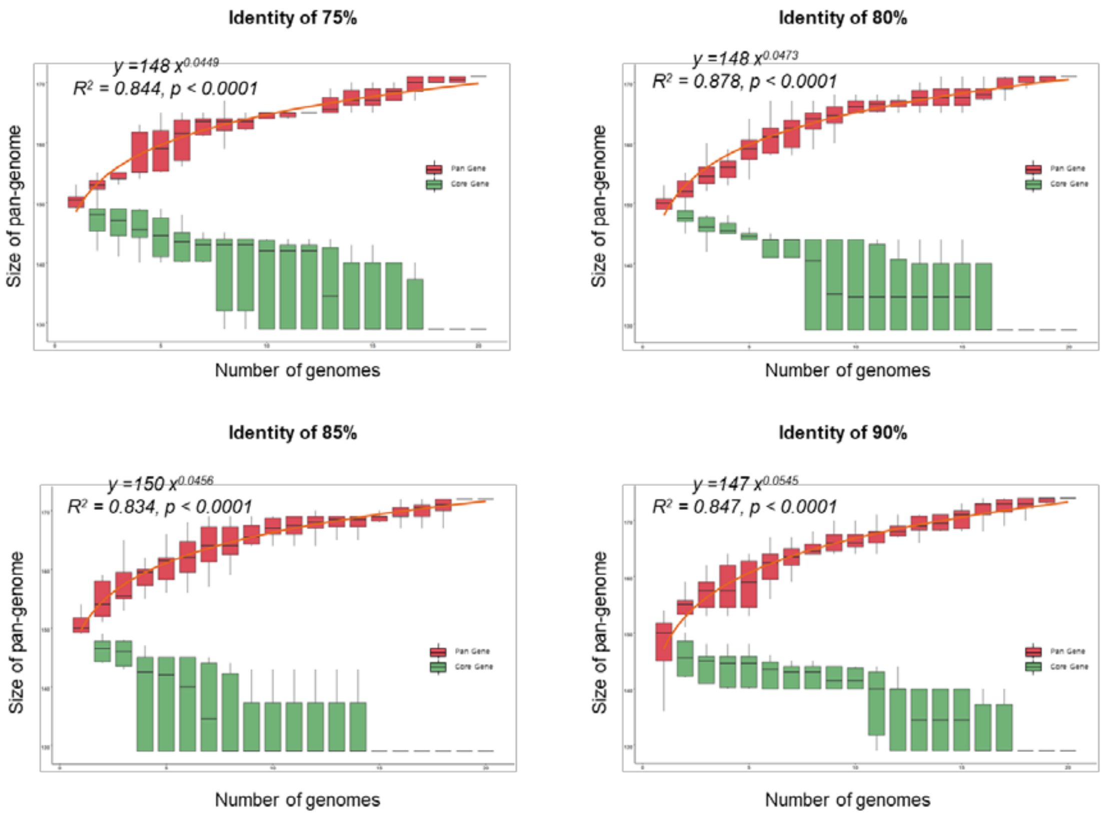

Supplementary Figure 5 Sheeppox virus pan-genome size estimation with different identities

from 75% to 90%.

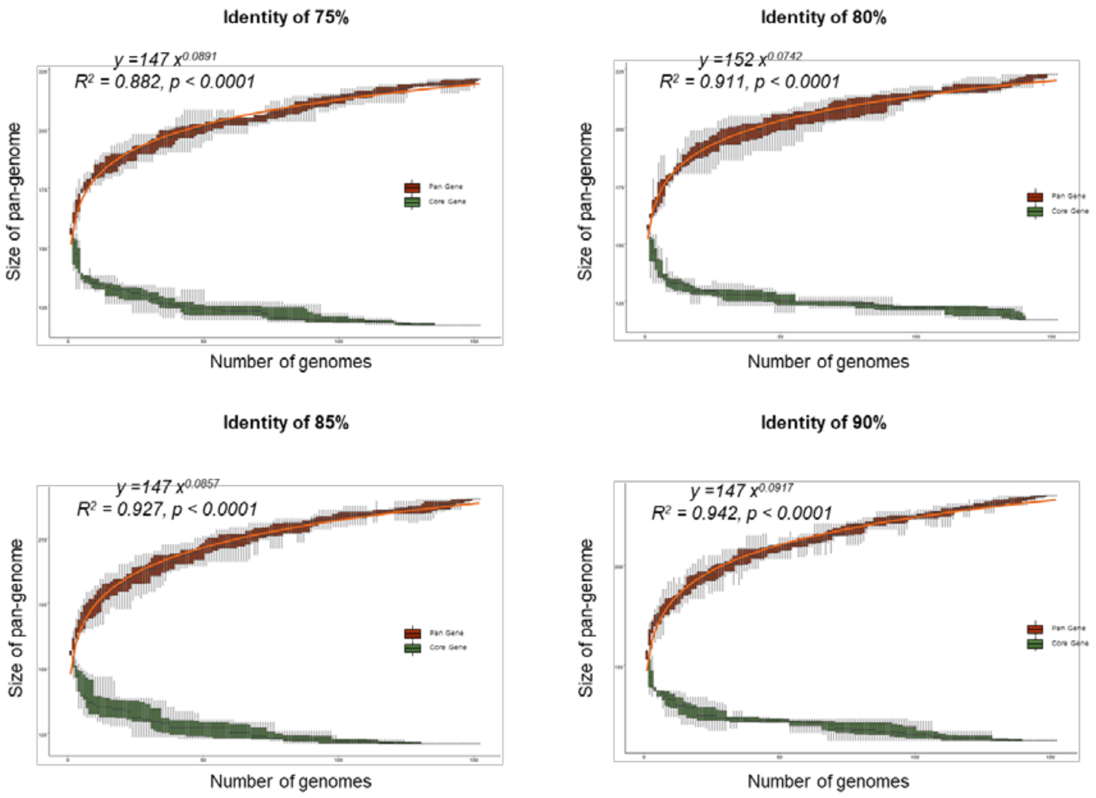

Supplementary Figure 6 Capripoxvirus pan-genome size estimation with different identities from 75% to 90%.

Supplementary Table 5 126 core genes of LSDV genome

LSDV00008	LSDV00034	LSDV00056	LSDV00077	LSDV00098	LSDV00120
LSDV00009	LSDV00035	LSDV00057	LSDV00078	LSDV00099	LSDV00121
LSDV00010	LSDV00036	LSDV00058	LSDV00079	LSDV00100	LSDV00122
LSDV00011	LSDV00037	LSDV00059	LSDV00080	LSDV00101	LSDV00123
LSDV00012	LSDV00038	LSDV00060	LSDV00081	LSDV00102	LSDV00124
LSDV00013	LSDV00039	LSDV00061	LSDV00082	LSDV00103	LSDV00125
LSDV00014	LSDV00040	LSDV00062	LSDV00083	LSDV00104	LSDV00126
LSDV00015	LSDV00041	LSDV00063	LSDV00084	LSDV00105	LSDV00127
LSDV00016	LSDV00042	LSDV00064	LSDV00085	LSDV00106	LSDV00128
LSDV00019	LSDV00043	LSDV00065	LSDV00086	LSDV00107	LSDV00130
LSDV00020	LSDV00044	LSDV00066	LSDV00087	LSDV00108	LSDV00131
LSDV00021	LSDV00045	LSDV00067	LSDV00088	LSDV00109	LSDV00132
LSDV00023	LSDV00047	LSDV00068	LSDV00089	LSDV00110	LSDV00133
LSDV00024	LSDV00048	LSDV00069	LSDV00090	LSDV00111	LSDV00134
LSDV00027	LSDV00049	LSDV00070	LSDV00091	LSDV00112	LSDV00135
LSDV00028	LSDV00050	LSDV00071	LSDV00092	LSDV00114	LSDV00136
LSDV00029	LSDV00051	LSDV00072	LSDV00093	LSDV00115	LSDV00137

LSDV00030	LSDV00052	LSDV00073	LSDV00094	LSDV00116	LSDV00138
LSDV00031	LSDV00053	LSDV00074	LSDV00095	LSDV00117	LSDV00139
LSDV00032	LSDV00054	LSDV00075	LSDV00096	LSDV00118	LSDV00140
LSDV00033	LSDV00055	LSDV00076	LSDV00097	LSDV00119	LSDV00142

Supplementary Table 6 117 core genes of CAPV genome

LSDV00013	LSDV00038	LSDV00059	LSDV00079	LSDV00099	LSDV00120
LSDV00014	LSDV00039	LSDV00060	LSDV00080	LSDV00100	LSDV00121
LSDV00015	LSDV00040	LSDV00061	LSDV00081	LSDV00101	LSDV00122
LSDV00016	LSDV00041	LSDV00062	LSDV00082	LSDV00102	LSDV00123
LSDV00019	LSDV00042	LSDV00063	LSDV00083	LSDV00103	LSDV00124
LSDV00020	LSDV00043	LSDV00064	LSDV00084	LSDV00104	LSDV00125
LSDV00021	LSDV00044	LSDV00065	LSDV00085	LSDV00105	LSDV00126
LSDV00023	LSDV00045	LSDV00066	LSDV00086	LSDV00106	LSDV00128
LSDV00024	LSDV00047	LSDV00067	LSDV00087	LSDV00107	LSDV00130
LSDV00027	LSDV00048	LSDV00068	LSDV00088	LSDV00108	LSDV00132
LSDV00028	LSDV00049	LSDV00069	LSDV00089	LSDV00109	LSDV00134
LSDV00029	LSDV00050	LSDV00070	LSDV00090	LSDV00110	LSDV00136
LSDV00030	LSDV00051	LSDV00071	LSDV00091	LSDV00111	LSDV00137
LSDV00031	LSDV00052	LSDV00072	LSDV00092	LSDV00112	LSDV00138
LSDV00032	LSDV00053	LSDV00073	LSDV00093	LSDV00114	LSDV00139
LSDV00033	LSDV00054	LSDV00074	LSDV00094	LSDV00115	LSDV00140
LSDV00034	LSDV00055	LSDV00075	LSDV00095	LSDV00116	LSDV00142
LSDV00035	LSDV00056	LSDV00076	LSDV00096	LSDV00117	
LSDV00036	LSDV00057	LSDV00077	LSDV00097	LSDV00118	
LSDV00037	LSDV00058	LSDV00078	LSDV00098	LSDV00119	

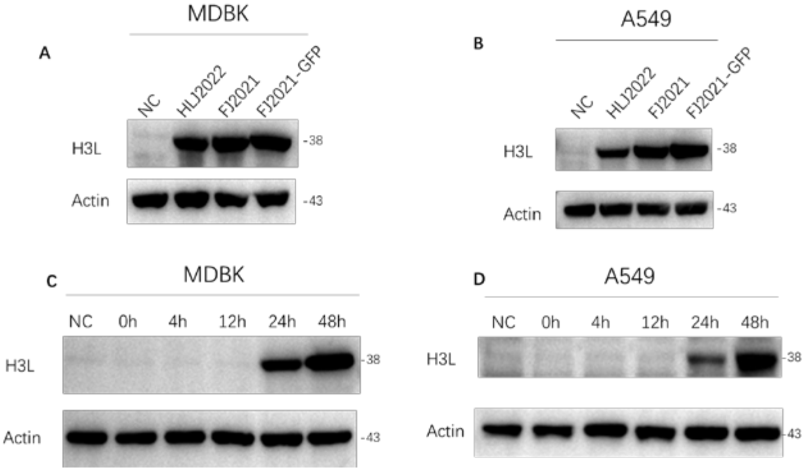

Supplementary Figure 7 The expression of the LSDV074(H3L) in MDBK and A549 cells. Replication status of different LSDV strains after 48 hours of infection at 0.1 MOI in MDBK(A) and A549(B) cells. The detection of H3 proteins at different time points post-infection in MDBK (C) and A549(D) cells.

Supplementary Table 7 The laboratory animal used for LSDV study

Strain/Vector	Antigen	Animal	Inoculation Symptoms	LSDV Detected	Challenge	Reference
LSDV Volgograd		Mice	NO	NO		[45]
		Guinea Pigs	NO	Yes		
		Rabbit	Inoculation site skin nodules	Yes		
		Syrian hamsters	Inoculation site skin nodules	Yes		
LSDV Neethling	SARS-CoV-2 S and N	Mice	NO	NA	SARS-CoV-2	[46]
		Syrian Hamsters	NO	NA		
LSDV Neethling	T. parva p67 and BLV gag	Mice	NO	NA		[47]
LSDV Neethling	HIV-1 gp150 and Gag	Rabbit	NO	NA		[48]
LSDV Neethling	Grttn	Monkey	NO	NA		[49]
LSDV KS-1	RVFV NSm and Gn	Mice	NO	NA	RVFV	[50]
LSDV Neethling	Grttn	Immunodeficient mice	NO	NA		[51]
LSDV Neethling	RVFV Gn and Gc	Mice	NO	NA	RVFV	[52]
LSDV Neethling	glycoprotein (RG)	Rabbit	NO	NA	Rabies virus	[53]
		Mice	NO	NA		

T. parva: Theileria parva; BLV: bovine leukemia virus; Grttn: HIV-1 subtype C Gag, reverse transcriptase, Tat and Nef as a polyprotein; RVFV: Rift Valley fever virus;

Supplementary Table 8 Cell lines tested for LSDV

Cell lines	Permissive	Reference	Cell lines	Permissive	Reference
sheep embryonic hearts (EHs) cell	Yes	[54]	Vero	Yes	[55]
primary bovine embryonic fibroblast (BEF) cell	Yes	[56]	293 T	No	[55]
primary lamb heart cells	Yes	[57]	MDBK	Yes	[58]
primary lamb skin cells	Yes	[57]	BHK-21	Yes	[55]
primary lamb testis cells	Yes	[57]	CHO	No	[55]
primary lamb kidney cells	Yes	[57]	PK15	No	[55]
primary goat ovarian cell	Yes	[59]	Hep2	No	[55]
TSTSCs	Yes	[60]	Hela	No	[55]
hTERT-CSF	Yes	[55]	MCF-7	No	[55]
hTERT-ST	Yes	[55]	A549	No	[55]
egg chorioallantoic membranes (CAMs)	Yes	[61]	OA3.Ts	Yes	[57]
Foetal bovine muscle (FBM) cells	Yes	[62]	ESH-L	Yes	[57]

1 Zeng, X. *et al.* Complete genome sequence analysis of goatpox virus isolated from China shows high variation. *Veterinary microbiology* **173**, 38-49, doi:10.1016/j.vetmic.2014.07.013 (2014).

2 Wolff, J. *et al.* Experimental Infection and Genetic Characterization of Two Different Capripox Virus Isolates in Small Ruminants. *Viruses* **12**, doi:10.3390/v12101098 (2020).

3 Mathijs, E. *et al.* Complete Genome Sequence of the Goatpox Virus Strain Gorgan Obtained Directly from a Commercial Live Attenuated Vaccine. *Genome announcements* **4**, doi:10.1128/genomeA.01113-16 (2016).

4 Biswas, S. *et al.* Extended sequencing of vaccine and wild-type capripoxvirus isolates provides insights into genes modulating virulence and host range. *Transbound Emerg Dis* **67**, 80-97, doi:10.1111/tbed.13322 (2020).

5 Tulman, E. R. *et al.* The genomes of sheeppox and goatpox viruses. *J Virol* **76**, 6054-6061, doi:10.1128/jvi.76.12.6054-6061.2002 (2002).

6 Wolff, J. *et al.* Establishment of a Challenge Model for Sheeppox Virus Infection. *Microorganisms* **8**, doi:10.3390/microorganisms8122001 (2020).

7 Krotova, A. *et al.* Genetic characterization of sheep pox virus strains from outbreaks in Central Russia in 2018-2019. *Transbound Emerg Dis* **69**, e3430-e3435, doi:10.1111/tbed.14727 (2022).

8 Tulman, E. R. *et al.* Genome of lumpy skin disease virus. *J Virol* **75**, 7122-7130, doi:10.1128/jvi.75.15.7122-7130.2001 (2001).

9 Kara, P. D. *et al.* Comparative sequence analysis of the South African vaccine strain and two virulent field isolates of Lumpy skin disease virus. *Archives of virology* **148**, 1335-1356, doi:10.1007/s00705-003-0102-0 (2003).

10 Vandenbussche, F. *et al.* Complete Genome Sequence of Capripoxvirus Strain KSGP 0240 from a Commercial Live Attenuated Vaccine. *Genome announcements* **4**, doi:10.1128/genomeA.01114-16 (2016).

11 Mathijs, E. *et al.* Complete Genome Sequences of the Neethling-Like Lumpy Skin Disease Virus Strains Obtained Directly from Three Commercial Live Attenuated Vaccines. *Genome announcements* **4**, doi:10.1128/genomeA.01255-16 (2016).

12 Toplak, I. *et al.* Complete Genome Sequence of Lumpy Skin Disease Virus Isolate SERBIA/Bujanovac/2016, Detected during an Outbreak in the Balkan Area. *Genome announcements* **5**, doi:10.1128/genomeA.00882-17 (2017).

13 Agianniotaki, E. I. *et al.* Complete Genome Sequence of the Lumpy Skin Disease Virus Isolated from the First Reported Case in Greece in 2015. *Genome announcements* **5**, doi:10.1128/genomeA.00550-17 (2017).

14 Lojkić, I., Šimić, I., Krešić, N. & Bedeković, T. Complete Genome Sequence of a Lumpy Skin Disease Virus Strain Isolated from the Skin of a Vaccinated Animal. *Genome announcements* **6**, doi:10.1128/genomeA.00482-18 (2018).

15 Sprygin, A. *et al.* Analysis and insights into recombination signals in lumpy skin disease virus recovered in the field. *PLoS One* **13**, e0207480, doi:10.1371/journal.pone.0207480 (2018).

16 Sprygin, A. *et al.* Complete Genome Sequence of the Lumpy Skin Disease Virus Recovered from the First Outbreak in the Northern Caucasus Region of Russia in 2015. *Microbiology resource announcements* **8**, doi:10.1128/mra.01733-18 (2019).

17 Douglass, N., Van Der Walt, A., Omar, R., Munyanduki, H. & Williamson, A. L. The complete genome sequence of the lumpy skin disease virus vaccine Herbivac LS reveals a mutation in the superoxide dismutase gene homolog. *Archives of virology* **164**, 3107-3109, doi:10.1007/s00705-019-04405-8 (2019).
18 van Schalkwyk, A. *et al.* Potential link of single nucleotide polymorphisms to virulence of vaccine-associated field strains of lumpy skin disease virus in South Africa. *Transbound Emerg Dis* **67**, 2946-2960, doi:10.1111/tbed.13670 (2020).
19 Mathijs, E. *et al.* Complete Coding Sequence of a Lumpy Skin Disease Virus Strain Isolated during the 2016 Outbreak in Kazakhstan. *Microbiology resource announcements* **9**, e01399-01319 (2020).
20 Di Felice, E. *et al.* Complete Coding Sequences of Lumpy Skin Disease Virus Strains Isolated from Cutaneous Lesions in Namibian Cattle during 2016 Outbreaks. *Microbiology resource announcements* **9**, e00124-00120, doi:10.1128/MRA.00124-20 (2020).
21 Orynbayev, M. B. *et al.* Genomic Sequence of the New Attenuated Vaccine Strain Neethling-RIBSP of the Lumpy Skin Disease Virus. *Microbiology resource announcements* **9**, doi:10.1128/mra.00318-20 (2020).
22 Sprygin, A. *et al.* Full-length genome characterization of a novel recombinant vaccine-like lumpy skin disease virus strain detected during the climatic winter in Russia, 2019. *Archives of virology* **165**, 2675-2677, doi:10.1007/s00705-020-04756-7 (2020).
23 Mathijs, E. *et al.* Complete Coding Sequence of a Lumpy Skin Disease Virus from an Outbreak in Bulgaria in 2016. *Microbiology resource announcements* **9**, e00977-00920, doi:10.1128/MRA.00977-20 (2020).
24 Ma, J. *et al.* Genomic characterization of lumpy skin disease virus in southern China. *Transbound Emerg Dis* **69**, 2788-2799, doi:10.1111/tbed.14432 (2022).
25 Mathijs, E., Haegeman, A., De Clercq, K., Van Borm, S. & Vandenbussche, F. A robust, cost-effective and widely applicable whole-genome sequencing protocol for capripoxviruses. *J Virol Methods* **301**, 114464, doi:10.1016/j.jviromet.2022.114464 (2022).
26 Bamouh, Z. *et al.* Draft Genome Sequence of the Capripoxvirus Vaccine Strain KSGP 0240, Reisolated from Cattle. *Microbiology resource announcements* **10**, e0044021, doi:10.1128/mra.00440-21 (2021).
27 Van Schalkwyk, A., Byadovskaya, O., Shumilova, I., Wallace, D. B. & Sprygin, A. Estimating evolutionary changes between highly passaged and original parental lumpy skin disease virus strains. *Transbound Emerg Dis* **69**, e486-e496, doi:10.1111/tbed.14326 (2022).
28 Flannery, J. *et al.* A novel strain of lumpy skin disease virus causes clinical disease in cattle in Hong Kong. *Transbound Emerg Dis* **69**, e336-e343, doi:10.1111/tbed.14304 (2022).
29 Kumar, N. *et al.* Evaluation of the safety, immunogenicity and efficacy of a new live-attenuated lumpy skin disease vaccine in India. *Virulence* **14**, 2190647, doi:10.1080/21505594.2023.2190647 (2023).
30 Mathijs, E. *et al.* Coding-Complete Sequences of Recombinant Lumpy Skin Disease Viruses Collected in 2020 from Four Outbreaks in Northern Vietnam. *Microbiology resource announcements* **10**, e0089721, doi:10.1128/mra.00897-21 (2021).

31 Wolff, J. *et al.* Characterization of a Nigerian Lumpy Skin Disease Virus Isolate after Experimental Infection of Cattle. *Pathogens* **11**, doi:10.3390/pathogens11010016 (2021).
32 Krotova, A., Byadovskaya, O., Shumilova, I., van Schalkwyk, A. & Sprygin, A. An in-depth bioinformatic analysis of the novel recombinant lumpy skin disease virus strains: from unique patterns to established lineage. *BMC Genomics* **23**, 396, doi:10.1186/s12864-022-08639-w (2022).
33 Huang, C. W. *et al.* Complete Coding Sequence of Lumpy Skin Disease Virus Isolated from Kinmen Island, Taiwan, in 2020. *Microbiology resource announcements* **11**, e0120421, doi:10.1128/mra.01204-21 (2022).
34 Suwankitwat, N. *et al.* Rapid Spread and Genetic Characterisation of a Recently Emerged Recombinant Lumpy Skin Disease Virus in Thailand. *Veterinary sciences* **9**, doi:10.3390/vetsci9100542 (2022).
35 Shumilova, I. *et al.* Overwintering of recombinant lumpy skin disease virus in northern latitudes, Russia. *Transbound Emerg Dis* **69**, e3239-e3243, doi:10.1111/tbed.14521 (2022).
36 Li, L. *et al.* Genetic analysis of genome sequence characteristics of two lumpy skin disease viruses isolated from China. *BMC Vet Res* **18**, 426, doi:10.1186/s12917-022-03525-9 (2022).
37 Issabek, A. U. *et al.* Genome Sequence of Atyrau-5BJN(IL18), a Recombinant Lumpy Skin Disease Virus with Knockout of Virulence Genes. *Microbiology resource announcements* **11**, e0038022, doi:10.1128/mra.00380-22 (2022).
38 Paungpin, W. *et al.* Coding-Complete Genome Sequence of a Lumpy Skin Disease Virus Isolated during the 2021 Thailand Outbreak. *Microbiology resource announcements* **11**, e0037522, doi:10.1128/mra.00375-22 (2022).
39 Putty, K. *et al.* First complete genome sequence of lumpy skin disease virus directly from a clinical sample in South India. *Virus genes* **59**, 317-322, doi:10.1007/s11262-023-01967-3 (2023).
40 Zan, X. *et al.* Molecular characterization of a novel subgenotype of lumpy skin disease virus strain isolated in Inner Mongolia of China. *BMC Vet Res* **18**, 295, doi:10.1186/s12917-022-03383-5 (2022).
41 Kumar, A. *et al.* Genomic characterization of Lumpy Skin Disease virus (LSDV) from India: Circulation of Kenyan-like LSDV strains with unique kelch-like proteins. *Acta tropica* **241**, 106838, doi:10.1016/j.actatropica.2023.106838 (2023).
42 Parvin, R. *et al.* Clinical Epidemiology, Pathology, and Molecular Investigation of Lumpy Skin Disease Outbreaks in Bangladesh during 2020-2021 Indicate the Re-Emergence of an Old African Strain. *Viruses* **14**, doi:10.3390/v14112529 (2022).
43 Wei, Y. R. *et al.* Retrospective genomic analysis of the first Lumpy skin disease virus outbreak in China (2019). *Frontiers in veterinary science* **9**, 1073648, doi:10.3389/fvets.2022.1073648 (2022).
44 Wang, J. *et al.* Isolation, identification and phylogenetic analysis of lumpy skin disease virus strain of outbreak in Guangdong, China. *Transbound Emerg Dis* **69**, e2291-e2301, doi:10.1111/tbed.14570 (2022).
45 Pivova, E. Y., Vlasov, M. E., Sevskikh, T. A., Povolyaeva, O. S. & Zhivoderov, S. P. A Study of the Susceptibility of Laboratory Animals to the Lumpy Skin Disease Virus. *Life (Basel)*

13, doi:10.3390/life13071489 (2023).
46 de Moor, W. R. J. *et al.* LSDV-Vectored SARS-CoV-2 S and N Vaccine Protects against Severe Clinical Disease in Hamsters. *Viruses* **15**, doi:10.3390/v15071409 (2023).
47 Whittle, L. *et al.* Development of a dual vaccine against East Coast fever and lumpy skin disease. *Front Immunol* **14**, 1143034, doi:10.3389/fimmu.2023.1143034 (2023).
48 Chapman, R. *et al.* Assessment of an LSDV-Vectored Vaccine for Heterologous Prime-Boost Immunizations against HIV. *Vaccines (Basel)* **9**, doi:10.3390/vaccines9111281 (2021).
49 Burgers, W. A. *et al.* The novel capripoxvirus vector lumpy skin disease virus efficiently boosts modified vaccinia Ankara human immunodeficiency virus responses in rhesus macaques. *The Journal of general virology* **95**, 2267-2272, doi:10.1099/vir.0.067835-0 (2014).
50 Ayari-Fakhfakh, E. *et al.* MBT/Pas mouse: a relevant model for the evaluation of Rift Valley fever vaccines. *The Journal of general virology* **93**, 1456-1464, doi:10.1099/vir.0.042754-0 (2012).
51 Shen, Y. J. *et al.* A novel candidate HIV vaccine vector based on the replication deficient Capripoxvirus, Lumpy skin disease virus (LSDV). *Virology journal* **8**, 265, doi:10.1186/1743-422x-8-265 (2011).
52 Wallace, D. B. & Viljoen, G. J. Immune responses to recombinants of the South African vaccine strain of lumpy skin disease virus generated by using thymidine kinase gene insertion. *Vaccine* **23**, 3061-3067, doi:10.1016/j.vaccine.2004.10.006 (2005).
53 Aspden, K., Passmore, J. A., Tiedt, F. & Williamson, A. L. Evaluation of lumpy skin disease virus, a capripoxvirus, as a replication-deficient vaccine vector. *The Journal of general virology* **84**, 1985-1996, doi:10.1099/vir.0.19116-0 (2003).
54 Rhazi, H. *et al.* Poxvirus sensitivity of a novel diploid sheep embryonic heart cell line. *Archives of virology* **168**, 232, doi:10.1007/s00705-023-05855-x (2023).
55 Ma, C. *et al.* The comparative study revealed that the hTERT-CSF cell line was the most susceptible cell to the Lumpy skin disease virus infection among eleven cells. *Journal of Virological Methods* **317**, 114745, doi:10.1016/j.jviromet.2023.114745 (2023).
56 Tan, J. *et al.* Lumpy Skin Disease Virus Infection Activates Autophagy and Endoplasmic Reticulum Stress-Related Cell Apoptosis in Primary Bovine Embryonic Fibroblast Cells. *Microorganisms* **11**, doi:10.3390/microorganisms11081883 (2023).
57 Rhazi, H. *et al.* Comparative sensitivity study of primary cells, vero, OA3.Ts and ESH-L cell lines to lumpy skin disease, sheeppox, and goatpox viruses detection and growth. *J Virol Methods* **293**, 114164, doi:10.1016/j.jviromet.2021.114164 (2021).
58 Fay, P. C. *et al.* Madin-Darby bovine kidney (MDBK) cells are a suitable cell line for the propagation and study of the bovine poxvirus lumpy skin disease virus. *J Virol Methods* **285**, 113943, doi:10.1016/j.jviromet.2020.113943 (2020).
59 Kononova, S. *et al.* A lumpy skin disease virus which underwent a recombination event demonstrates more aggressive growth in primary cells and cattle than the classical field isolate. *Transbound Emerg Dis* **68**, 1377-1383, doi:10.1111/tbed.13798 (2021).
60 Du, G. *et al.* Generation and application of immortalized sertoli cell line from sheep testis. *J Virol Methods* **316**, 114727, doi:10.1016/j.jviromet.2023.114727 (2023).
61 Munyanduki, H., Omar, R., Douglass, N. & Williamson, A. L. Removal of bovine viral

diarrhea virus (BVDV) from lumpy skin disease virus (LSDV) vaccine stocks by passage on chorioallantoic membranes of fertilized hens' eggs. *J Virol Methods* **275**, 113752, doi:10.1016/j.jviromet.2019.113752 (2020).
62 Binepal, Y. S., Ongadi, F. A. & Chepkwony, J. C. Alternative cell lines for the propagation of lumpy skin disease virus. *The Onderstepoort journal of veterinary research* **68**, 151-153 (2001).

**Supplementary Table 9.** Functional annotation of 223 gene clusters in the CAPV pangenome										
gene clusters	refenerce gene	eggNOG annotation	VFDB prediction	Victors prediction	Pathofact prediction	Pathofact toxin prediction	Scoary Clade 1.1	Scoary Clade 2.1	Scoary Clade 2.2	Scoary Clade 1.2
group 30	GTPV 01 00004	N-acetylmuramoyl-L-alanine amidase activity								
group 56	GTPV 01 00010									
group 142	GTPV 01 00025									
group 33	GTPV 01 00147	aspartic-type endopeptidase activity			pathogenic					
group 172	GTPV 03 00007									
group 55	GTPV 03 00012									
group 166	GTPV 03 00026									
group 167	GTPV 03 00047									
group 174	GTPV 07 00002									
group 41	GTPV 07 00136	dsDNA Poxvirus								
group 175	GTPV 07 00155									
group 10	GTPV 08 00022									
group 176	GTPV 10 00003									
group 177	GTPV 10 00150									
group 80	IAFEDADN 00091	phosphatase activity		pathogenic			1			
group 47	IPFAONOG 00043	Poxviridae protein		pathogenic			1		1	
group 36	KLAIMAEJ 00149	Protein of unknown function (DUF2718)								1
group 194	KLAIMAEJ 00150	dsDNA Poxvirus		pathogenic						1
group 182	LEFNJLPD 00001				pathogenic					1
group 49	LEFNJLPD 00032	Chordopoxvirus A33R protein								1
group 126	LEFNJLPD 00085									1
group 183	LEFNJLPD 00155						1			1
group 193	LSDV 01 00001	dsDNA Poxvirus		pathogenic			1	1	1	1
group 35	LSDV 01 00002	Protein of unknown function (DUF2718)					1	1	1	1
group 189	LSDV 01 00003	Poxvirus T4 protein, C terminus					1	1	1	1
BCRF1	LSDV 01 00004	Interleukin 10			pathogenic		1			
group 29	LSDV 01 00005	N-acetylmuramoyl-L-alanine amidase activity					1			
group 143	LSDV 01 00006	Poxvirus C4/C10 protein					1			
group 65	LSDV 01 00007	cytokine binding		pathogenic			1			
group 66	LSDV 01 00008	dsDNA Poxvirus								
group 67	LSDV 01 00009									
group 68	LSDV 01 00010	7 transmembrane receptor (rhodopsin family)								
group 69	LSDV 01 00011	aspartic-type endopeptidase activity			pathogenic					
group 54	LSDV 01 00012	N-acetylmuramoyl-L-alanine amidase activity								
group 70	LSDV 01 00013	peptidase inhibitor activity								
group 72	LSDV 01 00014									
group 23	LSDV 01 00015			pathogenic						
group 73	LSDV 01 00016	Apoptosis regulator M11L like								
DUT	LSDV 01 00017	dUTPase		pathogenic	pathogenic		1			
group 16	LSDV 01 00018	Kelch motif		pathogenic	pathogenic	pathogenic	1	1	1	1
F4L	LSDV 01 00019	Ribonucleotide reductase, small chain								
group 39	LSDV 01 00020	Protein of unknown function (DUF2701)								
group 52	LSDV 01 00021			pathogenic						
group 57	LSDV 01 00022	Orthopoxvirus F8 protein					1		1	
group 116	LSDV 01 00023	Lipid membrane protein of large eukaryotic DNA viruses								
VPK2	LSDV 01 00024	Poxvirus serine/threonine protein kinase			pathogenic					
group 9	LSDV 01 00025	Poxvirus F11 protein					1	1		1
group 85	LSDV 01 00026	Poxvirus F12L protein		pathogenic			1	1		1
group 209	LSDV 01 00027	PLD-like domain				pathogenic				
group 24	LSDV 01 00028									
group 145	LSDV 01 00029	Poxvirus protein F15								
group 90	LSDV 01 00030	Poxvirus F16 protein								
group 117	LSDV 01 00031	DNA-binding 11 kDa phosphoprotein								
PAPL	LSDV 01 00032	Poxvirus poly(A) polymerase C-terminal domain								
group 119	LSDV 01 00033	Poxviridae protein								
E3L	LSDV 01 00034	Double-stranded RNA binding motif		pathogenic						
group 91	LSDV 01 00035	Poxvirus DNA dependent RNA polymerase 30kDa subunit								
E5R	LSDV 01 00036									
group 146	LSDV 01 00037	Pox virus E6 protein								
group 196	LSDV 01 00038	Poxvirus E8 protein								
POL	LSDV 01 00039	DNA polymerase family B			pathogenic					
group 92	LSDV 01 00040	E10-like protein conserved region								
group 120	LSDV 01 00041	Chordopoxvirus E11 protein								
group 46	LSDV 01 00042	Poxviridae protein		pathogenic						
group 200	LSDV 01 00043	Poxvirus protein I1								

group 121	LSDV 01 00044	Pfam:Pox_EPC_I2-L1								
I3L	LSDV 01 00045	Poxvirus I3 ssDNA-binding protein								
group 147	LSDV 01 00046	Poxvirus protein I5					1			
group 93	LSDV 01 00047	Poxvirus I6-like family								
group 192	LSDV 01 00048	Vaccinia virus I7 processing peptidase								
NPH2	LSDV 01 00049	N-methyltransferase activity		pathogenic	pathogenic					
group 211	LSDV 01 00050	Protein G1								
group 148	LSDV 01 00051	Chordopoxvirus G3 protein								
group 218	LSDV 01 00052	Chordopoxvirus protein G2								
group 188	LSDV 01 00053	Glutaredoxin-like domain (DUF836)								
G5R	LSDV 01 00054	Poxvirus G5 protein								
RPO7	LSDV 01 00055	Chordopoxvirus DNA-directed RNA polymerase 7 kDa polypeptide (RPO7)								
group 76	LSDV 01 00056	Pfam:Peptidase_C92								
group 123	LSDV 01 00057	Poxvirus G7-like			pathogenic					
VLTF1	LSDV 01 00058	Viral Trans-Activator Protein								
group 124	LSDV 01 00059	Pfam:Pox_G9-A16								
L1R	LSDV 01 00060	Lipid membrane protein of large eukaryotic DNA viruses								
group 78	LSDV 01 00061	Chordopoxvirus L2 protein								
group 223	LSDV 01 00062	Poxvirus L3/FP4 protein								
L4R	LSDV 01 00063	Poxvirus nucleic acid binding protein VP8/L4R								
L5R	LSDV 01 00064	viral entry into host cell via membrane fusion with the plasma membrane								
group 164	LSDV 01 00065	Poxvirus J1 protein								
TK	LSDV 01 00066	Thymidine kinase		pathogenic						
group 97	LSDV 01 00067	Poxvirus C7/F8A protein								
PAPS	LSDV 01 00068	Poly A polymerase regulatory subunit								
group 150	LSDV 01 00069	Pfam:Pox_G9-A16								
rpoC	LSDV 01 00070	RNA polymerase Rpb1, domain 3								
group 206	LSDV 01 00071	protein tyrosine/serine/threonine phosphatase activity		pathogenic	pathogenic	pathogenic				
group 199	LSDV 01 00072	Viral late protein H2								
H3L	LSDV 01 00073	Poxvirus P35 protein		pathogenic	pathogenic					
group 212	LSDV 01 00074	RNA polymerase-associated transcription specificity factor, Rap94								
H5R	LSDV 01 00075	Pox virus Ag35 surface protein								
TOP1	LSDV 01 00076	Eukaryotic DNA topoisomerase I, catalytic core								
H7R	LSDV 01 00077	Late protein H7								
group 204	LSDV 01 00078	mRNA capping enzyme N-terminal, ATPase and guanylyltransferase			pathogenic					
D4L	LSDV 01 00079	Pox virus D2 protein								
group 58	LSDV 01 00080	Chordopoxvirinae D3 protein								
UNG	LSDV 01 00081	Uracil DNA glycosylase superfamily								
group 202	LSDV 01 00082	D5 N terminal like								
VETFS	LSDV 01 00083	N-methyltransferase activity			pathogenic					
group 198	LSDV 01 00084	Poxvirus DNA-directed RNA polymerase, 18 kD subunit								
D9R	LSDV 01 00085	phosphatase activity		pathogenic	pathogenic					
group 79	LSDV 01 00086	phosphatase activity		pathogenic	pathogenic					
NPH1	LSDV 01 00087	N-methyltransferase activity								
group 215	LSDV 01 00088	Poxvirus mRNA capping enzyme, small subunit								
group 203	LSDV 01 00089	response to antibiotic								
VLTF2	LSDV 01 00090	Poxvirus trans-activator protein A1 C-terminal								
VLTF3	LSDV 01 00091	Poxvirus Late Transcription Factor VLTF3 like								
group 201	LSDV 01 00092	Poxvirus A3L Protein								
group 207	LSDV 01 00093	Poxvirus P4B major core protein								
group 130	LSDV 01 00094	Orthopoxvirus A5L protein-like			pathogenic					
group 115	LSDV 01 00095	Poxvirus DNA-directed RNA polymerase 19 kDa subunit								
A6L	LSDV 01 00096	Poxvirus A6 protein								
VETFL	LSDV 01 00097	Poxvirus early transcription factor (VETF), large subunit			pathogenic					
VITF3S	LSDV 01 00098	VITF-3 subunit protein								
group 154	LSDV 01 00099	A9 protein conserved region								
group 155	LSDV 01 00100	structural molecule activity								
group 156	LSDV 01 00101	Poxvirus A11 Protein			pathogenic					
group 60	LSDV 01 00102	Poxvirus A12 protein								
group 99	LSDV 01 00103	Chordopoxvirus A13L protein								
group 185	LSDV 01 00104	Poxvirus virion envelope protein A14								
group 131	LSDV 01 00105	Chordopoxvirus A15 protein								
group 157	LSDV 01 00106	Pfam:Pox_G9-A16								
group 100	LSDV 01 00107	Poxvirus P21 membrane protein			pathogenic					
group 132	LSDV 01 00108	N-methyltransferase activity								
group 133	LSDV 01 00109	Protein of unknown function (DUF678)								
group 101	LSDV 01 00110	Poxvirus A21 Protein								

group 158	LSDV 01 00111	Chordopoxvirus A20R protein								
group 114	LSDV 01 00112	four-way junction DNA binding								
VITF3L	LSDV 01 00113	Poxvirus intermediate transcription factor							1	
rpoB	LSDV 01 00114	ribonucleoside binding		pathogenic						
group 104	LSDV 01 00115	viral entry into host cell via membrane fusion with the plasma membrane								
group 208	LSDV 01 00116	Poxvirus A28 family								
group 137	LSDV 01 00117	Poxvirus DNA-directed RNA polymerase, 35 kD subunit								
group 159	LSDV 01 00118	Chordopoxvirus A30L protein								
group 134	LSDV 01 00119									
group 210	LSDV 01 00120	adenyl ribonucleotide binding			pathogenic					
group 48	LSDV 01 00121	Chordopoxvirus A33R protein								
group 81	LSDV 01 00122	carbohydrate binding		pathogenic						
group 105	LSDV 01 00123	Chordopoxvirus A35R protein		pathogenic						
group 160	LSDV 01 00124									
group 50	LSDV 01 00125									
group 82	LSDV 01 00126	Protein of unknown function (DUF1235)								
group 64	LSDV 01 00127	CD47 immunoglobulin-like domain		pathogenic						
group 83	LSDV 01 00128									
group 51	LSDV 01 00129									1
group 84	LSDV 01 00130	Copper/zinc superoxide dismutase (SODC)	pathogenic	pathogenic	pathogenic					
group 106	LSDV 01 00131									
LIG	LSDV 01 00132	DNA ligase (ATP) activity		pathogenic						
group 2	LSDV 01 00133	Poxvirus B22R protein C-terminal			pathogenic		1	1	1	1
group 135	LSDV 01 00134	N-acetylmuramoyl-L-alanine amidase activity								
group 40	LSDV 01 00135	dsDNA Poxvirus								
group 136	LSDV 01 00136	Poxvirus A51 protein								
group 25	LSDV 01 00137	N-acetylmuramoyl-L-alanine amidase activity								
VPK1	LSDV 01 00138	protein serine/threonine kinase activity	pathogenic	pathogenic	pathogenic					
group 107	LSDV 01 00139	acid-amino acid ligase activity			pathogenic					
PS/HR	LSDV 01 00140	Sushi domain (SCR repeat)		pathogenic						
group 108	LSDV 01 00141	dsDNA Poxvirus					1			
group 109	LSDV 01 00142	protein serine/threonine kinase activity			pathogenic					
KBTB1 1	LSDV 01 00143	Kelch motif		pathogenic	pathogenic	pathogenic	1		1	
group 12	LSDV 01 00144	aspartic-type endopeptidase activity	pathogenic		pathogenic			1		1
group 42	LSDV 01 00145	PLD-like domain			pathogenic	pathogenic	1			1
group 110	LSDV 01 00146	aspartic-type endopeptidase activity			pathogenic					1
group 32	LSDV 01 00147	aspartic-type endopeptidase activity			pathogenic		1		1	1
SERP2	LSDV 01 00148	Serpin (serine protease inhibitor)		pathogenic			1			1
group 111	LSDV 01 00149	dsDNA Poxvirus					1			1
KBTB1 2	LSDV 01 00150	Kelch motif		pathogenic	pathogenic	pathogenic	1			1
group 62	LSDV 01 00151	aspartic-type endopeptidase activity	pathogenic		pathogenic		1			1
group 162	LSDV 01 00152	Pfam:DUF5409					1		1	1
group 190	LSDV 01 00153	Poxvirus T4 protein, C terminus					1	1	1	1
group 37	LSDV 01 00154	Protein of unknown function (DUF2718)					1	1	1	1
group 195	LSDV 01 00155	dsDNA Poxvirus		pathogenic			1	1	1	1
group 15	LSDV 02 00018	Kelch motif		pathogenic		pathogenic	1	1	1	1
group 18	LSDV 02 00019	Kelch motif		pathogenic	pathogenic		1	1		1
group 11	LSDV 02 00027	Poxvirus F11 protein								
group 86	LSDV 02 00028	Poxvirus F12L protein		pathogenic			1	1		1
group 112	LSDV 03 00001	dsDNA Poxvirus		pathogenic			1	1		
group 8	LSDV 03 00135	Poxvirus B22R protein C-terminal			pathogenic					
group 20	LSDV 03 00145	Kelch motif		pathogenic	pathogenic		1	1	1	1
group 14	LSDV 03 00147	aspartic-type endopeptidase activity	pathogenic		pathogenic		1	1		1
group 113	LSDV 03 00158	dsDNA Poxvirus		pathogenic			1	1	1	
group 163	LSDV 08 00004	Pfam:DUF5409					1	1	1	
group 59	LSDV 14 00082						1			
group 17	LSDV 28 00019						1			
group 89	LSDV 29 00001						1		1	1
group 88	LSDV 29 00159						1		1	
group 178	LSDV 31 00063	Rhabdovirus spike glycoprotein					1			
TK 2	LSDV 31 00064	Thymidine kinase		pathogenic			1			
group 180	LSDV 33 00027							1		
group 103	LSDV 38 00115	Poxvirus intermediate transcription factor							1	1
group 138	LSDV 38 00120	Poxvirus DNA-directed RNA polymerase, 35 kD subunit							1	
group 38	LSDV 38 00159	Protein of unknown function (DUF2718)							1	
group 45	LSDV 47 00116								1	1
group 77	LSDV 48 00058	Pfam:Peptidase_C92								1

group 181	LSDV 57 00065	Augments natural killer cell activity in spleen cells and stimulates interferon gamma production in T-helper type I cells					1			
group 3	LSDV 58 00133						1			
group 94	LSDV 63 00049	Poxvirus I6-like family							1	
group 184	PFHFNDGI 00100						1			
group 22	SPPV 01 00002	Poxvirus T4 protein, C terminus								
group 63	SPPV 01 00124	CD47 immunoglobulin-like domain		pathogenic						
group 31	SPPV 01 00142	aspartic-type endopeptidase activity			pathogenic					
group 26	SPPV 01 00143	Serpin (serine protease inhibitor)		pathogenic						
group 21	SPPV 01 00147	Poxvirus T4 protein, C terminus								
group 168	SPPV 02 00002									
group 170	SPPV 02 00127									
group 1	SPPV 02 00131	Poxvirus B22R protein C-terminal			pathogenic					
group 169	SPPV 02 00150									
group 171	SPPV 03 00024									
group 43	SPPV 03 00144	PLD-like domain			pathogenic	pathogenic				
group 27	SPPV 03 00148	Serpin (serine protease inhibitor)		pathogenic						
group 173	SPPV 04 00049	Protein of unknown function (DUF1029)		pathogenic						1
group 71	SPPV 06 00009	peptidase inhibitor activity								
group 5	SPPV 07 00119	Poxvirus B22R protein								
group 6	SPPV 07 00120									
group 7	SPPV 07 00123	Poxvirus B22R protein C-terminal								
group 13	SPPV 07 00134	aspartic-type endopeptidase activity			pathogenic					
group 4	SPPV 08 00131									
group 179	SPPV 16 00131									

>LSDV_01_00001 group_193
ATGTCTTCCGGCAACTATGTCTACCGAAACAACTTTTCAGATGATGATGATATCACAACC
GCCATCTCTGATTATTTGTTTTGGTCATCACTGGCATTTTCCTCCAGGGAGGTTGCTGGA
AAGGTGTTCTTAGTTTTTGAATCTTTCAAAAAGGATGCCTCACTTGTATTTGGAAATGAT
CTAACAGCTTTTGTCAAAAACATGTTTTTGGATTCTAAAATTGGATTTGAACAATCAAAA
ATTATGATTAATTCCATGTTAAAAAAAGAAAATTACATTAGGGAATCATGTGCAGTGATT
GGCATTTTAGCAAGAGCAGCAGAATATTGGGGTGGTGAATCATCTCCAACTTGTTCTTCT
GTGAAAGTGTTGGTATTGCTTCGAGACCTCGTTTCTGACAACGATATTTCGCTAGTGAAA
TCAGCACTAATAATTAGACTTAAAAGATTGAATGAAAAAAGTATCCATTTACAGGTTTAA
>LSDV_01_00002 group_35
ATGTTTGGAAACCTAAATCCGTTCGATTCTGTCTTGGATAAGGATGAAAAAATCCAAGAC
AATATAAACACTAATCACAAACCTATAGAAAGTAAAGAAATAAATAATGATCTACCATTA
ACTGTATTAGATAAAAAAGATACAAGCGATATAAATATTAGAAATGATAATGGAGTTTTT
GATTTTATTAAAATTCCTAATCCATTTAAAAAACACTACGAATACTACTGTAATCAAAAC
ACAATAAAGGAACCACCTAGAAAAGGATTAGTTGAAAGGATGATGAATATGGTTGAATAA
>LSDV_01_00003 group_189
ATGTCATCATTAACTTTGTTCATTTTCTTTTTGCAACTATTCATTTTTACTAGTAGTGTA
AGCGGAATATCAATAAAGAGATGCACAGAAGAAGAAAATAACACATGGGAAATCGAAGTA
GGATTATGTATCCAAACAGAAAATTTTAGAGCGATTAAAACTGGCTGTTATAAAATTCAA
GGACCTGGAGGACTTCTAACAGAGGGAAATGGATTTAAAATTTTCGCACATGATGATTGT
TCCAAAGAAGAAACACAAAACAACTTTATATTAGATAGTGTTAACGAAGCAGTTTATGCA
TTGGGTAAATATGTATATATGGAAATATCAACCAGTAATATAACAACTCTGTATTCGCTG
CCACAGTGTGCTAAGAGAATATCATTGTCAATTTCTTGTGATCAAGTAACTACAGAAATG
AAATCATACGTTGAGAGTGTAAGTTTTAAAGATTATGATTTAGAATTTGTTATAACAACA
GATATTAGTTGTGTAAAACATGTAAGTTCAAGTGTTATAGTTAGAAATGAATGTGAAAAA
AAATATATATCTACAGGAAAAAAGATTTTTGGGTTTAATAATAAAATAGATTGCTCAGCT
GTAAAATTTAGTGAACATGTAAATTATTTAAAAACGTGCAGTGTTGGGAAATTTGATAGA
AAAAAATATTATGAACATCAGCATAATTATATTAAAAAAATATTTCATCATAATGAACTA
TAG
>LSDV_01_00004 BCRF1
ATGAAAACAAACACAAAAATAATACTATTCTGTTATGTTATTCTTAGTTTGTATGTATTT
AGTTGTGCAATAGCATCGGCTAAAAAATGTGACGACGTTAGCTTTGATTACATATTAAAA
GATTTACGTTCAGAATTTAGCAAAATAAAAAGTTTTGTTCAAGACAATGATCAAGAAAAT
ATGATGCTATTAAGTCAATCAATGTTGGACAAGTTGACGAGTCGCATAGGATGTAAATCT
TTATCGGATATGATAAAATTTTATTTAAATGATGTTTTACCAAATGCAGAAAAAATAGAA
CACATGAAAAATAAAATAACTTCAATAGGAGAAAAATTAAAATCGTTAAAAGAAAAACTC
ATATCGTGTGATTTTTTACATTGTGAAAATCATGACGAAATAAAAACAGTTAAAACAATT
TTTAACAAATTAAAAGATAAAGGTATTTATAAGGCTATGGGAGAGTTTGATATTTTCATT
AATTACTTAGAAAAGTACATAGTTAAAAAGTAA
>LSDV_01_00005 group_29
ATGAAAGTTATAAATTTTATCATATTAATATGTTGTTTTAAAAGCGCATTTTTATCAGAA
TATTGTAAATATAGAGGGGGTATTCTAACTCCAGTGAATGTAAATTTCAAAGATCCTGCA
TTTTTAAAATGTCCATTGGATAATTATAAAAAATCTATTTTTTTTAAAAAAAATATAACA
TCATATGACATAACATGGTTTCAAACTAACTCTCTATCGTTTGTAAGAAATTATAACGAT
TACAAAAATTTTAAAAAAATAGAAGTAAATAATGAAAACGCGTCAAGGGTACAACTTATA
GGAAACAACTTATGGTTGTACCCTGTTATAAATATTGATGACGGAATTTATATTTGTAGA
TTTCGTTCACATAATATTTGTGAAGAAATGTCTATAAAATTAACTTTTTTGGATAAAGAA
GAAACAATTACTTACAGATTCAACGAAGGACTTAACTCAACAGTACATTGTATGGATATT
GACCTTATTAGTAGTTATTTTAAAAATAAAACACTAGATTGGTATAAAAATAATAACAAA
GTGTTATACGATGATAGGATACAGAGTAACGAACTTAAGTTGGTTATAAAAAACACAACA
CATGATGATAGTGGTATTTATACATGTAACTTAAGAATAAACAAAGATAGTATTAACTAT
GACATAAAAAGAAACTATAATGTTGTAGTTTTATAA
>LSDV_01_00006 group_143
ATGGAAACGATTAGTGATGGTATAATAAAAGTAAAAACCTTTAATGATGATTATTTTAAT
AACGTTAAAAAAATAATTATGGACATGATTAAATACAAAAACATAATATGGGAAGAGTCT
AAAGTATTTGATCATGAAAAAGGTATAGAAGTTATTAATACTAATGAAAGACAATGTAAA
CAGTATATAATAAGAGGTTTAGATGATATTTTTAAAGTTATAAGAAAAAAACTGTTATTA
TCTTTTGAATTTCCACAAAAAATTAGTGATATTATATTAGATAACACAATTACGCTTATA
AAGTACGAAAAAGGAGATTTTTTCAACACACACAGAGATTTTATACATTTTAAATCAAAA
AACTGTTATTGTTATCATTTAGTACTGTATTTAAACAATACGAGCAAAGGAGGTAATACA
AATATACATATAAAACCCAACACTATATTTTCTACTAAAAACGATGTCCTATTTGATAAA
ACATTAAATCACAGTTCTGATATTATAACTGATGGAGAAAAAAATATAGCATTAATAAAT
GTAGTTATAAAATATAATTCAAATAATGACGAAACCATATTTAAATTACCCTATTTATCG
GAAAATAATCATATAAACTTTTACGAAGATGAAGGAAATAGGAAGTTTTGTTACTGTCTT
GTTACCGTAAATAATTATTCTACAGAAGTAGATACATTTGGATTAATTTTAAATAGATCA
GGTAAATGTGTTTTGGTAAACAAGTATGATGATATAATAACGGAAAAATTAATATTTGAA
AATTTTGATGATATGTGTATGGAAATATCATTTAGTTATAATGATATTATTTTTGATTAC
TTAAAAAACGAAGATGGAAAAAATATAGCGTGGAAAGCCTTAAATTCGTCGTGTGATGAA
ATATGGTATCCAAAATCAAAAAACGATTTTGATTTTTTAAAAGACTTAGTTAAGTACGTA
AATAATAACTTAAAAGATAAAATAAATTATTATGTATTAACAGGGAACTGTAATTCTGAA
ATACATTATATAAATTTTAACGTAGTTCGATGCTATTTTTCTATTTAA
>LSDV_01_00007 group_65
ATGGGCGCGGCAGTATTTTTTGCTTTTTTGCTCTTTAAACTATGTTCTTCTTTTCCTAAA
GATATAAAACTAACATCTAATGATTTTAATTCTAATATTGGATGGATTAATGATGACAAT
AGTAGTAACTATAAAGTAATGATTATGACGTATAGTAGCGGAGAATGGAAACAAGCATGT
AATTATACTACTCAGCAATATTGTAACGTTTCGCCTTTTATAAATGATAATACTGATGAT
TTTTGGATAAAATTTATTTCAATTGACAATAAGGAATCAAATGTTTTTACGTTTAAACCA
ATTTGCGAAAGTGTTGTTATTACTACGCCATCTGTATTTATTAAACGGCAAGATGTTAAT
>LSDV_01_00074 group_212
ATGGAAACTAAAGAATCTGTTTTAATTGAGATTATACCAAAAATTAAAGAATACATTCTT
AATCCAAATATAAATCCAAAATCATATTCGGATTTTATATCAAATAATAAAAATATTTTT
ATTATTAATTTATATAATGTTTCTACAATCACTGAAGAAGATATTAGGTTGTTATATATG
ACAATAGAACAAAATTTAGAAGTTGACGATCAAACATTAATATCCATATTTTCATATATA
GGATACAAGTTTGAACAAAACGTTAAGGAAGAGATAACATCTAGTTTATTTATAAATGAG
TATAATACTACAACTGATGAGATGACGTTTAATTTATATAATTTATTTTTTGATAACCTA
GATGTATATCTTAGACAAAGAAGGATAAATGTATTAGTTAACGATGATCTTAATGGCGAC
ATAATGATAAATTATAAAACTAGTGATTTAACATCTAATTTTAACAAAGATATTGATCCA
GAAGTTAGAGAAATACCATTTAACATGAAAGATATGATAAGTTATGTATCTAAAAACATT
GACCAGTTCAGATTTTCTAAAAAATATTTAGATTTTGCATACTTATGCAGGCATATAGGA
ATACCTATTTCAAAAAAAAAACTTAATATGCGATATATATACTTATATAAAGTGGATGGA
GTTTCAATACCTATTGTTATAAAGGACTTTTTAGATGTTAAGTACGTTTATTTGGAAGAA
ACTAATAAAACGTATAAAAATTCCTTTTCTGAGGATAATAACAGTTTAATAGATTGGGGG
AACATTATAATACCAAAAATAAAAAATAAACATTTGTATAGTTATATATTTTTATCTAAT
TTTTACCTTCGAGACTTATTTCCAAAATTAATAGAAGAAAAAGAAATTACGTTTAAGGAT
TTAAAAAATATTAAAAAAATAGAAGTAATCGAACCAAAATCCTTAAGAGGTGAAGTAAAA
ATAGAGTTTGTACCATGTGAACATCAAGTTAAACTAATAGAAGCACTAAAAGTTGATACG
GAATATTTTACAAAAGTAAATAATTTTGCTACTGAGTACATTTATTACGAAAACGGTCTT
GCGTTTTGTAATATCTGTGGTATGAATGTACCAAACTTTAATTTAGATGCTGCAGATGTT
GTAAAGAATAATGTAATAGTTTCTACGTTTAATAAATCCATTTTTTTGAGCGAACCATAT
AGTTACTTTGTACATAGTCAACGATTTATTTTTAATATAATAATGTCTTTTGATAATATA
ATGAAATCACAGACATGGGTAATGAAATATAACATAAATAGGATTATATTAAATTTTTTA
ATTGAGATAAACTCTCGAAGACAAAAATATGAAAAAAAAATTTTCTAACGAAATTAAAAAA
GGTATTTTCTTCCTCAGATTATCCGCTAATTTATTTGATATTCATGTATCATCTACTGAA
TTATTTTATTCATCAAAAATATTAAACTTAAACTATATCGTCGTTTTAGTGATTATACTA
AACAGTAGTGCTGATTTTATTGTTTCGTACATGAATTCTAAGAAAAAAAAGGTTGAGGAG
TCATCTTTAAAATATGCAATTTCGGTTATAATATATGATTTTTTGTTAAAAACTAAAATT
TGTGAAAAAGGGTTTTTAGATACGATAATATTATTTACTGATGTTTATACATCCATCATG
CCAGAAGAATTAGATCTTCATCTTCAAAGAATAATAATCGAACTTAGAAAATTAGTTTCT
ATTGAAAGATCTTTAGTTGCGCCAAATTATGACGTTGAAGTTAAAACATTGGATTTGCCA
CTTGCAGCATTAAAATTTTTTGAAAGTTATACACTTATCGTGAACAATATGCCGGTTATC
GAAAACAAAAATAAAAAGCGTAATATTATTGCTCACCCTATTCCTATTTTAAACGATACG
AAGGAAATGTGGGAACAATTTAAAAAGATAACATCTGATGATGAATTAAAAGTTTTGATT
AGATACAACGACACAAACGCTACTAAATTAGTTATTTTTCCGACGCATTTAAAAATAGAA
ATAGAAAGGAAAAAAATGATAATCGCGTTAAAAAGCTTATTTATAAATAATGTTTTAAAA
TATTACTATTCTATCCCATCATTATACGTATTTCGATTCGGAGATCCGTTTCCTTTTGAT
GACGAACTTGTAGATAAATATCATGTACAGAGTAAAGTAAATTGTTACAACTTATTCCGT
TACCACTTGCTTCCAGAAAGTGATGTTTTTGTATATTTTAGCGATTCGCTTAATAGAGAT
AAATTAGAGTATACATTTTATAGATTTTTATCAAGATACGTAAATGATGTTTCTATTTGG
ATAGATGAAAACATAACTAAAATTAGAGAGCTATACTTCTTTAATTTTAATAACTAA
>LSDV_01_00075 H5R
ATGTCGTGGTCTATAAATTTGGGAAATAACGGAGATAATTTTAAAACATTAGAAGAAATA
CGAGCACATGTAAGGTCGACAACTGAAAATATGGATAAGGTAGCGGACGAAATCTTTCCG
AGTGATGTAAAAATTCCAACGACTTCCTCTTCTTCAAAGCAAAAGAAACCAGTTGTTCGT
AAGAAAGCGGTTACGACAACAAAGAAAAGTAAAAAGATTAAAGAAAAAACCCCTATCGAA
GATCACGATGACGAAAACGATAATGATAATGATGATGAAAACAATGATAATAATGATAAT
GATGATATTAATAATATTGAAGCAGATGATAAAAATAATGATACAAATAATAGTGACACT
GAAGAAAATGAAAATGACGATAAAAAAGAAGACAATAAAAAGAGCAAAAAAAGCACCAAC
GACAATAATCAACACGACGATGACGTGATCGATAATTCTGATTTAAAAATTGCAACTGAG
TCAATAATTAAAGGTTTGAAAATACTTAACAATAAGGTATCGTCCGTTTCAACTGTATTG
GAAGATGTACAAGCTGCGTCAATAAATAGGCAATATAGTTCTTTAATTAAGAACATAGAT
TTGCTTAAATCGCTTGCGGAATGTGGCAAATCTCAAGTTATTAGGAAAAAAGTTAAAAAC
ACTAAAAAGTAA
>LSDV_01_00076 TOP1
ATGAGAGGATTATTTTATAACGATGGAAAACTTTTTACGGATAAAAATTTAACAACTCTT
GTATCATTTGATAATCCTACTTATGAAATTATAAAAAGGATTAGGATACCGCCCCACTTA
ACTGATGTTATTGTTTATGAACAAACGTATGAACAGTCATTAACTAGATTGATTTTTGTT
GGTTCAGATTCAAAGGGTAGAAGGCAGTATTTTTATGGAAAACTGCACGTAAAAAATAGA
AACTCCAATAGAAACAAAATATTCATAAAGGTGTTTAATGTTATTAATAAAATAAATGAG
TTTATAGACAATAATATTATTAATGAAAAAGATAAAAATGATGTTTTGTTCCAATCAGGA
GTGTTTATGCTAATGGAAACAAGTTTTTTTATCAGGATGGGAAAGATGTGCTATCTAAAA
GAAAATGAAACGGTAGGATTATTAACTTTAAAAAATAAACATATAAGCATAAGTAATGGA
AAAATAGTTATAAAATTTATAGGAAAAGATAAGGTTCCGCATACCTTTATAGTACATAAA
TCTAATAAATTATATAAACCTCTTTTAAAATTATTTGATAAAGAATTTCCTGATAACTTT
TTATTTTCTAAGTTGAGCGAACAAAAAATTTATAAACTAATGAAAAAATTTAAAATACGA
TTAAAAGATTTACGTACATATGGTGTAAATTACACGTTTTTATATATAATTTTTGGTCTAAT
GTAAAATCGTTAAATCCTATACCATCTACAAAAAAGTTGATATCTACATCTATAAAACAA
ACGGCTGAAATTGTTGGTCATAGTCCTTCAATTTCTAAAAGTGCATATATCGCAACAACT
GTTATAGATTTTTTAATTCAAGATTCTAATATATTAGAAACAATAAAAGAAAAATCGTTT
GAAGAGTTTATAGAATTAATTGTAGATTATATAGTAAATAGAGACACTATATAA
>LSDV_01_00077 H7R
ATGGATGAAAGATTAAAGACGATTTCTATGACCTTTTTTTATGGTGAATTAACAACTACA
GATATAATGGTTTTAAAGGCACACTTGTTAAACATTTTTCCAAATGATACTATTTTTTCT
ATAAATGAGGATAATAAATTTATAATTGACTTTAAATATAACGACTGTTTAGCTTCAGGT
TATATTAACCAAAAAATAATACCTATAACTCCAGAAAAATACAATGAATATTCATCGATG
ATAGCTAAAGAGTTGACGAACTATAATATTATCATTGATGATATTAAAGGGCATATACTA
TCATCTAAAAAACTTAAAAGATTTATTAAATTTTATAAAACAAATAATAATAAACAAAAT
>LSDV_01_00138 VPK1
ATGCCAAAACGTAACATAAACGTTTTTGAGGAAGGTGATGTGTTAGTAGATTCTGTAAAG
AAAGAATGGCGATTAGGGAAAATAATTGGTCAAGGTGGGTTCGGTTTCATATTTTTAGCA
TATTCACAAAATAATGAAGAATATGTGGTAAAAATTGAACCTAAGAGCAATGGACCATTA
TTTGTAGAACAAGTGTTTTATCAACGGATAGGAAAACGAGACATGATAACGTTATGGTCA
AAAAATAATCACATAGATCATTTAGGAATTCCAGTATTTTATGGTTTTGGATTTCATAAA
AAAAATGGAATAGATTATAGGTTTATAATTATTAATAGATTAGGTTGTGATTTAAATAAA
ATAATACAGTGTAATAACAATAAACTTCCTGAAAGATCTGTGTTTTTAATAGCGTCTAAA
ATAATAATGATATTAAAATACCTACATGAAAATGGCTATACGCATAGTGATATTAAAGCA
TCTAATATAGCAATTGATATCAATAATAAAAATAAAATTTATTTATTGGATTATGGATTA
TCTTATAGATTCATGATAAACGGCAACCATGTGGAGTATAAGCGAGATCCCAAAAAGATG
CATAATGGAACAATAGAATACACAAGTATAGATATGCATAAAGGTGTATCCCCGTCTAGG
CGAGGAGATTTGGAAATTTTAGGATATTGTATAATAAAATGGTTAGGTGGTAAATTGCCA
TGGGAAAATGATTTAAAAAATTGTAAATATGTAATGGAGTCAAAGATTAAATACATGAAC
GATATTGGAAATTTAATGACTGACTCGCTAGGATCTAATTATCCTGAAAAAATTTTAAAG
TATTTTAATTATATAAAAACATTACAATACGATTCTATTCCAGATTATGAAAAAATAATG
TCATTTTTTCTTCTTTAA
>LSDV_01_00139 group_107
ATGGATTCTGATAGTCGCAACGGTTTTATTTTGCTAAATTATGAAAAAGTCAAAGTTATT
ATTGTGTTAGCAAATAATTATGTTAACATAACAAAACTTTGTAAACCTATGGGTAAATCA
TTCCACAATTGGTGGAATTTAAAAAGTAGTTATCAAATTATATGCAACATAGCAAAAGAA
GAAGATATAGAAGTTGAAAATCTTTCATTTAATGTTTGGAAAGGACAAGAAACTAAAGAG
GTTTATGGAAAATATATTCATCCTAAGTTGTTGGTATATATTTTAAACTGGATTTCAGAA
GAGTATTGTGCCAAAATTCTATCAATAATTAATGAATTTAATTCAAAAATATTAAATAAT
ATTACAACCAATACCAAAATTAACAAAATTTATACGCGTCTACCGAAAGAAGAGTCTATG
TTTAATGCAATATTTAATAAAGAAAATAGCAAATCTAAAAATTACAAAAAATTTCTAATA
AACATTCCAACGATATTATCAAAATACGAAAAATATTTTTTGGAATCTGAAGAAAAAGAG
TGCGCAGTTTGTTTAGAAAAAGTATATGATAAGGAATATGATAGCATGTACTTTGGAATA
TTACCTAATTGTGATCATGTCTTTTGTATAGAATGTATTAATATTTGGAAAAAGGAAAAC
AGTACATGTCCAGTATGTAGGAATGAATTCCTATTTGTTATAAAAAGTAGATTTTTTCCA
TAA
>LSDV_01_00140 PS/HR
ATGAAAAATATACATATGTTATTGATTTTATTATGTAATAAAGTATATTCGTTATGTGAT
TTAAATAAATGTTGTTATCCTCCATCGATAAAAAATGGATACATATATAATGAAAAAACT
GAATATAATATTGGATCAAATGTAACATTTTTTGTGGAAATAACACGCGAGGTGTTAGC
TATACTTTAGTAGGAGAAAAAAATATTATTTGTGAAAAAGATGGTAAATGGAATAAAGAA
TTCCCTGTTTGTAAAATTATAAGATGTCGATTCCCAGCTTTACAAAATGGATTTGTAAAT
GGAATACCTGATAGTAGAAAATTTTATTATGAATCTGAGGTAAGTTTTTCATGTAAACCG
GGTTTTGTTTTAATAGGAACAAAATATTCAGTTTGTGGTATAAATTCGTCATGGATACCT
AAAGTACCCATTTGTTCAAGAGACAATATTACATATAATAAAATTTATATCAATAAAGTA
AATATAGATGATAACTTTTTTAACCAAATAAATAACAGTAATACTTATTACTTTGATAAA
ATATTACAAATAAATAATGTTAACAGATACACTTTAATTTTTTTTGTTGTTGTATCAATT
AAAATATTATTTGGTTTTGTATTTATATTCTTTTCGTGTAATAAAAAACCTTTAGATAGT
ATAAAATATTATAAATAA
>LSDV_01_00141 group_108
ATGGGAAATAAAAAAAATGATGATATTAAACATATTATTAAAGAATATATTAGATGGAGA
GGAAACGGTGAAGATATATGCGAAGAACGTGAACTAAATATTTTTTTAAGAAACTCAAA
AAAATGGACTCGTTTGTAAAAAAAAATATTAAAGAAAGTAATAATGATGAATATGATATA
ATCAAATCTATGAAGTTGTCGTTGGATGATGGTCCAAGGATGGATAAGCTTCCAGAATAT
TTGTTGGATGGGTTGAATGCAGAAGAAATACTAGGTCTATGTGGTATAATATCTGAAAAG
GTTGTTAGTGCAACATATGAAGTAAATTGGGATCGTGTTTATGAATCGTTATTTAACATA
TTAGATGAAAAAGATTTAAACTACATTAAAAGTAAAATAATGTAA
>LSDV_01_00142 group_109
ATGGACGAAAGAAACTCATTTTGGAGTTTAGTAAAAAAAAATAACTACAGAAAATTCACAA
AATATAATAAACCAAAGCTATATTAATCCAATAAGATGTATAAAATCAGATGATATTTAT
CAATCTTATACAAGTGTCGTTATAAAAGAAAATGATCAAAATTACATTCATAAAGGTATT
TTTAATAATAAAGAAGTTATCGTTAGAACTTTCAAAAAATCGCACACAAGTCACAAAATA
CTAATGGATATTTCAAATAACGAAATAAAAAATTTAAGAAGGATAGACAGTAATAATATA
TTAAAAATTTACGCATTTTATATAGAAATGTGTGACGGTCTTCCAAGGCTTTCGTTGATA
TTAGAATATTGTAAAAGAGGATATTTAAAAAATGTAATAAGAAAAGAAAGAGATTTAACT
TTTAAAACAAAATTAGATATGGCAATAGATTGCAGTGTTGGTTTATATAACGTTTATAAA
TACACAAATAATAAACCATATGGATGTATTTCAAGCGTTAGTTTTTAGTAGAGGAAAAT
TATAAAGTAAAGATTATATGTCATGGTTTAGAAAAAATACTAGCCAATCCACCTTTTAAA
AATATAAACGCTATAGTTTATCAATCTCATAAAATGTTATCAAATGTGTTTAATGAACGC
ACTATGGATGATGATATGTATAGTTTTGGCGTTGTTTTGTGGGAAATATTTTCTGGAAAA
ATACCATTTAAAAATCTAACAACGAAAGAAATATATGATTTAATCATTAATAAAAATGGA
CAGTTAAAACTTCCTGAAACGCCGCATTTAATTAAATATACAGTAGAAAAATGTACTTCA
CATACCAGTAAGAGACCAAATATAAAAGAAATTTTGTACAATTTTTCATTGTATAAATTT
TACAACTAA
>LSDV_01_00143 KBTB1_1
ATGTATTCATTTTACGAATTACGTAAATACTGCGATGTATCAATATGTTTTAATGATGTG
ATTAAAATTAAAGGTCATAAAATAATTTTATCAAATTCATCTAAATACTTTGATACCATG
TTTAGTAATAAATATTTTATAGAAAACCAAAAAAATGACATAAGTATTAATATATCAAAT
TTAGATAATCCAGAGGTATCAATAAATGAGATAATTAAATTTATGTATAACGGGAAATTA
GATAAAACTCTTAAAGATTATGTAATCTTAAAAGATATGCTTAAAATAGCAGATTATCTT
ATTATAGATGATATTATCCCACTTTGTATAACATCTATAGTTAAAATTATAGATTTTAAT
AATTGTCTCGATGCGTGTATTTTTTCAGAATTTTATAACTTAAAAAAACTAAATAGGTTT
ACTTATAAATTTATACGAAAAAATATACGTAAAATATATTTAAAAAAAGATTTTATTTAT

ACTGTACTAACGATTAATCACCCACTAGCCAAGGATAATAAGAAAAATTCTCCTATATAT
AACAACGATAAGTATTGTAATATTGTGTTTAAATATATTTTAAGTATTACGTTTGGTGAT
TCAAATACAATTAAATATGAAGTTGATGAACAGTTTTGTGATAAAAAAGGATGTATTGTT
GAATTCGGGTCTCAGGAAAAAGTTTGTGTTACAGTTTTTGGAAATTACGAAACTGATTAT
TATCAAATAAGGACAAATGAAACAAGTAAAGAATGTATTAACGAATTGACGCCTAGGAAA
GTTGATACATTTCTTGTAAAACAAAAAACAGATTTAAGTTTGCTTAAACGGCGAATTCAG
AAAGTTACTAAAAATAAATTCAAAGGAGAAAAATCATTTAATAAAATTGTAGGAATTTAC
AATCTTGTGTTATCACTTTTTGAGTATATCGTCAATGAATTGAATTAA
>LSDV_01_00008 group_66
ATGTATACTAAAAATAAATTTAAAATCTTTACTAAGTATATAACCTTTCTTTTTTCTATC
AATGAAAAAAAGGTAAAATATTTTTTAAATAAAGTTTTTTATCTTTCAAATTATGATTAT
ACGAAGTATGACATAACGAAAATTGTATTAAAAAATTCAAATAAATTTACGTTTACTATG
ATAAAAGTTATATCAGAATATGTACTTAATGAAAATGATTATAAAAAAGTTATAAAAGAA
TTAAAAATTGATGGTGAATACTATGCTGATTTTTATCCATTTATAAAATCTAAAAGTTTT
AAGCATATCAATTCCATGAAAAAAGTAAATTTTAACGATATGATGGAAAATTATTTTATG
GTACGAAAAATGAAAAATACAAAAAGAATAGGTAAAATATACACTGAGTTTTTAACATAT
GATACTATGGCTGTTAATTATTACGGCGTTGACTATATTAATAAAATAATATCAAAATTG
CGTTTAAGTAAAACACATTACTGTTTTTCACTTTATGAAAAAAATGAAAATAATATAATT
GATATAACCACAATAAAATGTATGATAGGTTTAATATCAATAATTTCTTCACATGATAAG
GGTAAGTATCCAAGTTTTTACATATTTTTTGACGAAATAAGTCATTTTAAGAGAAAAGAA
TTTGAAAAATATATCTGTGATAGATTTCCATAA
>LSDV_01_00009 group_67
ATGGAAGGGAGTGATAATACCAACACTCATTGTTGGATTTGTAAAGATGAATATAATGTA
AGTACAAACTTTTGTAATTGCAAGAATGAATTTAAGATAGTTCATAAAAACTGTTTAGAA
GAATGGATTAATTTTTCACATGATACAAAATGTAAAATATGTAATGGAAAATACAATATT
AAAAAAAATAAAAAGAGTTGTTTAAGATGGAAATGTTCGTTTATGTATTGTAACATACCA
GCTATTTGCGTTTCTTTAATATGTTTACTACTTTTGCCACTAACCATACTTTTAGTTAAG
TTTAATTTGAAAAGTATGTTAGAAAATATTGAAAATCGTGATCTTATTACGCTAATATCC
GCGATTGCATATTCCCTTCCTTGTGTTGTTGGATTTATAACTGTAATACATATACTAATA
GCATTGTACGACTATTATCTTGCCGCGAAGAGTGATAATATAACATATCAGGTTTATGAA
TATATATAA
>LSDV_01_00010 group_68
ATGAATTATACTCTTAGTACAGTTAGTAGCGCAACCATGTATAATAGTAGCAGTAATATT
ACCACTATAGCTACTACAATTATTAGTACAATTCTCAGTACAATTTCAACAAATCAAAAT
AATGTTACAACGCCTTCAACTTATGAAAATACAACAACGATATCTAATTATACAACCGCA
TATAATACAACTTATTATAGCGATGATTATGATGATTATGAAGTGAGCATAGTCGATATC
CCACATTGTGATGATGGTGTGGATACTACAAGTTTTGGACTGATTACTTTATATTCGACT
ATATTCTTTCTTGGATTATTTGGAAATATAATTGTGTTAACTGTTCTTCGTAAATATAAG
ATAAAAACAATACAGGATATGTTTTTGCTTAATTTGACACTGTCTGATTTAATTTTCGTG
TTGGTGTTTCCTTTTAATTTATACGATAGTATCGCTAAACAATGGAGTTTAGGAGATTGT
TTGTGTAAATTTAAAGCTATGTTTTACTTTGTTGGTTTTTACAATAGCATGTCATTTATA
ACATTGATGAGTATTGATAGATACCTAGCTGTAGTTCACCCAGTAAAATCAATGCCGATA
AGGACAAAACGATATGGAATTGTACTTAGTATGGTGGTTTGGATTGTCTCAACTATTGAA
TCCTTTCCAATAATGTTATTTTATGAAACAAAAAAAGTATATGGAATAACGTATTGTCAT
GTATTTTATAACGATAATGCAAAAAATTTGGAAATTATTTATAAATTTTGAAATAAACATA
TTTGGAATGATTATACCGCTAACTATTTTGCTATATTGTTATTATAAAATCTTAAATACT
TTAAAAACATCACAAACAAAGAATAAGAAAGCCATAAAGATGGTGTTTTTGATTGTTATC
TGTTCAGTATTGTTTTTACTCCCATTTAGTGTAACTGTATTTGTTTCATCGTTGTATTTG
TTAAATGTTTTTAGTGGATGTATGGCGTTAAGATTTGTCAACCTTGCAGTTCATGTAGCT
GAAATTGTGTCTCTATGTCATTGTTTTATCAATCCACTAATTTATGCGTTTTGTAGTAGA
GAATTTACTAAAAAGCTTTTACGATTGCGTAGCACCAGTAGTGCTGGTAGTATTAGCATT
GGATAA
>LSDV_01_00011 group_69
ATGGAAAAGGAAAAATTATGTAGCGATTATGATAATGACTTTACAGATTACTTATTTTAT
AGATATTGTAATCCATTATTTTATTACGTTGAACAAGATGATATAAATGGAGTAAAGAAA
TGGATAAAATTTGTTAATGATTGTAACGACTTATATGAAACGCCATTGTTTTCGTGTGTT
GAAAAAGATAAAGTTAATGTTGAAATATTAAATTTTCTCATAGAAAATGGATCCGATATA
AATTTTAAAACTAGAGATAATAATTTATCAGCACTAGCTCATTATCTTTCTTTTAACAAG
AATGTCGAACCTGAAATAGTTAAAATATTAATTGATTCTGGATCATCTATAACAGAAGAA
GATGAAAATGGGAAAAATTTGTTACACATGTACATGTGTAATTTTAATGTTCGTATAAAT
GTAATAAAGTTACTAATAGATAGTGGAGTTAATTTTTTGCAAAAAGATTTTGATAATAAT
AATATATTGTATAGTTATATTTTGTTTCATTCTGATAAAAAGATATTTGATTATCTAACT
AGTCTTGGTATCGATATGGATGAAACTAATACGTCAGGTTACAATTGTAAGGATTTAATT
AAATTTAGAAATCTCACCTTTTTTGACAAACAATAA
>LSDV_01_00012 group_54
ATGGAAAAAGTAACGACATTTATTTTCTATTTACTGATTATCAATGTTAAAATTCTTGCT
TTTACAAAATTAAGCTTTGGTTGTCTACAATCCGTGATGAAAACGTTCGAAAATAACTCA
GAAATAGTAAAATGTCCATATGTTGATACTTTAAATTATAATACAACAGTAAAATGGTTT
TTAGTTAATTACAATAAATACGTTAAAACAGGAGATGTAAAAAAATCTTTTCATGAGTTC
AAAAATACCGATAGAGTATATTCTGTTGGGCATAGCCTGTGGATAAATAATATAACAAAA
AATGATTCTGGAATATATGCATGTAATGTACATAACTCAACATATTATTTTACTTCATCT
GTTATGTTAGATGTTTATAAAAAAGAGACTATAAACTCTGTCATTGGCACAAACTATGAG
TTGTTGTGTATAAATACAAAAAACTTTATAAATACGTTTAATTGTTCTTATGAAGTAAAT
TGGTTTAAAGACAGGGAAAAAATAAACAATAATTTTAAATATAAAAAAAGCAAAGAAAAA
TTACTAATACGAAATATTTCTAAGAGTGATGAAGGAGTATATTTATGTTCCATAACTACG
GATACACGGTGTAATCCAAAAAACTTGGCATTAAAACGTTATTATCATAATAACGTAATT
AAAAAAAGATTTAAAGATGATATAAATTTAACAGTAAATTTTTTAAATAGTACAATAGCT
TTAGGTAAAAACTTTTTAATGGAATGTATTGGAGAATCTAAAAACATACAACGTGTTTAT
AAAAAAATAATTAACGCAAGTAAAAAATTAAATATTGCGTTAAATAAAGGAATTGATTAT
GATTATATTAGACATATTAATTAA
>LSDV 01 00078 group 204
ATGGACGAAAGAGACTTAGAAAACTCATTAAGTGATTATATTAACAAAATAGTTGAAATA
TACGATGAAATTCCAAATCCTACATTATCTAATGATGTAAATCATGAAGTAGAATTAGTA
TTTATACAACCCCCAATAATTACATTAAGTAATGTATATAATATTTCATCGGTTACAGAA
TCATATATACTTTTTACTGTAACCAATAAAGGAGAATGTATAAAAATTAGAACTAAAGTG
CCCATGTCAAAAATACATGGGTTAGATTTAAAAAATTTACAATTAGTTGATTTAATCGAT
GATATAATTTGGGAAAAGAAAACACTAATAAAAGAAAAAAAAATTGATAAACATTGCATT
ATAAGATACTCTTCTGAAGAAAGACATGTATTTTTAGATTACAAAAGATATACTTCATCT
ATAAAACTAGAATTAGTTAATTTAGTACAAACTAAAGTAAAAAATATAGTGGTTGATTTC
AAAATTAAATATTTTTTAGGGTCTGGTGCACAAGCAAAAAGCTCATTGTTGCATGTCCTT
AACCATCCTAAATCTAAACCTACTCCTACTTTAGAATTTGAAATCATTACACGTGAAAAA
AAAAATTCTCGTGATATATTACTAGATGAATTACATATACTTTTTAAAAATATATTTATG
GCAGATCCAAAAAATGTTTTCCTCCCTACCGTTATTTTTACTAGTCCTGTAAAAACTATA
ATGTTAAAAAAACAAGAAATACATAATATAGAATTGGATAAGTTATATATTACAACAAAG
ACTGATGGGGTTGGTACTTTAATAAAAATTAATAAAAATGGTATATTTTGTTATTTTACT
CACCTAAACTACATAATTAAATATAACCTGTTACGTAAGTTTGACGATACTGTAATATTA
TACGGAGAGGCGATAAAACAAAATCAAAAATGGTTCATATACTTAATAAAGTTATTGAAA
CCAGAATTATCTAATAGATTTAAAGAACGAGATTTTTTGATAGAAAAGTTGACAAGTGAT
ATATCGGATAGAATTGTGTTCAAAACAAAAAAATATGAAGGACCGTATGAAACACATTCT
GAAGTTATAGATTTATTATCTACATATCTTCCATTACAAACCGAAGGCGTGATTTTGTTT
TATTCAGAAGGTAATAAATCAAAAATAGATTATAAAATTAAACATGATAATACTACCGAT
CATATGATAAATGCCGTATATAGATATATGTCAAGTGAACCCGTTATTTTTGGAGATAAA
TATACGTTTATAGAATTTAAAAAGTTTAGCGACGAACGGGGATTTCCTAAAGAGCATGGA
ACGAGTAAAATAATACTAGGGGATAACATAAAATATTTAAACAATATATATTGTATCGAA
TTTAAAAATATTTATAACGATGTAGGATTAAAAAATATAGTTTTACCGATAAAATTTATT
TCAGAATTTTCATTTAATGGAGATTTTTTAAAACCTAGACTGGATAAAACTATGAAATAT
TTCTATAAGGAGTATTATGGAAATCAATACCAAGTAGTATTAGAACATATTAGAGATCAA
GAGTTAAGAATTAATGATATTTTTGACGAAAATAAATTATCTGATTTAGGAAAGAAGTAC
GTAGACGACGAATTTCGACTGAATCCAGAAGTATCTTATTTTACTAATAAACGAACTAGA
GGCCCGTTGGGAATTTTATCAAATTATATAAAAACTCTTTTAATATCTTTATATTGCTCT
AAGACCTTTTTGGATAATCCAAATAAAAGAAAAGTATTAGCCGTTGATTTTGGAAATGGT
GCAGATTTAGAAAAGTACTTTTATGGTGAAATAGCATTGCTAGTAGCAACTGACCCAGAT
AAAGACGCAATAAGTAGATGTATAGAAAGATATAACAAATTAAATTCGGGAATAAAATCA
AAATACTATAAACTGGACTACATACGAGAAACAATACGTTCAAGCGTCTATGTTTCTAAA
ATACGCGAAGTGTTCTTTTTTGGAAAGTTTGATATTGTAGATTGGAACTTTGCAATTCAT
TATTCATTTCATCCAAAACATTATTCTACAATAATGAAAAATTTGTCAGAATTAACAGCA
CCTGGAGGTAACGTATTAATAACCACCATGGATGGGGATAAACTGTCTTATTTATCAGAA
AGGAAAGTATTCATTATCCATAAAAACTTACCGAGTAGTGAAAATTATATGTCCGTTGAA
AAAATAAGTGATGATAAAATATTAGTTTATAATCCATCTTCGATGTCTAAGCCAATGGAA
GAATATATTGTAAAAAAATCAGATATAAAAAGAGTATTCTCAGAATATGGTTTTGTGCTA
ATAGATTGTGTTAATTTTGATACAGTTATTAATAGAAGTAAAAAATTCATCAATTATGTT
TCTAAAATGGAAGAACGACAATCAACAAAAAACTTTTTTGAACTTAATCGGGAAGCGTTA
AAGTATGACAATATAGATATAGAAGAACTTCTAAATTATTATGTTGTTTATGTATTTTCC
AAAAGGTAA
>LSDV 01 00079 D4L
ATGGAATCTGAAATTAAAAGATTAACAAATATTATTAAAGAAAGGAATATTATATGCCCT
ATAGATATAGAAAAAATTTATAACGAAAAATTTTTGTTATTGGAAAAAGGATTAGATGAA
ACCTTATATGCTATTCATATATATGATTCTATGGCTAGATTTGACAATGGTACATTTTTT
AAAATTGCAAAATTTTTATATAGAAATGATATAGATGTTTTAAAAAAAATTTTTATCGAC
GAAAAAAAAATAGAAAGCACAAATGCACTACTTCCTACATATAGTGTAAATATTATTGAA
CGTAAGAATGCGTTGATACCATTAACCGAGATACCAAACGGTTCCGAAGGTATTCTTTTA
ATATTGTTACACTTATTTAATTCGTTTAAGTTTAATAAACTAACTAATATATCAACTATT
CCATACTATTTACCTTTTGGAAAATACATAAACAACATAATAATTTAG
>LSDV 01 00080 group 58
ATGAATATTTGTATAATAAAAAATAACGAATATCCAAAAACTGATTTGCATGACGTAACA
TTTTTTCTATTGGGAAACCATAAAGGTTTTTTTGAAAATGTTTTAGAAAAAATTAATAAA
TATAAATATTTTTTTTCTCAATATGAAGTAAGTTCGGACAACGATGGTGTGTTAGAATTA
AAAAATGTAAAGTCTTCTCACATTATTAAAGATAGATATGTTACAACAAGAGAATTTATT
TTGTCAGGATGCCAAAAAGAATGGTGTAATGAAATTATTCAAAAAAATGATAATATCACT
AATTACGATACAATAATAATACAAGATATTACTTATTTTGAAAAAAAAATATGGAAGAGG
TTATTATTTGTCAACTGCCCAGAAATGAAAAATAGTTCATATGAAATCTTTTTAATAAAT
CCATTTGTAATAAAAAACAACACAGATATATTTAAAAATTTGATTTTAAGGTCATCGATT
AACTATTTCATATTTGATAATTATGCGCCACTGAAAAAAATTTTAACGCATATATACCCA
TCCTTTAATATAGAAAAAACGTATATATATATAAACTTTAACGAAATAAATAATATTAAA
ATAATAAAAAAATGTTATCATAGAAATAAAATTAGAGCTTTCGTTTATGCGTGGTTTAAT
TTTCAAATATGTGAAAATAGTGAAATAGAAAATGAAAAGGTAAAAAAAAAAATACAATGAT
TTGTATAGCTTAATATGA
>LSDV 01 00081 UNG
ATGAAAACCATAAAAACAAAATCTTTTCCTTATTGTATAGAATATCATGACGATTGGGAA
GCCGTTATTAATCAATTGGTAGATTTATACGATGAGATAGCTGAATGGATTTTAAAAGAT
GAAACATCTCCAATTCCGGAAAACTTCTTTAAACAATTACGGAATCCACTTAATGATAAA
CTAGTGTGTATATGTGGAATAGATCCGTACCCAAGGGATGCGACAGGTGTTCCGTTTGAA
TCACCAAATTTTTCAAAAAAAACAATTAAATCAATAGCTGAAACTATTTCAAATATAACA
GGTATTAGTAACTATAAAGGATACAATTTGAATAATGTAAAAGGTGTATTTCCGTGGAAT
TATTATCTAAGTTGTAAAATAGGAGAAACTAAAAGTCATTCATTACACTGGAAAAAAATT
TCCAAACTTCTTCTTCAACATATTACTAAGTATGTAAATATATTGTACTGTTTAGGAAAA
TTATCATTTAACGTTATTGATCTTCTTTTACGAGATAAAAAAACTGTATTTTATAATGAA
GATGATGTATTATGTATTATATTACAATGGCTTAATAATGAAGAAAATAATAAATATTTT
CATGATGTTTTAAAAGTAATAAGATTTTCACTTTTGTCAGAAGAATGTGCCATCAAGCTT
AAGATAAAATATAAATACTTATTTCCTGATAGTTTATTAAGTAAGTCATTATTTTCCTCA
AAAATGTCACGCAGAGAATCAACGTTTGGATCGTTTATATATATATCCGACCCAAATTGG
TATAGTAAAAAATATGATATTATGTACATAAGAACATTTAATTATATTACAAAAAGAGTT
AAAACAATAGATAGTATACCGTTTGTTGAAAAGTTTCATTCTGTATTACATAATGACGTT
ATTTACTTTTTAAGATTTGAAGGTAGTAATAATAAAAATAAATTTGATAGGAATTTTAAC
AGTTATGATATTGTAACTAAATCATGGAACAGTTTTCCAAAAAATAGACGATTGTGAAAAT
TTTTCATCCTGTGTATTAAATAATAAAATGTACCTAATTGGTGGTGAAATTAATGGTATT
TCAACAAATAGGGTATTGTGGTGGGATTTTAAATCTAATTATTGGAACCAAACAACTCCT
ATGCGTTTTCCAAAATCTGAATCATGTGTGGTACCGGCTGAAAATTTTATATTTGTAATA
GGTGGAAAAGATATGTACTCATTAGATGTAGTTGAAAGGTTTGATACAAAAACACAATCT
TGGTCAACTTTAATGCATTTACCTATACGATTAAAAAGAAGTTCTGGTATATATCATAAA
GGATTTATTTATATAGTAGGGGGTATCAGTTACGCTAGTGCTGAGCTCGGAATAGGCTAT
GAAGGTTTTGTTAATAAAATATATAGATATGATATATCAAATAACTATTGGATTGAATTA
AACCCATTAAGACATACAAAAATAAATGTTAATCTGGGTATATTAGATAATGACAATAAA
ATATATGCAATTGGAGGCGATAAAAATAATACAATAGAAGTGTATAATATATCAACAAAT
ACATGGAGTATGTTTGGTAGATCGTTTTGTAATTTAATTAACTCAAAGCAATGTAATATA
TTTACAAAAAGTGTTTTTTTGTAA
>LSDV 01 00144 group 12
ATGATTTTGTTATACGATTACATTACGCTTACAAAATCTAGAAATATCAAAGTTAAAAAT
ATAATAAAACTTATAAAAAATGATCAGCTAATTGATTTTAACTATAAATTATTTACGTTG
TGTGAATATGTGAGCAAAAAACATATTCGTATAGATGTTTTAAAAATATTGTTTGAAATA
GGTTGTAAAGAAAATTTACATAGGTTATCATATTATACTCTTTTAAGTTTCTTTAAAAAC
TATAAAATTAAGTATAATTTTAATCATGTTAAAAGTATTATAGAATTAATAACTAGTTAT
GGCGTATCGTTTAATGATGAACCAATCAAAAAAGATTCGTATCCCATTTTATTTTTAGTA
AGTAATGAAAAATTTAATCATATTAGCCTTTATGAATATTTAGAAAATAATAATATAAAT
TTCAATATAGTAAGATCTGATGGTTATAACTTGTTGCATTTATACTTAGAATCCTGTAAT
AATATAAAACTAAGTGTATTAAAACTACTCATCAAAAATAATGTAAACGTTAACGGATTA
ACTAGATTTAGAAACCTAACACCGTTACATATATATTTGTGTAAAGGTTATTGCTTAAAC
TATAGTGTAATAAATTTTTTAATAGACACTGGATCTGAAATAAATTGCGGCAAGGAAACT
TTATTATATTCATTCTTTACTACTTGTGATAATGAAAAAGTATTCAAATCACTAACTACT
AAACTTATTAAAAAAGGAGCTGATATAAATCAAAAATCAGAAAGTGGATTAACGCCATTA
ATGGGATTTGTTGCATATTCAGATTTAGCTACTCCTAATAATATAAAATTCATACTAAGT
TGTGGGGGAAACCAATTAGTTGTAAATCCTCAAAGTAATGAAACTTTGTTACATATTTAC
TTACGTAGGTATGATGTATCATTGTCAACAATATCAACGTTGTTAGAAAGTGGCATTAAT
ATAAATTCTGTCAATTTTAAAAATTATACTCCTTTACACGTATATGTTGATAAAAATATA
GAACATTTATCTTTAGATGTCGTGGATTATTTAATCTATAAAGGTGCTACTACCGAAAGA
GTTAACAGGTATGATTGTTTTGCAAAAACTATATTAGAAGTATTTTTAGAAAAAAACAAG
TTTTTAAACAATTCTTCAAGATATTTTATTAACTATATTTTAAAGTATTTTCCAATAAAT
GAAAAAGATCTATATGGATTTACCCCACTTTTATCTTCTGTATATGTAAATAATGTAAAC
TTTTTTAATTATTTTTTGAGATTAGGATCTAGTATAAATGTAATTTCTGAAATAGGTGAA
ACATGTGTTAGTATTCCAATTGAAAATCAGTATAAGCGTTTTTTAAAAATAGTTTTAGAT
AGTAAACCAAATATAAAAACAATAAAAGCTACTCTTAGATATCTTAGCGATAAGGAGTTT
AATACAAGAACAAAGTTTAAATTGATGAAACATTGTATTAAATATTTATTCACTTTGGAT
CCTGACGAATATAAAAATCATAGGTTTATTTTTTCAAAGTTCAAAAGTGTCGTCAATGAA
TGTCAAAAAGATATTATAACTATGAAAAGTACATTTTTAAAGACTGTATCTGTTTACGAT
TTAATATTTAACAAAAATGGAACAATGCATAAAAGGTATTTAAATACCATGGAAGTAAGA
AAATACTTAAAATCAAATATTTATGCTGAAAGAGTTAGATGTGTAATAAATAATTCAATA
GAAAAAGATAAAAAAAATTGATTCCATAATCAAAAAAGTTAATATATTATGTAAACATACT
ATTTGGGATATATTACCTGAAGAAATAAAAGACAATATCTTTAACTGTATGTCGTTGAGG
GATATTAGAGTAATCTTTCATATGTTTGTTGTGTATAAGCCATAA
>LSDV 01 00145 group 42
ATGAAAGATTGTCACGAAGATACAAAATTGGTAATTGTTGAAACTATACCATGTGATCTT
GACTTATTGTATAATGTTTCAACATATGATTTTTGGATGAAACTTATATCAGAAACAAAG
GAAAGTTTGTATATAGCATCGTTTTATTGGAGCTTATGTGATGAAGATAGTAAAAAAGAT
GAAACTTCAAATAACGGAAAAATGGTATTAAAAGAACTAATCAAGTTAACATCTAGAGCA
TCTTTAAAAATAGTAGTTAATAAATCTAATCAACCCTCTGAAGATTTATTATTATTATCT
AGTTATGGTGCACAGGTTATCTACGTAGATATAAAAAATATTTTTGGTGGTGTTTTACAT
ACAAAATTTTTAATTTCGGATGAGTTGCACGCATATATTGGAAGCGCTAATATGGATTGG
AGGTCTCTTTCTCAAGTTAAAGAACTTGGTATAGGTATCTACAATTCTTCATGTTTGGTA
ATGGATTTAATAAAAATCTTTAATGAATATTGTTATATTGGGTATACTAACGTGCCTTGT
TTTTGGTCAAGTGAATATCAACTATGTTATAATATCGATAATCCATTAAGTTTAATAAAT
AGCAATTATAAAATGTTTATAGCATCTTCGCCGCCATCGTTAAGTAAAAACAATGGTACC
GACGATTTATATTCGTTATTATCTGCCATAAAAAACGCAGAAAAATTTATTTATATTTCT
GTAATGAATTATATTCCAGTAATATATCAAAATAATAAAACACTATTCTGGCCTGATATA
GATATAGAATTACGAAGAGTTGCTATAGATAAAAAAATAAATATAAAGCTATTGGTTAGT
TTTTGGGATCATACACCTTTAATAATGAAAGGATTTTTAAAATCACTAAAAGATATAAAC
TATAAAAATATTAATATAGAGGTAAGATTTTTCATAATACCAAAAAATCAATTAAATATA
CCGTACACTAGAGTAAACCATACAAAATACATGGTTACTGATAAAGTTGCATATGTGGGC
ACGTCAAATTGGTCAGGTAATTATTTTACTGATACCTGTGGAGTTTCATTAAATATAATT
GATTTTAATGAATTAGCGATAAGGTCAAAGTTAGAAAATATCTTTATAAGAGATTGGTAT
TCCGTATATTCATTTCCTATTTCATATGTTACCACACCAAAAGATAATAATGAAAAAGTA
CCTATAAAACAAGATAGAGATTTTTTAGATTATAAGCTATAA
>LSDV 01 00146 group 110
ATGTCACTGTTATATGAATACGTCTTACATTCTCCAACAGTAGATAAAGATGCAGTAGAA
TTATTAATTAGAACGGGATTTGATGTAAATGAAGAAAATTGTGACAATGAATCAATATTA

GGAAGTTCAATATTTTGGTTAATTAACGGAGTTAATGTACTTGATTACGATAGAAGTAAA
AATATTTACTTAGGTAGAGAAATGTACGTAGGAAACTCTTATAAAATTGCATTAATCATA
AAAGAAGTTAAAAGAAAATATCTATCTGCTAATTTCACATGTAACTTACGCAACTTAGAA
TACAACTATCTCACAAAAAAAATTAAATATGTAAATTTAAAAACACTTTACAAAAATGAA
ATATAA
>LSDV_01_00013 group_70
ATGTCATCGAATAGCGATTTGGCATTTTGTTACGTTTTACCTAACATTAATGAAGTAACA
GATGGTATTGTGTTTATAAAAGATAACATTGTATATGTAAAACTAATCAACTATGGTTTG
GAAGCACTTGTAATAGATTATGTTAATATAAACATGGATCAAATGAATAATATAAAAAAA
ACATTAGTTAATAAATTAATTAATGTGCAAATTATAAGGATGAACAAAATAAAGGGCTAT
ATTGATGTAAAAGTTTATAATAACAACTAA
>LSDV_01_00014 group_72
ATGATCTCTTTCAAGAAAGTAGTCATTTGTCTGACGTTATTTACAACCTTTTATGTGGTT
TATAGTAAATGTGCAAAAAAAAAGGGATTTAGTTATTTACTTTCCTCATAAAGAGGGCGAA
AACGTTTTACTACAATGTAAAGGATATAGTCATCATAGCAATTATGCATATGTGTATTGG
TTGATAGGAAATAATAATTCGTTTGTTGAATTTATGAACGGTGACATTTATAAAGAACGA
ATGTATTTTAACAAAAAACCTTTAAAATGTGGCAAAGAACCTAGATCTGATTTGATAATT
AAAAACGTAACTGAAGAAATTAAAAATACTAACTTAACATGTGTTCTAATGGATTTGGAG
GAACCAATTAAAAAAACTTTAATATTGAACGATATATGGAATTGTTTAAATAATACATGC
GAGCACAAAAACGGTATTTCTGAAAAAAAAAGTTTCAGAAAGTGAGAGCAAAAATGTCATC
GAATAG
>LSDV_01_00015 group_23
ATGGAAGTAAAAAAAAATATTTTTTACCGTTTTGTTATTCTATAAAATTATATTAATGCTT
TCTAAAGAAAATAAAAACGATTACATAACTATCTTAAACAAAGATATAATGATGTGTGAT
AAAAGTATAAATTTTTGTTTAAATGGTGGTACATGTTATAAAACAACTTTTATTTTATCT
TACAATAAAAAACCACTAATGTTTTGCAGATGCAAATTAGGATATGAGGGCGTTAGATGT
CATTTAAGAAGTTTGTTTCATTATTTTTAA
>LSDV_01_00016 group_73
ATGGATAACTGCAATTACAACATAGAAAAAGTTTTGAATGTGTACTTGCGAGACTTAAGA
ATAGAATCATTAAATAATAATGAGTTGGCAATTTTAATAATGATACGCGAATGCTGTGAG
GTAATAAAAAAAAGATTATAAAACTGAGTTTAATGAAATATGTAATTTTATTTTGCGCAAT
AACGTTAAATCTTGTTATGATATAAATGATGTAAAAAATATAATTATCGAAACTATAAAT
AGTGATTTTAGACCTTCAGTAATATTAGCTTCTATATCGTTGTTATCCATTATCATAAAA
AAGAAAAAAAAATGAAAACAATGAAGTTGTTAATGATGATTTAGCACTAAATGAGCTAATA
AATACATTTTCTAGTTATCAAAAAGACATTATAAGTTTTGTTGAAAAAAATAAAAAAAAC
AATGAACACAATGATTTTATATTTTCAATAATTAACTTTTTCGTAATGGTAGGAAGTATT
ATTATTGCATATTATTTGCTTAAAATAATTGGTAGGATACGATGGAAGTAA
>LSDV_01_00017 DUT
ATGGATTATAGTTCGTCTGTCGTTAACTGTATACGGTTGTCTGCTTTTGCTAAACTGCCT
ACCAAATCAACAGATTTTTCTGCAGGATATGATTTATATAGTGCTTATGATTATATCGTT
AAATCAAAAGAAAGAATTTTAGTTAAAACTGATATAAGTTTATCTATTCCAAAAAAATGT
TATGGACGAATAGCGCCAAGGTCTGGTCTTTCGTTATATTATGGCATAGATATAGGAGGA
GGAGTTATAGATGAAGATTATAGGGGTAACATTGGAGTTATATTTATTAATAATGGTAAA
AATACATTTAATATTAAAAGAGGCGATCGAGTGGCGCAAATTATTTTTGAAAAAGTAGAA
TATCCAAAAATTAAAGAAGTTAAATGTTTAGATGGTACAAATAGAGATAATAATGGATTT
GGGTCAAGTGGGATATTATAG
>LSDV_01_00018 group_16
ATGACTTTAAAAAGGTATATCAATAAAGAATATGTAGAAGAATTAAAGTACATGTTATTA
AAAAATGATTATGATAGAATTTTAATATTTACAATTGGAGGTATATCACAAACAAGGAAA
GATATTTTTGAGATATCATCTAATTACTTTAAAAAAGCTATTAAAAAAATACAGTAACGAA
GTTAAGTTACCTTTTAAATATAAGCCATTTACGTATGTGTTAGAGTATATTAATACTGGT
TATATTACATTGAATAGCAAAAATGTTGTTGACATTTTTGCTATTTCTAACGTAATCGAA
ATTAAGTTTATAATGGATGCATGTACTGATTTTATGATAAATTACATTGATGATAGTAAT
TGTGTTGATATCTTTAGAAGATCTTATATGTATGGTTGTTACGATGTGTACGATGTTGCA
GATAAATATATTAAAAAAAGGTTTATGTATATAAGCAACGATTTAACTAAATTAAGAGTT
AATGAATTAAGGCCCATATTAAAAAGTAGTGATTTAGTTGTCGATACCGAAGATTTTGTT
CTTAATTTTATAATTAAGTGGGGAACTGTCAAAAAAACTAATAGGCTTAATACGATGAAG
TTAGTAAATGACTCTGTAAGATTTACCTTTTTAACAGAAAAAGGAAAAAAAAAGGTTACGA
GCGTGGTCGTCCAGATTTAAAAAAAAACCTTATGATTTTAACAAAAATAGTGATAAACAC
ATACCACGATGTAATTATAAAAATAAAGAATTTATTAATAGTGACAAATTTACAAAGTCT
TTTAAGTTTAAGGTTTATAATGATAACTTAACAAAAATACTGTTTACAAATGATATAATG
GATATAAATATAATAGATGAAAGTGTTTATGATAATGAACTATTGTATGAAAACAATTGT
AATGAAATTGATGAAAACAAAACATTAAATGAAAGTATTCAACAACCTAGTAACTTAAGT
AAATTAAGCAACATAATAGATGAAAATTTTAAGTTTTGTACTTCAATTGCTATTGGTAAT
AATATTTACTTTTTAGGTGGTGTGGATAAATACTTACGATCAGTAAATAGTGTATTCGCA
ATCAATGCAAAAACTTTTGAAAAAGAAAACTTACCTTCATTAATTTATCCAAGAAAATGC
CCAGGTGTCACTTATTTTAATAATAGAATATATGTTATTGGTGGAATCTATAATAATTGT
ATAGTTAATAAAGTTGAAAGTTGGTCATTTGGAGAAAGTGTTTGGAGAGAAGAACCAAAT
TTACTATATCCTAGATACAATCCATGTGTTGTTAATGTCAATGATACCATCTATGTTATT
GGAGGAATATCTGAATATGATAAATCAGTAGAAGTGTATAATTTGAAATATAATAAATGG
TCATTGGGTGAGTGTACAAAATATTCTCATTATGGTGGATGTGCTATATACCATCATGGT
TTAATATATGTTGTTGGTGGAATTTCATATATCAATAATATAAAAGTTTTTAACATGGTC
GAAAGCTTTAATCCTATGTCTTGTAAATGGAGAATTGAGTCGTGCCTGAACCAGCCAAGA
TTTAATGCATCAATATGTATTTTTGATGATTGTATTATGATCGTTGGAGGTTTTCATTAT
GAAAGATATATACGTGAAATAGAAATATTTGACGATAAAACAAAAGGATGGAACGTTATA
GGCGCATTAGACATAGATAGTGTTTTTTAA
>LSDV_01_00019 F4L
ATGGAACCAATTCTTAAAGAAACATCTTCAAGATTTGTAGTTTTTCCTATATTATATAAA

ACTGATTTTTCAAATATAAAATCTATAATAGAAGTTCCGGTCACTACAATAATAGGCTAT
CATCCCGCTGCTAGAGATAGACAATTTAGTAAAGATAAGTCTTTTGAAGCTATAAATATA
TTATTAGAAATTAATGGTAAAGAAATAATACATTGGGAAGAAGGATTTTGTTATTAG
>LSDV_01_00082 group_202
ATGGCAAGTCATTTAGTCAATAATGAGTATATATTTGTTTTAAAAAAAATTGGCGTTCAT
ACATCTCATAGAGAGAGTGAAGATCCGAGGTTTGTTGATATTTTTACATGTGAAGAATTA
GAAAACTATATATTAAAAAATCCATCATGTACATTATTCGAAACATTAAGAGATGAAGAA
GCATACTCCGTAGTAAGAGTATTTTTTGACGTTGATTTGGATATTGTATTAGATGAAATA
GACTATACTGCGGCTTTAGAAGATTTCATATTAAATGTTACAAAATTTGTATCTATATTT
TCTTCTCGGGAATGTGGTACGAATCAAAATATTGTGTTAAAATGTATGAGATCAAATTTT
TCGCTTACAAAATCTACAGATACTAGTCGTACTAGTTTTCATATAGTATTTCCCGATACT
TACACGAATATGGATACTTTAATTTATATGAAAAAACCATTATTAGAATTTTTAAGATCG
TCAGAAAATCCATTAATAAGATCAATTGATCCTGCAGTGTATAGGAGAAAAGCAACACTA
AGAATAGTTGGGACGCGAAAAAATCCAAACAATGATAAAATTCATATTAGACAACCGCCT
CACAACAATATATCTGATTATTTGTTTACTTATGTGAACATGAATGAAAAAAGTTGTTAT
TTTACATTATCAAAAAAAGTAGACGATAAAAAGGCAGATGTATTATGGGAACCAAATTAT
ATACACTTTAATGATGCTATGAAAAAAGTTTCTAAAATAATAATAAACGAAATTGTTAAT
ATAAACGATTTTAATGTGAATAACTTTACTAGTATTCCAATAATAATAGATTATATTATG
CCGTGTGCGTTATGTAAAAAAAAAAATACATAAACATCATCATCAACTTACATTAGGAAAT
GGAATTTTAAAAATATTTAAAGCTGGAAATCCTCACAGTTGTAAAGTTAAAGCGATAGCA
TTAGAAGGAAATAAGCTATTTACTATATCTCAGCTTATAATAGATTCAAATGTTATACAT
TTAACAGAACGCGGAGATCATGTAGTATGGATAAAAAACTCTTGGAAATTTAATACAGAT
GAACCCCTTATAACTAAATTAATATTAAATATGAAAGATCAACTACCTTCGGAATATATA
CCAGATATATTATGTCCTAGAAAACGTAAAGTAATTGAAAACAATTTAAAAGATATGTTG
GTTGATACAATCGAGACAGATACATACCCTTTTATGCTTCCATTTAAAAACGGGGTATTG
GATATATGTAGTGGTAAATTTTACAAAGGGGCAGAATCAAAAAAATTTATTTGTACTGTA
TCAACTGGATTTAATTTAGACATTGAACAGTATTTAAATGAGGATTCGCAAGAAATGAAA
GATCTAAATAATATTATAAACGATATACAACCTTTAACCGAAGAAAATAAAGAAAATAGA
GAACTATATGAAAGAACACTTTCTAGCTGTTTATGTGGATCAACAAAACAATGTTTGACA
TTCTTTTTTGGAGAAACAGCAACAGGAAAATCCACAACGAAAAAATTACTTCAATCATCG
ATAGGTGAATTATTTATAGAAACGGGTCAAACAATTCTTACTGATATTATAGATAAAGGC
CCCAATCCTTTTATTTCTAATATGCATTTAAAAAGATCAGTATTTTGTAGCGAACTTCCT
GATTTTTCATGTAGTGGAAGTAAAAAGATTAAAGCGGATAACATAAAAAAACTTACAGAA
CCTTGTATAGTTGGGCGACCATGTTTTTCAAATAGGATACATAATAAAAATCATGCATCT
ATTATTATAGATACAAATTATAAACCTGTATTTGATAAAGTCGACAATGCATTAATGAGA
CGAATAGCATTAGTAAAATTTAGAACACATTTTTCGCAATATTCTAATTCTGACTCTGTA
AAAAATAATGCAGCATATGATGATGTAAAACCATTAGACGAAAACTTAGATATGAAAATT
CAAAAAAATTACTTTAGGTATGCATTTTTAAACTTACTAGTTAAATGGTATCAAAAATAT
CACATTCCTACAATGAGATTATTTCCTACACCTGAAGCAATTCCAGATTTTGTATTTCAA
CTTAGAGTTGGTTCGTTACTCATCCCAAGTTCTAGCACACATTTAAAGTTTATGTCTTCG
TTATCTAAAATAGGATATGTATTAGAAAATGACTTAATGGTATTACCCCTTCAGTTATTT
CAACAAAAAATATCAAAACATTTTAATATAAAAGTATACGGTAACGACATTGAAAGTTTT
ATAACAAAACATAAAAAGTTTGCTAATGTTAGCGAAGAATACCTAGAGTATATATTTATA
GAAGACATCATATCTAAATGA
>LSDV_01_00083 VETFS
ATGAACTTAGGAATAATAAAATTGTTTAATAATCACGTAAATAGTATACCAAATATATTA
CCACATCAATTAGCTACATTAGACTATTTAGTTAGAAGTATTATAGATGAAAATAAAAGT
GTGTTACTTTTTCATATAATGGGATCTGGAAAAACAATTATTGCATTATTATTTGCGTTA
GTGGCGTCTAAATTTAAAAAAGTTTATATATTAGTACCAAATATAAATATACTAAAGATT
TTTAATTATAGTATGGACGTAGCGATAAATTTGTTTAATTCAGATTTTATTCTTGAAAAT
ATTTTTATCTATTCCACCACGAGTTTTTATTCTCTCAACTATAACGATAATGTAATTAAC
TACAACGGATTATCTAGATATAATAATGCAATATTTATAATTGATGAAGCCCATAACATA
TTTGGTAACAATACGGGAGAACTAATGACAGTAATAAAAAATAAAAACAAGATTCCTTTT
TTGCTACTTTCTGGCTCACCAATAACTAATACACCGATAACACTTTCAAACATAATTAGC
TTAATGTCAGATGAAGAAATAAATTTTGGAGATATTATAGTACAGGGGAAAAAAGTATTT
CAAATTTTGTTAAATGAGCACGGCGTCAATGTTTTAAAAAACATATTAAGAGGGAGAATA
TCATACTATGAAATGCCCGATTCTGATTTACCAATGATAAAATATCATGGTAAAAATTTT
TTAGATACACGTGTAGTATATTGTAATATGTCTAAGTTACAAGAAAAAGATTATATCAAC
GTAAGAAAATTATGCAATAATGAAATGTTTGAAAAAAATATGAACAATGTTTCGTTAGCG
GTATTAGGGCAATTAAATTTGATAAATAACTTAGATATTTTGTTTCAAGAACAAGATAAA
GAACTTTATCCAAATCTTAAAATAAGTAATGGTATTTTATATGGAGATGAACTCATTAAC
TTAAATATTAGTTCTAAATTTAAGTATTTCATTGACAAAATAACTTCATTAAAGGGGAAA
CATTTTATATACTTTTCTAACTCAACATATGGTGGATTAGTAATAAAGTATATAATGCTT
AGTAATGGATACTCAGAATATAATGGGTCACAAGGGACAAATCCAAAACTTATAAATGGA
AAACCTAAAATGTTTACAATAGTAACTAGTAAAATGAAGTCATCATTAGAAGATTTATTA
TCTGTATACAACTCACCAACAAATGATGATGGGAGCCAGATAATGTTTTTATTTTCATCA
AATATTATGTCAGAGTCATATACTCTAAAAGAGGTTAGAAACATTTGGTTTATGACAATT
CCGGATACCTTTTCTCAGTATAATCAAATCTTGGGAAGATCAATAAGAAAGTTTTCATAT
AATAATATAACAGAACCAGTTAATGTATATTTACTAGCTACAGTGTATTCTGATTTTGAT
GACGATATAACATCGTTAGATGATTACAGCTTGGATGAAATAAACACACTTCCATTTGAT
ATAAAAAAATTATTATATTTAAAATTCAAAACTAAAGAAACAAATAGAATATATTCGATA
TTGGAAAGTATTTCTGAAAATTATACAAAGGAACCACATCCGTATATTATCGAAATTGTA
TTAGGAGAAATTATAAGACAATTTTTTATCACCATTCCAGAATTAAGTTTGATAATAAA
CAACTAATAAAAGAAATATCGTCTGTGTTGCAAAATATAGAATTGGTAAAAAAATACATT
GATGATATTGTCAATGGACACTTTTTTGTATCGAATAAAGTTTTTGATAAATCGTTATTA
TATAGATACAATGATGAAATTATAACTGTTCCATTTAAATTATCGTATGAACCGTTTGTG
TGGGGAGTTAATTTTAGGAAAGAACATAATCTTGTATCATCGCCTTAA
>LSDV_01_00084 group_198

TTACTATATTTAAAAAGGAAAGATGTGAGTATTGATATACTGAGATTGCTACTAGAAAAC
GGTGCTGATGTTAACTACAGAAGCTATTATGATACTTCAATAACCAGTGTTATTAGGAAC
TATGAATTGGACAGTAATACTATAAAACAAATTATTATTCTACTATTAAAGTTTGGAGCG
GATATAAACCTAAAACCGTTCAATGGTGTTTATACAATAATGCCTTTTATATATATAATTTT
CATATAAATAATTGTAATATGTTTAAGTTTTTGCTAAGTAAAGGAATATATGTTAAAAAT
GATATAATTGATTCTAGAGGATTTAATCTATTTCATATGTACTTTGAAAGTTTTTCAGTT
AAAATAGATGTTATTAAAATTTTATTAAGTTTTAATGTTGGATTGTTTGAAAAATCATTT
AAAGGATTAACGCCAATGAATATATATTTTGATCATTTCATAGACGTTATATCGTTGAAA
GTTATCAAGTATCTAATTTCAAAGGGGGTAAATATTGAAACCAACAATAATGGTAGCAAA
TCTTTATTGGAGACTTTTCTACGATCTAACAAAATATTGTCAAGAAAATGCTTTAAGGTT
TTGAATTTTATTTTAAAATATGTAAAATTAAACAAAGTTGACGAAAAAGGACTTAATCCT
ATTCTGATATCTGCAAAGGCGGATAATTATGATGCGTTTAATCATTTACTGAAATTAGGA
GATGATATATACAATGTTTCAAAAAAAGGAAATACAGTCATAACCTATGCAATAAAAAGA
GAAAATATTGATATTCTCAATAGAGTATTAGCGATAGAACCCAAAAAATATCTTATAAAA
CAAACATTTGAATATTTTTCAAATTATGGATATGGTGGAATTAATGGATTGTTTTTAAGT
GAAAGAAAATCACTAATGATGATATTATTAATGCAGTACTCTTTTAAAGTTTATCCGATA
TTTTACAAAAACTTTTACGAGGTTAGACTTTTATTTCCAAACATTATAGAAATGTATAAC
GAAGATATATTGTTAATGGAAAAAGAAAAGGTTGGAAAAAATATATCTGTATATGATTTA
GTTTTTAATAGAGAAAAAGAAACTATCCCCGTAAAATTTTTAAAAAACGAAAATTTTTTA
AAATTTACTTCGTCTACTATATACGGTGAACGTATAAAACAAATAATCAATAACACTTAC
AGATATGAAAACTGTTTGAATACATCAATTAATATACTGAACAAATATTGTAACAAAAAA
AATTATTGGAATTATTTACCTACAGAAATAAAGATACACATATTAGAATATTTAGATTTT
TCAGACTTTGATACTATTATTAATCCAAAAAGAAAATCAAAGAAGTACTTTTTATAA
>LSDV_01_00147 group_32
ATGGAATACATTAGCGATTATGAAGAACTATTAAAAGAGATTTATATCAATCAATCAAAT
TTAAAAATAAATGTGCTAAAAAAGATAATAAATGATATACCTACAACATATCATAAAGGT
ATTTATAACAATTTATTACTAACAGTTTTGGGCAACAAAAGTATTAGTCTTAGTGTACAA
AAGTATAAGAAAATTTTGTCATTTTTAGTAGATAATGGTGCTGATTTAAACACAAAAATT
AAATACAAATATAATGCTTTACATTACTACTTATATAGTAATTCAAATGTTACAGTTGAT
ATAATAAAATTTTTAATAAAAAAAGGAGCTGATATAACAAAAAAGTGTAATGGTGACAAT
GTCTTACATACATATTTGTGTAATAAAAACATAGATTTTAAGGTTATTAAGTTTTTAGTT
AATAAAAAGATAGACTTAGGCGATAGGAATTTAGATGACCATACTCCAGTTAATATATAT
ATAAGGAATAAGAGAAACAATTGTGAGATCGATACATTGAAATTATTATTTCTTGTTGAT
TTTAATATATACAATAAGGAAGATATTTTCTTATCCGCATTAGATGTATTTTTAGATTAT
TTAAAATCATATACACGTAAATCCTTAGATATAGTTAATTATATATTAGAAAATATATCA
ATAAATTCTGTTGATTCTAATGGCTTTAATCCAATTTTATACGCTACAGTATCAGGACAA
AAAGTATTTTTTGATTATTTTTTAAAATTAGGATGTAGTATAAATATAACAACATCATGT
GGAGAAACTTGTGGATCCTTATCGTTGATGGATTGTGATTATTGTACTTTTAAAACTTTT
TTAAGCAAAAAACCAAATTTACAAACAATCGAAAATACTTTAAGTTGCTTATCAAATTAT
TTAGAGGATATTTACTATTGTGATTTAAAGTTTAAAATGTTTAAAGAATTACTTTTAGAA
GCTTTTATGTTAGATAGTGAATTTTACAATAGACACGAATCAATCAAATTATATTTTCCA
AAAACGATTTCATTGTATAAAGAACCGATAGTGCAAATGTGTAGTGATAAAATAGGTGAT
AAATCTGTTTATGATATTATTTTTAAAAACAGTGATATTAGGTATTGCTATAATGATTAT
ATTAACAAATATACTAACTTAAAATATTATGGAAATCTTATAAGGAAATGTATTTTAGCT
TCTAAGATGAGGAAAAAGAATATAGTTAAGATTATAAAGACAATTAATATTGCACCTTAC
TGGAATACATTACCAACAGAGATAAAAAATGTACATCATTAATTTTTTGAGTGATAATGAA
ATAAAGTTGTTGGCAATAAAATGA
>LSDV_01_00148 SERP2
ATGGATATATTTACGCACCTATCATCTATAGCATCTCCCGATTCAAATGTGTTAATTTCT
CCTGTTTCTATATCGTCAATACTTTCAATTTTACTTTTTGGGTCAAATGGTGATACAGCT
ATACAAATATCATCAGTATTAGAAAGTACGTATAGTAATTGCTGTTCGGACGATATAATC
ATTGCAAATAGAATTTATGGAGATTTGAAATTGCACTTAAAAACACAATTTATTGATAAA
TTTGGGAAAGAACTTATTTTGGTAAATTTTAATCATAATACCGAATTAATAAAAAATGAT
ATTAATGAGTGGATAAAAAGATTAACTCATGACAAAATAAAAAACTTGATAGATGAAATA
AGTGAGAATACAAAGCTGTTATTATTAAATGCTGTGTATTTTAAATTAAAGTGGAAAAGT
CCTTTTGATAAACAACAAACAAAAAATAGAAAAATTTTGGTATGATAATAGTAGATATGAA
AATATTGAAATGATGAACGATGTTAATGTTTATCCTTTTATTGAATTAAAAGAACTAGGA
TTAAAAATTATTGAACTACCTTATGAAAACAATTATTCAATGATAATATTGTTACCAAAA
GATATTAAAAAAATAGAAAAAAATTTGACTGGTAATAATTTAACTTTGTGGATCGACAAA
ATGAACCTATACGAAGTTAATGTAAAAATTCCAAAGTTTAAAATAGAAGAAACGTATGAT
TTAAAACTAAGCTTAATTAGTTTAGGTATTATTGACATATTTGATGGATCCGCAGACTTT
TCGAATATGACAAAAAACAAAAACCTATCTGTTGATAATTTTTACCATAAAACTTATATA
GAAATAGATGAAGAAGGAGCAGAATTATCCGCAGCATCATATTGTTGTGTAGCTGATTGT
GGATGCAATGAAAAAGAATTTATAGCAAACAAACCTTTTATATTTCTCATAAAGGATAAT
ATTAACGTATCTTTTCTTTTTATCGGAAAATTTAGTTTTCCCCATATTCGTTAA
>LSDV_01_00149 group_111
ATGGATACTAACACTCTTCCTTTTAGTAAGCACTATATTCATGATTTTACAGATTACAAT
AATTTTGAAAAAAAGAATAGTTTTTCGATGTCTTACCAACTGACAGGTATATTACAGTAT
TACTTTATTTGGAGATTTAATGGTGGTAAAGTTGATGAGACTACAGGGAAAATTTTTAAA
GAGTTTTTTAAATATGATACATTAGCAAAATGGAGATTCTCAAAAAGAGATTTTGAACAG
TGTAAGAATATGTTATTGGAAAATGATAAAAATATTTCATATGTGCTAGATGTTCCAAAA
CCAGAAAAAAATGATCCGTTGTTAAACTTAAAAGAATTAATTGGATTCTTGGCAATTTTA
TCAGAAGAAATTAAAAAAAAATACAAAGTTTTTCGCATTGTTTAATATAATGAATTCGTTA
TTTACGCATACAAAAATAGATATTGTAAATTGCTTAAAAACAGAATTACAATTTTTATAT
CCTTAG
>LSDV_01_00150 KBTB1_2
ATGTACGCCTTTTACGAACAACAGAGGTTTTGTGATGTTACATTGTTGTTCAACAATGGA
AAAAAATCTATTAAAACTCATAAAATAATACTATCATCTTGTTCAGATTATTTTAATGTT

GAAATATGGGTAATGTATAAAAAAGCTGTTGCTAGTTTTTGGACAGTTGATGAAGTAGAT
CTTTCTAAGGATTTAGCCGATTGGAAAAAACTCAGCAATGAAGAGCAATACTTTATTAAA
AACATATTGGCATTTTTTGCAGCTAGTGATGGTATAGTTAATGAAAATTTGGCCGAAAGA
TTTTACTCAGAGGTACAATGGTCAGAAGCTAGATGTTTTTATGGTTTTCAGATAGCTATG
GAAAACATACACTCTGAAATGTATAGCTTACTTATTGATACATACATTTCAAGTACTAAA
GAGAAGGAACATTTATTTAATGCTATTGAAACAATGGATTTTATTAAAAAAAAATCTGAA
TGGGCTAGAAAATGGATATCGAACAAAAAAGCATCGTTTGGAGAACGATTAATAGCGTTT
GCTGCAGTTGAGGGAATATTCTTTTCCGGATCATTCGCTGCAATATTTTGGTTAAAAAAA
CGAGGATTGATGCCAGGATTAACATTTTCAAACGAACTTATTAGTAGAGATGAGGGCCTT
CATTGTGATTTTGCGTGTTTAATTTTTAAACATCTAAAATATCCTCCTTCTAATGAAATT
ATTACGATTATTATAAACGAGGCTGTGTCTATAGAAAAAGAATTTTTAACCAATATAATT
CCTGTAAAACTAATAGGTATGAATTGTGATTTAATGTCTCAATATATTGAGTTCATTGCG
GATAGATTACTATTAGAACTAGGTTGTGATAAAGTGTTTATGTCAACAAATCCATTTGAT
TTTATGGAAAATATATCACTGGAAGGTAAAACTAACTTCTTTGAAAAAAGAGTTAGTGAA
TATCAAAAAATGGGCGTTATTTCTAACAAAGACGATAATGTTTTTTCGTTAGATGTTGAT
TTTTAG
>LSDV_01_00020 group_39
ATGCGTCAGATGAATTTCACAACAGCAATGCCTAACAATAATGCCTTTAGCATAGGAACT
GTACTAATTTTGATGGTTTTAATAATTGTTATCGTAATAAGTCTTTATTTGCTATTTCAA
CTGGTCAACTGCTTTTACTTATTCGATTTGTTTAATAAAATAAAAGTAAGAATGATGAAA
TTAACCACAAGAAGATCCTTTGACGAATTAGAAAATGTTTATTATATCGATGATACGTGT
GTGGGCGTCAATGTTGACTAA
>LSDV_01_00021 group_52
ATGGATAAAAAAATTTATTGTGGTATAGTATTTGGTTACAGATTATTAAAAAAAAGTATA
AAAGAAAAGGCTATTAAATGTGTTAGTTTTATAAGGAAAAGCAATATGAAAAAGGCAATA
TCTATTGAACGTGTTACATTGTTAGATGGTGATGGTGATGGTGATGGTGCTAATGATAGT
AATAGTGATAGTGATAGTAATACCGTTAGTAGTGATGATAATGATACTAAATATAATTAT
AACTACAAGTATAAATTTACAAAAAGAAAGACACCTCATAAAGTAATGTATTACATAAAT
TATGACGACAGTGTTTCAAATGATGATGTATTTCTATAA
>LSDV_01_00022 group_57
ATGAGCGAAAAACTAAAAAAGGTTAACAAAGTTGTTGTAAATGGAAAGCCATACAACAGC
AATAGTAGTAACTCTACCCCATCTAAAGACGAAATTAAAAAATATGGATATTGTTTAAAT
ATTAAAAAACCCTCATCTCACAATGATAAAAAAGAAACCTACCTGCATAACGATTATCCA
ATGGTAATAACAACAAATACTTCGTTTTATGATACATAA
>LSDV_01_00023 group_116
ATGGAAAACCACTTTCAAGTTGATACATTGTATAATTTATTCATAGAGCGCTATCTACAA
AATTTATCATTATACTCCGTTCCGGTTAACACAACTTGTGGTATTCACATTAGTGAAATA
AAGGGAACGCTTAAAGGATGTAAATTAAAAATAATAAATTTATGTATTGATGATAAAGAA
CTAAGTTTTTACTTATTGATAAAAACATTTAAAGAGTTTGCTAATACTCTATCTCAAAAC
GAGAAGTATGAATTAGCGAACGAAGTCGGGATTGATTTGGATAATCAAGATAAAAATTAT
ATACCCGAAATAATACGCAAATGTACTTCTTCGGCCGCCGTAACTAATGTGATTGATATT
CAAACTTTAGATATCGGAGAGTGTATCGCACCAAATGGAAAAAACATTTTATTACAAATA
ATTAATTCTGGAACTTCTGAAGCAAATTGTGTTATGAACACAATAATGAAATCAATGAGT
AATAGATATACAATTAATAATTATATCGATAATAGGATACCATTCATCAACGATGTTCAT
TGGTTTATTTTTTTTGTATTGATAATTTTTATTATTTTTTTACTTGGTTTATGTTCTATA
AAACGAAAAATAAACATTAAGTACAAATACGGTTCATTTTTATATGTATAA
>LSDV_01_00024 VPK2
ATGGTAATCATGGAAATAGATGATAACTCATTAGAATGCCAATGGGATAACGATGAAGAT
ATAAAATCTACAACAGTATTAGGCGATGATATTTATTTTGATTATGTAATATCTCAATTA
GATATTTACCAAAGTTGGTCACCTAATGTTAGGTTAATAAGATACTTTAAAAAGTTTACA
AAGGAAACATTAAATAAAATCGCTGAAAACGAATATATCAATCCATCGTTTTTTCAGCAA
AAAGATAAGAGATTTTATCCAATTAATGATGACTTTTATCATATTTCAACTGGAGGATAT
GGTATAGTTTTTAAAATTGATAAGTACGTTGTAAAATTTGTTTATGAACCTAATAAACAG
TATAGTCCTATTGAAACAACTGCAGAATATACAATACCAAAGTTCTTGTTTAATAACTTA
AAGGGAGATGAAAAAAAACTAATAGTGTGTGCATGGGCAATGGGTGTAAACTTTAAACTT
ACATTTTTATATAACTTATATAAGCGGGTACTATATATGATATTATTACTTATTCAAACA
ATGGATGATCAAAAAATTAAGTATAAGTAATTTTTCACATAAATATTTTTTAAAATCGTTC
AATGAGAAAAAAGGAGATGTAAAATTTGTAAAACTATTATCATATTTTTACCCTATAGTT
ATACAAAGTAATGTAAATGTAATAAACTATTTTACGCACATGTTTCATTTTTTTGAACAC
GAAAAACGGTCTAATTATTTATACGATAGAGGAAATATAATAATATTTCCTTTGGCAAAA
TTCTCTTCTGATAAAGTTAGTGAAAAAATGGCTATTGAATTAGGTTTTAAGTCATTAGTT
GAGTATATAAAATTTATTTTTTTACAGATGGCTCTTTTATATGTTAAAATATATGAATTA
CCATGCTGTAACAACTTTTTACATGTTGATTTAAAACCTGATAATATTTTAATTTTTGAT
TCGGACGAATCAATAAAAATATCATTAAATGAAAATACTTATATTTTTAATGAACCTATA
AAAGCTTGTTTAAATGATTTTGACTTTTCACAGGTTGCAAACATAATAAATAAAAAAATA
AAAAATAATATAAAAGTTGAACATAATTGGTACTACGATTTTCATTTTTTCATTCATACA
CTTTTAAAAACATATCCAGAAATAGAAACAGATAAAGAATTTTATAATACATTAGAAGAA
TTTATTATATGTTGTAATAAAAATACGTGTGATAAATTTAGGTTAAAAATATCAATATTA
CATCCTATTTCTTTTTTAGAAAAAATTATTTCAAAGAAAAACATTTTCTCAACGTGGATA
AATGGAAAACCACTTTCAAGTTGA
>LSDV_01_00025 group_9
ATGTCTATTATGTGTTTAAGTAATAAAAAAAAAGAAGAATATATTTTGAAGTACTTTTTA
AATAATAGATGTTATGATGATTTATATTCAGTTAATGGTGGGAATGTTGCAGTTGGTAAT
TTTTTAAAAAGTAAAAGTGAAGAGTTATATTTTTCTTTTAATCTTTTTTTAAATGACTTA
GATATAAGTAAAAAAAAAGTAGGATTTTATTCATTGAAAGAAATAAATGTAAATATTTTT
TATAAACTTTTACTCAATGAAGAGTTTTTTACAGTTATAGGATCGTATTTGGTTAAATTA
AAACACGGAAATGCATTTTTTTTAAACGAATCAACATTTAGTAATAGCGTTGTCATTACG
GCCTGTGTTAAGAATAAAGGAATATCTGGATTATTTATTCCAGATACTACATTTTTAAAA
ATGTCAACTTTTATAAAAAATGTTTATTTGCCTATTAACTTAAATCCTCATGAGTTAACA
TTAGACATAAAAAAAAATATAAAAGATGCAATTTATAAAGAATACTTACATCGAGAATCG
GGTGGGATAATGGCTAAAAAAATTGATATATGTTTTGATAAAGAATTACCACTAGGTGAA
ATTATAAACAATCACATAATGGTAAAAGTTCCGTGTATTGTTACGTATAAATATTATAAA
AATGGAGACATTGTTAGTGGCACATTAAACATAGAAGATGAATCAAATATAACAGTTTTA
TGCGGAGATTTAGTTTGTAAACTTAATAGGGATTCTGGATCTGTCTCTTTTAATGATTCC
AAATACTGTTTTGTAAGAAATGGAATAGCTTATGATAACGGATCAATGGTCACAGTTATA
TTAAAGGAAGAACAACAAGGAATGAACTCCAACTTTGTTTTTTTAGCATCTATAATGGAT
CCATCTAATTAA
>LSDV_01_00085 D9R
ATGTTGACAACAATGATCGAAACAGATAGAGAAAACGTTACAATTGAAAGAGTAAACGAA
ATACCAGATGAAAAAAATACACACGTTTTCGCTATTTGTATAACCAGTGATGGTAAACCA
TTAATTGCTGCAAGGAGGAACTCGTTCGCATTTCAAGAAATAATGTCACAAAGAGTGTCA
CCTACGTCGATATTGAAAGTATCTAAACACTTATTAAAATACATGTATAATAACGAAATA
AAAGAAATTCAAAGAAGACTTTTAAAAGGTTCATATTTAAACGTATCTCAAATGACTAAC
AACTCGTTTGAGGAATTAATATTATTGGGTGGAAAAATAAATAAATCTGAATCTATAAGT
GAGTGTCTACAAAGAGAAATACAAGAAGAAAGTGATTCTAATTTAACAATTAAGTGTTTT
GGAAATAAATGTGTGAAGTTAACAATATTTGATAAGTTGTTTGATAAAACTTATATAAGT
TATTGTACAACATGTTATATAACAGAAACAATATCGCAAGCTATATCTTCCATTATATAC
AACGTCGAAATTAGAGAGCTAAAGTCGTTGTTAGAATGTTCTAAAAATGATAAGTATCAT
TACTTATTGTTTATTTATAATACGTTGGTTAATAGTAAATAA
>LSDV_01_00086 group_79
ATGGATAATTATTATCCTTCTAGTTTATTTAATGATATAATCAAACGGAATAGAAAGTTG
TCAAAAACATATATATTATCTAGCAATAATCAAAAAATATATGTTACTGGTTTTAATAAT
CAAAGCTTGGATAACGTAAAAAAAGTTTCTATTTCTATAGTAATGTTAACAAGTGATAAC
AAATTTTTAGCATGTTCTAGAAGACATAGTTTTTTATTTACAGAAATCATAAGATCAAAA
AACATATTTCGTAAACGGAGATTATTTTTAAAATATTCAAACTTTTTAAAAAAAAACGAA
CGTATTATTTTATCATCCGAATTAGATATTAAACTGTCTTCGTCAGACCATGAAAATGAT
TTCAGTAATATAATATTTCCAGGTGGGGTAATTAGAAACGAGGAAGACGTTATTAAATGT
CTTTCAAGAGAAATTAAAGAAGAAGTTAATATAGATAGTAAAGATATTTTTTTAGATTAT
AGATTTTTTATACACATGTTAATAGAAGATCTTCTAACAGATAGGTTTTATGAAAACATA
TTATTTTTTGGAAAAACGTTTCTAACGAGTAATGAGATAATACATAATTTTTTTCTAAT
AAAGAAATAAAATCATTAGTTTTTTTCGATTCTTTATATAATGGAATAGAAGGAGATATA
ATAAGATTTGTGTTAGATATTTCAAGATTAAAATGTTTTGGAAATAAGGGTTATGAATTA
TATAATAAAAACACTTTTAAAAGTTTAAAAAGCTTTTTTTAA
>LSDV_01_00087 NPH1
ATGAGTAGTTATCACGCCGCATACATAGACTATGCGTTACGCACTACGGAAAAAATGCCA
ATTGAAATGGTTGGTCCAGTAAACAAAATAAAACTAAAATCGTACCAACATTTTGTTGCG
AGAATTTTTTTAGGATTAGATAAAATGCATTCGATATTACTATTTCACGAAACAGGTGTT
GGTAAAACCATAACAACTGTCTTTATTTTAAAACATTTAAAAGATTTTTATACAAATTGG
ACCATAATTTTATTAGTTAAAAAAGCACTAGTCGATGATCCATGGATGAATACATTAATG
AAATATTCTCCCGAGATAATCAAAGACTGTATTTTTATAACATATGATGATAAAAATTTT
CACAATAAGTTCTTTACAAACATAAAAACTATAAATGTTAGAAGCAGAATATGTGTAATA
ATCGATGAATGTCACAATTTTATTTCTAAATCATTAATTAAAGAGGATGGAAAAATACGT
CCTACAAAATCTGTATATAATTACTTAGTAAAAAATATAGCGTATCACAACCATAAGCTT
ATATGTTTATCTGCGACACCAATTGTTAATAACGTTAGAGAGTTTACCATGTTAGTAAAC
TTACTAAGACCAAAAATATTACAAACACAATCATTATTTGAAAATAAAAAATTGGTTAAC
GAAAGAGAACTAATAAACAAATTAGGAGGAATTTGTTCATATATTGTAAATAATGAATTT
TCTATTTTTGAAGATGTAGATGGTTCAGATTCTTTTGCTAGAAAAAAAGTGTATATGAAA
TATATAAACATGACACCAAAACAAGAATTTATTTACCAAAAAGCTAAAATGTTAGAAATT
AAATCTGGTATATCATCGTTTAGAATTTATAGAAGAATGGCTGCAACATTTTCGTTTGAC
GTGTTGCCAGAAAAAAAAAGATAGAAACTTAGATGATTTTAATAAAGAAGTTAATACACTA
TATAAAGATTTTGAAAATTCTATCAAAGGTAGATATTTTTCCGATAAAGCACTGGATTAC
TTCAAAAAAGGGGAAGAATTAAAAGGCGACGTTAATGCATCAGATTTATCTTTGTATAAT
GAACTAAAAGAAAAAAGTTGTAAATTTACAGAAGTGTGTTTATCCATACTTTCATCTCCT
GGAAAGTGTTTGGTTTTTGAACCATTTGTGAACCAATCAGGAATTATAATTTTATTGCTA
TATTTTTCTGTATTTAAAATATCTTATATAGAATTTTCTTCACGTACAAAAAATACTCGT
GTGCAGATGGTATCCAAATTCAACGAAGAAGTAAATACTGACGGTACAGTAATTAAAGTT
TGTGTGTTTTCACTTAGTGGAGGTGAAGGAATTAGTTTTTTTTCTATAAATGATATTTTT
ATACTAGATATGACATGGAATGAAGCATCTCTAAGACAGATAATTGGAAGAGCTATTAGG
TTAAACAGTCATGTAAATACTCCTAAAGATAGAAGATATGTTAATGTGCATTTTATAATC
GCTAGATTATCAAATGGCGAATCTACAGTTGATGATGATCTATTAGAAATTATACGAACA
AAATCAAAAGAATTTTCTCAATTATTTAAGGTGTTAAAGGAATCATCTATTGAATGGATA
TACGATCACCAAAAAAACTTTTCACCTGTAGATGATGAATCTGGGTGGATACCACTAATA
TCAAGATCAATTGATATGAATGCTAAAACAAAAGAAATAGTTAAAGTTGTAGAAGGTGAA
AATATATGGTTTTCTACATCAAAAAGATTGGTAAATATACATAAGGGATTTAAAACCAAC
GATGGAAAAATTTTTGACGCTGAAGGAAACTTTTTACAAACAATGCCAGAAAATCCAATT
ATAAAAATACAAAATAATAAACTGATTTATATATTGCCAGACGACTAA
>LSDV_01_00088 group_215
ATGGATGACGTTATAAAAAATATTAAAGAAGGAATTTACATTCTGTTGCCTTTTTACGAA
CATTTGCCAGATTTAAACTTAACTTTAGGGAAAAGTCCTCTACCTAGTTTGGAATATGGA
ACTAATTATTTTTTACAATTATCAAGAGTAAATGATTTGAATAGATTACCAACGGATTTA
TTAAGTTTATTTACACATGATTTAATGATACCAGAAACCGATTTAGAAAAAGTATATGAA
ATTTTAAATATAAAATCTATAAAATCTTATGGAAAAAATATAAAAGCCGACGCTGTCGTT
GCAGATTTAAGTGCTAAAAATAAATTTTTTAAAAAAGAAAGAGATTTAATAAAATCTAAT
AACTACCTAAACGAGAATAATCTTTATGTGAGTGACTATAAAATGTTGACATTTGAAGTA
TTTAGACCGCTATTTGAAATGTCTGTAGAAAAGTTTTGTATAATAAAATTACCAACGTTG
TTTGGTAGGTGTATTGTCGATACGTTAAGGGTATATTGTAGCTTATTCAAGTCGGTACGA
ATGTTTAACAATAATTTCATTGAAAATATTATAGATGAAATTAATATTTATATAGATATA
GAAAATGATAAATCGTTAGATGAAGTTATAAAGTTTATGTATTTAAAAAAGTTTAATTAT
CGCATAAATAATTATAAAATTTTAAAGGAAATGATATTTTTAGCAAATTACTTTATTATG
GATAATTTGGTAAATTTATGTATTGCGTCTATCATAAAATTTGTCGATGTAGATAATTGT
ATAGAGATATACAAATTTTCACAATATTATGGTTATAAAAAATTGTCATCATATACTTAT
GATTATATAAGATGTTATATAGAATCAATATATAATCAAAATGAATTTACAACATTAACT
ATTAATGATATTATGAAATTACTATCTGATAGGAAAACTATCGTTGCATCGGAAAATAGT
GTTGTTGAAATACTAGTTAGTTGGTTAACTTCACAAAATAGTATAAGTTGTTTTGAAATT
TGTAAAATACTCAAACTTATTAAAATACCATTTGTTACAGAAGAATATTTTAAAAAACTA
CAATATCATCCCAAAATATTATATAATGAAAAATGTAGAAAAATATTAAGAGTAAAAAAT
AATAATAATATAACACCAAGAGCTTCGACATTGGGTTCTATTATTTATATATCTAATCCC
AACGATCATTCAGGCGGAAATCCAATATTTATGGCAATATATACGTATAACTTTGTCACC
AACGAAATAAAAGCTATAGATAATATACAGTATGCAAATAACTTCTGTTCGATATTTTAT
AATAATATATTTTATTTTATAAATTTTTCATACAGAGTAAGGGATTCGTTAGTTCATGAC
GATTTCCAAAGTTATAATATTATCACTAAGGAATGGGGAAAAATTCCCAAGATAAGTGAT
AGAAAGGACTTTTCTATAATAATTTTTAATGAAAAATTATACGCAATTGGAGGAATAAAA
AATGGATCCGTTGTATCAGATGTTAGTTTTTGGGATTTAACATCATCAAAATGGGAAGAT
GCTCCACCTCTTATATTTCCAAAATCTAATATGTCTTTAGCAAATAATAACGAATACATT
TTTGCAATTGGAGGTAAAAATCATGAACTACTTAATAATGTTGAAAGGTTCGATATAAAT
ACTCTTAAGTGGGATAACGTAGCACCGCTTCCTATACCCTTATATAATTCATCCGCTATA
TCATATAAAAAAATACATATATGTTATTGGGGGAAAAACATATATAGATTTACCAGAACGT
TACAACATCGATCCAGTAGATGGCTCTAGTAAAAATTTGTTTATGTATAATATCGAATAT
AATGTGTGGAATGAACTTAATATGATGATATTTACAAAGGTTTTACCTAGTTTGGCGATC
ATTAATAATAAAATTTACGTAGTTGGTGGAGATAAAAATAACCTTATAGAAGTGTATGAT
ATAAAAAAAAATTATTGGTATATTTTTAAAAAATCATTTCCTAAATCACTTTCTAAGCAA
GAGAATATTTTCACTAATAAAGTTTTTTTATAA
>LSDV_01_00151 group_62
ATGGATATTGATGATATATATAATTATATAGCTTTTAGGGGCAATCCTAAATTAAGTATA
ATTAAATCATGTGATCCAAACGAAATAGTTAATGAGTATTCATTGTTCCAAAAATACTTA
CAACGAGATTCGCCATCGGTCGATATTGTAAAATTATTAATTAATTTAGGTGGTAATGTT
AATGGATTAGAAAATGAATACTCAACACCTTTATGTACAATTTTATCAAATATAAAAAAA
TATAAACATATGTTAGATGTTGTTAAAATATTAATCGAAAACAATGCAGATGTTAACAAA
AAAAATTCCGATGGAGAGACACCTTTATATTGTTTACTATCAAATGGATATATAAAAAAT
AATAAAGAAATTTTGTTATATATGATACAAATGGGTGCCGAAACAACATTGCTATCTAAA
GATGGATATACAATGCTTCAAGTATATGTTAAAACAAATCATCATATCATTGACATAGAA
ATTATAAAAATATTGTTAGAAAGTGGTATTGATATAAATACAATAAGTAATAAAGAAAAG
TATGATACATTAGATTGCTATTTCAAGTATAATATCGATAGGATAGATGCAAATATTTTG
AAATTATTTGTTGATAATGGATTTATAATAAAAAAAGAGGATAAATCTCATAAAAAAAAA
CTCATGGAATACTTAAACTCATTATTATACGATAATCGAAGAGTTAAAAAAAATATACTC
GATTTTATATTTACATATATTAACGTTAATCAAGTAGATGAATTAGGATTTAACCCACTA
TACTATTCAGTTTCTCATAATAATAGGACGATATTTGAATATATTATCCAATTGGGTGGT
AATATTAATTGTATATCGGAGTTAGGCGACACTTTATTATTTAAAGCATTTGAGAATCGG
AGTCTTTTCATATTTAATTCAATACTTGAAAAAAAACCAAACAAAAAAACAATTTCTTAT
ACATATTATAAACTAAGAAAACATCTTTTAGATGTAGGCGATTTTATTAATCAAATAGAG
TTTGATATTATTAAAAAGTTTATAGCGCACGTAATTTTGTATGTAAAAAATTTCAGCGTT
CGAAATCGAACAAAAGCATTTATTTATTTTGATGATTTTATAGAAAAATGTACAAAATCT
TCTAATTACATTCATAATACGTACATAAATAATGAAACAATTTTTCAACTTTGCTTTAAT
AAAAAATATATACCAATAAGTGTGATAAGTAATAACGAAAAACTGTTAAAAAAACATACA
AAATTATTTTACTATGGAAATATTTTAAAGAAAAACATAGAAAAGTCTAAAAAATATTAT
GAAAATATTTACAAGGTATCGTGTTGCATTTCTAATTTATGCGATACATGTAGTTATTGG
AATACAATACCGTTAGAAATAAAATTTAAAATAGTAAATAATTTATCACTTAATGACATA
GAAATGTTTTTGAAAAATAATAAAAAATAG
>LSDV_01_00152 group_162
ATGATTTCATATATTTTAAGCCCTTTACTAAGTGTTTTTTATTTTGTTATAGGTAAAGTA
ACTAATCTTGTTACATTTTTATTTTTGAAGTTTTTTTGGGCAATGATAGGGTTAATGAAT
CCTTATAACATGATTACCTCATCGACAAAAAATCTGTTTTCGTTTAATATAAACCCCTTT
AGGCAAAAGAAAAAAAGTTTCAGTTTTCTATCATATATAAATCCTTTTAAGAAAGAGGAA
AAAAAGAAAGGTTTGTTTTCAAGTTTTTTTAGTTAA
>LSDV_01_00153 group_190
ATGTCATCATTAACTTTGTTCATTTTCTTTTTGCAACTATTCATTTTTACTAGTAGTGTA
AGCGGAATATCAATAAAGAGATGCACAGAAGAAGAAAATAACACATGGGAAATCGAAGTA
GGATTATGTATCCAAACAGAAAATTTTAGAGCGATTAAAACTGGCTGTTATAAAATTCAA
GGACCTGGAGGACTTCTAACAGAGGGAAATGGATTTAAAATTTTCGCACATGATGATTGT
TCCAAAGAAGAAACACAAAACAACTTTATATTAGATAGTGTTAACGAAGCAGTTTATGCA
TTGGGTAAATATGTATATATGGAAATATCAACCAGTAATATAACAACTCTGTATTCGCTG
CCACAGTGTGCTAAGAGAATATCATTGTCAATTTCTTGTGATCAAGTAACTACAGAAATG
AAATCATACGTTGAGAGTGTAAGTTTTAAAGATTATGATTTAGAATTTGTTATAACAACA
GATATTAGTTGTGTAAAACATGTAAGTTCAAGTGTTATAGTTAGAAATGAATGTGAAAAA
AAATATATATCTACAGGAAAAAAGATTTTTGGGTTTAATAATAAAATAGATTGCTCAGCT
GTAAAATTTAGTGAACATGTAAATTATTTAAAAACGTGCAGTGTTGGGAAATTTGATAGA
AAAAAATATTATGAACATCAGCATAATTATATTAAAAAAATATTTCATCATAATGAACTA
TAG
>LSDV_01_00154 group_37
ATGTTTGGAAACCTAAATCCGTTCGATTCTGTCTTGGATAAGGATGAAAAAATCCAAGAC
AATATAAACACTAATCACAAACCTATAGAAAGTAAAGAAATAAATAATGATCTACCATTA
ACTGTATTAGATAAAAAAGATACAAGCGATATAAATATTAGAAATGATAATGGAGTTTTT
GATTTTATTAAAATTCCTAATCCATTTAAAAAACACTACGAATACTACTGTAATCAAAAC
ACAATAAAGGAACCACCTAGAAAAGGATTAGTTGAAAGGATGATGAATATGGTTGAATAA

ATAAATATAGACGAAGGAGATTTTATTATGAGTGGCTTTCTTAATGGGGTTAATTTTTA
CCACAAATAGGTGGCGAATCAATTTATTTAGTGACAATCTTATCTCCTAAAGAATTATCA
ATTACAGAAAAAGCATTTTTTTATAAAAAGAATAATGAAGAAACATTAAACATAACATCA
AATATTATTTCAATTAGAAGATTAAAAATAATCGAATTAGTTAATAAAATTATAAAGTTA
AAAAAGGTAATGAAAAAAGAAAATAATTGTTATATTCCTAACAACTTTGTTATTTATGAT
ACTATTTTAAATAAAAATAATAACTTTTTGTATAGTTTCAAAAAAAAGTACAAGATCGAC
GATGATGAAACCCTTTGGGAAAAAATCACAAATTTATTTTTAGAAATAGACAAACTTTTA
GATTATAATATAAAACATTATCAATTGATTAACGAATATATAATAAATAAAGTAAAATAT
TATTATTAA
>LSDV_01_00026 group_85
ATGTTTAAAAAACAAAACAATGAATTTAATATTATTAATGCATTAGAAACATATAAACAC
TCTATTATATTAGCGAGATTAGGAAGCGGAAAGGGAGTTATAATATACCTAAATAATTTA
GATTTAGTACGATCTGTAACAAAACTATCTCATTTAGAAATAGTTGGAATTACAAAGTAT
GCAGAACCATATTTTATTGACATGCATATGCACAAGTTATTCATTGACGAAGTCGACACT
GAAACATTTTTTTCACCTAAAACATCTATATCTCCTTTGGTAGATATTGTCAAAAGAAGA
GCTTTAGAAAACGATAAAATAAATAAATCTGTTCATAAATTGCATGTTTTTGAAAATAAA
AATATTTCATTGAGTGAGATAAATTATTGGATGTGTGATAATGGATTATCTAATTATAGA
TTTGTTAATTATAAAAATAGTAAAAAAGGTAATGATTATACATTAATTGATGAAATGATA
ATAGGGTGCATTGGAAAACATTATATATGGGTTAAACAAAAAAAAAAATATAATAGACCG
GAACTAGACATATTACCTTATAATGTTAAAAGTATCGCATCGAAAAAGTTATGGTCAAAA
AATAACATATATGATGAAGTAGTTACAATAATACCTATAATTATAAACTCAATAATAACT
CCATTAGGTCCATCAATTTATATGATGACAAGTTTTGGTAAATCATTTATAAATTTTAAC
TCAAAAGAATTAATATTATGTTTTTTAAAATGGTTAAAGGAAACAATACTAATAAATATT
AAAACAGCATTTTTAGTAGGGTTTTTAAGCAATGTTTTTGACTTTTTACTGCTTAAAGCG
TCGTGGCCAAAAAACATAGGATGGAATTTTATAGGAAATGATTATATTGTATCAGATGAC
GGATTCAAACTAATATTAGTAGATATCGCTAACTTTTCGTGTAAAATGGAAATAAGTGAT
TATTTTAAACAGTGGAATGATAAAAAAAAAGATAATTATACCAAAAGATTTTATCACGGAA
GAAGAAGCAAAATGTAAAATAAAAGCATTAGAAAAAATGGCATTAGAGTCAGTTTTGGTA
TTATATGATTCCATCAACAAACAATATGATGTTTTAAATAATTTAATATATCCTTTTAAT
ATGTTTTCTTTTAATAGAATAGAAGATATTTTTTTAACTAAAATAATACAAACATCACTA
TCTAAAGTAAACAATTTTATGTATTATCCACTAGAACACGACACTGTACGATTTATTATG
CAGTTAATAAGGTTTAAAAATGTTAAAACGGTCAATTATTCTCCTAAAAAAAAAAATTGTT
ATATTTAAAGTTAAATCCATATTAGATATAATAAGTTTAGGGCGATACCCTATTGGTTTC
CCCATCTATAAAAGAAGTATAGATATAAATAATAACAATTTATATATAGCATTATGTAAA
GTGAGTAAAAATAATAACGTAAAGATTCCTATAATCGATGATGAATTAGAAACATCTGTA
TTTTTTACGCCATTGACCTCAATAGATATAAAATTAATGAGAAAAATAGGCGGATATAAA
ATAAAGGAGTTGGGTGTGTTAGAATGGGAAAACAGTATTACGTTTCATAACGACGATTTT
ATGAAAACTATTGATCATATAAAAGAAATATTAAGCGATGATATCGTAGACTATATAGTA
AATAATAACGAATTACTAGAGCATTATGAAAATAATACTACATTAAATAATTATTTATTA
TTATTTAAAGCATTTTCTATTAGTTACTGTAGATTCAATATTCATTCTTTAATACAAAAA
ATTGATAGTCATTTTTTAGGAGACTATGTGTTAATGTATAATTATAAAGAAATATATATA
TCGGACGATTTTAAAAAAGATAAAAATGCATTATATGAAATGATATATGATTTATAA
>LSDV_01_00027 group_209
ATGTGGTCCTTATTTTTTTCAAAACCTCCATCTGGAGCAGGATGTAGACTCGTTGAAACA
TTGCCTAAAACCTCAGGGATAACAACACAACATATGCTCACACATGAGTGCTTTGATGAA
ATTATTTCACAAGCTAAAAAAAATATAAATATAGCATCATTTTGTTGTAATCTTCGCACT
AGCGAACATGGAAAAAATATTTTAAATAAATTAAAAGAAGCCGCAGCATCTGGAATTAAA
GTTACAATTTTAGTAGATCATCAAAGCGGTAACAAGGATGAAGAGGAATTAATATCAAAT
AAAATTGAGTATGTCAAAGTAAAAATAGGAGGAGATAACGATCCGGGAGTATTATTGGGA
AGTTTTTGGGTTTCTGATTATAATAAATGCTATATAGGCAACGCCTCTTTAACTGGTGGA
TCCATTTCCAATATAAAGACTTTAGGTATATATTCTACTTACGCGCCATTAGCTTTAGAT
TTACAAAGAAGGTTTGAAACATTTAAAGCTTTAAATAACAACAGCAGTATTTTTAGAGGA
ATATATATAACATGTTGTTTGCCAGTGAGTACAAAATACCATATTAATAATCCAATTGGT
GGTGTGTTTTTGTCAGATTCTCCTGATCATATATTAGGATATTCTAGAACTTTAGATGCG
GATGTTGTGTTGAGCAAAATTAATTCAGCGAAAAAGTCAATAGATTTGGAATTGTTATCG
TTGGTTCCCGTTATAAGAGAAGATTTAAAAACAACATTTTGGCCAGATATTTACAATGCT
ATCATTGGCGCAACTATCAATCGAGGTGTAAAAGTTAGGCTTTTGGTCGGATCTTGGGAT
AAAAATGACGTTTATGTTATGTCATCGGTTAAAAGTTTGCAATCTTTGTGTAGCAACAAT
GATTTAACTGTTAAAATTTTTCATGATACTAATAATACCAAGTTGATGATTATTGATAAC
GAATTTGCACATATAACTCCTGCAAATTTTGATGGAACACATTATTTACACTATGCGTTT
GTTAGTTTTAATACCAATGATAATGAATTAGTTAAGATGTTATCAAATATATTTGAAAGA
GATTGGTCAGACAAAAAAAATACAGTGCTGTAA
>LSDV_01_00028 group_24
ATGTTTGAATTTTTTTTGGTCTTTATTGCTGCTATTTTTGCATTAGTGGGTATAGTGTCA
ATAATTTTAGATTTAGTTATTATGTTTAAAGAAATAAATAAAGATAATAACTACAAACAT
ATTGAAGAAAAACAAACATTGTTATAA
>LSDV_01_00029 group_145
ATGAATGTTAACAAGTTAGATGAAATAATTAAATTAAGTCCATTTAGAAATATGGACAAA
ATAAAAATCAATAAAGATGATAATTGCTTACTTGGAAATAGATGCTTTGTCAAGATGGAT
ACAGTAAAACACTTTCCTACAAATTCCGTAGCTACATCAGATTCTGTTATTATAAAAGGT
TATAGGTTTACATTAACTGAGTTACTATATTCACCATTTCATTTTCAACAACCTCAATGT
CAATACTTATTACCAAGTTTTGTTTTAAAATGTATTGACGAAGCGACAAAAAACAAAACA
ATATGTAAATATTGTATCATTAATAAAGATGATAATAGTTTAAATATAAATATATTTGTA
CCAACTTTTGACTTAAAATATTTAATAATTGGGTTAAGAATAAAACATTTTTGGTCTTCA
AAGTTTCAAATAGAATAA
>LSDV_01_00030 group_90
ATGTTAAAAAATGCAGCTATAGTAACAAGTTTAATATCGTTATTTGATACATCTATAGAT
TATCAAATAAAAATATGTAGAAACTATTGCAATTTAATACATTCATCTATAAAAATTGAG
TTGTTTAAATGTGCGAGTGATAGTTGGTTAAAAGATAGTGCGATTATGGTAGCGAGCGAA
CCATATAAAAAAAATTTTGATAGTTTTATGGCTGAAATAAAAAAAACTACAAAATCTTCT
ATTTGGAAGGATGCCAACAATGTCCAATTTAACATATTAGAGTATCCAGTAGATAAAGAA
TTCATAAACAAGTTTTTAGAGTTTTCAACATCTGTTTACGAATCACTATATTATGTACAT
TCATTACTTTATTCAAGTATGACTTCGGAAAATAAAAGTATTGAAAATGAATATCAAAAA
AAATTAGTTAAAATTTTATTATGA
>LSDV 01 00089 group 203
ATGAACAACACGGTAATTAATTCATTAATTGGAAACGATGATAATATTAAACGTCACAAT
GTTTTTGGTGTGGATATACAAAATCCAACATTATATATGCCACAGTATATAACCCTCAAC
GGCGTTTCGTCAACGGGGTCATGTACACAAAATGTTGTTTCGACGTTTGAGATTAGAGAT
CAGTACATCACAGCATTGAGTCATTTAATGTTAAGTATAGATCTTCCGGAAGTAAAAGGA
ATTGGCAAATTTGGTTATGTTACTTATGTTGGTTATAAGTCGATACAACATGTTTCTATC
TCATGCAATAATGGTACAATTTGGGAATCGTCAGGAGAAGACTTGTTTTATTCCTGTAAA
AATAACGAAACGGCTCTCAACAATTCTGGATTTTGTCATGAACTTAATAGCATCTCTACT
GGTTTAACAAATAACGATACGATTAAGGAGTCTGCTACGATATATGTATATATAAAAACC
CCATTCGATATGGAAAAAACATTTAGTAGTTTAAAATTATCAGATTCAAAAGTTATTGTT
ACTATTACATTTAATCCTGTATCTGATATAATAATTAGGGATTCTACATTTGATTATGAA
TCTTTTGTTAAAGATTTTATTTACGTAACCGAACTCAGCTTTATCGGGTACATGGTTAAA
AATATTCAAACAAAAGAATCGTATATAGAAATACCTCGTAGAGTATTAGGACAAATAAAC
CAATCAACTGCTGTAATTTCAGAAATAAACTCAGTTACATCTTTTTCGGTTTATGTAAAA
CCGTATTACGGAAATACTGATAATAAATTTATAGCTTATCCAGGCTACACACAAAGTGAA
CGAGACTTCATTTGTGTTTTTGTCGAAAGATTATTGGAAGACTTAGTGGTTGTAAGCAAG
GAACCACCTAACTATTTTCCAGAAAGTGCGGAGTTTATCGAGGTGCCTCCAAACGGTATT
GTAAATATTCAAGATGTTGATGTTTTTATTAAAATTGATAACGTGCCTTGTGGAATGAGT
ATCTATTATCATTCAAATATCTTAGTTTTTGGAACACGAAAAAACTCTATCACGTATAAT
ATTTCAAAAAAGTTTTCTACAATAACTGGAAGTTATAGCGAATCCACAAAAAGAATTATG
TTTTCTCATATATCACACTCTATTAGTATTACAGATGTTTCTATTCCGGTTAATTTATGG
TCGTGTCAACGAAACGTTTATAATGGAGATAATAGATCAGAATCGTCAAAAAATAAAGAT
TTATTTATTAACGATCCTTTTATAAGAGGAATAGATTTTAAAAATAAAACTGATATTATT
TCTAGGATGGAAGTTAGATTTGGAAATGATGTACTTTATTCTGAAACTAATCCTATTTCA
AAAATTTATAACGATCTACTATCAAACTGTGTATTGGGTACAAGGACTTTGAAGTTTAAC
TTTACACCACATACTTTTTTTAAACCAACTACTATTGTTTCTAATACAGCCAGAGGAAAG
GATAAACTCTCAGTTAGAGTTATTTTTTCATCATTTGATCCAAATAATCCAATTTATTAT
GTATCAAAACAAATTGTGTTAGTTTGTAATGACCTATACAAAGTAACTAATGAAAATGGT
ATAAATGTTATCAAAATAACGGAAGAATAA
>LSDV 01 00090 VLTF2
ATGGCAAAAAGAGTTACGTTGCCAGATATTACTATTTCTTCTCCTAAATCAGTTGCCAAA
TCAACAAAAAATGAATGTTTTTCGTCGATACTTCCAAAATATTACGTACCCGTAGCGGAT
ATATCAATAGATACAAATTCAGATAATAGTTATTGTTGGTTTTGTTCCCAAATTTTAATT
ATAAAACCATTATGTATAGAAACCATAAAAGGAAATAATGTTGGGGCTTTTTGTTCCACT
ATATGCAGAGATTCTTTTGCATCAATGGTGAAATCTCATATAGCGTTGAGGGAGGAACCT
AAGGTAACATTATTGCCTCTAGTTTTTTATGATAATCCCAAAGAAGTAATTAATATAATT
AACACATTACGTAATAAAGAGGGTGTTTATGGGAGTTGTTTTTATAAAGAAAAAGATAAA
TCCATACATGTTTCTCTACGTAGTTTACTTTAA
>LSDV 01 00091 VLTF3
ATGAATTTAAATTTAAAAATGTGCAGTGGATGCTTTCATAATGGAATAGTTTCAGAACAA
GGATATGAGTTTTGTATTTTTTGCGAATCCGTTTTTCAAAAATATTCGAAGAGTGTTCAA
AAAAAATCTAATTTTCATGTTTCTAATAAACTTATACATTTAAGAAATGTTCTTAGACGA
CTTTTATCTAATCAATGTTCGGGGGAAATTATTTCAGAGTTATTAGATTTAATGAAAAAA
AATCAAATTTCTTCTGAAGATGTTGATGCTAATTTTGTGTCTAGTTTTTTAAAAGCACAC
GAACGAATTAATAAAAAGGACTATAAACTTGTTTTTGAAATAATAAATCAAGTAAAATCT
GAAAAGTTAAATTTAACAACGGAAAAAATTAATGAAGTTGTTGAGATATTTAAACATTTA
GTATTTTTTTGCCAAGAAAATACACCATCTAAGACGATAAATTATTCCTTTTTTTTAGAT
AAAATTTTTGACATAACACTTGTTACAAAAAAATTTAAAACCGCAAACAGTTAAAAATTAT
ACAAAAAATAACAGTAATCAATTAATATGGGAAAATTTCTTAATATATATGAAAAATAAA
AAAAAGACTGCGAATATAATAGACTTTGGTCACGAGTATGTTTTTACTGATGAAAAATTT
ACAACTTGTTCTTTAGATATATATAA
>LSDV 01 00092 group 201
ATGAGTTGGTATTTAAAGTATAAAGTTGATTTGGATAAACCACAGAAATGCTCTAAATGT
CTATCAAATTTATTTGAGTTTATTACAGAGGATGAAAAAACAATAAAAATGGTATTAGAA
TCTCAACCAAATAAGTTATCAATATTAAAAAATTTTTAACTTCTTGTAGAAATAAAGAT
TTCATATATAAAATACTAGATAAAGAACTACAAAGAGTCTTAAAATGA
>LSDV 01 00093 group 207
ATGGAATCTAGTAAAGATATATTTCTAACTTCAAAAGTTAACTTACATACAGAATATAAA
AATCAAACTCTATCATTGGTGGATAACCATGTTCATACTCCAATTACATCACTATCTTGT
TCAATTTGTAATTCACTATCAAATTTTGCAACATCCGACGATATTATCTCAGCAGGGGCT
AGACAAAGATCTTTACCAATGAAAAGGAGAATTCTTAGTAAAGATAAAACAAACGAAACG
TCATCGCCTTGTCCTAACAACAAAAAAGAAGATACCGATACAGCAACTGTGCCAATCGAT
GAAGTCGCATCAACTAATGATTGGCAAGTTAAATTAAGAAAAGATGGTAATATGATAGCG
AGGTATTTGACGGATAACAAATGCAATGTTTCTAATTTTACAATTCAAGACATGCTCACA
ATAATGCGAAAATTAAATATAATGAGATCTAATAGAAATGAGTTATTTGAACTTTTAGCA
CATGTTAAAAGTTCGTTAACTAACTCCAGCATGTCTGTTAAGAGCACTCATCCCTTAGTT
TTAATTCATTCGCATGCACATCCTAAAATAGGTGAACAACTAAAAGAATTAGAAAAATTA
TACTCTCCAACTAAGCAAGAAATACTTTTATCAACAACTCGATTTCAATCTATCCACTTT
GTTGATATGTCTAGCTCATCAGATTTAGCATTTCATTATAGAGATAATGAATCAACATAT
TACATTCATCCTATTTTTATGTCGCTTTTTGGTGTAAAACTACCGGCGTTAGAAAATACA
TTTGTATATGGGGATAGTTATAGTTTACTTCAACAACTTTACGATTACAAAAAAGTTAAT
CCGGATAATTATATGCTTTTAGTTAATAGACTTACTGAGGAAGCACCAATTGTATTTACT
>LSDV 01 00155 group 195
ATGTCTTCCGGCAACTATGTCTACCGAAACAACTTTTCAGATGATGATGATATCACAACC
GCCATCTCTGATTATTTGTTTTGGTCATCACTGGCATTTTCCTCCAGGGAGGTTGCTGGA
AAGGTGTTCTTAGTTTTTGAATCTTTCAAAAAGGATGCCTCACTTGTATTTGGAAATGAT
CTAACAGCTTTTGTCAAAAACATGTTTTTGGATTCTAAAATTGGATTTGAACAATCAAAA
ATTATGATTAATTCCATGTTAAAAAAAGAAAATTACATTAGGGAATCATGTGCAGTGATT
GGCATTTTAGCAAGAGCAGCAGAATATTGGGGTGGTGAATCATCTCCAACTTGTTCTTCT
GTGAAAGTGTTGGTATTGCTTCGAGACCTCGTTTCTGACAACGATATTTCGCTAGTGAAA
TCAGCACTAATAATTAGACTTAAAAGATTGAATGAAAAAAGTATCCATTTACAGGTTTAA
>LSDV 02 00018 group 15
ATGGATATAAATATAATAGATGAAAGTGTTTATGATAATGAACTATTGTATGAAAACAAT
TGTAATGAAATTGATGAAAACAAAACATTAAATGAAAGTATTCAACAACCTAGTAACTTA
AGTAAATTAAGCAACATAATAGATGAAAATTTTAAGTTTTGTACTTCAATTGCTATTGGT
AATAATATTTACTTTTTAGGTGGTGTGGATAAATACTTACGATCAGTAAATAGTGTATTC
GCAATCAATGCAAAAACTTTTGAAAAAGAAAACTTACCTTCATTAATTTATCCAAGAAAA
TGCCCAGGTGTCACTTATTTTAATAATAGAATATATGTTATTGGTGGAATCTATAATAAT
TGTATAGTTAATAAAGTTGAAAGTTGGTCATTTGGAGAAAGTGTTTGGAGAGAAGAACCA
AATTTACTATATCCTAGATACAATCCATGTGTTGTTAATGTCAATGATACCATCTATGTT
ATTGGAGGAATATCTGAATATGATAAATCAGTAGAAGTGTATAATTTGAAATATAATAAA
TGGTCATTGGGTGAGTGTACAAAATATTCTCATTATGGTGGATGTGCTATATACCATCAT
GGTTTAATATATGTTGTTGGTGGAATTTCATATATCAATAATATAAAAGTTTTTAACATG
GTCGAAAGCTTTAATCCTATGTCTTGTAAATGGAGAATTGAGTCGTGCCTGAACCAGCCA
AGATTTAATGCATCAATATGTATTTTTGATGATTGTATTATGATCGTTGGAGGTTTTCAT
TATGAAAGATATATACGTGAAATAGAAATATTTGACGATAAAACAAAAGGATGGAACGTT
ATAGGCGCATTAGACATAGATAGTGTTTTTTAA
>LSDV 02 00019 group 18
ATGACTTTAAAAAGGTATATCAATAAAGAATATGTAGAAGAATTAAAGTACATGTTATTA
AAAAATGATTATGATAGAATTTTAATATTTACAATTGGAGGTATATCACAAACAAGGAAA
GATATTTTTGAGATATCATCTAATTACTTTAAAAAAGCTATTAAAAAATACAGTAACGAA
GTTAAGTTACCTTTTAAATATAAGCCATTTACGTATGTGTTAGAGTATATTAATACTGGT
TATATTACATTGAATAGCAAAAATGTTGTTGACATTTTTGCTATTTCTAACGTAATCGAA
ATTAAGTTTATAATGGATGCATGTACTGATTTTATGATAAATTACATTGATGATAGTAAT
TGTGTTGATATCTTTAGAAGATCTTATATGTATGGTTGTTACGATGTGTACGATGTTGCA
GATAAATATATTAAAAAAAGGTTTATGTATATAAGCAACGATTTAACTAAATTAAGAGTT
AATGAATTAAGGCCCATATTAAAAAGTAGTGATTTAGTTGTCGATACCGAAGATTTTGTT
CTTAATTTTATAATTAAGTGGGGAACTGTAAAAAAAACTAATAGGCTTAATACGATGAAG
TTAGTAAATGACTCTGTAAGATTTACCTTTTTAACAGAAAAAGGAAAAAAAAGGTTACGA
GCGTGGTCGTCCAGATTTAAAAAAAAAAACCTTATGATTTTAACAAAAATAGTGATAAAC
ACATACCACGATGTAATTATAAAAATAAAGAATTTATTAATAGTGACAAATTTACAAAGT
CTTTTAAGTTTAAGGTTTATAATGATAACTTAA
>LSDV 02 00027 group 11
ATGTCTATTATGTGTTTAAGTAATAAAAAAAAAGAAGAATATATTTTGAAGTACTTTTTA
AATAATAGATGTTATGATGATTTATATTCAGTTAATGGTGGGAATGTTGCAGTTGGTAAT
TTTTTAAAAAGTAAAAGTGAAGAGTTATATTTTTCTTTTAATCTTTTTTTAAATGACTTA
GATATAAGTAAAAAAAAAGTAGGATTTTATTCATTGAAAGAAATAAATGTAAATATTTTT
TATAAACTTTTACTCAATGAAGAGTTTTTTACAGTTATAGGATCGTATTTGGTTAAATTA
AAACACGGAAATGCATTTTTTTAA
>LSDV 02 00028 group 86
ATGTTTAAAAAACAAAACAATGAATTTAATATTATTAATGCATTAGAAACATATAAACAC
TCTATTATATTAGCGAGATTAGGAAGCGGAAAGGGAGTTATAATATACCTAAATAATTTA
GATTTAGTACGATCTGTAACAAAACTATCTCATTTAGAAATAGTTGGAATTACAAAGTAT
GCAGAACCATATTTTATTGACATGCATATGCACAAGTTATTCATTGACGAAGTCGACACT
GAAACATTTTTTTCACCTAAAACATCTATATCTCCTTTGGTAGATATTGTCAAAAGAAGA
GCTTTAGAAAACGATAAAATAAATAAATCTGTTCATAAATTGCATGTTTTTGAAAATAAA
AATATTTCATTGAGTGAGATAAATTATTGGATGTGTGATAATGGATTATCTAATTATAGA
TTTGTTAATTATAAAAATAGTAAAAAAGGTAATGATTATACATTAATTGATGAAATGATA
ATAGGGTGCATTGGAAAACATTATATATGGGTTAAACAAAAAAAAAAATATAATAGACCG
GAACTAGACATATTACCTTATAATGTTGAAAGTATCGCATCGAAAAAGTTATGGTCAAAA
AATAACATATATGATGAAGTAGTTACAATAATACCTATAATTATAAACTCAATAATAACT
CCATTAGGTCCATCAATTTATATGATGACAAGTTTTGGTAAATCATTTATAAATTTTAAC
TCAAAAGAATTAATATTATGTTTTTTAAAATGGTTAAAGGAAACAATACTAATAAATATT
AAAACAGCATTTTTAGTAGGGTTTTTAAGCAATGTTTTTGACTTTTTACTGCTTAAAGCG
TCGTGGCCAAAAAACATAGGATGGAATTTTATAGGAAATGATTATATTGTATCAGATGAC
GGATTCAAACTAATATTAGTAGATATCGCTAACTTTTCGTGTAAAATGGAAATAAGTGAT
TATTTTAAACAGTGGAATGATAAAAAAAAAGATAATTATACCAAAAGATTTTATCACGGAA
GAAGAAGCAAAATGTAAAATAAAAGCATTAGAAAAAATGGCATTAGAGTCAGTTTTGGTA
TTATATGATTCCATCAACAAACAATATGATGTTTTAAATAATTTAATATATCCTTTTAAT
ATGTTTTCTTTTAATAGAATAGAAGATATTTTTTTAACTAAAATAATACAAACATCACTA
TCTAAAGTAAACAATTTTATGTATTATCCACTAGAACACGACACTGTACGATTTATTATG
CAGTTAATAAGGTTTAAAAATGTTAAAACGGTCAATTATTCTCCTAAAAAAAAAAATTGTT
ATATTTAAAGTTAAATCCATATTAGATATAATAAGTTTAGGGCGATACCCTATTGGTTTC
CCCATCTATAAAAGAAGTATAGATATAAATAATAACAATTTATATATAGCATTATGTAAA
GTGAGTAAAAATAATAACGTAAAGATTCCTATAATCGATGATGAATTAGAAACATCTGTA
TTTTTTACGCCATTGACCTCAATAGATATAAAATTAATGAGAAAAATAGGCGGATATAAA
ATAAAGGAGTTGGGTGTGTTAGAATGGGAAAACAGTATTACGTTTCATAACGACGATTTT
ATGAAAACTATTGATCATATAAAAGAAATATTAAGCGATGATATCGTAGACTATATAGTA
AATAATAACGAATTACTAGAGCATTATGAAAATAATACTACATTAAATAATTATTTATTA
TTATTTAAAGCATTTTCTATTAGTTACTGTAGATTCAATATTCATTCTTTAATACAAAAA

CTAAAAGAGGTAGGATATATAGATGAAAAAGTTATATCTTCAAAAAGATGGGAGTGTATA
TCTCATGATAATAAAATTGATATATTGGTATTTTACCAGAAAAAACAACTATCTGTTTCA
AATGATCATTTATATAACTTTTTAATGAGAAAAAGAAATAAATCCTTAACGATATATTAT
GTTAAAGATAATTTATTGTTTGATGGGATAACCCCATCATTTAGACATATTAATCTTGAT
AGCAAAATTAATACAAGAAAAAAGCTCAAAGATTTGTTTGTTTTAATTAATATGAATACA
TGTAGTGATGATATCATAAAGAAATTTATTTACGATAACTTTGAAACTTTTAAAATATTA
ATGGATGCAATTAATTGTGATTCGTTATGGTTAAGGAAAGCATTGCATCCAAGAAAAAGT
AAAAAAAATATAAAGGAAAAAAATTATTATTATAATAAATTTGTAAATAAAATAAAAGAT
TTAGATAACATCCATTATGTAAACGATATATGCACATCCTTTAAAAATATAACAGTGTAG
>LSDV_01_00031 group_117
ATGGCAGCATATAATTCACAATATTATGCAAACACACCATTTTATATAACGACAAAAGAA
GGAAAATATCTTGTATTAAAAGCAATTAAAGTTTGCGATATAAGAACAGTTGAATGTGAT
GGCGATAAAGCATCATGTATTTTAAAAGTGGAAAAATCAAATCAAACTTGTGACAGGCCA
TCTTCACCATGTGAACGAGCTGCAAGAGCTTCATCTCCAAATAGAGGGAATAACTCTGTA
CCATTTATGCGCACAAATATGTTAGAGGACCTTCAAGCGAAAAATCGTAGCGTTATGTCT
AGAATACTTGGTTAA
>LSDV_01_00032 PAPL
ATGAATGATAAAATTGTTGTAGATGTTTTAAAATTGTATTTAAAAAGAGATCCATCTTTA
ACCGAATTTCACATGTTGAAGTCACAATATAAAAACATACAAAGAGTAAACATTTTTAAT
AAAGAAATATTTATATCTTTGATAAAAAAGAATAAAAAGAAATTTTTTTCTGATATCAAG
TCTTCTCCATCTGATATAAAAAGATCAATTCTTACTTACTTTTCTAAACAAGAGAACACA
TACTCAATTGGTAAGTTATATACCATAATAGAATTACAAAGTATATTAGTTACAACATAT
ACTGATGTATTAGGGATATTGACAACTAAAGGGCCTGATTTATTTACTTCTAATATTTAC
TATAATACATCATCGATGCAATCTTTAGCTAGGGATGCTTTAAATTTCATGAACGTTGCA
CAGATGACGGATAAAACCATGGGTAGACATAATGTTTCGTCGTTAGTTGATAATGTAAAC
TCTTTAATGGAAGAGTATTTGCGACGACATAATAAAAATTGTATCTGTTATGGATCATAT
TCTCTACATTTATTAAATCCGGATGTTAAATATGGAGATATAGATATATTACAAACAAAC
TCTAGAACTTTTCTTATAGATCTTGCGTTTTTAATCAAGTTTATTACTGGCTTAAATGTT
GTTTTATTAAAAGTACCATACTTAAAAAACTATATGGTATTAAAGGATCAAAACGATAGC
CATATAATAGATAGTTTTAATATACGACAAGATACCATGCAATCAATACCAAAAGTTTTA
ATTGATAATATATATATATAGTAGATCCAGCATTGCAACTTATGTCAATGTTAAAAATGTTT
TCACAAATAGACAGATTAGAAGATTTAGCTAAAAATCCTGAAAAACTAACAGTTAGATTA
GCTACATTATTAGAGTATGTTAGAGTTAACTATGGTATAAACTTTATCAGTAACCAAACA
AAAAAAAATAACATGCCTATGTATTCAGTTATAGACATTGATAAACGTATCATCACGGTC
GATACTAGCAATTACGATTTCCCATTCAAAAAATGTTTAGTTTATTTAGATGAAAGTTCA
TTATCTAGTGATATATTAGATTTAAATGCTGACGATGCTATCGATTTTGAAAATGTTTCA
AATTCTGCATTTTTGATAAATAATAATATTTTATATACTTATTTTTCAAATACAATACTA
ATGAAATCAAAAACCGATATTCATGAGATAAGTTCCAGAGGATTAAGCGCACATATATTA
ATTTATCAAATTTTAACTAAAGGAGATATAATAAAACCACTTACAGATATCGTAAACTCA
TTAATTGGGGTGGAAAAAAACCCAATTTATGATATTATACCTAGAGATAAGAAAAGTGGT
AAACATGGTATAATTGATATAGAAAAAGATATTATAACGCATTAA
>LSDV_01_00033 group_119
ATGTTTAATTTTTTAATTCCGTTAAAGGAAGCATTTATTTTATATAATTGTGATAATTTA
ACAGAATCATATAAAAAAGTAAATATAGATGATGCTATGAAAATGCTCATTAATGGAATT
AACCCCAATAATTTGCCACAAAAATGGTATTTTAGGATAAGAAAAGAATTTCCTAGCAAA
ATATATTTATTCAAACCAAAAACTGTACCATTTGTCGATTTGATAGCTTTAGTTTATAAT
CAAAAAAATATTGAGTTATATAGAGAACATATAAATTTCCATAAAAAAGATATTATTAAA
AAATGTAGTAAAAGAATAATACTAAAATGTATTCAGTATATGGTTATTGCAGATGATGAT
ATTTTTTGTCTAATACACAGATTTTCTAACATTGAAGTAGAAAATATACTAACTAAAATA
AATTCGGAAAGTATTAAAAATATAAACTATTTGTTTAGTGAATCATTGGCAGAAAAAATT
TTTATTAATGACTTTAGTTTATGTGATAGTTTATATCAACATCAATCCTTTGAAAGTAAA
TTTTTAATGGATATGTTATATAAATATGGAATAATACCAAACAATGATGGAATTTTAAGT
GCGATTTCTATTGAACTTATCTTGGATGTTTTACAATCTATAAAATTTCATAAAGATATT
TTAACATTTTTAGATATGTTAACGTTTGAACAATTAAAAGATGACAGAATAAAAAAATTT
ATCATAACTAAAATAAAAAAATGGAGAAGTTGATCAATATAAAACCTATGCTATAGAATTT
TTATACGATAAAAAAGATGAATTAGGATTATATTATAGTTTTTTTTTAAAGATCCATTA
ATAGATTTTACAAAATATGAATTAACAAAAGATTTTTTAAAAATATGCTGTAAATATATT
GATAATTATGATTATAACAATAAAATGTATATTATCAATTACTTAATAGAAAAAAATTAT
ATCAATTTGCTTTCTACTATTATAGATAAAATACCATTAAATATATTAACAGAAGAATTA
TGCATAAAAATAGTTTGTGAATCAAATATTAAAGTTAAAATCAAAGATATTCCAATACAT
ACTTCGTTAGTTATGATAGTGTGTATTGAAATGGGATATGAAGATTTAGTTGAGCTACTA
GATACAATTGATATAAATGTATTATTAGAAAAAAACGCAAACTTAGTTTCGGATTACACA
TTTAGTACTGATTGGTATAATAAAAATAATAAGTTAATAGATATTTTTGTCCAAAAATAT
GGATTTTGCTCTCATAAAATGAACAAGTTAATGTTTGAATATCCGTTAAATAAAACATCT
ACGATGTATTTACTAAATATATTTTCATCTAATAATATAAATAAATATTTTAGCTATAAA
ATTCAACCGTCGTTATTTTATCTATCCTGCACACACAGCGAAAGTAATGTTAATTTTATT
CTTAAAAAGGTTAGTGATATAAAAAACTTAATACCATTTAAAAAATTGTCAAATAAAAAT
AGTATGTTTATATATGATCAAAATAAAATAAAATCATTAATATTATATAGTAATATACCA
GAAATCGATATGACCGTTTTTAAAAATATATCTTATATTGAAGATAGTACTGGAATAAAA
ATACAAATAAATAAAGAAAATGATATTATTTTAAAAACAGAAGAAGATATTTTAAATCAA
GTATTTACAATTGCTAAGTTAACCACATATGGTTTGGTATATTTACCATATAGATATTTT
CCTTCCTGGGTTCCTATAATTGATATAATAAAAGGATCTAAATATTCACACCCTTCAAAG
ATAGAACACGGTGTTATTTTTAAGATATCGCCTGTAGATTTTATCGAATATAAATTTTTA
GGCAATTATATAACTAATATTTATTCACTAAATGAACCCATATCCAATTTCCACTCTATA
ATAAATTCATTTATTCAAATTCTTTTTTTATACTTAGTAGTAGGATCAACGTATTCTAGT
AGAGGAACTAATGTTTTTATATCTTTACTTATAAGTAATTTCTTTACAGGAATGAAAATA
AATGAAACACTTACTGAAGACATATCTAGCGTTTGTAAAGAATTAAATCTTTTTAAAAAA
TATATATGTGATAGTGGATTTGTAATAATAAAAAAAAATTATTTAAAAAATACACTACTT
GGCATTTCAGATGTTATATCTACCGAAATACAAAGAGCTAACATTCATACAATGATAAAA
AAGCTTATAATGAGAATGAGAATGGGAATTTTTTACTGCAGTGATGAAGAGTCAATAGAT
CCATTCTTAATGAAGATAATTCACACAAACTGTTCAGAAGTTATGTCGGACGAAGAACAA
ATAATAGCATCTATATTATCAATAGTAGGATTTAAACCAACACTTGTTTCGATTCCTAAA
CCTGGATTTATGAATAATTATGAAATGCAGTTGCAATCTGTGCCATATATCGTTATCAAT
CCAATGAAAATGATAACTACTTCAAATTGTCCAATTTCTATCAATACGGGTAACATAAAT
TCACTTACTTTTGATGGAAATACTGGCAGGGTAGTTTTTGCACCACCTAATATAGGATAT
GGTTATAGTAATCCTTTAGAAAATCATTTTAATGCTTTTGGTAATAATTTCCCAACAATA
TCGAATATCAGTTCGCCAGTTATTGTTAATGGTGTTTTAATTTTTTACGTTGAACGTAGA
CAGCACAGAAATACATTTGGTGGGGAGTGTTACACTGGATACAGATCAATCATAAATGAT
TCTCCAATAGAATTATCTCAAGAAGTTGTAATTAACGGCATTATGTACAAATTAAAATCT
GCTGTATGTTATAAAGTAGGCGATAACTTTTTTGAATGCCATACTAATAACACGGATATA
TTTTTAAAGGGGCATTACTCAATTATATTTACTGAATTAGGACCTTGGATGTATGACCCA
TTATCATTTTTTAATAAATCATCGAGAGATTCTAGAATGATGAGATCTTTAAAAAATCAA
TATAGAAAGACGTCAAATAATAATCAACTTGATGAATCTGATTTTTATGAGTGGTTAAAA
GGTGAAGGCGCTTCGTTTTTCCTATCAAAACAACAAATGCTTATGAATCATATAACGATG
TTCGATGATGATTTGTTAAAAATGGAAGAAGCAATGTCATTAATATCTAGACAATGTTGT
ATGTTAATTTATTCACAAGATTATGATTCTTATATAACTGCTAAAAATATTACTGAATTA
TTTTAA
>LSDV 01 00094 group 130
ATGGACTTCATGAAAAAATATACTAAAGATTTAGAAACAACGGTAAAGAATAAAAAAGAT
GAGGAAATAGCATCTACTTCAAATTTAATTAACAACACATCTGTTACATTAACTGATGTA
GATACTATGTTAAAAAGTAAAGAACATTTATATCAACAAATGATGATGAATCAATTGGAA
GAAAAAAAAACATTAAAAATCAAAAATATAGAAATCAAAAACAACAGTAATAAACTCAAC
GATCAATGTAGTGAAAAAAAACAAAATGATCCGTTAAAAAAAATAAAATCTATTAGCCAT
GATGAACTAGTAAAGGAACTGAAAGATATAAAAGATAAAACTAAATCACTTCAAGATGAT
TCTGATTCACTTATTAAAGATATTTCAGTTGCTAAAGATACAACTTTTGATGCTATAAAC
TCAATTATGAATGACTTAAAAAAGAGATTTAATATAGACAAACTGGATGATAATAACAGC
AAATAA
>LSDV 01 00095 group 115
ATGGCCGAATCTGATGATATTATCGACGATTATATTTCTGACGAAGATGCGAGCGATGAA
TTTGAAGAAGAAGAAGAAGAAGAAGAATCGTTAGAAACAAGTGATGTTCCGTCTTTGAAG
CAATCTAATTATAAAGTTGGCTCGTCCATATCTATTAATATAGAGGATACCAATCAACAA
AATATAACTAACGCAAGCCTAATAAGTTCTAAAATATCAGCTATTAAAAAAAGATACACA
AGAAGAGTAAGTTTATTTGAAATTACTGGTATTATAGCTGAAAGTTATAATCTTTTACAA
CGTGGAAGAATGCCACTTGTATTAAATTTATCAGATGATACTATGAAACAAAAGATTTTA
CATATAGTTATTGAAGAAATTAAAGAGGGTACTTGTCCTATAATAATTGAAAAGAATGGA
GAATTGTTATCCGTAACGGATTTTGATAAAAAAGGACTTTATAATCATCTTGATTATATT
ATCAATATTTGGAAAAAACAAAACAGATTTTAA
>LSDV 01 00096 A6L
ATGGATAAACTGAGGACATTATATTATGATTTTTTTAATATTAGTAAAAAATATTTAGAA
AAAGAAACTAATGTGTTAACATCTGATCCAAATTTTGAATCAGATGTTTCTATTCTTATG
AACTTAGTACCTGTTTTAGAAAAGAAAATTTCATCAACTATATCAGTTTCAACAAAAGAT
GAAGATATTATCTTAATGATGAAGTATTGTAACTACAAATTATTTTCTTTTTGGTTTTTA
AAATCTGACGCTGTAGTTAAATCTGTTTATAATAAGTTAGAATTAGAAAACGAAAAAGAA
AAATTTAAAACTATATTTAAGGACATGTTAATTAATGTACAAACACTTATATCAATTAAT
AATATGTATATAAATTTAAAACAGGACACTGCCGAAATAGTATCTGATTCAAAAAAAATT
ATCGATATTATAAATCAAATTAAAAATACTAATTGTGAAAGTAGTGCTTATAAACTTTTG
CAAAATAATCATAGTTTTATTGTTAAGACAATAAATAAAATTCTTTCTGATGAAAATTAC
TTATTAAAAATAATTGCTGTTTTTGATTCAAAACTTGTAACTGATAAAAGTAAATTAACT
GAGTATAGAGAAATATTCACCATTTCAACAGAAAGCATTATTCACGGAATAAAATGCATA
TCAGACTTAGAAATTTCTACAATAGATGTAGATAATAACAAATATATTATGTTTTTTAAG
AAAATATTAGGCACTGTTATATTGTTTCAAAATAATGACTTAAATTCTCAAAAATTCATT
TATATAGTTGCCAAACTATATTCTTTAATAAATCAACAATTTAAAACAAATCCAAACGTG
GGCTACTTATTAACTGATGTGCTAGATTCTATAAAGACGAAAATATCAATAGATGATATA
AAACAAAAGGGGGTAAATAATTTACAATCATTAATTCGGTTTATATCAGACAACAAATCT
TCGTATAAGTCTATATTATCAGAAGAATATATTAAACGGGAAGGAGAAATAATAGAGATA
TTGCAAAATATTTCTAATGAAAGTAAAATAGAGTACTGTGGAGAAATAATTGATATACGA
AAGTTAATAAAAATCACAAAGGATAGATTCTTTAAGAACGATGTTTAA
>LSDV 01 00097 VETFL
ATGAAATATATTGTAAGTCCGCAATTAGTGCTATATGTTGGCAAATCACAAGAAATAAAA
AGAGCTTTATATTTAACACCATACGGTGTGTTAGATGATAAATCTCCTATTTATTATTTT
TTAACATCATGTTTAAAAATTACTACGCCCGAGGTATTAACTAGACATATACTATTAACC
TTAAAAATGAGACAGTTAAAAGGATACTTATGTGATTTACTAAACATAGACGATGATATA
ATTATTTATTCTCATAAAAACAATTTAGAATATAGTTATGTTGATAATACTATTTTTAAC
CCTTTCGTTCATACTCAACGAAAAACATTGATAAAATCTGATGGATTTTTATATAATATA
TATCCGGACGCGTGCGATTTTTTAGTCATATGGGTCGCTAACGCAAACGATACATCAATC
CCGGAATTTGGATCCCATGAAGAAGTTGATAATAATATTTTAAAATTTGAATCTAGATTA
CTTGACGTATTTCCTTCCATGGATTTGGATATGACGGTGGAGTCAAAATTCAATAATATT
TTTAGGACAAATTTAAAATTTACAGGATTAAAAAATATTATAAAAAAGGTAAACGAATTA
AAAGAATGCAATTATAAATCTTTATTATACAAATCTGATGAATTTTTATTAACATCACA
GGGAATAAATTTATTTTAACTGATGAAAAAATAAACTTATCAGTTTGGGATACAGATGGC
ACATTAGCATTTTCAAGCGATGGAGATACAATAATGATAAACAATGTCAAACTATTTACT
AGTTTATTATCAGATATTGATACACAAATGGAACGTATAAAGTGCGACGTAACGTATAAA
ATTTTTTTATCTACGCCAATCACGTCTAGAATAAAACTTGATATAGAAACCAGTTTTATA
TTTGTCGAAACAGCTACAAACAACATACTGCTTTCATCAGATAAGAGAATATCAATTATT
TTAGCAAAAAACCACATATCTATAAAAGTTAAAAACTACATTCCCAATATTGAAAAATAT
TTTACATTTTTAGTTATTGCAGTTAATAATATGTTTAATAATATACAACAATCATCTGAT
ATTGATAGTCATTTTTTAGGAGACTATGTGTTAATGTATAATTATAAAGAAATATATATA
TCGGACGATTTTAAAAAAGATAAAAATGCATTATATGAAATGATATATGATTTATAA
>LSDV 03 00001 group 112
ATGTCTTCCGGCAACTATGTCTACCGAAACAACTTTTCAGATGATGATGATATCACAACC
GCCATCTCTGATTATTTGTTTTGGTCATCATTGGCATTTTCCTCCAGGGAGGTTGCTGGA
AAGGTGTTCTTAGTTTTTGAATCTTTCAAAAAGGATGCCTCACTTGTATTTGGAAATGAT
CTAACAGCTTTTGTCAAAAACATGTTTTTGGATTCTAAAATTGGATTTGAACAATCAAAA
ATTATGATTAATTCCATGTTGAAAAAAGAAAATTACATTAGGGAATCATGTGCAGTGATT
GGCATTTTAGCAAGAGCAGCAGAATATTGGGGTGGTGAATCATCTCCAACTTGTTCTTCT
GTGAAAGTGTTGGTATTGCTTCGAAACCTCGTTTCCGACAACGATATTTCGCTAGTGAAA
TCAGCACTAATGATTAGACTTAAAAGATTGAATGAAAAAAGTATCCATTTACAGGTTTAA
>LSDV 03 00135 group 8
ATGATTTGTGACTCAAGGTCTGCATCCTCTATATGTAATGCAAGAGGGTTGGATTCGGCA
AATTATAGAGGTGATGGTAACATATATGAAACAACTGACGACGATTTTGTGAAACGTGAA
AATTCTTTATATGCAAGATCAAAACTAGAACCAGAATTAAAGGATAATCCATTGTATGAA
TCATCGTCCGATAGCGGCATTGTATCAAATCCATATAACAATCCTAAATTGTCGAGAAGA
AATGCTATTAAAAAGAAAGTATTAAACGATGGTTATGAGGAATTCGTTATACGGACCGAC
GAAGAGCCTAATGAAAAACCTAATATGGCAGCAAATACGTATAATAACAATGATAAAGCT
AATAATAAAGATAAAAATAAAGGATTTTCTTACTTAAATAATGATATACAAAAAGATGAA
AAAAATGTTAATAAAATTAAAAAATCAAAAAAAGGAAGAACTAAACGTTTAACTGAATTA
TCCACTGATAATAAAATGAATAATATGATAAAAACAATTGCTATTTCCAGTTATTTATCA
TCAACGAATAGTAGGATATCGTCCATAATGGCACAGGCTAATCACCAACCAAAAGAATTA
ACAATTGTTAACATTGTAACGTCCGTATTATCTCAAATCGGTGGAACTTTAGCAATGGCT
GGCTCTGGGTCTCCCAAAGCTGCAGCTGCAGGTTTAGTTATTCAAGGAATATCTGGATTA
ATAGATGCAGCGACATCAATTTATTTTCTTCTTTCTGGTCAAGAACAACCTAAAGATCCA
GCTATTGAAAAGTTTTCCAACTATGCATCTTACGTTTCAAGGACAGATGCAGGAGCAAGG
GTTTGTATGATGCCAGATTCAGATATTACAATAACGTTAGCATATAGACATAGTAACATG
AATGTAGAAGCTGAAAAAACTAGAGGTGAGTACACAGATATAATTCCAAGTACCGTATAC
TACTTAAAAAACAGTCAAATAAGTTATACGGTGAAAGTAACATTGGTATGTCCAATCGGT
CAGTTGAGGCTTTTGGAAGCAGATATAAATACATATGCCACACTTACTCGGGAAGATAAA
GATGGTGTAAAATTCTATCATGTGTTTGGGATTTTAGAACTTCTATCATATCATCCTAAT
GTTACATTTACTTGTGGAAATGAACCAGGTGTAATCTTCATCCCGTTTGAGCAAAAATTA
AGCGACATGCAATTATTAAGGATATCAACTCCTGGCGAACCTAGAGAAGCCGAAGCAATG
AGTTCTGATGTTTGTGATATTTATCCTCTTAAGAAGTTTTATGTATTAGCTGGAAACTGC
CCTTTTGATATGAGCAGAAAATCAGTAGCGTATGTTACATGTAGTACGTTACTAAGAATG
TCAACGTATGAACATGAAAAACAGAGGTGGATTTTAATGAATCCGTTTTCAAACGGTAAT
GAGGACAATATTCAATTGTTTACGTTTAAAAAATATGACTTTAGTGCAGAAAAAGATAAA
ATAAAACTAAATAGTATTTCACATAGTGATACTATTTGTAGCCAATCTGATACTAGTACA
TGTTTTTGGGCAGATGCTATGATTTTAGAAGATGTAACATCTTGCAATTCTAGGATAAGA
AAATTGTCAGTTGAGATGTCTACAGTATCAGAAAAAGGATATAACAATTTTGTACTTACG
TGTCCATATGGGTCTACACCGTTTTATATAAGTAATGGTTCAATAATAAGTATTCCAATG
AACACAAGAAGGACGTCGGTTAGATTTGCTTCTAAACATAACAATGTTGCACTAATATCA
TGTATACATAATTCAAACCCTGCATATAAATCGGATATCGTTGAAGTTATGTTTAAACAA
TCAAATATGAGTGATATGTATTTGGATTTCAAATATTTTAAGGATAGAAAATATTTATTT
GATTCATTTAGTGATATAATGCCTAAAAGATCGAAAACTTGTAAGAGAGCATCAGAAAAT
AGCAATTGTAAAAACTATTATTATATTAAACATGTTCCTGAAATAGAGTTTAAAGTGTAT
GTTGCAAAACTTCCAATGGTTAGATTAGGCACAAGTTATTCAGGTGTTTTGAATTATCAA
ACATTAGAAAAAGTTAATAAGTATTTTTCAACACCTATAAGTATTTCTGTAGATGCTAGT
ACTCTATCTGATGTATACAAAAACCAAGAACATTTTTGGAAATTTGCAATGGAAAAGAAA
AGAACTTTTAGTTCTATTACGGCTACCATATTTGCGTGCTCTGTTGTAGCTGGGAAGGTA
AACGTAAACATGGGAGTTAAAGGTTCACACGATTATTATGGAAGAAGCGGTAAATATATT
TACATGGGATCAAAAGATTTTGCATCTAATGATAAAATATATTTCAAATTCATACCTGAC
AAAGCAGAGTACCGTTTAAAGGATACATATGGCGAATGTGAAATTTATTTAGATTTAAAA
ACTAGGAGAGTGAATGTAAACTGTCCGGAATTGACTATACCACAACATCCATTTGATTCT
CCAGATGTTAATAGTTTATGTGTGTTAGTTGCCACATCAAGAGATCATTGTGCTATATCT
GAGGAAAATTGGAGAAGTTATGATAGAACTCATGGTGTTGGGTATAGCCACGAATACGTA
GATGCGGAGTTTGATTCTTGTAGGAAGAGCCATGGTCCAACATATCCAGTTGATAACTTT
TGCTTTTATTGGTGTGCTGGGATATATTGGCCACCTGACTATGATCCATGTGCTTCATCA
ATGGTGTTAGGTTATTCTCCGATTTTTCCAGAAAATAGAATTGTGCACCCTCCATATATA
AAGGAATTTGGTTATGAACCAGGAAAAAATGAATACGTGAAAAGAGAGTTATATAACAAA
TTACAAAATCTTTATGAGAAATATAATATGTTGGTTTTATATTCAATGAACCCGGTTGTT
GAAATGTCAAACGGATTAGCAAAATCTATGACGCCAGAAGGCCGTGAAATTTTTAGGTTA
ATAGCAAATAGTAACGAAATGCAAAAAGCTAAAGAAGAAAATGATATGAAAGCTGAGAAA
GTTAAAATGGAAATAGAAGAAACATTAAATAATATTTACGTTGATACATTATCATACTCC
GAATCAACTTCCCTTTTGCGGTCTGCTATTTCCACAAGGTGTTGTGTTTTGGACGGTACT
AGTGTATATAAGTATTTTGATTTAGAATATTATCTTTGTGGTAATTATTCTGACTACTTA
ATTAATATTAATAATGTAACATATGTAAAAATTAATGATAGTCTAATAGAAGAAGATATC
TATTTGGTAAGAAATATTCCACAGGTAACATGTTTTCATATAACGTTGGTGGCTGTAAAT
AACGAAGAACAACAGAAAAAGTTTGAAACAGAAATTGTGACAATGGCATTTGAGGATGTT
TTAACTGAAATATTTGACGAGTATGATGATAGAATGGTTAGTTATTTTGACAAATATATA
AGCGATGATAATAATAATAAAAATAGCAAATTTACCACTAATAACACAATTGCTATCATA
TTATCATTTGTTTTTACAGCAATAATTATTATAATTTCTACTAAATTAATAATGCGCTCT
AGAAAAGGCAAATATACCATACATAATAATTTTGTTATCTTTAAAAATAATAATAGCAGT
AGTGAAGATGATAATAATACAGTTTCATTATCTGATTTAGATTTAGATAATAGTGAGTTT
TTATATTTTTGA
>LSDV 03 00145 group 20
ATGTATTCATTTTACGAATTACGTAAATACTGCGATGTATCAATATGTTTTAATGATGTG
ATTAAAATTAAAGGCCATAAAATAATTTTATCAAATTCATCTAAATACTTTGATACCATG

TTTTGTGAAAAAGTTTGTACCTTCATCGTATTGAATAAACAAAAATGA
>LSDV 01 00034 E3L
ATGTATTCTTGTGATGAAGTAGATTCGTATGAACTTGTGAAAAAAATGGTAAATAATCTA
TCAGAAAGTGAGTTTATAACAGCAATAGAAATATCTAGAAAACTAAATATTGAAAAATCA
AATGTAAACAAACAGCTGTATAAACTACATAATGACGGTTTTATTTTTATGATAAGATCG
AATCCACCTAAATGGTTTAAAAAAAATAGCATCGATAACGATGACAATGAGAATAACGAT
ACGAAAAAACTAAATAAATCATTTTCTGATACAATTCCGTACTATAAAATTGTTTTATGG
AAAGAAAAAAATCCTTGTTCTGCGATAAACGAATATTGTCAGTTTACATCTAGAGATTGG
TACATAAATATATCTAGTTGCGGAAATGGTAGAAAACCAATGTTTTTGGCCTCTGTTATT
ATAAGCGGTATAAAATTTTTCCAGAAATTGGTAATACAAAAAAAGAAGCTAAACAAAAA
TCGACGAAACGAACTATTGATTTTCTAATCAATACATCTATTATCAAATTTTAA
>LSDV 01 00035 group 91
ATGGATGATGATAATACTAATTCATATAGTGATAATACTACCCCCACATATCAAGACATA
GAAGATATAATTTATAAATATGTAAAAGAAAAATCAAAGGTAAAAGAAATATTAAAATGG
GCAACAGACAAAGCTTCCAAGTTTTATATAAGAAATATTATTAATACAAAGTCAAATATA
GAAGAAACAAAATTTGAACCAAGAAACAACATAGGTATTGAATACTCAAAAGATTCAAAA
AACAAATTATCGTATAGAAACAAGCCTTTAATAGAGACAAATAAAGATTATCCTGACATA
TGTGATCTTATACGTACGACAAATGGAACAGAGAAAGAAATTTTAAGATATATACTTTTT
GGAATAAAATGTGTTCAAAAAAATGTAGAATTCAATATAGACAATATTAGAGATATAAAT
CACGAAGAATATTTTAATGTTTTAGATAAAAAGTATAACCTCCCATGCCCTGAGTGTAAA
AGTAAAAACACTATTCCCGTCATGATACAAACAAGAGCAGCAGATGAACCACCATTAGTT
ATGCATTCTTGTAGAGACTGCAAGAAAAATTTTAAACCTCCGAAGTTTAGAGCTGTAGAA
AAATAA
>LSDV 01 00036 E5R
ATGGAAAATAATACACCACCTAATCACTTTGAAAAAGCTTCTGTTTATGCATGTAGGTTA
TTTAAACAGTTTTATAACGAAAAAAAATATGATTTAGATAAATGTTTTTTAAAAGTTAGA
AATAGCATGTCTCAATACTATTCTATGGTTGGTGATGATTTAGTCTGGAACAGAGAAATA
TTAAGTTACTTATATGAATACATAGCATATAAAAATAATGCAAATAATCCAAATTTTGTT
TTATCTTCAAAAATTATTAATTTAACTAAGAAAAATACAAAAAAGATAAAAAAATGAAATA
AATTTTGTTGGTCAAACTTACAAATTGGATAAACATATAAAAAAAGTAAATATAACACAC
TATGATTATATAAAAATAAAAAAAACGTATGACGCATTCAAAAAAAATGTTGACATTAAA
TCAAATGATATATCTGTAACACTATTAAAAATTTATAAACAACTAAGCTTAATTAGTGAT
GTTTCAATAAATACGCGTTCTATGACAAAATCTTTAATGTATTACATGTTTCCTGATCTC
TTTAAAAAGGATAAACGACATGTATTTTACAGAGTAGACGCTAAAAAAGGATATTGTGTA
TTATCATCTAAAAAGATAAACTTAATTAGGGTTTTATTAGAATATAAATTTGATATAAAA
TACAAAGAGTGGGGATTAATAAAAAGATATATTAATACTATTTGTAATAAAGGTAGTAAA
AGTAGCAAAATCTGTATTGGAAAATATCCATATTTAAAGTTAAAACCTGTTTCTAAAATT
AAATTTAATTATAGATCTAAATACGTAAGTGATTTTAAGAGAATAGTGAGTTCTAGTTTT
TCATTGGTTGATTTGACAGAAAAAATAACGAAAAAAACTTTTAGTAATATTTTCAGAAAT
GAAATTAGTCATCTGTATAAGTTTAATGCAGAAAAAAACCATCTCGCGCTAGATGAATAC
AAAAAAATGAGAATGTGTAATAAAATAATATCTGCTATAGATTATCCAAATAAAGATCAC
ATTTATAGTGCGATAATAGAAACTGTTAACAATTACTTAGATAATCCTCCGAAAAAGCTA
GGATACAATATTGATACTTGTTTGACTATAGCAAAAAACAATTAA
>LSDV 01 00037 group 146
ATGGATTTTATACGAAGAAAGTATTTAATATATACAATAGAAAACAAAGTTGATTTTTTA
AAAGATGAAGTACTAACAAAGATATCAAACTTTACATTAAATCATATATTAGCCATTAAA
TATTTAATTCTTAACTTTCCTAGAGATGTTATATCAAAAGATGTATTATCGAACCCAAAT
TTTTATGTTTTTTTACATATGGTAAAGTGTGATGACATTTATGAAACTGTTTTAAAACAT
TCGTTCGATATTCCTACGTTATATATAAAAGCACTTATAAAAAATTATCCGTTATTTAAT
AACACAATAGAAAGATATAAAACTTTAGTTAGTGAACTTTTTTTAGACGATAAATTTATC
GAAATAGTAAAATATTCGTCTGAATTTGATAATATCATAGCTGTTAATTACGATCTTTTA
CTAAATCCTTTATTCCATAATAATGAACCGATTAAAAATATGGAAATTATTTATAGTAAA
CTTTTTAAAAAATCTAATTTTAGGAAGGTAAAAAAAATGGAGGTTATCAGATTAATGATA
TGGGCGTACCTAAGTAAACAAGATACTGGATTAGATTTTACTGATTTTGACTCTCAAGAT
ATATATACACTGTTTCAAAAAAGTGATAAAGTTATTATTCATAGTGATATGACAGAAAAG
TTTAAAGAGTATATTTTCGGGTCGGAAAAAACTAGTTATTGGTTATGGTTAAATGAATCA
ATATTTAATGATAATGTCTACAATGAAGGGGGTTGCAGAATTAATGTACGATAAAATTCTT
AGTTTTATCTATTCAGAGATTAAACAAGGAAGAGTAAATAAGAATATGCTGAAATTAGTT
TATATATTTGAAAATGATGAATATATACAGTCTATTTTATTACAAATAATTTACGGGGTA
CCTGGTGATATCTTATCAATTATAGATGTAAAGGATGATAACTGGAAAAAATATTTTATA
GGTTTTTATAAAGAAAATTTTATCGATGGAAAAACTTTTATAAGTGCAAAAACATTCAAC
TATGATTTATTTAAAGTAGTGGCTAAAATAAATCCTGAGTACTTTGACCCAGACAAAATA
ATATCCATATTTGATCATAAACCTGAAAAAGTTAAATATTTTGATACAATAGATATAAAT
AATACATTTATATCAAATATAATTTATGAAACGAATGAATTAAATCTTTCAACGATTGAA
GAATTACAATCATGCCAAATATATAATGAAGAAACAGAATATTTCATTAAAGAATATAAT
ACTTATCTTTATTTAAAGGAGAAGGATCCATATATTTTGTATAATGGCATATTAACAAAT
ATATCAAAAGTTCCATCAAATAAAAAGTTTTCATTGTTTAGTAAAAACATATTAAAATAT
TATATAGATGGAAAATTGGCAAATATAGGTCTTGTGTTACCAAATTATAAAGGTGATATA
TTGGTAAAAATATTATCGCATTTGAAATGTGTAGAGGACGTTACTGTTTTCATTAAATTT
TCAGTATGTAAAAACTCTAGCATACTACCATCAATAATAAGAACAATTTTAGCAAACTTT
AATATTTCAATAATAATATTATTTCAAAAGTTTTTAAGGGAAAACTTATTTTACGTGGAA
TCTTTTTTAGAAAAAACAAATCATTTAACTAATAACGATAAAAAGTATATACTAGAAATA
ATAACTCGTGGAAGATCTTAA
>LSDV 01 00038 group 196
ATGATGGGAGACATTTATTCAGAGTCACAAAAAAGAAACGACGATCAAAAAACATACTTT
ACAAGAAATTTAAGTCCTTTAATGAAAAATACATATTTATACCATAATTATTCATATGGA
TGGATACCGGAAACAGCTATTTGGAGTAGTAGATTTGCAAATTTAGACGTAACAGAGTTT
TACCCAATTACAATAAATTTAATAAAAAAAATTTGAATTTATGCTTTCATTATACGATGGC
TTTACTAAAGTTGAAACCGTATACTGGTCTAGGATATGTCAAAACACCAAAAACAAACAT
AGAAAGCCAGTGATCGTCTCTTCTTTAGAGGGAGATATGAAAAAAATAAGTGATAATTTT
TATAAATCACGCACAAAGGAAGTTTTTGTAAATTCTAATGGAATTATGTTTTCATGTTTG
GACCCGTTGGGTAAATATAATAATATAGGATTTTTATCTATCTTTCATAGATTACAAAAA
ATGTGTATACCTTGTTGTTTTTTGAAAGACCAATCACACACTGAAACTTTTTCATCGTGT
GTATACCAAAAAGATATTATTAGTAACGTAATAAATCCATATATACTCAACTTTGGAAAG
GTGGTAACAACATCAAAGATATCCTTTTTACCCATTATTTTTGATACATTTTTTAATGAT
GGATTAAAAATAATATTTGAACAAGATAATAAAAGACTAAAAGAAACAAGCGGATATCAT
GTAGTTAAATCATGTGAAAATGAAAACATTAAACGTATACGTACAATATCGGATATAATT
TCATTTGTTAATGAAGATAAAAATATTTTAATTTCTGACGATATTGTATATTTTCCAATG
AATTATTTTGATATTGGAAATAAAGTTTATATATTAATTCAGGAAATAGTCCATGAAATT
GTTATGGTAAAGAAAACACTTGATAAAGATATTATAGAAATTTTTCCACCAAATTATAAA
ATAATAAAAGATCTGTTTCCTAGACAAACAAAATCAATAATAATACGTTCTGATTCAGGA
ATGGATTTGACAACAGATGGATTTTTAATTGATGGTAAAGAATTTAATCAAGATTTATCT
TCAAATTATGTAACATTTACAAAAAACATAACAACATCGTATAAATTACATAAGTACTTT
TCTCCACTTTTAAAGTATGTAGTTACAGAATCAAAAGACAGATTTACTAAAACTTGGTTA
ATAAATATCATGTTAAAACTGGGATTAGATATAGAAATGGATCCTTCTATTCTAACAAAA
CTTGAAAAATATTATCCGAATAACGGTAAGTCGATGACAAATTAA
>LSDV 01 00098 VITF3S
ATGTTTGATCCCGTTCCAGATCTTAATTTGGAAGCTAATATAGAAATTGGTGATGTAAAC
ATCGATAAGATAACATCAGGAGAAGCGAGCGAATCTTTATCGTATGCGTCTAAAAGTAGA
CGATTGTTTATTCACAAAACTAAGGATGAAGAAAGAAAGTTAGCTCTTAGATTTTTCTTA
CCTCGTATGTACTTTTTATCATATAAGGAAGTCAACTATTTATTTAGATGTATTGATGCT
GTTAAAGATGTCGCCATAACAAAAAAAAAATAACATCATTGTCGCACCTTATATGATTTTA
CTTATAATGGATTCAAAGGGTTATAAATTATCAGAATCAATGATAGAAGTATTTTTCCCT
GAACTATACAACGAAAATAGTAAAAAATTTAAGTTTACTTCACAAATATGTAGTATACAA
GAAAAGTTAGGTTATAATGTAACAAATTATCATACATATGATTTCGAATCATATTATTCT
ACTTTTGCATTAACCTTGAGGAAAAATACAGAATCGGATATTATATTTAACACCAGAAAT
GAGAGCGAATTTATAAGTTCGTTATCTGAAGTAACATATAGATTTTATATTATACTACTT
AAAAATAATTTAGTTCAATGGAGTTCAAGTACCGGATCCGTAGTCAATCAAATGATTAAT
ACTGTATTAATAACAATTTACGAAATTTTAAAACAAAAAGAAAGAGACAGAAAGAAATAT
ATATGTCATTTAGCAAATGAGACAAAATTTCCATCTGTCTTTTTGTTAGATCGTATGAAC
TTATTTGATAAAATTATTTCAGATATAAAATCTACTAATTCTTTTAAAGTAAGCAAGAGA
GATAAATCAATTCTTTTAACATATTTTCAATAG
>LSDV 01 00099 group 154
ATGCTATGCTATTTTACAATTTTAAAATCTATTGGAGGGTTAGCTTTATTTCAAGTAGCT
AATGGCGCAGTTGATATCTTTAGGCATGTTTTTATGTACTTCTGTGAAAATAATTTTAGA
CCGAACTCATTTTGGTTTGTAATACTAAGATCGATATTTACCATGATAATGTTTTTAATA
CTAGGCATAGTGCTATTATCAATTTCTAATAATGAAAATAAGAAAGAAGACGATTAA
>LSDV 01 00100 group 155
ATGATGCCCATTAACGCAGTTACCACATTAGACCAATTAGAAGACTCGGAATATCTTTTT
AAAGTAATATCATCTATTTTGCCATCATTATGTTTGGATTATAAAGTTGATGAAAATTTG
AAAACGTCATACGTGCATCCATTTGATGTGTTGTTACATCCTGATTATGGAAAGATATGC
GACGAAAATGAAATACAAAATTCTATAGAAAAACTAGGAATAAATTATTTATTAGATGTA
TATTCTAGCAACAAACTTTTTCACAATGTAATTAATCCTGGATATATTACTCCTAATGAT
GTAGTTCCTAAATTTGATTCAAGTAATAATCCAATTGTGAACACACATAGTTTTAACGAT
CTTCCAAAATTTACTAGAGAACTACTAATGTACAGACTTAAGTATCCTGAGTATAATTCT
AGGTTTGTAGGCGGATATGTTAAACCAGTAGAAGGCGGATTCGATATTCAAGTAGAAGAA
AAAATACACCCAGATTTAAACTTTGAAAATACATACCTATTGAACCTTTTATATAAGGAT
GTATTGTCTACTAGTATACAAGGTTTTAGAGTAAGAAAATCTAACGGTGTTATGTTTTAT
AGAGATTTCGAAAATCTATTGGGAGTAAGAAGGGTTATTGATGCAAGATCAATTAATAAG
TTTGATGAAGAATTTAAAATGTTTGATGCCGCAATTGATCACAATACCACATTGCCACCG
TTCCAACGAGCAATGGATACTGATATTCTTACAATGTCTATGAAACACTTTATTATTTAT
TATCAGTACTTTTCTAACGAATACGAAAACAGCTACGTAACATTTAATGGAGATTATGTA
CTGAATAGCATTCCTGACTTAATTAGTATTATAACTTCTATGCGATTTCAAGATCAAATA
CCTAAACTGATTGAATTATATCCAGATTTAACAGTACAAGATTATGCCGATCTTCTTTTA
TTGGACACCGCAACAAATAACAGATACATCCATCGACTCAATAACTTAAATATTAAATAT
GTTGATATTTCTGGACGAGATCATTATTATATAAATTTACTTAACTTGTTGGCAAAGGAA
TCCAGATCTAGAAAATTAACATCTAAATTGTCACTATTTTGGGATGGTATCGACTATCAA
GAGTATAAATCAAAGAAAATATCTGATATCATTTTTTATAATGCAACATGTTATGTGATG
GCATTGTTCAACAAAAACGGAACAACATATTGCTCAATGCTTTCGGACGTAATCTCATCA
AATGAAACACCTTTAAGGGTATGTTTACTACCTAGAGTACTATCAGGAAAGACGGTATCA
AAACTAATATCTGAAGTGTTAGAAAGTGTAAACTCTATATCTAAGAAAGATTTTCCAAAA
AGACCAGCGTCCAAGCTAATGCATATCGGACTGTCTGAGAATAGTTTTATGAGATTTTTT
CAACTACTTAGATTAATTACTAATAAATCTCCAGAATTATCAATTAAAGAAGTTTTAATG
CTATACGCTGGATTAAAAATTGACGATAATGGTTCACCACACTTAATAAAAAAGGAATCT
TATCAAGATTTTTCCATTCTCCTTTTTTCGGCAATGGGATTTAAAGTTAGTATTAAGAAA
TCTATAATTGGAAGCAATAATCATACAATAATAAGCGTAAGACCTAGAGTATCAAAACAA
TATATACATAATATGTTAGTAAAGGCTAGCTGTACAAAGGAAGAAGCCGATAAACTAATA
TCGGCGTCGTATGATTTATTACATTTTATGGTATCTGCAGGCGATTACCGAAATTATCAA
GAATACTACTACACGAAAAACTTCTTTCCTAGTTACTTTTTTTATGGTGGGAATCCTCAA
GATAGTTCGTTGCCGCAAAATGTCGATGAAGAAACAACACTAATTCATATATCTGAACCA
ATAAATATCTTGGATAGGATAGATATTAGAGGTATATTTTCAGCAAATACAACAGATGAA
ATGATGACTGTGGACGCATTTGGACCAGAAAATATCGTCTTTAAAAAAAATTTAGAAAAT
CTTATTAGGGATAATAAACTATCTGGTGATACGTTATTACAGACGATGCCATTAAATATA
TTAGATAAGTTAATAACCGTTGCGGGTTCTTGTAATGTTTCATTTAACGATCTTATAGAT
GGAATATCTAACGAAAATGATGATTGTGATTCTACAAATGATATAACTGAACTTATTAAT
ACAGCTCTTAAAGAAAATTATGCTAAAAAAAACACATCATTGGTAACACAAACATTTAAT
TTTAGTAATAAATATTTTATAGAAAACCAAAGAAATGACATAAGTATTAATATATCAAAT
TTAGATAATCCAGAGGTATCAATAAATGAAATAATTAAATTTATGTATAACAGGAAATTA
GATAAAACTCTTAAAGATTATGTAATCTTAAAAGATATGCTTAAAATAGCAGATTATCTT
ATTATAGATGATATTATCCCACTTTGTATAACATCTATAGTTAAAATTATAGATTTTAAT
AATTGTCTCGATGCATGTATTTTTTCAGAATTTTATAACTTAAAAAAACTAAATAAGTTT
ACTTATAAATTTATACGGAAAAATATACGCAAAATATATTTAAAAAAAGATTTTATTTAT
TTATCATTTAACGTTATTGATCTTCTTTTACGAGATAAAAAAACTGTATTCTATAATGAA
GATGATGTATTATGTATCATATTACAATGGCTTAATAATGAGGCAAATAATAAATATTTT
CATGATGTTTTAAAAGTAATAAGATTTTCACTTTTGTCAGAAGAATGTGCCGACAAGCTT
AAGATAAAATATAAATACTTATTTCCTGATAGTTTATTAAATAAGTCATTATTTTCTTCA
AAAATGTCACGCCGTGAATCAACGTTTGGATCGTTATATATATATCCGATCCAAATTGGT
ATAGTAAAAAATATGATATTATGTACATAA
>LSDV 03 00147 group 14
ATGATTTTGTTATACGATTACGTTACGCTTACAAAATCGAGAAATATCAAAGTTAAAAAT
ATATTAAAGCTTATAAAAAATGATCAGTTAATCGATTTTAATTATAAATTATTTACATTG
TGTGAATATGTAAGCAAAAAACATATCCGTATAGATGTTTTAAAAATATTGTTTGAAATA
GGGTGTAAAGAAAATTTACATAGGTTATCATATTATACTCTTCTAAGTTTCTTTAAAAAT
TATAAAATTAAGTATAATTTTAATCATGTTAAAAGTATTATAGAATTAATAACTAGTTAT
GGTGTATCGTTTAATGATGAACCAATCAAAAAAGATTCATATCCAATTTTATTTTTAGTA
GGTAACGAAAAATTTAATCATATTAGCCTTTATGAATATTTAGAAAATAATAATGTTAAT
TTTAATATAATAAGATCTGATGGATATAACTTGCTACATTTATACTTAGAATCATGTAAT
AATATAAAATTAAGTGTATTAAAACTACTCATTAAAAATAATGTAAATGTTAATGGATTA
ATTAGATTTAGAAACCTAACACCGTTGCATATATATTTGTGTAAAAATTATTGCTTAAAC
TATAGGGTAATAGATTTCTTAATAGATTCTGGATCTGAAATAAATAGCGGCAAAGAAACT
TTATTATACTCGTTTTTTACCACCTGTGATAATGAAAAAGTATTCAAATCACTAACTACT
CAACTTATTAAAAAAGGAGCTGATATAAATCAAAAATCAGAAAGTGGGTTAACACCATTA
GTAGGATTTATCGCATATTCAGATATAGCTACTCCTAGCAATATAAAATTTATACTAAGT
TGTGGCGGAAATCAATTAGTTGTAAACCCTCAAAATAATGAAACTTTGTTACATATTTAC
TTACGTAGGTATGATGTATCGTTGCCAACAATATCAACGTTGTTAGAAAGTGGTATCAAT
ATAAATTCTGTCAATTTTAAAAATTACACTCCTTTACACGTATATATTGATAAAAATGTA
GATAATTTATCTTTAGATGTTGTAGAGTATTTAATCCATAAAGGTGCAACCACCGAACGA
GTTAACAGGTATGATTGTTTTGCAAAAACTATATTAGAAGTATTTTTAGAAAGAAACAAG
TTTTTAAACAATTTTTCAAGATATTTCATTAACTATATTTTAAAGTATTTCCCAATAAAT
GAAAAAGATTTATATGGATTTACACCACTTTTATCTTCTGTATATGCAAATAATGTAAAC
TTTTTTAACTATTTTTTGAGATTAGGATCTAGTATAAATGTAATTTCAGAAATAGGAGAA
ACGTGTGTAAGTATACCAATTGAGAATCAATATAAACGGTTTTTAAAAGTAGTTTTAGAT
AATAAACCAAATATAAAAACAATAAAAGCTACTATTAGATATCTTAGTGATAAGGAGTTT
GATACAAGAACAAAGTTTAAATTGATGAAACATTGTATTAAATATTTATTTACTTTGGAT
CCTGAAGAATATAAAAATCATAGGTTTATTTCTTTAAAGTTTAAAGGTGTTGTCAATGAA
TGTCAAAAAGATATTATTACTATGAAAAGTACATTTTTAAAGACTGTATCTGTTTACGAT
TTAATATTTAACAAAAATGGAACAATGCATAAAAGGTACTTAAATACCATGGAGGTAAGA
AGATACTTAAGATCAAATATTTATGCGGAAAGAGTTAGATGTATAATAAATAATTCAATA
GAAAAAGATAAAAAAATCGATTCCATAATCAAAAAAGTTAATATCTTATGTAAGAATACT
ATTTGGGATATATTGCCTGAAGAAATAAAAAACAATATCTTTAGCTGTATGTCGTTAAGG
GATATTAGAATAATCTTTCATATGTTTGTTGTATATAAGCCTTACTGTTAG
>LSDV 03 00158 group 113
ATGTCTTCCGGCAACTATGTCTACCGAAACAACTTTTCAGATGATGATGATATCACAACC
GCCATCTCTGATTATTTGTTTTGGTCATCATTGGCATTTTCCTCCAGGGAGGTTGCTGGA
AAGGTGTTCTTAGTTTTTGAATCTTTCAAAAAGGATGCCTCACTTGTATTTGGAAATGAT
CTAACAGCTTTTGTCAAAAACATGTTTTTGGATTCTAAAATTGGATTTGAACAATCAAAA
ATTATGATTAATTCCATGTTGAAAAAAGAAAATTACATTAGGGAATCATGTGCAGTGATT
GGCATTTTAGCAAGAGCAGCAGAATATTGGGGTGGTGAATCATCTCCAACTTGTTCTTCT
GTGAAAGTGTTGGTATTGCTTCGAAACCTCGTTTCCGACAACGATATTTCGCTAGTGAAA
TCAGCACTAATGATTAGACTTAAAAGATTGAATGAAAAAAGTATCCATTTACAGGTTTAA
>SPPV 01 00002 group 22
ATGATTACCTCATCGACAAAAAATCTGTTTTTTTTTGTTAATATAAACTCCTTAGACAAA
AGAAAAAGAAGTTTCAGTTTTCTATCATATGTAAAAATCCTTGAGAGGAAAAAAAGGTTT
GTTTTCAAGTTTTTAGTTAATAATAGAAAAAAAAAGTTCAGTAGTATCGAGGTGGTTTGTG
TCGACAATACGTCACAAAAAACAACCAACCAATATGTCATCATTAACTTTTTTCATTTTC
TGTTTGCAACTATTCATTTTTACTAGTACTGTAAGCGGAATATCAATAAAGAGATGCACA
GAAGAAGAAAATAATACATGGGAAATCGAAGTAGGATTATGTATACAAACAGAGAATTTT
AGAGCAATTAAAACTGGCTGTTATAAAATTCAAGGACCTGGAGGACTTCTAACAGAGGGA
AATGGATTTAAAATTTTTGCACATGATGATTGTTCCAAAGAAAAAACACAAAACAACTTT
ATATTAGATAGTGTTAACGAAGCAGTTTATGCATTGGGTAAATATGTATATATGGAAATA
TCAACCAGTAATATAACAACTCTGAATTCGCTGCCACAGTGTGCTAAGAGAATATCATTG
TCAATTTCTTGCGATCAAGTAACTACAGAAATGAAATCATACGTTGAGAGTGTAAGTTTC
AAAGATTATGATTTGGAATTTGTTATAACAACAGATATTAGTTGTGTAAAACATGTAAGT
TCAAGTGTTATAGTTAGAAACGAATGTGAAAAAAAATATATATCTACAGGAAAAAAGATT
TTTGGGTTTAATAATAAAATAGATTGCTCAGCTGTAAAATTTAGTGAACATGTAAATTAT
TTAAAAACGTGCAGTGTTGGGAAATTTGATAGAAAAAAATATTATGAACATCAGCATAAT
TATATTAAAAAAATATTTCATCATAATGAACTATGA
>SPPV 01 00124 group 63
ATGTTTTATTTTAAAAAAAAGAAAATAAACATAATATTTCTTTTTTTGACAATTTATATT
AATTCATCTTTTTCAATATTAGAAAACAATTTTTCTTCAATAATTTATTCAAAATGTAAT
AAAACAATTGTCATCGATTGTAACATTAATCATTTTGTTAAGTACAATGAAAATATTACA
ATTAATTGGTTTTTTAATAACTACCCACTTTTAAAAAAAAAATAATACTAGTACTCAAAAA
CTGATATTGGATTTTTCAAAATACTTATACGGAAATTATACCTGTTATGTAATTAATAAC
GATAATATACTCTTAAAAAAAACTATACAATTAAAATATATTATAAAATGGTTAAATGAA

CCAATATTACCATATGAAGAAAAAATTAATACAGAATTTATTTCAAGAGGTTCTTTTTCT
GGAAGATATATAAATTTTTTAAGAAATTTTTCATTACTACCAACTAGTGAATTCATTTCA
TTTTTATTATTAACATCTATACCAATATATAATATTCTTTTTGGTTTAAAAATACTCAA
TTTGATATTAAAAAACATACACTTTTTAGCGAAGTTTATACCAATAACGATAAACATATA
GAACTAGGAAAATATTTTAGACAAACCGGTGATTATAAGCCTTTATTTAGCAGGCTAAAG
GATAATAATATATATACTTTGCCATTTCCAATCAACCAAAGTAACATTAACAACCCAACA
TTACCTATTGGTTTTTCCAAATCAGATTATGAAACATTATCTAATTTAAGCGCTATACTT
TATTTAACTAATTATGATCCTGTTTTGATGTTCTTAGTGTTTTATGTGCCTAAAATATCT
ATAACGACAAAAATAACACCAGCTGTTAAATATTTAATGGATAAACTAAATATGAATGAA
ACGGATATAACATTAGTTTAA
>LSDV 01 00039 POL
ATGGAAATAAAGTGCATAAACTGGTTCGAAAGTAAAGGAAACGAAAGGTTTTTATTTTTA
AAAGCCAGAAATAGACAATCTGAAACCATATTTTTAAGATTTAACTTTTACTTTTATTAC
GCAGTTACAGAAGATGTTTACATATCATTGTCACCACCAGCATATAGTTCTGATTTTTTA
GGAAATATGAACTTAATAGATATTAGTGAAAGAGTTAGCAATCATGTTACCGATGTAGAA
AAAAAGAAAACAAACGTTAACGTCTGGTTGATAAAAGAACCACAAAAAAGGAATATTCCA
AAAGCGATCATGGAAGAATTTCTTAACGTTAGTTGGTTTTTTATATCAAATAATATATCA
CCAGAAGGTTGTTATAGTATTAACGTAGAAAAATTAAAAAAAATTAATGAACAATGCTAT
CATTGTGATTGTCCTAAAGAATGTTTTGCAACACCGATAAACAGATTTAACATAAATAGA
TCTTATCTTTTCTTAGATATAGAATGCCAGTTTGATAAAAAGTTTCCTTCCGTTTTTACA
AATCCAGTTTCTCATATTAGTTGTTGTTATATTGACCTAACAAATTTAGAATTTAAATTT
ACTTTAATAAACGAAGATATCTTATCAAATAAAGATAAGAAAGACGCTACGTTGAAAGGA
TATTACAAAGCTAAATCTGTTTCTGATGTAGATTACACAAAAGAAATAATAATATGTAGT
GAAATTACCATGCTAAAAGTTGCAAAAAAGCTTTTGGAATTGACATTCGATTATGTAGTA
ACTTTTAACGGAAACAACTTTGATTTACGGTATATTTCAAACAGATTAGAGCTGTTAACA
AGTGGGAAAATCATTTTTAAATCTCCAGATAAAAAAGAACATGTACATTTATGCATATAT
GAGAGAAACTTGTCTAGTCATAAAGGCGTTGGTGGTGTGGCAAATACTACATATCATGTA
AATAATAATAACGGAACAACATTTTTTGATTTATATACATATATACAGAAGTCAGAAAAA
TTGGATTCGTATAAGTTAGATTCTATATCTAAAAATGCGTTTAGTTGTTCAGTAAAACTA
AAAAGTGAAAACGACAATTTGCTTACCTTCGTGGGTGATAATACTACAGATGATATAGGA
AAAATATCTTCGTTTTCTGAAGTTTTATCTACTGGGAATTATATAACTATTGTAGATGAT
GTTTTTAAAATAGTAGACAAATCAGTTGGTAATGATAAATTCCAGTTAACTATTTTAAAC
AAAAATAACACTTTGAAAAAGAATATCGATAATGATATTTACACTATTTCTTTTGGAAAA
GACGATGTTAGTTTATCTGATATGTATAAAAACTATGATTTAAATACATCAATTGAGATG
GCAAAATATTGTATCCATGACGCCTGTTTATGTAAATATCTTTGGAATTACTACGGAATT
GAAACAAAAATAGATGCTGGAGCTTTTACTTATATATTGCCACAATCCATGGTGTTTGAA
TACAGAGCTAGTACATTAATAAAAGGACCTCTATTAAACTTATTATTAGATGAGAAAATA
ATTCTATCACGATCAGAAAAAAAAAAATAAATATCACTATGAAGGTGGTAAAGTATTTGCA
CCGAAACAAAAAATGTTTATAAATAATGTATTGATATTTGACTATAACAGTTTATACCCT
AATGTATGTTTATTTGGAAACTTATCCCCTGAAACATTAGTATCTGTATTTGTTGCAAAT
AATAGATTAGAAGCAGAAATAAACAAACAAGAAATAGAGAAAAAGTATCCTCCGCCCCGT
TATATATCAATCCATTGTGAACCTAGATGTGATGATTTAATTTCTGAAATTGCTGTATTT
GATAGGAATGTAGAGGGCATCATTCCAAAACTTTTAAAAACATTTTTAACAGAGAGGGCT
AGATACAAAAAAAAATACAAAGAAGCAACGCTGTCGACTGACAAATCTATTTATAACTCC
ATGCAGTATACTTACAAGATAATAGCCAATTCTGTTTATGGTTTAATGGGATTTAAAAAT
AGCGTATTGTATTCATATTCTTCAGCTAAAAGTTGTACAGCAATCGGAAGAAAGATGATT
GATTATTTAAACTCTGTGTTAAATGGATCAAAGGTAGAAAATGGAAAAGTATTATTAGCT
ACAAAACCTATAAATCCATTTTTTGAAGATGGTAGAAATGTAGATATTAATATAGATACA
AAACTACCGACAGAATACACATTTAATTTTAAAAGCGTTTATGGGGACACAGATTCTATA
TTTTTAGAAATGGATACAAATGATATATCTAAATCGATAAGAGTTGCAAAAGAACTTGAA
AAAATTATAAATGAAAGGATTTTGTTTTCTAACTTCAAAATAGAGTTCGAAGCCGTATAT
AAAAATCTTATAATGCAATCGAAGAAAAAGTATACCACCCTTAAGTATTTGCCATCTTTC
ACCAATGAATCAATTCCTGAACGTATCAATAAGGGAACTAGTGAGACAAGGAGAGACGTG
TCTAAGTTTCACAAATATATGATAAGAATATACAAAACAAGGCTTTTGCAAATGTTATCA
GGTGGAAATATGTCATCTGTACAAGTATGTGTTGAAATTTTATCATCGTTAGAAAGTGAT
TTACAAATAGAATTTGAAACAAAAACAGCTCCATTGGATATGTTTCTATTGAGTAGAACA
CATCATTGTAATTATAAATCAAGCGATAATCCAAATATGTACTTAGTAAATGAATATAAT
AAAAATAATGATGAAATTATTGAAATAGGTGAACGATATTACTTTGCATACATATGCAAT
AAAAAAGAACCATGGCAAAAAAAATTAGTAAATATAAAAACGTATGAAAGAATTATAGAT
AGGAGTTTTAAATTAAAATCTACTGAACGAATATTTTACGAAGTATACTTTAAAAGACTC
GCCACTGAAATAGTAAATTTGTTAGATAACAAAGTATTATCCATATCATTTTTTGAAAAA
ATGTTCGGAACTAGACCTATTTTTTATAATTAA
>LSDV 01 00040 group 92
ATGAATCCTAAACACTGGGGAAGAGCTATTTGGACAGTGATTTTTATCGTTATAACAAAA
ACCAAAACTCATAAAAATTTAGAACTATGCAAAAAACATATATATACTATAGTTGAAACT
CTTCCTTGTCCTATGTGTAGGATTCACGCAATAAGGGAAATAGAAAAAAATAATGTTATG
TCTAGCGATGATTTAAATTATATTTACTTTTTTTTTATTAGTTTATTCAATAATTTAGCA
TCTGATGAAAAATATAAAATCGATTTAAATAAGGTATTACCGCTTTAA
>LSDV 01 00041 group 120
ATGGAATTAGTTAATATTTTTTTAGAATCTGATTGCGGCAGAGTTAAAATAGAGGTACAA
AATAATACCAACGAGTGTGCGTGTAAAAAAAGTTCAGTGGTAAAATCAATAGATACTTTT
ATTGATATAATTAAACAATACATAAATGTTGAAAAATCAACATTTTATTTAGCAATTAAA
CATCAAGATATTTTTATTTTTAAATATGATAAAGGGAAAATATCACAAATTGAAAATGAA
TTTTTTACATTTAATAGTGAACTAATGTTCGTTAATAATTATAACAAAATAACGGGTATA
GAATTTATAGTTACTGATACAATGTCGATGCACATATATCCAAAAGTAGGTCTATCAGTT
ATTTCATCATCTAACAATAATAAGTTTTATTAA
>LSDV 01 00042 group 46
ATGTCATCGTCTTATATATATCCCAAACGGGCAAGAGATGCTCTATCTAAAATAATTTCA
TCAGTGGCTTCCGTATCACAAAAACAGTTTAATGATATTAAACAAACATCTTGCCAAATG
GCATATATATTTAAAAACTTAGCAAGATCAATATACACTATAGAAAAAATTTTTAACGCA
AAACTTAGCGACGATGTAAAGGTTGAGCTATTAGAAAAACTAAAATCATTTTCACACCTT
TCAAAATCGTTATATCAAGATTTGATTTCCGTAGAAACATTAAAGGCTATTTTGTACATA
ATTAAAAGAAATGGAAGAACTATCGACGATGTAGAAATTGGATCTGATGAAATAAGAAAA
TCGTATGAAATAATTAAACCTAAAATTATTAATATGACAAATTATTACACTGAAATGAGT
AGGGCATATTTTAACTTCATCAAAAAAAATCTTAATATGGATGATAAGAATTTAATCACT
TTTGATATAGAATAA
>LSDV 01 00101 group 156
ATGACAACTATACCGGTTACAGATATTCCTAATGATTATAGCGTTACTGCGTTTTCTGAA
GATGGATATCCATCAAATAAAAATTATGAAATAACTACTGGTCAACTATCTATTTTAAGA
ACAGTAAACGATAAATTATTGGCAAAAACGAAATCGCCATCATTTACAGAATTAGAAATA
GCCAACTCTCCTGATTTTTTTATACCAGGTGAGGATTCACCTATTACCATAATAGAACAC
GTCGAATCTCCTCAAACAAAATTTATAGATAATAGTTTAAAATCGGAAATAGTATTAGCA
GAAAAACAACGACAACAACGCGTAAATATAAGGGTATCAGGGTTAGAGACGGTGATAGAA
AAAGAACCTATAGAGGAGCACATGCGAATAACTTCTATTCCATCTAAAACACCATCGCTT
GGTGTTATGTTTGATAAAGATAAAAGGATAAAACTACTAGAGGATGAAATATATGAACTT
AAAACACAACAAAGTAAATTACTTAATACTAATAATAATTTAGATAATTTTACAAAATTA
TTATTTGGGAAGAATATACACCAGTCTTCAGAAATAAATAAAAGAATAGCGATAGTTAAC
TATGCGAGCCTTACAAAATCTGAATTAACATTGGAAGATTTAGAAATATGTTCTGAAGAA
GAAATAGATAAAATTTATAAAGTTGTGAAACAACATAATGATAGTTATAAAAAAAGAATT
ATCGTGACCCATTTTATAACTATTATAATTATAATATTGGAACAAATATTAGTCAAATTA
GGTTTTGAAGAAATGAAAGGTGTGAGCAACGAACTTACATCAGAAATAATTGATTTAAAC
ATAGGAGAAGATTGTGAAGCGATAGCTGTAAAAAATTGGTATAGTTAATAGCCCCATTATT
AATATTGTTATATTCTTAATTAAAAAACTTATGACAAGGATAAAACTTTGTTAA
>LSDV 01 00102 group 60
ATGTCTGATAAAAAATTATCTCGAAGCAGTTATGATGATTATATCGAAACTATAAATAAA
CTAACTCCTCAACTAAGGACCATTCTTGCCCACATTAGTGGAGAACAAGCATCTCAAAAA
TCAAATCTTACTCCAGAGGATAATACTACTAATAATACAGATGAGAATGAAGTAAAAGCT
GGCAATGTGAAAACTAAAGCTTGTATGACAAAACCAAATAAAAAATCAAAATCTTGCAGT
AATAAACAAACTACTTCGAGAAGCAGTAACGTATGTTCTTCGAAAAGTGTTAATAATGGA
GCTGTTTTTAAAAAAAGAAATACATTTAACGAAACTGATCAAATAATGCAAGCAGTAACA
AATGGCGGAAAAAATAGTATACGGAACCATGAAAGAAGGAAAATTAGAAGTTCAAGGTATG
GTAGGTGAAATAAATCAAGACTTGTTAGGAATAGAATCAGTAAATGCTGGTAGACGAAAC
AAAAATATTTCACAATCAAAAAAAAAAGTTAATTAAACGTGGAATGTATAAAGTAGAAACC
GCCGATGACTCTATCGACGATGGTATGGATTAA
>LSDV 01 00103 group 99
ATGATTGGTGATATATTTTTAGTCATCATTTGTGTCGTAGTAATAGGACTAATAGTTTAT
GGAATATATAACAAAAAAACGACTACTAATCAAAATCACGAAGCTGAGAAATATGAAAAA
ATGAATGAGTTAAAAACTGGATATGTTGATAAGTTGGAATCTAAACATTTAACATCTTTT
TATAAATTATTTTCTAGTAAATAA
>LSDV 01 00104 group 185
ATGGACATAATGGGTATGATTAGTAACTATTTTAGTACAGCACTTATTGGTGGAATAATT
TTACTTGCGGCTTCATGTATTTTTGCATTTGTCGATTTTTCTAAACATAAAAACAACAAC
GGGATAACTGTATGGCGAGCTTTGAGCGGAATCGCATTTGTTTTAGGAATAGTGATGACT
ATAGGCATGCTTATTTATTCCGCATGGGGCAAATATTGTTCTCCTAATAAGGTTTTGATA
GATGGTGGTAGATACAATTCAAGTGCCATAGAACTTAACGGACAATAA
>LSDV 01 00105 group 131
ATGTTCGTAGACGATAATACTATAATAGTTTACAAAAAATGGCCTCTGTGTTCTGTATAT
GATAAAAAAGGATTTATTTCTTTTCCTACTAACAATTCTTGTACATTTAAAAATTTTAAC
AAAATTAACAATGATGATAGCAAAACGATATTATTAATAAATCCATCGTTGGTAGAATTA
ATGTTATTTTGTGTTTATATTAAAAGAACAAAGTGGAATGGTAAGATAGCTATCATGTTT
AAACGTGATAATAAACTCCCACCTTTTAGGCTAGTAAATGATAACTAA
>LSDV 01 00106 group 157
ATGGGTGGATCTGTATCTGTAACAAATTTAAACGTATCAAAAGATTTAGCGGATTATAAT
AAAAAGTATATGTTTATAAATTTTAACTATCCTGAATATAATAAAATTATAACGTTTTTA
GAAGAACAAAAAATATATAACGATGATAAAAAATCGGAAATTTTACCTAATTTTTGTTTA
ACCGATAATTTAAAAATATCTCACTGTGGCAACTTTGTCTCTGACGAAATATCAAAAAAG
TACATTTTGGTTAAGGGGGATTCGTGTAGATCATTTAGTTTTAGACCAGGATCTATGATA
TTATATACAAATGATATTACTGAGGATTACTTAGATAATAAAATCCCAGAATCCGCAAAG
GAATACATATTAAAAGGTACTCAATGTAAGTTTATAAAAAAAGATTATTTTATTAATGAT
GATGATATTATAAAATGTTGTACAAATCCTAGTATTGGAAACTGTCCTAAAAAGTTAAAT
AACGAATACCAAACATCTCATTGTGATAATTCGATGTCTTTTTTTTGCAAATCAAACCCC
GATAATGTACAATGTTTAAAATGGCTAAGAACAAAAAGAAAAATAGCACTAAGTACATAT
ACAGACATATGTTCTAATAATATGGATAAAAGATATTGTTCTGAGTTTATTAGGGTTGTA
AGACCTAATTTTTTTACATTTGGAGATATAGCATTATTAAGTTACTGTAATAAGAATAAA
GGAAACAGAAATTGCTGGTGTGTGTCACCTCCAAATAATATTACGTTTGATAGATATTTA
GGCCCTAGAGTATGTTTGTTACACGAATGTACTGATAAAACTAGAGATAGAAAATGGTTA
TTATATGATCAAGATATTCAACGATCTAGATGCAAATATATAGGATGTAACATAAATATT
AATTCATTGACTTTAGAGAACTCTAAAATTGATCTAATATCTGATTGTAGTAAAAATAAA
AATATTATCGGTGATCTTGATCCTGGAATACCCAAAGCTAAAAAAAAAAAGAGATCTTCCT
AATATAATAGGATTTCCATTTATTTTTATTTGTCTTGCTGTTTTATTTTATTTTTTGGTA
ATATATAATCGTAAAAAAATTAAAACAAATAATATAAATGTTCGTAGACGATAA
>LSDV 01 00107 group 100
ATGAGTTATTTAAGTTATTATAATATGTTTAGTGACTTCACTGCAGGAGCAGGCGTATCT
GATAATGAATTATTTACTAGAGAAGAAGAAGAAGCATTTTTGCCAAAAGAACATCATGAA
GAAGGAGAAGAATATAAAATATCAAAACATCACTCATTAAAAAATCGTTTTCCTAATATT
CTCATGAGAAGTGATATTAGAGCATTGATTGGATTGATTTTATTTGTTTTAGCTATCACG
AAGGAAATTAATACAGTTTTTATAATGCTTTCTATTTATGTTATAATACTATGGGGTAAA
ATAATAGCTACAACAATTAAGTTTAAACTTAGTTTTAAAAAAGATAAAAAAATAATTTAT
CTATATTATTACATAGGATTTGTTATAACTCTGATAATGTTACTAGGACAATACATGATA
GGATTAAACACAAACAATATTTTTATACGTATTTTAGGAACTTCATTTATACAAGTATCT
ATTTTTATATCTATAATATTACAAATAATATTTTTTAAGAAAAAATTTAAAAACACATTA
TCGCTAATAACAGTTTTTTTGTTACATTTTATATCATATATAATATCACTAATTATGTTT
ACTATGTCCCTTATAGGTTGTTATAATGAAATATATGGCAAAGTCATAGTTTATAAACTA
TCATTAGTTATTATTTTTGAATTTATTAGTTTATTATCATTGTTTATATTTTCTTTAAAT
ACTAGTATTAAATACAGAAAACTTCATATAATAGAAGACGAAAACTTACTTTTTTCTATA
GAATAA
>SPPV 01 00142 group 31
ATGGAATACATTAGTAATTATGACGAACTATTAAAAGAAATTTATATCAATCAATCAAAT
TTAAAAATAAATGTGCTAAAAAAGATAATAAATGATATACCTACAAAATATCATAAAGGT
ATTTATAACAATTTATTACTAACAGTTTTGGGCAACAAAAGTATTAGTCTTAGTGTACAA
AAGTATAAGAAAATTTTGTCATTTTTAGTAGATAATGGCGCTGATTTAAACACAAAAATT
AAATACAAATATAATGCTTTACATTACTACTTATATAGTAATTCAAATGTTACAGTTGAT
ATACTGAAATTTTTAATAAAAAAAGGAGCTGATATAAAAAAAAAGTGTAATGGTGATAAT
GTCTTACATACATATCTGTGTAATAAAAACATAGATTTTAAGGTTATTAAGTTTTTAGTT
AATAAAAAGATAGACTTAGGAGATAGGAATTTAGATAACCATACTCCAGTTGATATATAT
ATAAGTAATAAGAGAAACAATTGTGAGATTGATACATTGAAATTATTATTTCTTGTTGAT
TTTAATATATACAATAAGGAAGATATTTTCTTATCCGCATTAGATGTATTTTTAAATTAT
TTAAAATCATATACACGTAAATCCTTAGATATAGTTAATTATATATTAGAAAATATATCA
ATAAATTCTGTTGATTCTAATGGCTTTAATCCAATTTTATACGCTACGGTATCTGGGCAA
AAAGTATTTTTTGATTATTTTTAAAATTAGGATGCAATATAAATATAACAACATCATGT
GGAGAAACATGTGGATCCTTATCATTGATGGATTGTGATTATTGTATTTTTAAAACTTTT
TTAAGCAAAAAACCAAATTTACAAACAATAGAAAATACTTTAAGTTACTTATCAAATTTT
TTAGAAAATATTTACTATTGTGATTTAAAGTTTAAAATGTTTAAAGAATTACTTTTAAAA
GCTTTTATGTTAGATAGTGAATTTTACAATAGACACGAATCAATTAAATTATATTTTCCA
AAAACGATTTCATTGTATAAAGAACCGATAGTACAAATGTGTGGTGATAAAATAGGTAAT
AAATCTGTTTATGATATTATTTTTAAAAACAGTGATATTATGTGTTGCTATAATGATTAT
ATTAACAAATATACTAACTTAAAATATTATGGAAATATTATAAGGAAATATATTTTAGCT
TCTAAGATGAGAAAAAAGAATATAGTTAAGATTATAAAGACAATTAATATTGCACCTTAC
TGGAATACATTACCAACAGAGATAAAAAATGTACATAATTAATTTTTTGAGTGATAATGAA
ATAAAGTTGTTGGTAATAAAATGA
>SPPV 01 00143 group 26
ATGGATATATTTACACATCTATCATCTAAAGCATCTCCCAATTCAAATGTGTTAATTTCT
CCTGTTTCTATATCATCAATACTTTCAATTTTACTTTTTGGGTCAAATGGTGATACAGCT
ATACAAATATCATCAGTATTAGAGAGTACATATAATAATTGCTGTTCGGACGATATAATC
ATCGCAAATAGAATTTATGGAGATTTGAATTTGCATTTAAAAACAAAATTTATTGATAAA
TTTGGGAAAGAACTTATTTTGGTAAATTTTAATCATAATACCGAATTAATAAAAAAATGAT
ATTAATGAGTGGATAAAAAGATTAACTCATGATAAAATAAAAAACTTGATAAATGAAATA
AGTGAGAATACAAAGCTGTTATTATTAAATGCTGTGTATTTTAAATTAAAGTGGAGAAGT
CCTTTTAATAAACAACAAACAAAAATGGAAAAATTTTGGTATGATGATAGTAGATATGAA
AATATTGAAATGATGAACGATGTTAATGTTTATCCTTTTATTGAATTAAAAGAACTAGGA
TTAAAAATTATTGAACTACCTTATGAAAACAATTATTCAATGATAATATTGTTACCAAAA
GATATTAAAAAAATAGAAAAAATCTTAACTGGTAATAATTTAACTTTGTGGATAGACAAA
ATGAACCTATACGAAGTTAATGTAAAAATTCCAAAGTTTAAAATAGAAGAAACGTATGAT
TTAAAACTAAGCTTAATTAGTTTAGGTATTATTAACATATTTGATGGATCCGCAGACTTT
TCTAATATGACAAAAAACAAAAACCTATCTATTGATAATTTTTACCATAAAACTTATATA
GAGATAGATGAAGAGGGAACAGAATTATCCGCAGCATCATATTGTTGTGTAGCTGATTGT
GGATTCAATGAAAAAGAATTTATAGCAAACAAACCTTTTATATTTTTAATAAAGGATAAT
ATTAACGTATCTTTTATTTTTATAGGAAAATTTAGTTTTCCTCATATTCGTTAA
>SPPV 01 00147 group 21
ATGATTACCTCATCGACAAAAAATCTGTTTTTTTTTGTTAATATAAACTCCTTAGACAAA
AGAAAAAGAAGTTTCAGTTTTCTATCATATGTAAAAATCCTTGAGAGGAAAAAAAGGTTT
GTTTTCAAGTTTTTAGTTAATAATAGAAAAAAAAAGTTCAGTAGTATCGAGGTGGTTTGTG
TCGACAATACGTCACAAAAAACAACCAACCAATATGTCATCATTAACTTTTTTCATTTTC
TGTTTGCAACTATTCATTTTTACTAGTACTGTAAGCGGAATATCAATAAAGAGATGCACA
GAAGAAGAAAATAATACATGGGAAATCGAAGTAGGATTATGTATACAAACAGAGAATTTT
AGAGCAATTAAAACTGGCTGTTATAAAATTCAAGGACCTGGAGGACTTCTAACAGAGGGA
AATGGATTTAAAATTTTTGCACATGATGATTGTTCCAAAGAAAAAACACAAAACAACTTT
ATATTAGATAGTGTTAACGAAGCAGTTTATGCATTGGGTAAATATGTATATATGGAAATA
TCAACCAGTAATATAACAACTCTGAATTCGCTGCCACAGTGTGCTAAGAGAATATCATTG
TCAATTTCTTGCGATCAAGTAACTACAGAAATGAAATCATACGTTGAGAGTGTAAGTTTC
AAAGATTATGATTTGGAATTTGTTATAACAACAGATATTAGTTGTGTAAAACATGTAAGT
TCAAGTGTTATAGTTAGAAACGAATGTGAAAAAAAATATATATCTACAGGAAAAAAGATT
TTTGGGTTTAATAATAAAATAGATTGCTCAGCTGTAAAATTTAGTGAACATGTAAATTAT
TTAAAAACGTGCAGTGTTGGGAAATTTGATAGAAAAAAATATTATGAACATCAGCATAAT
TATATTAAAAAAATATTTCATCATAATGAACTATGA
>SPPV 02 00002 group 168
ATGTTTCCAATTCAAAAAGCTCTCATCTCCTTGTTTGGAAACATAAATCCGTTAGATTAT
GTCTTGGATAAGGATAAAAAAAAATCCAAAAGCAATGTAA
>SPPV 02 00127 group 170
ATGTTGTCGAGTAAATTAAGCAAAATATTGTATACATTATGTATACGTTTTAATATTGAA
TTAATATATAGAGTTTATAATTATGGAAGAACTTGA
>SPPV 02 00131 group 1
ATGAGATCACATTCATCATTTGAATTGAATACAAAACGTCAAGACTTTGATGTTTTGAGG
AAAGCAAATGCTATGGCGCTTGAACAAAAAAATTAATTGGACAATGTTAAAAGAAGAAGTA

TCATCTATAACTATAGAAAGATTTTTAAACTCAAACGTATTCGTTGATACAATTTTAGAA
TTTGGATTTCATGGTATATTACCAGTTAGTTTGTATAATAGAGCAATTGATTTTGATATT
AATAACTTAAAGTACTTTCCAGTTGAAAGTATACGTTTGGATGATTTAATAAATCTTTTA
TTGGAATCGAACACAATTGATAGTAAGTTACATAATGCCGTGTTGACATACAGAAAAGAT
TTGATTACGTCAAGGAATGGTGATAAGTTAATAAAATTAATGATTGAAAACGACTTGCTT
AATATCGATGACATAAACTACATAGTATCCGAATGCATCGTTCCTAACTCATATATATTA
AAGTTAAACCCAATGTTAGTATCCAACGCAATGATTTTATCAAAGGATGAATTAATTGAA
GTTATAAAATTTACACCATCATATACTTGGAAATATTTATATAAACACTTGTCAATAGAA
TTAGATACGTTGCTATATATATCTGATACGCTTAACGTACCACCAACAAATATTTCTTTG
TTAAAACTAAATAATATGAAAAAGGCTATAGAAATAATAAAAAAGTATCCTAATGATGAT
ATTATTACGTACGTATCTAGTGATATAAAATTAAATTTGCCTTTTATTGAGAAAATTCAT
GAAATAGTTAACGACAATTTCCCTAGGATGCTCACTCATATGAACGATTTCCTGTTTTCA
ACAGTTTCTAAAGAAGAACTTATTAAAAGATATGGAATTAAATCAGTGGCTATGTTTTCG
TTTAATTTAGAGCTTCATCCGGAAACAATATCTGACGATGATAAAAATTTTATATTAGAA
AATATTAATATATATGAATCAAAGTGTAAAAATTTTTTCGATTTCAAAAAAAAAATTTTT
TACAATCAATTAAAATTGTATAAACCCCCATCAATAATTAAATCTCCATCATTTAAAAAA
AATAATAACATTGACGATGAGGAAAAAACGATTGATGATGTACTAAATTATATAGATAAT
TCAAAAAGAAAAATTTTAATAACGGAAGTAGAATTTTTAATTTCACCATTTAAATTTAAT
CCGTGTATTGTTAGGAATATTTGTTTATCTAATTCTTCTATAAAAACTAAGCTGCTTCTT
CTAAAAATAATCAAAAAATGGAAATATTCTTATAACTTAACAAATAAATTTTTATTTGGT
ATACAAAGTTTTAAAAAAATCAATGATATTATTACTTTCGATATGACAAATTATATAACC
TCTGTAATATTATGCAAGTACAAAAAAATATATGACAGAATAAAAACATTAGAAAATTTA
GTTAATATGAACTTAATTTGCAATTGTGAAAATTGCAACAATAGTAAAATAAATCACAAA
AGTAAAAATATCTATCTTGGTAAAAAAACTTCAGATGATGATATCATTAACTGTTTATAT
GATTTAGTAAATTATGCTATTCATGGATTAGTAAATCCATCACTAATTGGAATTAAGGGA
TGGGGGGCATTAATTTGTTTACTTAATGGAACAAAAAAAAATAAAATAGATATTAATATT
TATAATACAATGGATTTTAATTTTGTATCCACCAATGGAGATATCTTACTATCTGGATCT
TACCTTGTAAATAAACTTATTCCAATAACAAAGACACCATTATCGTCATTTGTTCCATTT
ACATGTTTTTCACTAAATGATTATTTTTCAAAAACCGTTATTTTATTTAATATAATTTTA
GAATATATGACATCGTTTGTACTGTATCGAATTATTACAATTCAAAGATATAATAATTTA
AAGGAATATATTTCAAAAGTAGTTAATACATGTTTGGAAGCATCTGGTGTTTTCTTCGCA
CAAATAAATATAAGTAATATAATTGAAACAGAAATAAACGAAATCGTTATAAACGGTTCT
TTTCCAGTTCGTTTACTTAATATATTTTTGAAAGTGATTACTACTATATTAGAGGAAATA
AATGGAATTAGTTAA
>LSDV 01 00043 group 200
ATGGCGGATAATGACGATCAATTGGTATTAAATAGTATTAGTGCTAGAGCGTTGAAGATT
TTTTTTACTTCTAAAATTAATGATATTGTAGACGAATTAGTAACTAAAAAGTATCCACAA
AAAAAAAAGTCACTCGCTAAAAGAATAGAAACAAGGATTCCAATAGATTTAATAAGAGAA
GATTTCGTTAAGCGTTTTAAACTCGATAACTATAAAAATGGCATTCTATCTACGCTAATC
AATAGTTTAGTTGAAAATAATTACTTTTCTAAGGATGGTAAGTTAAATGATGATGCTATA
AATGAATTAGTTATAAATGATATTGAAAAAAAGATTTTAGAAAAAATATCTAAATCTTCT
TCTCTTTATATAGATGTAAGTGATGTGAAAATATTATCTTCCAGATTAAAAAAGCCTGCA
AGTTTTTTTATATTTAACAAAAATAGATATGTTTTAGAAAATGATAAAATAGAAGAACTA
ATAAACCAATTAGCTAAAAATAATGATATAACGTTAGATGAAAAATATTCTGTGAAAGAT
AATGTATATATCCTATCAGATGAACTATTGGAAGTTTTAAAATCTAGATTATTTAAATGT
CCCCAGGTTAAAGATAATTTAATATCAAAAACTAGGTTATATGATTATTTTAATAGGATA
ACCAAACAAGAAGAATCAAAAATTTATGTTATTTTAAAAGATAAGAAAATAGCAGATATT
TTAGGAATAGACACAATAAAAATTGGATCTTTTATTTATACTAAACATAGTATGTTAATG
AATTCTATTTCATCTAATGTGGATAGGTATTCTAAAAAGTTTCAAGAATCTTTTTATGAA
AAAATATCAGAATTTGTAAAAGACAACGAAAAAGTTAATGTATCTAAAGTTGTTGAGTGT
TTAATAACCCCAAATATTGTATTGGAAACAAAGTTAACAGAATAA
>LSDV 01 00044 group 121
ATGGATAAGTTGTATTCTGCAATATTTGGAGTTTTTATGTCATCGTCAGATGATGACTTT
AATAACTTTATAAATGTGGTCAAATCAGTTTTAAACGACGACGAAAATTATAACAAAAAG
ACGAAAAAATCATTTTTAAGATATTATTTAATTATTGGATTAGTTACGATTGTTTTTGTT
ATAATTTTTATCATTTTTTATTTAAAAGTTATAAAATGA
>LSDV 01 00045 I3L
ATGCGTAAAAGTATACAAAAGTCAGTGGTTGTTAAAAAAGAAACACCCGGTATGTCGACC
CAAAGTGATGAAAAAAAAGTATTCAAAATGTCACGAAGTAATAGAGTATTTAAAAACATTA
AGTAAAAGTAAAGAAAAATGTGTTGAAAGTGTTATATTGTCACAATCACAGTATCCCTCG
TGTTCATCTATAATGATTAACTTAACAGAAGCATTATCATCGAAAATGACATCTACATAT
ATAGCCGTAGAAGGAGAAGCAAAAATATATAAAAACAAAAAGGATGTAAAACTTGGAGAA
CAATACTTTTTAAAAATAAAACTTTCTGCAACTAGCCCTATTCTATATCAATTATTAGAA
AATATTTATACAAATATAAAAGAGGGTAAAAAGGTGCCACCGTCGTTGGTGAATATTTCA
ATAAATGATTTAGATGAAAAAACACTTAATAAGGGATGCTTATATATAAATAAAATGAAT
GGTGCTATCATTGAATATAAAGTTCCTGGCAACAGATCAATAGTTCAGTCTATGATAGAG
GAATTAGAAAGCCTTTCAAAAAGAGATAAACAAATGGCAAAAGCTATAATTGTACCAATT
GTTTTGTATAAAAATTCTGAAACAATAAAGGTTACATTTGCATTAAAAAAATTAATCATG
GAAAGAGACTTTAGTGCTAATATTGTTGATGTAGATGGTAAAAGTGAAAAATTTTCCATG
GCTGAAGTTTCCGAAGAAGATTCAGTTAGAGGGTTAGGCGTGATAGAACTTGAAGATGAA
TGTTTAGAAGAAGATGATAATAATCCAACATCAACATTATTTAATGTATAA
>LSDV 01 00046 group 147
ATGGCATTATCTACTAGAGAAACATTTTCTTTGATAACGGTTACACTATTGGCTATATTT
ATGATAGTTAGTGGCAGCGCGTTACTGTTTAAATCATTCGCTCCACATAGATTTATTATG
GCTAGATCATTAACGTTTGACAAAATATTAAGCTTTTTAGAGTACGCGGCTATATTTATT
TTTGTTCCAGGTACTGTTTCGTTATACTCTGCATATATAAAAACATTATTTAAATAA
>LSDV 01 00047 group 93
ATGAATAACTTTATAAAACACGTAAGTTACAAAATTTTAAAACCAAACAAAAAACTATCT
ACTACTCCAATTATTGCGGTTATTATGATTGCTGTAGCGTCTGCATTGCTACCATTCCCT
TCTTTAGTTATTGCGTATTGTTTATCAATACAAAAATTCAGCGGAGGGGTAGGTAATAAC
AAACAAATTATTATGTCTATTTTATGCGTGTCGGTTTCTATAATTACTTTGATTTTTAGA
CATATATCTAGTACAGCTTACACCATTTCATACATTATTCTAGCAATTTTATTTTGTGTT
TATGCGTTTAATATTTCAAAAATTTCTTATCAAGATCAAGATCAAGCGTGTTATAAAAAC
TTTAAAGGTGGTAACAAATACAGAGAGAAACCAAGTTTTTACGAAGAGTAA
>LSDV 01 00108 group 132
ATGTCTATGTATTATGAGATAGACTATAAACTATATAGTCAGATAAAAAGAATTATAGAC
GATCAACAATTATTTCTATTTAACGATAAAAAAGAGTTTGTAGAAGTTGAACCAAAATCT
ATGTTTAAATTTATATTACCTATTGGTTTATTCTCATCTGAAAGTTTTCCATTACCATCG
AAAATCTTTGCAGAGAAAAAGAATTACATTATTAATTTAGATGCAATAACTTTGCCTAAT
TTATATCCTCTTCAAAAAAAAGTTGTATTTAAAGTATTATCTTTAATGCGTGAAAAACGA
TTACAAGGAAGACCGATGTATATAACATTACATCTATCTTGTGGATTTGGAAAAACAATA
ACCGCTTGTTATTTAATGTTGAAACATAAGAGAAAAACTGTAATTTGTTTACCTAATAAG
ATGTTAGCATCTCAATGGAAAACAGTTATTGAATCCACTAAGCTGAGTTATATAATTTCC
TTAGATGGTGTTAAAAAGTTAATGGATAAGTTAAAATTTGAAAACGTTGACGTGTTGATA
ATTATTAATAGGCATTTATCTAATGAAGATTTTTGCAAAAAAATATATAGCGATTATGAT
ACTTTTATATTAGACGAATCACATATGTATAATTTAATGAACAATTCTATACTAACTAGA
TTTTTAACATTTTATCCACCAAAAAATATGCTATTTTTTAACAGCTACTCCTAGAAAATAT
AACAGGCTTTATTGTAATGAAATCGTTAACATTTTAAAAATTACTGATATGGTAAAGACA
ATTAAAGTTGTTGAATACTTTTTTGAAAATTATTCGTCTGTATCTATACGTAAAATGTTA
AAGAGAAAACTTGACAATGATAAGTATCATATATACACTGAAAAAATACTATCAGAAGAT
TCTCCAAGAAATGAACTAATATTAAAAACAATTTTTGAAAATTTTAAAAATGATATCATA
AAAAGAACTATAGTTGTAACAAAACTAAGATCGCATATGATGTTATTATATAGCGAGCTA
AATAAAATTTTGGGAAACGAGTTGGTATTTTTAGGGGATGCAAAAAACAAAAGCTCATAT
GACGTAGTAAAACGTATAAGGGAAAAAGAAAAATTTATATTTGTTTCAACTATTAATTAT
TCTGGTACTGGATTAGATATACCAACATTAGACTCTCTAATTTTGTGCAACGTAGTATTA
AACACCATTCAAGTTGAACAACTATTTGGAAGAATATGTAGAGAAAGTGACCATCAAAAA
CGAATCGTTTTTCTATTTCCGACTACGTCAATAAAAGAAATTAGACACTTAGTTGGGTTT
TTTACACAAAAAATAATATCGTTTTCAATGGAAAAATTAGGATTTGAAAAAGAGATAGAA
GGAAACGATAGAGGAAAAAAAGAACTTGCGTTATGTAAAGCACTTAATTTACAAACCCAT
TAA
>LSDV 01 00109 group 133
ATGGAAGATAGTGCGGGTAATAAACGCAGAAAGAAACGAAAACCAAAAACAACTGTACAA
GATGAAGAATGCATGACATGCTCATCGTGTTATTCAAAATTAGTAAAAGTTTCAGATATA
ACAAAGGTATCATTAAATGAATACAAAGTTGGTGGAAAGGGAAATATATTAACATGTTCT
GCATGTGGGTCTGAACTTAGAATGCTTAATGGGTTTGTAAATTAA
>LSDV 01 00110 group 101
ATGTTACTTGATAATAATAAACAAAAACCTCTAAGGTGTTCGGAAATAAAAAAAGCAAAT
AAAAATAATTATTTATCGTGTGAAAATAATGATAATGATATAGTAAACTTTTCGAGATCC
TGTTCAAATGCTTACTTAAATTTATTTTTTACTACATAA
>LSDV 01 00111 group 158
ATGACTACAGAAAGCGATTTAAAAAACTTAAAAGAGTTACTAAAATTAAAGAAATGTATA
AATATAGCAGACCAAAAAACAAGGGAAAAATATAATTCTATCGTTGAATGGGCATCGAAT
AAGTTTTGGAAAATAAACACTCATAAGGTTATCGATGCCGAAACTTCTATTAGTGATTAT
TACAATACTTCAAAAAACGAATCATTTATTTTAAAAAATGGTAAATATGTATTTTTACCC
ATGTGTTTTGGTAATATATTTATTTATTTTAAAGGAACTATGATGGAACTCGGATCTGGA
AATATACAATATATTGATATTAAAATTAAAAAAAAGATAGATAAGTTATTAAAAGAAAAC
TTAGATATAGAATTTCTTAGATTTGTGTACTTTAAAGGAGAGTGGATATTAGAAGATTGT
TTTTCAAAATGTAAATGCCCAATAAATTTTTTAAAAACGGCATCAACTAATGGTCTAAAC
ACTGTTCCATACATATTATTAAATACTAAAGAAATAATATTTACAGAAGATGATTATGAG
AATATAGATTCTTATTTTACATTTTATATAAAAGAACCTTTTAAAATTAGTTCGTTGTGT
TATATAGAAGAAGGGAACAACGAAAGAAATATAATAGATTTCTATAAAAAAACATATGTT
TTTGTCGAATCTATAAAAATAGAAAATATACACAACAATTATTATATTCCTAAAATAGTG
ACAAAATCAAAAAATGTGGTGTTAGTAAGGGATATAAATCACTTAATAGACTCTAAAATA
AAAATAAATTCATTCATAAGCGTTAAGAAAAAAAAAATACTTTTTAATACTAGACAATAAT
TCTATTTTTTCATTAGAGACACCCTCAGAAGCATTTTGTAGAATTATGAAATATATAGGA
AATGAATTTTATGTTAACGGTAAATATCTATCAAAGGTTAGTAATGATATAGAAATAAAA
CATTTATCTAGTAAATTGGGATTATTATCATCCACATTAAGTGAACTTATTACTGAGATT
AGCCTGTGTGATAAATTAAGGGAAAAAATAAAAAATCAATCAGTGTTTGAATTTGTAATG
GAATGTTTGGAATATCCAAAGGCAGATTTTCTAACATTAGTTAATAATATGTCATTTGAT
GTAAAAAATGGTATTGTTTTAAATTTTAAGTTAGAAAATATAAATTGTTTAGATAATCCA
AATATATCTGCAATATATGGTAACTTTAATAAGTTTATATCATTATTTAATATTTTTATA
GATGTCAAACAATCATTGTTTAAAGCATATTAA
>LSDV 01 00112 group 114
ATGTCAAACAATCATTGTTTAAAGCATATTAAAAAAGAGGTTATTTGTGCATTTGATATA
GGTGCTAAAAATCCAGCTAGGACAGTATTAGAAATAGAAAGTAATAATATAAAAATAATT
GATATATCGAAATTAGATTGGTCATGTAAATGGGAAGAACAAGTTGCTAAAGATTTATTT
CAACATAACTATAATTATGTGCTATTGGAACGACAATCAAAAAGATCGCCATATATTAAA
TTTATCCATTTTATTAAAGGGTTGTTATACAATTCTGATACAAAAGTAATATCAGTATCG
CCGGTAATGGTTGGTTACTCATATAAAGATAGAAAAAAAAAATCTACAGAATCTTTTTTA
AATTGGATGAAAGTATTTGGATTAGTAAATTCTATCCCAAAGGGAAAAAAAATAGATGAT
GTAGCAGATAGTTTTAATATCGCACTAAGGTTTGTTTTAGATAAATGGGATATTAATTAT
ATTCCGTATAAAAAAATAAAAAAATAG
>LSDV 01 00113 VITF3L
ATGGATGAACTATTTACATTTTTACATAAAATAGAGAATGAGTATTCTAGAACTATATTT
AATTTTCACCTTATAAGTTATAAAGACAAGTACAAAATATATGATATAATGAAAGAAAAA
ATATCAATCGAAAACATTTTTAACAATATAGCATTAACTGATGAAATTAAGAATCATATT
AATGATCTTTTTAAAAAAAATTGTACTGATTCTGATAATACAATTTATAACGGCATATTG
TCTGAATCCATAACTTTTGAAATAAATCATGGATTTTTTAATGTGGATACTGAAGTCAGT
ATATCAAAATCATTATTATATATGGATTTAGGAGTAGAAAATTATAGTGATGTACTAGAA
AGTTATAACATTACAATATCAAACATGACAACGATGACCACACCGACTCCCGCTACAGTA
ATAACAACACCGTTATATTTAGAAAATAAAAATAACACCAATGATATTAAAGTAAAGCTT
TACATGGTTAATTCAAGTATTATTCTAACATTTGAAAATATAACAATTGTTATAAATAAT
ACATGTATTGGTACATCCATAAACAGTATTTATGCAAAAATAACAAGTGATTTTATAACT
ATTAATGTCACAACAGATCCGTTGTCTACAACACCTCCATTTTTAACTAAGTCTATGTTT
GATAATTGTACCTTAACACTTCCTGTTACTATTTCTCAAGAAGGATATTATACAAAAGAA
TATAATGAACATACTGGTACAACTGAATCATTTACATATAGTTATTATTCAGATAATAAT
ATGACAGAGTCATTTACTACTGCATTTAGTTATTATTCGGATGATACCACTACTGAACCA
TTAACTACATCTGATTATTTAAATTATACCACATTGGAACCATCATTTACTACATCTAGA
TACTATTTAAACAATGGTACATCTAATTATAAGAATATTTCATATGTATACAATAACAAA
ACATGTATCGTATTTGTAAAACAACAAAATAACATTTTTAAAAATATAACAATACAAACA
GAGTTTAAAAATTTATGTAACGAATCAAGTTTAGAAACAAAGGTATATGCTGTAGGGGTT
CCAGAAAAGTTTAATACAATTTTAAAGAATATTAGTGTTGAAAAATCTAATGACAGTGAT
TCATATTTTATTTGTAAAATGATTGAAGATGGAGGGAATTGCGAAATTGATATATTCATG
GATGCTGCAACCAATAATATTAATGATGAACTGGATAGTTCTAAAAAAGGAAGTAATACC
AGACACGCTAGATCTGTTGATCATATAGAGTTAGATCCATTCTGTTTGCATATGTATCAT
GGCATTGACGAAACCATAGATTGTTCACTAAAGAAAAACAACAAAGATATGAGTAAACAC
ACAAACAGAATCAGGAGATCTCCTCCTGATAAAGGTAAAAAACCACAAGTACCGACAAAA
GGAAATTTACAACTAATGAGCGCTGAGGAAATGGGAGCTAGACCAAAAATAAGAAAACAA
ACGCAAGATGTACAAATTGGTGCTAGAGGGGTTGATGGTCCAGTTTCTGGATCAGAAGAA
ATATATTCTCAAGTTAGGAAAGAACTATCTCAGAAGTTAAAATCTTTAGTCTTGGACGAT
GATGGAAAATTGACAACAACAAGATTGCCTAAATCAACAAAAGCTTTAATTGGTGGGTTG
GTAGATTCTAAGATAGAGACATCTAGTGCTGCGTATGATATAACAAGACAAATAGTTCAT
CAACAATCAGGAGATATTTATTCGTTGCCTGTAAAGAATAAAGTATTCGGTGTAGAATCA
AATAGAAAGATAACATCTCCAATTATAGACGTTGATACATCATCTAATATATACGCAAAC
GTTTTACAAAAAAGAGATTTTAAAATTACGTCTCCAAAAAAATGTTGATGTATATGAAAAA
ACTAGAACTGATTCTTTTTCAGACGATTCGTATTTTTTGAAAAGATCATTTTCTTCTAGT
TCCGACGATACTTACTTTTTAAAAGAATCAGATGACAGCGATAGTGACATTTTTAGAAGA
TCTTCAAAAAAGGATCAAACTTATTCACTAGCAGGAAAACCATTAAAAAAACAATCACTT
AGAAGGTATTCATCTAGTGATTATGAAACAATAGGAGAAAATATATATGAATCTATTCGT
GAACCAGAATATGCACTGCTTTCAAAACCAAGAGTTTTAAATCCACGAAGTCATATACCT
ATACCATCATTACCTAAGGATGAAATACCATTTACTCAACAAAAAAGAAAGGTGATTGAC
ATGATTTGTGACTCAAGATTTGCATCCTCTATATGTAATGCAAGAGGGTTGGATTTAGCA
AATTATAGAGGTGATAGTAATATATATAAAACAACTGACGACGATTTTGTAAAACGTGAA
AATTCTTTATATGCAAGATCAAAACTAGAACCAGAATTAAAGGATAATCCATTGTATGAA
TCATTGTCTGATATCGATATTGTATCAAATCCATATAACAATCCTAAATTGTCGAGAAGA
AATGCTATTAAAAAGAAAGTATTAAACGATGGTTATGATGAATTCGTTATAAGGACCGAC
GAAGAGCCTAGTGAAAAACCTAATATGGCAACAAATACGTATAATAACAATGATAAAGCT
AATAATAAAGATAAAAATAAAGGATTTTCTTACTTAAATAATGATATACAAGAAGATGAA
AAAAATGTTAATAAAATTAAAAAATCAAAAAAAGGAAGAACTAAACGTTTAAGTAAATTA
TCCACTGATAATAAAATAAATAATATGATAAAAACAATTGCTATTTCCAGTTATTTATCA
TCAACGAATAGTAGGATATCGTCCATAATGGCACAGGCTAATAACCAACCAAAAGAATTA
ACAATTGTTAACATTGTAACGTCCGTATTATCACAAATCGGTGGAACTTTAGCAATGGCT
GGATCTGGGTCTCCAAAAGCTGCAGCTGCAGGTTTAGTTATTCAAGGAATATCTGGATTA
ATAGATGCAGCAACATCAATTTATTTTCTTCTTTCTGGTCAAGAACAACCTAAAGATCCA
GCTATTGAAAAGTTTTCAAACTACGCATCTTACGTTTCAAAAACCGATGCAGGATCAAGG
GTTTGTATGATGCCAGATTCAGATATTACAATAACGTTAGCATATAGACATAGTAACATG
AATGTAGAAGCTGAAAAAACTAGAGGTGAGTACACAGATATAATTCCAAGTACAGTATAC
TACTTAAAAAACAGTCAAATAAGTTATACGGTGAAAGTAACATTGGTATGTCCAATCGGT
CAGTTGAGGCTTTTGGAAGCAGATATAAATACATATGCTACACTTACTAGGGAAGATAAA
GATGGTGTAAAATTCTATCATGTGTTTGGGATTTTAGAACTCCTATCATATCATCCTAAT
GTTACATTTACTTGTGGAAATGAACCAGGTGTAATCTTCATCCCGTTTGAACAAAAACTA
AGCGACATGCAATTATTAAGGATATCAACTCCTGGTGAACCTAGAGAAGCTGAAGCAATG
AGTTCTGATGTTTGTGATATTTATCCTCTTAAGAAGTTTTATGTATTAGCTGGAAACTGC
CCTTTTGATATGAGCAGAAAATCAGTAGCTTATGTTACATGTAGTACGTTACTAAGAATG
TCAACGTATGAACATGAAAAACAGAGGTGGATTTTAATGAATCCGTTTTCAAACAACAAT
GAGGACAATATTCAATTGTTTACGTTTAAAAAATATGACTTTAGTGCAGAAAAAGATAAA
ATAAAACTAAATAGTATTTCACATAGTGATACTATCTGTAGCCAATCTGATACTAGTACA
TGTTTTTGGGCAGATGCTATGATTTTGGAAGATGTAACATCTTGCAATTCTAGGATAAGA
AAATTGTCAGTTGAGATGTCTACAGTATCAGAAAAAGGATATAACAATTTTGTACTTACG
TGTCCATATGGGTCTACACCGTTTTATATAAGTAATGGTTCAATAATAAGTATTCCAATG
AACACAAGAAGGACGTCTGTTAGATTTGCTTCTAAACATAACAATGTTGCACTAATATCA
TGTATACATAATTCAAACCCTGCATATAAATCTGATATCGTTGAAGTTATGTTTAAACAA
TCAAATATGAGTGATATGTATTTGGATTTCAAATATTTTAAAGATAGAAAATATTTATTT
GATTCATTTAGTGATATAATGCCTAAAAGATCAAAAACTTGTAAGAGAGCATCAGAAAAT
AGCAATTGTAAAAACTATTATTATATTAAACATGTTCCTGAAATAGAGTTTAAAGTACAT
GTTGCAAAACTTCCAATGGTTAGATTAGGCACAAGTTATTCAGGTGTTTTGAACTATCAA
ACATTAGAAAAAGTTAATAAATACTTTTCAACACCTATAAGTATTTCTGTAGATGCTAGT
ACTTTATCCAATGTATACAAAAACCAAGAACATTTTTGGAAATTTGCAATGGAAAAGAAA
AGAACTTTTAGTTCTATTACAGCTACAATATTTGCGTGCTCTGTTGTAGCTGGGAAGGTA
AATGTAAACATGGGAGTTAAAGGTTCACACGATTATTATGGAAGAAGTGGTAAATATATT
TACATGGGATCAAAAGATTTTGCATCTAATGATAAAATATATTTCAATTTTATACCTGAC
AAAGCAGAGTACCGTTTAAAGGATACATATGGAGAATGTGAAATTTATTTAGATTTAAAA
ACTAGGAGAGTTAATGTAAACTGTCCGGAATTGACTATACCACAACATCCATTTGATTCA
CTAGATGTTAATAGTTTATGTGTGTTAGTTGCCACATCAAGAGATCATTGTGCTATATCT

AAAAAACTCGATGATGAAATAAAACTAAAAGAATGTATAATATCATTTAATTTTGAAAAT
TTTTATTATTCCAATGAAATTTATTCAATAAACCTACCAATTCTTTAGATGATGTAAAC
AAATCATGGTTAATAATGGAATCCTTTAAGTATGAAAAATATGTTATTATGGGACTGATA
AATATTTTAAAAAAAAAATCTTATATAACGGATTTTTTTTCATTCCCATAGGATGGATT
GTTGGCAATAATATTAACAACGATGATAATATAAATAATAATCACGTAGTTATAAAAATA
ACGATTGAAAATAATAAACCATTTCAACCATTAAGTAATAAAATAAAAGATTTTTTATCC
CATTATGATATTTTCGATTTACTAATAATACAAAAAGAAAAGGAACTTAATATTTTAAGT
TTTAAAACGCCTAATAACTTTCCTTCGGTTGTTATATCTTTATTTCCGTTTGATGTAACG
TCAATTATAATAGTATTATTTTTTGGTATATTTGATGATTCGTATTGTGGACTTTCTTAT
ATAACTAATAAAGATAACTTAAACTATGTGATCGAAGTATTAAAACCAATATCTTCGGAA
ATTAATATTTTATCTGACGAAATTACTAGATTCGTATCAATAAAACTTTTTAATAATGAG
TTAAATAAAAATAAAAAATTCCCTGAAGAAAAAATTTTTTTTATTTGTGAATTTATAAAA
ATTTTTGAAAAAACAAAATTTGAAGAACCTAAATCAGTTATTCAGACGAACATAACGACT
TTTATTCCTAAAAAAATGATATCATTAATAGACTTACCATCTAATGTTGAAATACGAACG
TTGTCAAAAAATGGAATAGATTATATAACACATATCAACGATAAAAAATTATCAACAATT
TTGATCATAACAAAAGATAATTTTTTAAAAAATGTTTCATTTTCTGGCACGTTTAAAAAA
GAAAATATTATATGGAAAGGATACTATACATACAGAATAATAGAATCGACGTTTGATGTG
CCTAAATTAAAAATATCACCGAATGGTAAAAAAAGATACATAACTTGTAAAAGTGCCTTT
AATAATTCAACTTTTACAACTAGGAGTGGACAATATATTGTTTAG
>LSDV 01 00048 group 192
ATGGATAGATATACAGATTTAGTTGTAAATAAGATTCCGGAGTTAGGATTTACTAATTTA
TTATCATATATATATTCTGAATCTGGGTTATGCTTTAATCTAGATATTTCAAAATTTTTA
ACCAATTGTAATGGATACGTTGTAGAAAAATATGATAAGTCAGTAACTGCGGGAAAGGAA
TCATGTATACCATTGTTTTTATTATTAGAATTAGTCGATTTAAAATATTTAGAAAATAAT
TATCAAAAAGATAAAGATAATGTAATTAACAATAATGAATATGAATTATCATTAAAGAAG
TATTTAGTTAATCAATTAAAAAACAAATATAAAAACATTCAAGAAATTTTCAATTTTCCG
ACTTCTATACCAATTGAATATTTTTTTAAACCTAAACTTAAAGAAAAGATTTCGAAGGCG
ATTGATTTTTCACAAATGGATATAAAAGTTGATGATTTATCTAAAAGGGGATTTTTAGCG
GGAGAAAATAGCAAAATAGTAAAGATAAAAAATCGAACCTGATAAACGGGCATGGATGAGT
AATAAAAGTATTCAAAGTTTAGTTTCGCAATTTTCATATGGAACGGATGTTGATTATTTA
GGACAATACGATATGAGATTTTTAAATAATGTTCCAATTTATGAAAAGTTTGATTTTTTT
ATAAACAAGCATATTTTATCATACATCTTAAAAGATAAAATAAGAAAATCCAAATATAGA
TATGCTATGTTTGGATTTTGTTACTTGTCTCATTGGAAATGTGTAATTTTTGATAAAGAA
AAATTAGTTGTTTGTTTTTATGATTCTGGAGGAAACATACCAAGTGAATTTCATCATTAT
GACAATTTTTATTTTTATTCATTTTCTGATGGCTTTAATACAAATGACAAAGATGATTCA
GTTTTAGAGAATGGTAATGGTGATGTAGATGTTTTGTTTAGATTTTTCAAATATTCATTT
GGAGCAAAGGTAGGTTGTATAAATGTAGAAGTTAACCAACTTTTAGAATCCGAATGTGGA
ATGTTCATAAGTATATTTATGATAATTTGTACACAAACACCACCGAAAGGATTTAAATCA
ATAAGAAAAATATATACATTTTTTAAGTTTTTAGCAGATAAAAAGATGACTTTATTTAAA
AGTATTTTGTTTAATATAGGTGAAATAAATATAGAAACTGAGTATTTAGAATGTCCCGCA
TTGAAAGAATATAAAAGAATGGAGGCGTGGACAAAAAAGGCAATAACTGCTTTATACAAT
AAAATAGTGATAAAAGCAAATAAAATAGTTAATAATGAATAA
>LSDV 01 00049 NPH2
ATGGATAAGCTACCAAATATATATTATTTCCCAAACTGTGTAAATTTATTTCCCTATCAA
TACTCTCAAAACGAGTTTGAAAACATGTCAAATGATGAAAAGAAAAATTTTTCTTTTGCT
ATATTTCCTTTAATAAAACATAGATGGAAGAAATCATATGTAGTAAAAGATAATAAAACG
TATAAACTAAGTAATGAGGTCAACAAAACACTTAAAAAAGTTACTAATGTGAATATTCCA
TCTATTTATCCAACAAAAATAGATGGCGCGTCAAAAGAATACACAATAAATAATATAAAA
ATTAGTTTTGAATGCTATAGCTATCTAAATTATTCTAAGTTCGATAATATCCACTCATTT
GATGAGTATATTTTAAGAGGGTTATTGGAGGGAGGAAATAAACTTAAAATATTTTCCAAT
AATATAGGTTCTGCCAACTCAACCATTGGTATTTTTAATAACACAGAACCATTTTTTAAA
ATTAAGTTGGCATCTTTGCAACCAAAATCACAGCAGGAAATATTTAAATCTTGGATATCC
CATAAACCAATAATATTGACAGGTGGTACAGGTGTAGGTAAAACGTCACAAGTACCTAAA
CTAATATTATGGTTTAATTATTTATTTGGTGGGTTTAAAAATTTATCCAATGATTATACA
TATATAGAAAAACCAATTGTGTTATCATTACCCAGAATTACTTTGGTAAAGTTACATAGT
AAAAATTTACTTAACTCTCTAGGTTTCAAATCACTAAACGAATCACCAATTTCACTGAAA
TTTGGATCTATAACTAAAGATCTAATAAACCTCAATCAAAGGAACTATGGCATTGTTTTT
TCAACGCATAGAATAACACTGTCTAAACTTTTTGATTATGGCACTATCATATTAGATGAA
GTGCATGAACACGATCAAATAGGAGATATAATAATATCTGTTACTAGAAAATATATTAAT
AAAATAGAATCATTGTTTTTAATGACAGCGACACTAGAGGACGATATTGATCGTATAAAA
GTTTTTTTTGAAAGTCCAATATTTGTTCACATACCAGGTAGTACACTATTTAACATATCT
GAAGTTTATATAAAAAATAGTGTAAATATAAAAAATAAATTTAAATATATTGAAGAAGAA
AAGAAAAACATGTTAAATGCCATAAAGAAATATACACCGCCAAAGAACTCATCTGGGATA
GTTTTTGTTTCATCTATCGCACAGTGTGAAAACTATAAGAAGTTTTTATCTAAACATATA
CCATATGATATACATATAATTCATGGAAAAGTAAAAAATATAGAAGATATATTGTCAGAT
ATTTATTCTACAAATAATACATCAATAATTATATCTACTCCATATTTAGAATCAAGCGTG
ACGATAAATAATGTAACCCATATTTATGATAGTGGAAGAGTTTACATTCCAGAACCATAT
GGTGGTAAAGAAATTTTTATTTCTAAATCTATGCGGGAACAAAGAAAAGGCCGTGTTGGT
CGAGTGAAGCCTGGAACATATATCTACTTTTACGATATCGCGTATTTAAAACCAATAAAG
CGTATAGATTCAGAATTTTTACATAATTACATTTTATATGGAAAAATATTTAACTTATCA
TTACCAAACGATTTATTTGTGCAACCATCTAATTTAGATTTTTTAAACAAAGTAAGTACT
TATATAGACTCATTTTGTATCAGTGATGAAAAATGGACATTTATTATGTCTTCTTATTAT
GTTAATATTTTAGAATATGCAAAAATATACTCTAAGGGTGGAAACGATGCATGTGCGTTG
GATATTTTTGAAAGAGAAAATGTTTTAAATGATGATGCATTTAACGCAATAAAGTTGTTG
AATATGAAAGCAAAAATAATAAAGCACAGAAGATATAATAAATTATATGTATTAACGTGT
AAATTAATGTTTGGTGTATATTTTGGTAAAACATTTAACATTATACATAATCGACCATTA
TTTGGTTATATTACTATGATAACAGATAGTACTTTTGTAACTGATGATTAA
>LSDV 01 00050 group 211
AAAAAGTTGGTTTATTGTGATATCCATTTAACAAAGCATATTATAAACAATACAGTTTAT
CCTAAATATGATAATTTAAATCAAAACAATAATTTAAAATTTTCTCAATTTTTTGATATC
AATAAAGATGATGACTATATTAGTTTAAGAACTGCTGAAATATTTGATAATGATAAATCA
TCATTAATATCTTATATAAAAACCACCAATAAAAAAAAAAAAGTTGATTATGGTGAGATA
AAAAAAACAGTGCATGGGAACACAAAAAATTATTCAAATTATTTTTCCGGCAAAAAATCT
GACGATTATCTTGTTACGACGATAAATACTAAAGAATCACAGCCATGGATAAAAACAATT
TCTAAGCGTATGCGAATAGATATTAAAAAAAATGCAATCATAACAAAGGGAAAAAGTTCA
ATTTTACAAACAATAGAAATAGTATTTGTAAATAGAACATGTATAAAAATTTTTAAAGAT
TCGACGATGCATATTATATTATCAAAAGAAAAGGATGAAATTGGTTGTGTTGGAATTATA
AATAAAATATTTCAAGTATATAAAATATTGTTTTGTTTACTTTACGATATTTCAAAAAAT
GAAAATTTTAAAAATGCATTTGATAATTCTGTATCTATTTTAAACTTAACTAAGTTTGAT
GATAAGATTGCAAAAATAAAAACAGTAAAAAATGAATACGGGTTTGAAAACTTTAAAATT
GGAATGTTTAATTTAACATATAACAAACCTATTTTGCATACAGTATTTCCGTCGTTGTTA
GATTACGAAAACAAAATAAAATTTTTTAAAGGAAAAAAACTAAATATAGTTGCTCTTAGA
TCATTAGATGAATGTATTCAATATGTGTCGTTAGCAAATAAAATATTAAAAAAGATGGCA
GATCGGTCCGATATTCTAAATAATTTAAATATAGAATCTGAAAGTATAGAAATACTAAAA
AAAATATTGTTTAATTAA
>LSDV 01 00114 rpoB
ATGGACCAAAGATTAGGATACAAGTTTCTTTCTCCTGATCCAAAAATCGGAGTATTTTAT
AGACCGTTACATTTTCAATATAAATCGTATTCAAACTTTATCAATTACAGATTACAAGAA
ATTTTATCGGTTAAAAGAACTCTATTGTCGTTTAAAAATGATACTGAAAAGATTATAATC
GAAATTGATAATATAAAAGTGACGCCACCAGAGTATTCACCTATTATAGCTAGCATAAAA
GGAAAGAGTTATGATGCATTAGTAACATTTACTGTGCACATTTATAAGGAAGTAATGACA
AAAGATGGAACTACTATAACCAAAATTAGTAGTTATGAAGGTAACGATTCTCATTTAATT
AAAATACCATTATTAATAGGTTATGGAAATAAAAACCCATTGGATACAGCAAAATATGTT
GTTCCAAATGTTATCGGTGGTGTATTTATCAATAAACAATCCGTTGAAAAAGTAGGAATA
AATTTAGTAGAAAAATTAACAACATGGCCAAAATTTAGAGTTATTAAACCAAATGCGTTT
ACTTTTTCTTTTTCATCAGTTTCACCGGCACATATACTACCGACTAAGTATAGGCATTAT
AAAATACTAATGGACTTATCTCAATTAGATAATTGTTATATTTCATCGACTAAAACATTC
ATAACCGTTAATATAATTTTATTGGTTCAATATTTATCTAGAGTAAGTTTAAGCTTTATA
AAACACAGTTTATCTTATGACATGCCTTCAGAAATTTCGTATTTGGTTAACACAATAATA
GAAAGTACAAAAGAATTGATAAAAACGATAAACGATTTTGACATTGACACATACATTAAT
GATTTAATAATATCTGAATATAATAAACAAAAATCGCAATTAATTTTAGAAGAATTTAAA
CATGAAATGATAAATAATTTTCTACCACATATGAATGACACACCTAACCAACTAAAAGGT
TTTTATATTATGTCATTATTAAGAAAATTTATTTATTGCATTTACTACACGTCTAGATAT
CCTGATCGTGATTCGATGGTTTGTCATAGAGTTTTAACATATGGAAAATACTTTGAAATT
TTAGCACATGACGAGTTAGAAAATTACATAGGAAATATACGAACAGACATTATCAATAAT
CATAAAAATAGAGGAACTTATTCAGTAAACATACATGTATTAACAACACCGGGATTTAAT
CATGCGTTTTCTGGATTATTAAGTGGAAAGTTCAAAAAAACAGACGGTAGTTACAGGACG
CACTCACATTATTCTTGGATGCAAAATATATCTATTCCAAGAAGTGTCGGATATTACCCG
GATCAGGTTAAAATTTCTAAAATGTTTTCAGTAAGAAAATATCATCCAAGTCAATATGCA
TACTTTTGTCCGTCAGATGTTCCTGAAAGAGGTCCACAAGTAGGTCTAGTGTCACAATTA
TCTGTTCTTACATCTATTACCAATATATGCACGAATGAATATTTAGAATTAGAAAAAAAA
ATTTGTAATTATATACGATCATATAACCATAACGATATAAGTTATTTTGAAACTGGGTAT
TATATAACATTAGAAAATTCATTAATTGCTTGTCTTAATCCAAATTTGGTCGATGATTTT
GTTATTGATTTTAGAAGAAAAAAAAGAATGAACTATTTTGGCAACTTAGAAATAGGAATT
ACGTTGGTTAATGATCATATGAACGAAATTAGAATCAACATAGGAGGAGGTAGATTAATT
AGACCATTTTTAGTTATTGATAATGGTAATCTTATCATGGATGAAATATTTTCAGAACTC
GAATTTAAAATAGATGATATGACATTTTCTGATATACAAAAAGAATTTCCACATGTTATT
GAAATAGTTGACATTGAACAATTTACATTTAGCAATGTATGTGAATCAGTGCAAAAATTT
AGAGCTCTTCCCAAATCTGAAAAATGTAAATATCATTTGTGTGACTTTCCTGCCGAATTT
AAAGATGGGTATGTTGCATCTTCACTGGTTGGTATAAATCATAATTCTGGCCCTAGAGCT
ATTTTGGGATGCGCGCAAGCAAAGCAGGCCATATCTTGTTTAAGTTCAGATATACGAAAT
AAAATAGATAATGGTATCCATTTGATTTATCCAGAAAGACCTATTGTTATTAGCAAAGCA
TTAGAAACATCTAAAATCGCGGTAAATTGCTTCGGGCAACATGTTACTATTGCATTAATG
TCTTATAAAGGAATAAATCAAGAAGATGGTATTATTATTAAAAAACAGTTTGTTGAACGC
GGAGGTTTAGACATAATAACTGCTAAAAAACATCAGGTAGAAATTCCATTAGAGAATTTT
AATAATAAAGAAAGAGTTAAATCTACAGCTTACTCAAAGTTAGAAAGTAATGGCTTAGTA
AGACTAAATGCGTTTTTAGAATCAGGAGACGCTATTGCCAGAAACATATCGTCGCGAACA
TTAGAGGATGATTTTGTTCAAGATAACCAAATTAGTTTTGACATATCTGATAGATATACT
GATATGTACCAATCACGGGTTGAGAGAGTTCAGGTAGATCTAACGGACAAAGTAAAAGTT
AGGGTTTTGACAATGAAAGAAAGACGGCCAGTATTAGGTGATAAATTTACTAGTAGAACT
AGTCAAAAAGGAACAGTAGCGTATATAGCAGATGAAACAGAATTACCATATGATGAAAAT
GGAATTAAACCAGATGTTATAATTAATTCTACATCTATATTTTCAAGAAAAACGGTTTCT
ATGTTAATTGAAGTTATATTAACATCAGCGTACGCAAGTAAGCCATATAATAATGATGGT
CAAAACAGACCAATATGCTTCCCTAGTAGCAATGAAACCAGCATCGATACATATTTGGAT
TTTGCCAAAAGATGTCACAGAGATAGATATCCAAGTTTATCAGACGATGATATAAATGAT
AAAATGTTTTGTGATACAATTTTATATGATCCCGAAACAGATAAACCGTATTCGTCAAAA
ATATTTATGGGTCCGATATATTATCTTAGATTACGTCACCTAACTCAGGATAAGGCAACA
GTTCGATGCCGAGGCAAAAAAACAAAGCTAATACGTCAAGCTAACGAAGGGCGTCGACGA
GGCGGCGGTATTAAATTTGGAGAAATGGAAAGAGATTGTTTAATCGCACACGGTGCAGCA
AATACGATAACAGAAGTATTAAAAGATTCGGAAGAGGACTATCAAGATGTGTATGTCTGT
GAAAACTGTGGAGATATAACAGCACAGATACAAGGAAATAAAGTTTGTATTAGGTGCTCT
AAACAAAATTTATCTACTATACTAACCAAAGTTGATACAACACATGTGGCAAAGGTTTTC
ATAACTCAAATGAATGCAAGGGGTGTGAAGGTTAAACTGGAGTTTGAAAAACGGAACCCA
TTATTTTATAAACCTCTGGATGTGGTAGATTTAAGTCCTAATTTTTTGTAA
>LSDV 01 00115 group 104
ATGGACAGAGCTTTATCAATCTTTCCAGGCGATGATGATGAAACCAATGAAAGAAATATA
GAGGAAAATTGGAGAAGTTATGATAGAACTCATGGTGTTGGGTATAGCCACGAATACGTA
GATGCGGAGTTTGATTCTTGTAGTAAGAACCATGGTCCAACATATCCAGTTGATAACTTT
TGCTTTTATTGGTCTGCGGGGATATATTGGCCACCTGACTATGACCCATGTGCTTCATCA
ATGGTTTTAGGTTATTCTCCAATTTTTACAGAAAATAGAATTGTAAACCCTCCATATATA
AAGGAATTTGGTTATGAACCAGAAAAAAATGAATACGTGAAAAGAGAGTTATATAACAAA
TTACAAAATCTCTATGAAAAATATAATATGTTGGTTTTATATTCAATGAACCCAGTTGTT
GAAATGTCTAACGGATTAGCAAAATCTATGACTTCAGAAGCCCGTGAGATTTTTAGGTTA
ATAGCAAATAGTAACGAAATGCAAAAAGCTAAAGAAGAAAATGATATGAAAGCTGAGAAA
GTTAAAATGGAAATAGAAGAAACATTAAATAATATTTACGTTGATACATTATCATACTCT
GAATCAACTTCCCTTTTACGGTCTGCTATTTCCACAAGGTGTTGTGTTTTGGACGGTACT
AGTGTATATAAGTATTTTGATTTAGAATATTATCTTTGTGGTAATTATTCTGACTACTTA
ATTAATATTAATAATGTAACATATGTAAAAATTAATGATAGTCTAATAGAAGAAGATATC
TATTTGGTAAGAAATATTCCACAGGTAACATGTTTTGATATAACGTTGGTCCCTATAAAT
AACGAAGAACAACAAAAAAAGTTTGAAACAGAAATTGTGACAATGGCATTTGAGGATGTT
TTAACTGAAATATTTGACGAATATGATGATAGAATGGTTAGTTATTTTGACAAATATATA
AGCGATGATAATAACAATAAAAATAGCAAATTTACCACTAATAACACAATTGCTATCATA
TTATCATTTGTTTTTACGGCAATAATTATTTTAATTTCTACTAAATTAATAATGCGCTCT
AGAAAAGGAAAATATAGAATAGATAATAATTTTGTTATCTTTAATAATAGCAGTAGTGAA
GATGATAATACAGTTTCATTATCTGATTTAAATTTAGATGATGATAATAATAGTGAGTTT
TTATTTTTTTTGAAAAAGTAA
>SPPV 02 00150 group 169
ATGTTTCCAATTCAAAAAGCTCTCATCTCCTTGTTTGGAAACATAAATCCGTTAGATTAT
GTCTTGGATAAGGATAAAAAAAATCCAAAAGCAATGTAA
>SPPV 03 00024 group 171
ATGTGTTTAAGTAATAAAAAAAAAAAATATATTTTGAAGTACTTTTTAAATAATAGATTT
TATTTATATTTATATTCAGTTAATGGTGGTAATTTTTTAATGACTCATGATATGAGTAAA
AAAGCAGGATTTTATTAA
>SPPV 03 00144 group 43
ATGAAAGATTGTTACGAAGATACAAAATTGGTAATTGTTGAAACAATACCATGCGATCTT
GACTTATTGTATAATGTGTCAACATATGATTTTTGGATGAAACTTATATCAGAAACAAAG
GAAAGTTTGTATATATCATCATTTTATTGGAGCTTATGCGATGAAGATAGTAAAAAAGAT
GAAACTTCAAATAACGGAAAAATGGTATTAAAAGAACTAATCAAGTTAACATCTAGAGCA
TCTTTAAAAATAGTAGTTAATAAATCTAATCAACCCTCTGAAGATTTATTATTATTATCT
AGTTATGGTGCACAGGTTATCTACGTAGATATAAAAAATATTTTTGGTGGTGTTTTACAT
ACAAAATTTTTAATTTCGGATGAGTTAAATGCATATATTGGAAGCGCTAATATGGATTGG
AGGTCTCTATCTCAAGTTAAAGAACTTGGTATAGGCATTTACAATTCTTCATGTTTGGTA
ATGGATTTAATAAAAATCTTTAATGAGTATTGTTATATTGGGTACACTAATGTACCTTGT
TTTTGGACAAATGACTATCAACTATGTTATAATATTGATAATCCATTAAGTTTAATAAAT
AGCAATTATAAAATGTTTATAGCATCTTCGCCACCATCGTTAAGTAAAAACAATGGTACC
GACGATTTATATTCGTTATTATCTGCCATAAAAAACGCAGAAAAATTTATTTATATTTCT
GTAATGAATTATATTCCAGTAATATATCAAAATAATAAAACACTATTTTGGCCTGATATA
GATATAGAATTACGAAAAGCAGCTATAGATAAAAAAATAAGTATAAAGCTACTGGTTAGT
TTTTGGGATCATTCACCTTTAATAATGAAAGGATTTTTAAAATCATTAAAAGATATAAAC
TATAAAAATATTAATATAGAGGTAAGATTTTTTATAATACCAAAAAATCAATTAAATATA
GCTTACACAAGAGTAAACCATACAAAATACATGGTTACTGATAAAGTTGCATATGTGGGC
ACGTCAAATTGGTTAGGTAATTATTTTACTGATACCTGTGGAGTTTCATTAAATATAATT
GATTTTAATGAATTAGCGATAAGGTCAAAGTTAGAAAATATCTTTATAAGAGATTGGTAT
TCTATATATTCATTTCCTATTTCATATGTAACACCAAAAGATAATAATGAAAAGGTACAT
ATAAAACAAGATAGAGATTTTTTTAGATTATAA
>SPPV 03 00148 group 27
ATGGATATATTTACACATCTATCATCTAAAGCATCTCCCAATTCAAATGTGTTAATTTCT
CCTGTTTCTATATCATCAATACTTTCAATTTTACTTTTTGGGTCAAATGGTGATACAGCT
ATACAAATATCATCAGTATTAGAGAGTACATATAATAATTGCTGTTCGGATGATATAATC
ATCGCAAATAGAATTTATGGAGATTTGAATTTGCATTTAAAAACAAAATTTATTGATAAA
TTTGGGAAAGAAATTATTTTGGTAAATTTTAATCATAATACCGAATTAATAAAAAAATGAT
ATTAATGAGTGGATAAAAAGATTAACTCATGATAAAATAAAAAACTTGATAAATGAAATA
AGTGAGAATACAAAGCTGTTATTATTAAATGCTGTGTATTTTAAATTAAAGTGGAGAAGT
CCTTTTAATAAACAACAAACAAAAATGGAAAAATTTTGGTATGATGATAGTAGATATGAA
AATATTGAAATGATGAACGATGTTAATGTTTATCCTTTTATTGAATTAAAAGAACTAGGA
TTAAAAATTATTGAACTACCTTATGAAAACAATTATTCAATGATAATATTGTTACCAAAA
GATATTAAAAAAATAGAAAAAATCTTAACTGGTAATAATTTAACTTTGTGGATAGACAAA
ATGAACCTATACGAAGTTAATGTAAAAATTCCAAAGTTTAAAATAGAAGAAACGTATGAT
TTAAAACTAAGCTTAATTAGTTTAGGTATTATTAACATATTTGATGGATCCGCAGACTTT
TCTAATATGACAAAAAACAAAAACCTATCTATTGATAATTTTTACCATAAAACTTATATA
GAGATAGATGAAGAGGGAACAGAATTATCCGCAGCATCATATTGTTGTGTAGCTGATTGT
GGATTCAATGAAAAAGAATTTATAGCAAACAAACCTTTTATATTTTTAATAAAGGATAAT
ATTAACGTATCTTTTATTTTTATAGGAAAATTTAGTTTTCCTCATATTCGTTAA
>GTPV 01 00004 group 30
ATGAAAGTTATAAATTTTATCATATTAATATGTTGTTTTAAAAGCGCATTTTTATCAGAA
TATTGTAAATATAGAGGGGGTATTTTAACTCCAGTGAATGTAAATTTCAAAGATCCTGCA
TTTTTAAAATGTCCATTGGATAATTATAAAAAATCTATTTTTTTAAAAAAAATATAACA
TCATATAACATAACATGGTTTCAAACTAACTCTCTATCGTTTGTAAGAAATTATAACGAT
TACAAAAATTTTAAAAAGATAGAAGTAAATAATGAAAACGCTTCAAGGGTACAACTTATA
GGAAACAACTTATGGTTGTACCCTGTTATAAATATTGATGACGGAATTTATATTTGTAGA
TTTAGTTCATATAATATTTGTGAAGAAATGTCTATAAAATTAACTTTTTTGGATAAAGAA
GAAACAATTACTTACAGATTTAACGAAGGATTTAATTCAACAGTACATTGTATGGATATT
GACCTTATTAGTAGTTATTTTAAAAATAAGACACTAGATTGGTATAAGAATAATAACAAA
GTGTTATACGATGATAGGATACAAAATAACAAACTTAAGTTGGTTATAAAAAACACAACA

ATGATAGTTCTTTCAAATGGAGTTAGAATATTTATTGATGAACTCATGAACAAAGATATA
TATTTAGGAATTGCTAGTTTTGGGTTTGAAAATGATATTGGTAATATAATAGGTATAGCA
CATTTGTTAGAACATATTCTTATCACTTTTGATCCATCAAAATTTGTCGCAAACGCTTCC
ACTGCAAGAAGCTATATGAGTTTTTGGTGTCGATCTATAAAAGGAAAATCTAAAACTATA
GATGCGGTAGAAACGTTAATATCGTGGTTTTTTGATGAAAATAAAATAAAAGATACGTTT
TCGATTTCTAAAATTAAGGACCATATAAAAGAATTAGAAAACGAATATTATTTTAGAAAT
GAAATATTTCATTGTATGGATGCTTTATCTTTTTTAGCAAACGGAGATTTATATAACGGA
GGTAGAATATCAATGTTGCAAAACATTAACAATATTGATGATATGCTATCAAGTAGGATG
CACAGAATTATAGGTCCTAATGTTGTAATATTTGTTAAAGCATTAGACGAATATACATTA
TCGTTGCTATCAAAAACATTTGGAAAATTGCCCACGTGTCCACTAACCATACCATATAAT
AATTTACCAACGATAAAGGGAAAATTTGTTATGATGCCGTCACCTTTTTATACAGTAATG
GTTAACATAAAACCAACTTTAGATAATATATTATCGATGATTTCTCTTTATGAAATGTAT
CATTTAATTGATTACGAAACAATAGGTGATCAATTATACATTTCAATTTCATTCATAAAT
GAAGACGATTTCGAAAAATTCATAAAAGGTATTTATATTATCGATTTTGAAGCATATAAA
AATATAACGTTTAACTATGGAGATGATTTCTTAATGAACATATATTTATCGTTTCCATGG
ATTCAACACGACATTTATGATTATATAACTGAAATTAATTGTAATATATCAATAATATTA
AAATCTTTAGAAGATAACATATATAACTCTATCGCATCTGGAAACTATGTGGCTATATAT
CCAAATTTTAGTAACGTTATTTTTAATAAAAATGATAGTCAAATGCACAAAATAATAGTA
ATGGATAATAATAACTTTTCTATTAACGATATTTATAATAATACTAATACTAAATCACGG
ATAAAACTAATGAAAAAACAAAGTCAAAATGAGATTTATATAAAATATGACGATGTAGAA
TTTATAAGCTATGTAAATTTAGCATTAGGATTTAAAAATAAAATAAAGAAAAAAAATGAT
GGTGTGCAAATAAATCATCAATTTTCATCAGAAGATATTAAAAATATATTGGAATCCGAG
ACTTTTTTAAAATACAGCAAATCAAAACCTGCTACCATGTATCAGTACTTACTTTTATCT
TTTTTCGTAAGTGGAAATTCTATTGAAGATATACTGTTAAATAGGGAATCTGTTTTAAAA
CCCGTAAAACATTTTAAAAATAAAATTGTGTTTGGTAAAAAAAGTAAATATCATATAAAA
ACATTTTCCAGTTTTGTTTGTGGTTTAGTTAAAGGAATTAATATCACGAATGAATCATTA
ACGAATATGATGTGGGTATTAAAAAGAAAAGGATTAATTTATTCTCTTGATTTTACTAAA
TTAGACAAACATCTGTATTACGTGTTTATGTTTACTATTTATCCAAATAGTGTTGTAAAG
TATATCACGACGGAAAATATTTTTAAAAATCATTGTTTAGTTATTTCAGAAAAAGGAATA
GTAGAAGATTTTTCATCGATGAAAAAAGATATAGTAATTAAATTAATTTAA
>LSDV 01 00051 group 148
ATGTCGGTTAAACAAAAAAATGAAGAAAATAACGTAATAAAAAATATAAACAATACTTTT
CCTACGTCACTCAATTCTATGATTTTTTCAAAACCCGAATCTGTAGCACTTACAAAAATA
GATACGTATTACGATTCTTTGTCTGGAACAGTAACAGTGATTTCAAATAACAAAAAATAT
GTTTTTAATTTATACTTTGACGAAGACATAAGGACTTTACTTCCTATTTTACTCCTTAGT
AAATGA
>LSDV 01 00052 group 218
ATGCCATTTAGGGATTTGATATTATTCCACCTTTCTAAATTTATTTTAACAAAAAATGAA
GAGTCTATTAAAACTACTTTATCTTTATGTAGAGGATTTGATGTAGATTTAGAAGAACTG
ATGCTTAGTTTTTTAAGTAAAAAATATTTAAAAAAACTTACAAATATAGAATATAATAAT
TTATTGCCAGAATTAACGATATCGTTTCCAAATGATATCGTTAACGAATTGATTAAGTTA
AGATTATATAAGTTTTATAAAATTATTAAACCTTCTTTAAAACTTAAAAATACAATGAAA
GGAATCGTATTTATTAAAAATAGAAATGTGTATATTTTACACACAAATGATGAATTAATC
GATTTCTTATTTAAGGAGTATGATCCTTATATATATACGTATGAAATAGATAAACCGGAT
AACTTATCAGGGTCAAAAATAATTTTGTGTGGATATGACAAAGTTACATTTTATACGTAT
CTAATAACTAAAATTGTAACAAATCAAACAGTAGATATAGTGGTTACTGATAAATGCATT
GAATTATTATTACAACCACAAAACAAACAACTTCTAAAAGATTTATTTAATAACAGCAAT
GATATGATTAATAAAATATTAAAAAAGATATTTTATTCAGTTATAGATGGGGGCGGCCAA
ACACCATAA
>LSDV 01 00053 group 188
ATGAAGAAAACGTTGATATTGTTTGGAAAACCTCTGTGTGGAGTTTGTGAATCTATAAGT
GATGCTATTAAAATTTTAGAAGATGAATATGATATATTACGTATAAATATATTATCATTT
TTTTCCAAAAATGGTCAAATTAAAGAGTTTGAAATAGATGAAGGGGTTGCGTTTATTAAT
AACTTCTTTAAATATTTAAGCTCAGAGGCTGCTTCATTATTTAAATATAATCCCGAAACC
GGACAAATGGCATATGTTAATATATCTAAGTTTTTAATCTAGCAGTAATAGATAAAAGC
TTTATTAAAAAAGATAAACTAAAAGAAGAAATAGAGAATTCTCCTTATGGTGTTTGGCCG
CCCCCATCTATAACTGAATAA
>LSDV 01 00054 G5R
ATGGGTATCAAAAATTTAAAAACTGTACTGGTAGAAATTGGCGCATTACACCAAATTAAA
AATGTACAAGAAAATATAATAAATGGAATATTTGTAGACACAATGAGTTTTTTCGTATCT
ATAGCACATTGTGTCAATAATTTAGATGATCTGTATGAAAGTTTTATGTTATACATATCC
CAATGGAAGAAACAAGGAAAAATTACACTGTTTGTTGATAGGGGAGTTATTCCAATAAAA
GAATCACTTAGAGAAAAAAGAAGAAATGCATCAAGAAATACATCAAAAAAAAACGTGTTA
GAAATAGAAAAACTTAATAACTTAATAAAAAATTTAAACGTTGACGATATGATGTATGAC
GAAATAAAAACAGATTTAGAGTTAAAAATTAAAAAGCTTGAATTTAATAATTATTTAGCT
AATCATGTACAATTAAAAGATGCTTTAGATAATTCATTAAAACTACTAGGAAGCGATGTA
ACAATCATATACTGTGATGGTATCGATGCAGAATTTGTCATGTGTCAAGAAGCGAAAAAA
ATAGCATATATGACCGAAAAATGGCCATTAATGATTAGTACTGATCAAGATACTTTGCTT
TTTTCGTCATGCGACGATCTTCCAAAAAATATAAGAACAATGAATCAAATTTATTCGTTT
ATACCTTGCGCAAAAACTAGATATCTTTCTAAGTTAGTTGCATTAACTAACGGATGCGAT
TATTTTCAAGGTCTTTATGGATTTTCTATAACTGCAAAATCATTAAAATCTATTAAATTG
TTTGATGATTTTACTATTGATAATGTGATAAAAAGCTTAATCATTAGAAATTATTCAAGG
AAAGATACAGACAGATATATAAATGCCGAAAAAATCATACATTTTATAAATAAATATTCG
TGCTTAAATGAATCTATATATAATGAAGTCCCCCCTGAAAGTATAACAGTGCAAGAATTT
ATTTTTTCAGCATTATATCATAAATGGAAAAAGTTTGACGATAGTTTACTAAAAGGAATT
TCATTATGTTGTTCACTTATATGTGTTCTCAAACCTAAAAAAGATATAAAAAGGAATGAA
ATTAGTAAATTATGTAATATAATTAATAATGATTCAAATAAGAAAAGTAGTCTAAATAAT
ATCAAATCTGTCATAGATATATTTGGCTATGATCTAAACAAAAATAAAAATATAGTTTAT
AATCACAGAGAGAAAACTAGTGGCGAACACGGGCATTATGAAGATAAACTTTTGGACTTA
AGTGAGGAAGAACCCAATATGATAAAAATAAAAAATGATATTAAGAAAATAATTAATGAA
AGATATAGCAATTATATTTCTATCGACGACGATGAAATTTCTGACATTTTAAAAGATTCG
TTCATTAGTAACGAAGAAATGCAAATAAAAGATTTTGTTTTAAGACTGTTGGTATTAGAA
AAACTATTTCAAACGTCAGTAAAAGAATGCAATTCACTAAAAAATATTATTAAAAGATTA
GAAAATCATATAGAAACTATTAGAAAAAATATGATTGTTTTAACAAAAAAGGTAGATTTT
CAAACAGGCCGAAGTACAACACTATGA
>LSDV 01 00116 group 208
ATGAACGCAATAACAATTTTTTTTATTATTTTATCCACAGTAGCCGTTTGTATAATTATC
TTTCAACTATACTCAATATATCTAAATTATGACAACATAAAAGAATTTAATTCAGCTCAT
TCAGCGTTTGAGTTTTCTAAATCAGTAAATACTTTATCTCTTGATAGAACTATAAAAGAT
CCAAACGATGACATTTATGATCCTAAACAAAAGTGGAGGTGTGTTAAACTTGACAATGAT
TATGTTTCTGTATCAATGTTTGGGTTTAAATCTAATGGATCAGAAATAAGAAAGTTTAAA
AATTTAGAGTCGTGTATCGATTATACATTTTCGCAATCAACTCATTCAGATATTAAAAAT
CCATGTATATTACAAAATGGTATAAAAAGTAAAGAGTGTATTTTTTTAAAATCTATGTTT
TAA
>LSDV 01 00117 group 137
ATGTTCCGAGAAGAGAAGGTAATCGTTATTGATTTAAATCCAGATTTGGCAACATTTATA
AAACATGGATTTAACAATAGAGTTAGATGGCCGTTATTAACTACAGGTGTGGTTTTATCA
AATAATACAACTGCCGTCAATGAAGAATGGTTAACTTCTATAGAGCATATGCCTACAAGA
AAGATATTTTATAATTACACATCGAAAATTTTAAGAAAAGAAGTAGGTTTTTGTGTATAT
TTAAAAAAATCAACAGAGAAAAAAAGAATTATTACATTGGGAGATTTTGACTATTATATT
ATAGATTTAGAAAATTATAAATTTATAAAAATAGATAAACCTATTGAATTAAATGAAACA
CTTTTACACACTTTTCAAGATTATAGGATAAAAAAATTTACAAGTTATTGAAATTATAGCG
TTTAGTTCGAACATTCAAATTACAGAAAAAATAATAGAGGGATTAACATTTATAAATATT
GAAACATTTAATAAAGAATACGACAACATCAAACCTACGTTAAAGACAAGCTTTGAAAGT
ATTATACCGTTTATAGTTACAGCGCCATTAGGTAGATTAACATTTTATGTAGAACATTAT
CCATGGATCGATTTTAAATCACATATGAAAGAAATATTAGACTTTTTAGAGGGATCACTA
GTTTCAGATGTACATTCGCATAAGTTAGAAACATCGGTTCTAGATAATTCAACAAGTTCT
TCTTATAATCCTGTTTCTGGAATGCTGTTTGTAAATGATTTGCTAACTATGACAGTGGTA
AACTTTTTTGGATGCAATTCTAGATTAAATAGTTACCATAGGTTTGATATGACAAAACTA
GATATAAATACATTTTTGAAAGCTCTAGCAGACGCATTTAAAAAAATCGCTAATTATTTA
GAAGTGTAA
>LSDV 01 00118 group 159
ATGAGTGAAGAAGATATAGATGAATCTAATTTTATTCATTTAATAACAAATTTATCAACT
AAATCAGATTGTAATGATTTCACTGCCACTCTATCAACAGTTAAAGAAATAATATCTCAG
ATAAATCTTAGAGTTTTATCATTAAATAAAAAGTCAAAAAAAAATTTTAAAAGTGAAAAT
CAGCAATTAAGTTATGTTCCGAGAAGAGAAGGTAATCGTTATTGA
>LSDV 01 00119 group 134
ATGAATAACTTTTTAAATGAAGCAATTAAAATATTATCCAAATGTTATATAGTTGATGGA
AAAGGTACTATTTGTAAACTATTAATATTAATATTTATACCATCTATTGATGATAATACA
GAATAA
>LSDV 01 00120 group 210
ATGATGAATAGGTTTAAGGAAAAACATTTTTTAAGAAATAGTTTAATAACAAATCCATTT
AGGATAGCATTAGTTGGCGGATCCGGATCAGGGAAAACTGCATATCTTTTATCTTTATTT
AGTACATTAGTTGAAAAATACAAACATATATTTTTATTTACTCCTGTTTACAATTCTGCA
TATGATAGTTATATATGGCCTGATCATGTCAATAGAGTGACGACTCCAGAAGAACTTGAT
TATGCATTAATTACAACAAAAGAAAAAATTGAAAAATATGTCGAAACAAAGGGAAATAAA
AAAGCAGATATGTTTTTATTAATTTTGGACGATTTAGGGGATAAACAAACAAAATCAAGT
TGTTTGTTGGATTTTTTAAATCATGGAAGACATCTTAATACTTCAATAATATTACTATGT
CAGACATATAAACATGTTCCAATTAATGGTAGAACAAGTATAACACATTTTTGTTGTTGT
AACGTGTCAGATTCTGATATAGAAAATATGTTGAGATCTATGTCGATTACTGGGTCAAAA
AAAGATTTGTTAAAATCAGTAAGTTTGATGAGGGCAGTTAATGTTTCTAAGAGAAGAGTA
TTAATTATAGAAGATTCTGTTTTTAGCAATGGCGAACAAAGAATATGTTATGATACTGCT
GATGAATCTGTAATTAGACATATAGTTGATCCAAGTATCCTTTTAAAACAATTTTCACAT
ATGAAGAAAAACTTAACGAAAATATTATCATCGTATGAAAAATAA
>LSDV 01 00121 group 48
ATGTTAGTTGATATTCCAAAGAGTGGAACTGAAACAGATTATGATGAAAGTAATAATTTT
ACAGCATTCGCAGGTTCCACTATATACGGATATGGTTTAAAATCAAAAAAAAATATAAAA
AAAAAAGTAAAATTAATTAATTTCTGTATAAAAATATCAATTATGGCATCAATGGTTTCG
TTAATTACAATAACAATTCTTTTAGCATTTTTTAATAATACATGTGAATTAAATCAATTT
AAGGAACACAAACCGTACTTTTTAAAAAATCCAAATCCTACTACATATAGTGACGACGAT
ACTGAATCTGAGTTAAATGTTTATAGATCATGTAAAGGTATTGTTTATAGCGGATACTGT
TACACGTTTAACTCAGAACCTAAAAGTTTTAATGATGCATACGATGATTGTGAAAAAAAA
AATAGCGAATTACCATCAAATAATTTAATGAATGATTGGATAAGTGACTACTTGGATGGG
ACGTGGGGGGAAGATGGTAACGTACTTTTTAAAGAAAAAAATCAAGAACTTGAAACTATA
GATATAAGCGACGAGATGAGAAGCTATTACTGTGTAAGATCTTTTTTTAA
>LSDV 01 00122 group 81
ATGAAGTCATTAAATAGACAAACCATAAATAAGATTAAACGTGCTTCTGCACCTACTGCT
ATTTTTATGCTAGTATCAACTATTGTTAGTGGTATAGGCACCGCAATACGATACAAAGAT
GAACTATTTCCTAATGCATGTAATAAAGGATGGGTACCATATGATGATAGCTGTTACTTA
GATTCGAGGCTTCAACTTTCATTATATGGTGGTGTAATGTTATGCAATAAGTATAATGCA
AAGATACCTAATGTTAGTATTAGACATTTAAAAGTAATCTCGTTAACTTATGGTAGACAG
TTTTGGTATGGACTAGTAAAAAAAAAAAATAATATATGGGTAGATGTTAATAGTAATAGC
ACTGTAGATATGAATAAAAACACAGAACTTAGTAATATAAAAAAAAGTAGTAAAGGAGAT
ATTAATGCATGTTATGTTTATAATTTTGGCCAATTTAAAAATGTGTCGTGTAACTACGTA
AGTTATATAATCTGTGTTAAAAGGTTATATAATTGA
>LSDV 01 00123 group 105
CATGATGATAGTGGTATTTATACATGTAACTTAAGAATAAACAAAGATAATATTAACTAT
GACATAAAAAGGAACTATAATGTTGTAGTTTTATAA
>GTPV 01 00010 group 56
ATGGAATGTATTGGAAAATCTAAAAACATACAACGTGTTTATGGAAGTTCAATATTTTGG
TTAATTAACGAGTATGTACTTGATTACGATAGAAGTAAAACATTTACTTAG
>GTPV 01 00025 group 142
ATGAAAAAAAGAAAAATATATTTTGAAGTACTTTTTTTAAATAATAGATGTTATGATGAT
TTATATTCAGTTAATGGTGTGAATGTTGTAGTTACTAATTTTTTAAAAAGTAAAGTAAAG
AGTTACATTTTTTCTTTTAATCTTTTTTAA
>GTPV 01 00147 group 33
ATGGAATACATTAGTGATTATGAAGAACTATTAAAAGAGATTTATATAAATCAATCAAAT
TTAAAAATAAATGTGCTAAAAAAGATAATAAATGATATACCTACAACATATCATAAAGGT
ATTTATAACAATTTATTACTAACAGTTTTGGGCAACAAAATTATTAGTCTTAGTGTACAA
AAGTATAAGAAAATTTTGTCATTTTTAGTAGATAATGGCGCCGATTTAAACACAAAAATT
AAATACAAATATAATGCTTTACATTACTACTTATATAGTAATTCAAATGTTACTGTTGAT
ATACTGAAATTTTTAATAAAAAAAGGAGCTGATATAACAAAAAAGTGTAATGGTAACAAT
GTCTTACATACATATTTGTGTAATAAAAACATAGATTTTAATGTTATTAAGTTTTTAGTT
AATAAAAAGATAGACTTAGGAAATAGGAATTTAGATAACCATACTCCAGTTGATATATAT
ATAAGTAATAAGAGAAACAATTGTGATATTGATACATTGAAATTATTATTTCTTGTTGAT
TTTAATATATACAATAAGGAAGATATTTTCTTATCCGCATTAGATGTATTTTTAAATTAT
TTAAAATCATATACACGTAAATCCTTAGATATAGTTAATTATATATTAGAAAACATATCA
ATAAATTCTGTTGATTCTAATGGATTTAATCCAATTTTATACGCTACAGTATCAGGACAA
AAAGTATTTTTTGATTATTTTTTAAAATTAGGATGTAGTATAAATATAACAACATCATGT
GGAGAAACTTGTGGATCCTTATCATTAATGGATTGTGATTATTGTACTTTTAAAACTTTT
TTAAGCAAAAAACCAAATTTACAAACAATCGAAAATACTTTAAGTTGCTTATCAAGTTAT
TTAGAAGATATTTACTATTGTGATTTAAAGTTTAAAATGTTTAAAGAATTACTTTTAGAA
GCTTTTATGTTAGATAGTGAATTTTACAATAGACACAAATCAATCAAATTATATTTTCCA
AAAACGATTTCATTGTATAAAGAACCGATAGTACAAATGTGTAATGATAAAATAGGTGAT
AAATCTGTTTATAATATTATTTTTAAAAACAGTGATATTAGGTATTGCTATAATGATTAT
ATTAACAAATATACTAACTTAAAATATTATGGAAATATTATAAGGAAATATATTTTAGCT
TCTAAGATGAAAAAAAAGAATATAGTTAATATTATAAAGACAATTGATATTTCACCTTAC
TGGAATACATTACCAACAGAGATAAAAATGTACATCATTAATTTTTTAAGTGATAATGAA
ATAAAGTTGTTGGCAATAAAATGA
>GTPV 03 00007 group 172
ATGATTGAAAATTATTTTATGGTACGAAAAATGAAAAATACAAAAAAAAATAAGCAAAATA
TACACTAAGTTTTTAACATATGATGATATGACTTTAATTATTACAGCGTTGACTATATTA
ATAAAATAA
>GTPV 03 00012 group 55
ATGCATGTAATGTACATAACTCAACATATTATTTTTACTTCATCTGTTATGTTAGATATT
TATAAAAAGACTATAAACTCTGTCATTGGTAAAAACTATGAGTTGTTGTGTGTAAATACA
AAAAACTTTAAGATACGTTTAATTGTTCTTATGAAGTAA
>GTPV 03 00026 group 166
ATGAAGAGTTTTTTTACAGTTATAGGATTGTATTTGGTTAAATTAAAACACGAAAATGAT
TTTTTTTTAAAAAAAAAAACGAATCAACATTTAGTAATAGCGTTGTTATATGACATGTGT
TAA
>GTPV 03 00047 group 167
ATGAATATAGCCGCATACTCTAAAAAGCTCAATATTTTGTCAAACGTTAACGATTTAGCC
ATCATAAATCTATGTGGGGCGAATGATTTAAATAGTAACGCGCTACCACTAACTATCATA
AATATAGCTAATAGTGTAACCGTTATCAAAGAAAATGTTTCTCTAGTAGATAATGCCATT
TAG
>SPPV 04 00049 group 173
ATGATAACTAATTATGAACCACTTATTTTATTTGGTATAATTTTAATAACGTTACTAGGA
AACTTAAAACTATCCTTCAAACTTAAAATTAATATTATTTTTTTATACAATCAATATTA
TTTATGTGGTTTATATTCCACTTTGTACATTCAGTGTTTTAA
>LSDV 08 00004 group 163
ATGATAGGGTTAATGAATCCTTATAACATGATTACCTCATCGACAAAAAATCTGTTTTCG
TTTAATATAAACCCCTTTAGGCAAAAGAAAAAAAGTTTCAGTTTTCTATCATATATAAAT
CCTTTTAAGAAAGAGGAAAAAAAGAAAGGTTTGTTTTCAAGTTTTTTTAGTTAA
>SPPV 06 00009 group 71
ATGGATCAAATGAATAATATAAAAAAAACATTAGTTAATAAATTAATTAATGTGCAAATT
ATAAGGATGAACAAAATAAAAGGATATATTGATGTAAAAATTTATAATAACAACTAA
>SPPV 07 00119 group 5
ATGAGATCACATTCATCATTTGAATTGAATACCAAACGTCAAGACTTTGATGTTTTGAGG
AAAGCAAATGCTATGGCGCTTGAACAAAAAATTAATTGGACAATGTTAAAAGAAGAAGTA
AATGATCTTTTTAAAAAAAATTGTACTGATTCTGATAATACAATTTATAACGGCATATTG
TCTGAATCCATAACTTTTGAAATAAATCATGGATTTTTTAATGTGGATACTGAAGTCAGT
ATATCAAAATCATTATTATATATGGATTTAGGAGTAGAAAGTTATAACATTACAATATCA
AACATGACAACGATGACCACACCGTTATATTTAGAAAATAAAAATAACACCAATGATATT
AAAGTAAAGCTTTACATGGTTAATTCAAGTATTATTCTAACATTTGAAAATATAACAATT
GTTATAAATAATACATGTATTGGTACATCCATAAACAGTATTTATGCAAAAATAACAAGT
GATTTTATAACTATTAATGTCACAACAGATCCGTTGTCTACAACACCTCCATTTTTAACT
AAGTCTATGTTTGATAATTGTACCTTAACACTTCCTGTTACTATTTCTCAAGAAGGATAT
TATACAAAAGAATATAATGAACATACTGGTACAACTGAATCATTTACATATAGTTATTAT
TCAGATAATAATATGACAGAGTCATTTACTACTGCATTTAGTTATTATTCGGATGATACC
ACTACTGAACCATTAACTACATCTGATTATTTAAATTATACCACATTGGAACCATCATTT
ACTACATCTAGATACTATTTAAACAATGGTACATCTAATTATAAGAATATTTCATATGTA
TACAATAACAAAACATGTATCGTATTTGTAAAACAACAAAATAACATTTTTAAAAATATA
ACAATACAAACAGAGTTTAAAAATTTATGTAACGAATCAAGTTTAGAAACAAAGGTATAT

GGAATATCTAAGTTAAAAAAAAATTATGCTTTGTTATGACGACCTGTTTTATTTTAACAAT
GAAAACATAATTAGAAATAACTTAAAAAAAAATGGTATTATAAATATATCATAA
>LSDV 01 00055 RPO7
ATGGTTTTCAATTAGTTTGTTCAACATGTGGACGCGACATATCAGAAGAACGTTATGCG
TTATTAATAAAAGAAGTAAATATAAAAAAAGTTTTATCTAGAGTTAAAAATAGTTGTTGT
AGACTAAAGTTATCAACACAGATAGAGCCTCAAAGAAATTTAACCGTGCAGCCTCTTTTA
GATATTAATTAA
>LSDV 01 00056 group 76
ATGGATCCTGCAACTTTTATAAAAAATTATGCGCCTAAAGGCGCTGTTATTTTTATTAAT
TATGAATTTTCCTTAACTGAATATTTTAATCCATCAGAAGATAAACACGCTGCTATATAT
ATCGGATCCGACTGTACATCTAACCATGTTAAAATGTTTTTATCGTGTAGCAAATTCGAT
GAAAAAATGGCAATAGAAGCATCGTATAAAAATGGAGTTAGATTAGTTAATATCGATGAT
TTAATAAAGGGAGCCATAAGTGTTAAAGTTCTTACATTAAATGATGTTATTAGTCAATAT
AAAATGGGATTGGCTGCGGATAACGCAATGAGCTTCTTAGGAATGCCATATGGTTTTGGA
AATGATAATATTTACTGTTTTAAATTAGTAGCTGACTCATACAAATTTTTAGGCGTTACT
CTTCCCACTTATAATATTTTAGGAAAACAAATATTTTTAAGCCAAAGTTTCGTAAATAAT
AAACAATGGAAAATAGTATACTCGTTTTCAAAACCGACAAACTAA
>LSDV 01 00057 group 123
ATGATAGAACCAAAACAATCATCGATTTATTCAATTATATCAAAATCAATAATTAAATCT
GTAGTTAAAGGATTATCGGTCAATAATGAATACTTAGAAAATAAATCAAAACATTTATGT
TATTGTAATAATTCATCATATAAAGAAAATATCATTAATAGTATTTATAATTGCATTGAG
AAAGATATATCAATTAATGATAATAATCATTTAAAGGATATTTTAAACAATTTAAAGGCA
CATTCTGTTTATGTATATAACATAGGCGAATTTTGGCGATTGTATAATAGCCTTTATAGA
TTTACACATTCTAAATCTTTTTTTGTAACATGTATGCCGACAATAGTAACCACGTTGGCT
ACATTAATAACGTTAGTCCTTTCTAATAAATTACTATATGCCGCCGAAATGGTTGAAAGC
ATTGAAACTTATCTTTTTTCATCACAAAAAACTCCGGCGCAAGAACTTGCAGATCTTTTA
GAGATGAAATATGGATTAATAAATTTAGTACAGTACAAAATATTTCCTATTATCATGGGT
ATAAAAGAGCCACTTATAGGAACATTATCAACGCCAATATTTTTTGCAGGATCTACATCA
ACAACTGATTACAGTGTTGAAGTAAACAAAATAATGGAATTACCAATTAAAACAGATATA
ATACCTAACTTGTATAAGTTTTTATCTGAAAAAGGAATTAATACATCTAATAATTTTGCA
GAATACGTTGCAGGATTAAAAATTAACGAAATAGTTAAAAATACATGCGTGCAAAATGAT
GAAGACAATAACGTAAAACAAGTTCTAAAAAACAATAATGAAAATTATTCTGGTAAAATA
ATAGATTTACTAAAAGAGGCTGAAAAACATTCAAAGGGTCATGTTTTAGATGGTGCCGTG
TCGAGCCCGATAACAGAAAACGCATTTATTTCAAACCAAATTCCGTTATCTGTAACGGAT
ATGGAAAAATTTACAATATTAGAATATTTATATATAATGCGTGTTATGGCTAATAATATT
AAAAAGAAAAATGGGGATAATAAAACTAGTGGTGGTATTATACTTAATATAAACTCACCA
TTTAAAACAATAACAGTTCCTGGATTAAATTCTGTAAAGTAA
>LSDV 01 00058 VLTF1
ATGAGTCTTCGTATAAAAATAGATAAATTACGACAAATAGTTGCGTATTTTTCTGAATTT
AGTGAAGAAGTTTCAATAAATGTTGATTCAAATAGTAACTTAATGTATATTTTTGCAGCA
CTTGGGGGATCTGTTAATATTTGGGCAATCATTCCATTAAGTGCTTCAGTTTTTTATGAT
GGTAAAAACAACCAAGTATTTAATATTCCAGTATCAAAAGTAAAATCGTGTCTTTGTAGT
TTTCATAACGATGCGATAATCGAAATAGAACCAGATATTGAAAATAACTTAATAAAATTA
TCCAGTTATCATGTTGTAAGCGTTGATTGTAATAAAGAATTAATGCCTATACGAACTGAT
ACAACTATTTCTTTAAAAATAGATCAAAAAAAGTCTTACATTTTTAACTTTCACAAATAC
GAAGAAAAATGTTGTGGTAGAACAGTAATTCATTTAGAACTATTGTTAGGTTTTATAAAA
TGTATAAATCAATATCAACATTTATCAATTTTATTTAAAAACGACAATATAATTTTAAGA
ACACCTGGGAATTTAGATACGTTTTCTAGAGAATATTCTATGACTGAATGCTCTCAAGAA
CTTCAAAAATTTTCATTTAAAATGGCAATATCATCACTTAACAAACTAAGAGGATTCAAA
AAAAGAGTTAATGTGTTCGAAACAAGAATCGTAATGGATAAAGACGACAACGTATTAGGT
ATGTTATTTAGTGATAGAATACCATCTTTTCGCATTAATATTTTTATGGCATTCCACGAT
TAA
>LSDV 01 00059 group 124
ATGGGTTCTTCTTTTACAGTTCCTGAAAAAATTAAAATAAGCAACAGGCCAAAAAAAGAA
ACAATGGAAATGAGATTAAATGTTGATAATATGTATGAACAAATAAAAACATTAAATCAA
ATCAATGATGAGTTATATATAGGCATAATAAACGAAGAAAAAAAGAAACAACTTATTAAG
CAATTTCCTGAGTTTTTATTTATTGAATCAGGTCCAGGAAATCTACATAAAGTAATAAGA
TCGAAGTATAACAATGATAAAAAATATTGTTGTAAAAGTATGAATCTTTTTAGTTATTGG
CAAGACAATAATGGAAACATTACTGATTTTTATAAACCAAATTCTATTTTGAGTAGCTGC
GATCCGGATATACAAAACAGTGGGTTTTGCGACGACACACTTTTAAGTTGGTGCGATACC
GCTACACAATCAAACTCAAAATTATGTTACGACTGGATAAATTCTGCATTAAATAGAAAT
AATTCATCGTCTGAAAAATTGATAAATGGACTAATAACAACGTGTTCTAATAATGCAAAT
ACGCCAATATGTAATATTTTTTTACATTGTTTAAGAGTAAAAAACACTGAGACATTTGAT
AATGTAATAGATTATATTTTATACTCTCAAAGTGATGATTTTAAACGAACATATATGAAA
TGTAGTTATCCATCAAATAAAATTATAAAAGAATCCCTAAAATTTTTTGAAGCGAGAGAG
TGTTGGGACCCAAATTGTTCAGAATCTAATTTAAACTTTTTATTGACAAAAAATTATAAA
AATCTTGGTTTGTGTAAAATAGATAGATGTAATATTAGCATTAACAATTTATTTTTTGAT
GAAGGATCTAGTGTACGGATGTCTTGCAACGACGAATCTATAAAATCTAAATTTCCTATT
AATAAAGAAAAAGTAATTTTACATAATGTAGATAATTCATTTCAGTTAAAAATACATCTT
TTAACAATATTATCTTTATTTGTTATATGGGTATTAATTGTAGCTATTTAA
>LSDV 01 00060 L1R
ATGGGAGCAGCCGCAAGTATACAAACTACAGTAAATACGTTAAATGAAAAAATAAGTAGT
AAATTGGAACAAACTGCTGAAGCCACTGCGGAAGCAAAATGCGATATAGAAATTGGTAGT
ATTGTATTTAGACAAAATAAGGGTTGTAATGTTACTGTAAAAAACTTGTGTTCGTCTAAA
GCAGAATCTCAATTAGATGCTATATTAAAAGCAGCAACAGAAACATATGATTCACTTACT
CCTGATCAAAAAGCATATGTTCCAGGATTGATGACAGCGGCCTTAAATATCCAAACAAGT
GTTAATACTGTGGTTAAAGATTTTGAAACGTATGTAAAACAAAAATGTACATCGAAATCG
GTTATTGATAATAAATTAAAGATTCATAATATTTTTATAGATGAATGTGCTGCACCAACC
ATGGATTTTGACTTCATTTTCGACAAAGACGAGGATGATATTTATACGTTAATAACAACT
TTAGGTGTATTAAAAATAAAAAAGAAAGAAATATCAAAAGTTTGTAGTGAGCTAGGAATT
AATCTTATAGAAACATTAGGACCTTATAACGTAGTATCTTTAAATATACACCCATTTCCT
AACAATTTCATAGAACAATCAAATTTGATTAATTGTTATATATCTTATAATGGGACACTG
TTTCACTGTTCTAAAGATGAAAGACTGAGTATCCCAATAAATGGCTTGTATAGAGGGTTT
TACTCTAACAATAGTTTTATATTTTCTTTTGATAAAGAAAATTATGGTAAGCTACTAATT
GATGAAAAAGAACAATATTTTTACCTAGGGACAGCATATGATATAGTTAATTCAAATATC
ATCGAAGTGTATAATTTATACAGGAAAGGAGATTACAATTTTATTATAAATCCATCGGAT
AATTTTTTAGAAATGATAGCTAATCAATCAAAAATGTGCTTAACTGATAAAAGTGGGTGG
TGTATTGTGGATATAAAAAATGAAATAGAATATTAA
>LSDV 01 00124 group 160
ATGATTAACAGAATAAATTTATCGTATGGAATTGTTAAATATATTAAAGCTTTGTTATTA
AAAGAAAATATATCAGAATCAGATAAAAGTATTATTAAAACAATAATAAATGAATCCATT
TATCCACATAATTATGTTTATAATATTTTAGATTTTAATTCACTAACGATAAATTTTAAC
ACATGTGAAGAACGTAACATAATAGATTTTAAAGATGTCAATATAAAACCACAAAGGTTC
ATAAGCTATTATAAAGATTATATATTAGAATCTAGCGAATATATGTTTATAGTTTGTTTA
AAAGGAGAAACCATTATAAAATGTTATAATAACAATGTAATTTCATCTAACAAAGTACAA
AAAGGAGAAGCTTTTACATTAAATATAAAAACTAGATATAGCACAATAACAAAAAATAGA
GATCTACATCTAGCGATTATAACATATACCACAATTTATCCATTAATATATTATAAAAAT
ATTGTTTTTTCAAAGGATAGTTCATTATATAACATTTTTTCCGGATACAAATTTGCACTT
TTTAGAATTTCAAGCGACGATGATAAAGTTTTAGAAGATATAATAATTTTGAATGGGAGG
TACTATTACAGCAATTCAATGGAAAAGGTTAATATAATTAATATAAAGACTCTATTGGAT
AAGTACAATATAAAAATTAATAGTTTTACACAAATTGATTATATTCCTAAAAGCTCACAC
AAGGCATGTGTAGAGGCATTAGAGTTGTCATTAGATGATGTGACAGTCAGACAAATTTAC
GATGGTACTTCTTTTAAAGATGTATGCTCTATATCTGGTATCAAATATAAAATAGTAATT
TCGTATTGTGTTCTTTGTTATAATTAA
>LSDV 01 00125 group 50
ATGGGAATAGTATCTGTTGTATACGTCGTAGTACCATTTTCATTTATTGTTTTACTTTCA
TATATATTTTTTGAATACAAAAATGTTATTAAAAAAATGTTATTTAAAAAAAAAGGGAAA
AATCAAAAAAGAACATGTGTTAGACTTAATTCAATCACTTATTCAACAAATAGTATAGAA
TCCACTATATCAGAAAGTACTTGGTCAAATTGTAGTAATGATACATTTGTAAAAAATGAA
AAAGAAAATGTAGAGATTGTTGAAATTAAACGTTGTGATAATGAATTAATTGAAGAAAGT
AATAATAACGTTTTAGAAAATGGATGTACCACAAATACAGGTGAAGAAAATTTAATTTGG
GACGATAACAACGTTTATGATTTACCACCTAATGATAGTGTTTATGATTTACCACCTAAC
GATTTGAGTTGTAACAACGATTGTGTTTATACATTACCAGATGACAATGTTTCAAACATA
GAGGAAAAAAATAACTAAGTTAATGCACAAAAATAATTCCGAGTCAAACTATTATAATTGT
TGTTAA
>LSDV 01 00126 group 82
ATGAAAAATAAAATATTTAGGTCGATTCCGTTTTTTGATATCATTAAAATAAAAACGCAA
GTTTTAGATTTACTTTGTATGAACATAACAAAATACTATAATGAAGAACATGTAAACATT
ATAGTAAAAGAGATAGAACAATATATTAACGAAAAGGTATTTGTAAAAAATTTAATATCG
ATACGATGTTTTAATAAAGAAGGATGTGATAAAAATAGACAAAATTTTAATACATCTTTT
TCTAAAGCACTTATATGTATCAAATCAGCAAAAAAAGGTGGGGTAATGGTTATTTTTAAT
AAAGCTACAAAAAATAAAAAAGTAGTATATCCTAGTTCTAATCATATAATATTGTTAAAT
CCATTAAGTTATTACACCATTTCTCAAGTAAATAGGGGTTCGGTAATAATGATGGTATTG
GATATTGATATTCCAAGTATGAGAATAATGTATAAAAGCATAAATAATGTAAAATATTCT
AATGTATTAGCGTTACTATGCCCGTTTTTTAAAAATGAGTTTGTTTTTGCACTAAAGCAA
TTAATTGATATAAATAATAATCAGGTCTTATGTGAACAAATAGTTATTAACCGGGAATAC
TATACAGTTATTTCGACAAATTTAAAAAAATTCTATATACCATCTATATGTTTTGGAAAA
AAAATAATAGATTTTAACTATGGATATGATGATTTTAATGAAGACATAATAAATAAAATA
ATAAACAGTGATACTCCATTTGATTTTATTTCACCACAGAAAAGTATTATTGATGCGTCG
TTAGTTTATGAAAAAGTTATATATGGAAAAATAAATGTTTAA
>LSDV 01 00127 group 64
ATGTTTTATTTTAAAAAAAAGAAAGTAAATATAATATTTCTTTTTTTGACAATTTATATT
AATTCATCTTTTTCAATATCAGAAAACAAAGTTTCTTCAATAAATTATTCAAAATGTAAT
AAAACAATTGTCATCGACTGTAACATTAATCATTTTGTTAAGTATAATGAAAATGTTACA
ATTAATTGGTTTTTTAATAACTACCCACTTTTTAAAAAAAATAATACTAATACTCAAAAA
CTGATATTGGATTTTTCAAAATACTTATACGGAAATTATACCTGTTATGTAATTAATAAC
AATAATATACTCTTAAAAGAAACTATACAATTAAAATATATTATAAAATGGTTAAATGAA
AAGGAAATTAATACAGTTGTTATAATGCTTTCTATTTATGTTATAATACTATGGGGTAAA
ATAATAGCTACAACAATTAAGTTTAAACTTAGTTTTAAAAAAGATAAAATAATTTATCTA
TACTATTACATAGGATTTGTTATAACTCTGATCATGTTACTAGGACAATACATGATAGGA
TTAAACACAAACAATATTTTTATACGTATTTTAGGAACTTCATTTATACAAGTATCTATT
TTTATATCTATAATATTACAAATAATATTTTTTAAGAAAAAATTTAAAAACACATTATCG
CTAATAACAGTTTTTTTGTTACATTTTATATCATATATAATATCACTAATTATGTTTACC
ATGTCCCTTATAGGGTGTTATAATGAAATATATGGCAAAGTCATAGTTTATAAACTATTA
TTGGTTATTATTTTTGAATTTATTAGTTTATTATCATTGTTTATCTTTTCTTTAAATACT
AGTATTAAATACAGAAAACTTCATATAATAGAAGACGAAAACTTACTTTTTTCTATAGAA
TAA
>LSDV 01 00128 group 83
ATGAAAGAGGTTTATACAAATTTTAATATAAGTTTAAATACTACAAATCAAAAAATTGAG
GTAATGGGATCAGTTCCTACAATCGATGGAAAAGATCCGTCTATAGATATTAGAATTGTT
AGTAAACCAAATAAAGAAAAAAATAATTTTGATAACACTAACGAAAATATTTTTTTAGAT
GATGAAAATAGTAACGACTATGATGAAAATTTTATAAAACCTAATAAAGTTTCGTATGAT
GAAACAAAAAAAGGAAAAGGAGACAACATTGAACAAACCGATAATGAAAAACAACTATTA
AGTGATTTTAGTGATATTCTAAACAATGATGAAAAAAATCACGATAAAGATAAAAAAAAG
GTTGTAGCGTAA
>LSDV 01 00129 group 51
GCTGTAGGGGTTCCAGAAAAGTTTAATACAATTTTAAAGAATATTAGTGTTGAAAAATCT
AATGACAGTGATTCATATTTTATTTGTAAAATGATTGAAGATGGAGGGAATTGCGAAATT
GATATATTCATGGATGCTGCAACCAATAATATTAATGATGAACTGGATAGTTCTAAAAAA
GGAAGTAATACCAGACACGCTAGATCTGTTGATCATATAGAGTTAGATCCATTCTGTTTG
CATATGTATCATGGCATTGACGAAACCATAGATTGTTCACTAAAGAAAAACAACAAAGAT
ATGAGTAAACACACAAACAGAATCAGGAGATCTCCTCCTGATAAAGGTAAAAAACCACAA
GTACCGACAAAAGGAAATTTACAACTAATGAGCGCTGAGGAAATGGGAGCTAGACCAAAA
ATAAGAAAACAAACGCAAGATGTACAAATTGGTGCTAGAGGGGTTGATGGTCCAGTTTCT
GGATCAGAAGAAATATATTCTCAAGTTAGGAAAGAACTATCTCAGAAGTTAAAATCTTTA
GTCTTGGACGATGATGGAAAATTGACAACAACAAGATTGCCTAAATCAACAAAAGCTTTA
ATTGGTGGGTTGGTAGATTCTAAGATAGAGACATCTAGTGCTGCGTATGATATAACAAGA
CAAATAGTTCATCAACAATCAGGAGATATTTATTCGTTGCCTGTAAAGAATAAAGTATTC
GGTGTAGAATCAAATAGAAAGATCACATCTCCAATTATAGACGTTGATACATCATCTAAT
ATATACGCAAACGTTTTACAAAAAAGAGATTTTAAAATTACGTCTCCAAAAAAATGTTGAT
GTATATGAAAAAACTAGAACTGATTTTTTTCAGACGATTCGTATTTTTTGAAAAGATCA
TTTTCTTCTAGTTCCGACGATACTTACTTTTTAAAAGAATCAGATGACAGCGATAGTGAC
ATTTTTAGAAGATCTTCAAAAAAGGATCAAACTTATTCACTAGCAGGAAAACCATTAAAA
AAACAATCACTTAGAAGGTATTCATCTAGTGATCATGAAACAATAGGAGAAAATATATAT
GAATCTATTCGTGAACCAGAATATGCACTGCTTTCAAAACCAAGAGTTTTAAATCCACGA
AGTCATATACCTATACCATCATTACCTAAGGATGAAATACCATTTACTCAACAAAAAAGA
AAGGTGATTGACATGATTTGCATCCTCTATATGTAA
>SPPV 07 00120 group 6
ATGGTTATGAGGACCGACGAAGAGCCTAGTGAAAAACCTAATATGGCAACAAATACGTAT
AATAACAATGATAAAGCTAATAATAAAGATAAAAATAAAGGATTTTCTTACTTAAATAAT
GATATACAAGAAGATGAAAAAAATGTTAATAAAATTAAAAAAAGGAAGAACTAA
>SPPV 07 00123 group 7
ATGCAATTATTAAGGATATCAACTCCTGGTGAACCTAGAGAAGCTGAAGCAATGAGTTCT
GATGTTTGTGATATTTATCCTCTTTTTTATGTATTAGCTGGAAACTGCCCTTTTGATATG
AGCAGAAAATCAGTAGCTTATGTTACATGTAGTACGTTACTAAGAATGTCAACGTATGAA
CATGAAAAACAGAGGTGGATTTTAATGAATCCGTTTTCAAACAACAATGAGGACAATATT
CAATTGTTTACGTTTAAAAAATATGACTTTAGTTCAGAAAAAGATAAAATAAAACTAAAT
AGTATTTCACATAGTGATACTAGTACATGTTTTTGGGCAGATGCTATGATTTTGGAAGAT
GTAACATCTTGCAATTCTAGGATAAGAAAATTGTCAGTTGAGATGTCTACAGTATCAGAA
AAAGGATATAACAATTTTGTACTTACGTGTCCATATGGGTCTACACCGTTTTATATAAGT
AATGGTTCAATAATAAGTATTCCAATGAACACAAGAAGGACGTCTGTTAGATTTGCTTCT
AAACATAACAATGTTGCACTAATATCATGTATACATAATTCAAACCCTGCATATAAATCT
GATATCGTTGAAGTTATGTTTAAACAATCAAATATGAGTGATATGTATTTGGATTTCAAA
TATTTTAAAGATAGAAAATATTTATTTGATTCATTTAGTGATATAATGCCTAAAAGATCA
AAAACTTGTAAGAGAGCATCAGAAAATAGCAATTGTAAAAACTATTATTATATTAAACAT
GTTGCAAAACTTCCAATGGTTAGATTAGGCACAAGTTATTCAGGTGTTTTGAACTATCAA
ACATTAGAAAAAGTTAATAAATACTTTTCAACACCTATAAGTATTTCTGTAGATGCTAGT
ACTTTATCCAATGTATACAAAAACCAAGAACATTTTTGGAAATTTGCAATGGAAAAGAAA
AGAACTTTTAGTTCTATTACAGCTACAATATTTGCGTGCTCTGTTGTAGCTGGGAAGGTA
AATGTAAACATGGGAGTTAAAGGTTCACACGATTATTATGGAAGAAGCGGTAAATATATT
TACATGGGATCAAAAGATTTTGCATCTAATGATAAAATATATTTCAATTTTATACCTGAC
AAAGCAGAGTACCGTTTAAAGGATACATATGGAGAATGTGAAATTTATTTAGATTTAAAA
ACTAGGAGAGTTAATGTAAACTGTCCGGAATTGACTATACCACAACATCCATTTGATTCA
CTAGATGTTAATAGTTTATGTGTGTTAGTTGCCACATCAAGAGATCATTGTGCTATATCT
GAGGAAAATTGGAGAAGTTATGATAGAACTCATGGTGTTGGGTATAGCCACGAATACGTA
GATGCGGAGTTTGATTCTTGTAGTAAGAACCATGGTCCAACATATCCAGTTGATAACTTT
TGCTTTTATTGGTCTGCGGGGATATATTGGCCACCTGACTATGACCCATGTGCTTCATCA
ATGGTTTTAGGTTATTCTCCAATTTTTACAGAAAATAGAATTGTAAACCCTCCATATATA
AAGGAATTTGGTTATGAACCAGAAAAAAATGAATACGTGAAAAGAGAGTTATATAACAAA
TTACAAAATCTTTATGAAAAATATAATATGTTGGTTTTATATTCAATGAACCCAGTTGTT
GAAATGTCTAACGGATTAGCAAAATCTATGACTTCAGAAGCCCGTGAGATTTTTAGGTTA
ATAGCAAATAGTAACGAAATGCAAAAAGCTAAAGAAGAAAATGATATGAAAGCTGAGAAA
GTTAAAATGGAAATAGAAGAAACATTATCATACTCTGAATCAACTTCCCTTTTACGGTCT
GCTATTTCCACAAGGTGTTGTGTTTTGGACGGTACTAGTGTATATAAGTATTTTGATTTA
GAATATTATCTTTGTGGTAATTATTCTGACTACTTAATTAATATTAATAATGTAACATAT
GTAAAAATTAATGATAGTCTAATAGAAGAAGATATCTATTTGGTAAGAAATATTCCACAG
GTAACATGTTTTGATATAACGTTGGTCCCTATAAATAACGAAGAACAACAAAAAAAGTTT
GAAACAGAAATTGTGACAATGGCATTTGAGGATGTTTTAACTGAAATATTTGACGAATAT
GATGATAGAATGGTTAGTTATTTTGACAAATATATAAGCGATGATAATAACAATAAAAAT
AGCAAATTTACCACTAATAACACAATTGCTATCATATTATCATTTGTTTTTACGGCAATA
ATTATTTTAATTTCTACTAAATTAATAATGCGCTCTAGAAAAGGAAAATATAGCATAGAT
AATAATTTTGTTATCTTTAATAATAGCAGTAGTGAAGATGATAATACAGTTTCATTATCT
GATTTAAATTTAGATGATGATAATAATAGTGAGTTTTTATTTTTTTTGAAAAAATAA
>SPPV 07 00134 group 13
ATGAAACATTGTATTAAATATTTATTCACTTTGGATCCTGACGAATATAAAAATCATAGG
TTTATTTCTTTAAAGTTTGAAAGTGTCGTCAATGAATGTCAAAAAGATATTATAACTATG
AAAAGTACATTTTTAAAAACTATACCTGTTTACAATTTAATATTTAACAAAAATGGAACA
ATGCATAAAAGGTATTTGAATACCATAGAAGTAGGAAAATACTTAAAGTCAAATATTTAT
GGTAAAAGAGTTAGATGTGTAATAAATAATTCAATAGAAAAAGATAAAAAAATTGATTAC
ATAATTAAAAAAGTTAATATATTATGTAAACATACTATTTGGGATATATTACCTGAAGAA
ATAAAAGACAATATCTTTAACTGTATGTCATTGAGGGATATTAGAGTAATATTTCATATG
TTTTTTGTGTAA
>LSDV 14 00082 group 59
ATGAATATTTGTATAATAAAAAATAACGAATATCCAAAAACTGATTTGCATGACGTAACA

GGAACAACGACAAACTTTGAATTTATTAATTCTGGAACCAGTCAGGGAATATGTGCAATA
AAAACGTTAATGGATGTAACCACAAAGGCGAGTACAAAATTTTCCCCTAGTCAAAGTTCG
GGATACGGATATCAATTTTATATAATTGCGGCAGTTGTTGTAATATTATCGATGGTTTTT
TTATACTATGTAAAAAAAATGCTATTTACTTCTACAAAGGATAAAATAAAAATTATTTTA
GCTAACAAACCAGAAGTTCACTGGACATCTTATTTGGATACCTTTTTTAGTAATACACCA
ACAATCATAGAAAAATAA
>LSDV 01 00061 group 78
ATGGCGGAAATATTAACAAATAAACTAAAAAGTATAGACAATGAAAATAAATACAACGAA
AAATTGATAGAATGCATAATTCAAGAAATAGAAAAACAGCAATATTTTATTTTAAAACCC
ATAGTGCGATTTATCATAGATGTAATGATAATAGTAATTGTTTTAAATGATTTAACAATT
AGGTTATTGAAGCGAAACTATAAAATTTTATTTTCTATCGTATTATTATCTATACTTTAT
GATTTATTTACACATAGCGATGTTTCGTCAGATATATAA
>LSDV 01 00062 group 223
ATGAATAGTAATAAACAACATAAACATTATGAAAATGATAAAAAAAATAAAAACTTTAAA
AAAGATTCGGATACTAAGTATAATTGTTATCACGATGATTTTATTTCAAATAGATTAATG
TTACACGAAAAAGAATCATTAGATTTAGAATGTAATTTAGTAAAGATTAACTGTATATTA
AAACAAGCGTGCAAAGAAGAAACCGAATGTTGGATTGAGTTATCATCATTAGTTAGATCG
AAAAAATCAATAGGGTTTCCATTACTTAGAGGTTACAAAAGTTTTTCATCTGGTAAAATA
TTATACTTTGAACAATTCAAAAATACAAAGGTTGAAAAGTTGGTAGTGACGTCGTCCTTA
CTACACGAAAGTATATTATTCCAAATTGCTGTAATTTTATATTCAATGCATAAAAGAGAA
ATATACGTAGATAATTTCGAATTTGACATTGTAGATATTCCAAAAACTACTTTTTTTATT
TCAGTTAATCAAATAGTCTTTGAAATATGCACCGACAAGTTGGTAATATTATCTTTATGC
ACTAGACCTTATAGAGTAAACCTACCACAATCTTGTTATTTAAACTTTTTACATTGTTTA
GAAACAACAATGAGGAAAAGTTATGAAACCTCAAACTACTTTTTTGAGTGGTTAATAAAA
AATCATATTTCATTATTGTCTAAAGATGGAACAGATTTGTTTAAAATACGCAAGAGAACC
GTTACTACCAATCACATAAATAGATTCGTTGAACCAGGAACCATAGTTTTGGTATATAGA
GATGATATGTACATATCTGGAATAACATTAACGGATATTTCAATTAGTGACAATGTAAGA
GTTTTGTTTTCTTTAGATGGCGGATACATTTTAGAAATAGATGATTTTAACATACAAGAT
GTTTTTCTCGCTAATGAATATTTTATAAGATCACAATTAACCTCAATAATTATTTGA
>LSDV 01 00063 L4R
ATGAGTCTCATTTTAGAAAACCTGATTGATGAAGAAACAATATTTTATGCGGGCAGTATA
ACCGATTATACAGAAATGGAATTAATAATCGCAGGTGCTAAAATAAAATATCCTAGATCG
TTTTTATCTATATTTAACATAATACCAAGAAATATGACTAAATATGAAATGGATTTAGTT
CAAACAGAAAATATCACAGGTGCAGTATTTACAACAGCTTTTAATATAAAAAAGAATTTG
GGTATCGTAGATGAAAAACTTACTATAGAATCCATAGAAAATTATTATTTAGATCCCAAC
AATGATGTTTTGACTCTTATGATTAATAATACTTCATTAGATAATGTAACAACAAGAAAA
AAAAGATCTAAAAGAAATAAAAATCCTGTACTTTTTAGGCAAGGATCCATTCCATTATTT
TTTATCTTTGAATCCAGAAAAAAGGTAGAAATTTATAAAGAAAATGTTGATAAAGCATCA
TCAGATAATTCATATATGAAAATTGATGACAATATTGCTGTAGCAAGCAAGTATGCTAAT
ATGTCGTTATTAGATGTTCATTCACCATCCGCATCAATGAAACTTAATGGAGTTTATGGT
TTTACGTATAAAAATGAACTAAGAAAACTTAGTTCAGATAAAGAAATTGATGAATATAGC
AACAAGCCACTACAAGAACCAGTTAGGTTAAATGACTTTATTGGGCTGTTTGATTGTGTA
AAGAAAAATATACCACTAACAAATATTCCAATTATGGAATAA
>LSDV 01 00064 L5R
ATGGAGAAAAATAACTCAAAAAACATTTATTTTACTCCTGTATTTATAGAACCTACTATA
AAACATTCCCTTTTGCAATCTTACAAATATACATATATCATAATATTTGAAATTATAACA
GTGATGGTATTATTATTTTTATTTTTTAAGTCAGAAATCAACATGTTGTTTAATTTCAAA
CAACGAAAAGTTATCAGCCCGATAGATAAATTTTCAAAAACCACTTTATACTGTAAAGAA
AATAAGCTTTTTATTAGTGGGTTACCTAATACTATGTACTCAAAGGAAGCACTATCATTA
AATAGACAACCGATAACATATAAATATTGTAATGATCTTTTACAATCAATAAATGGATCA
CAGCAAGTATCTATTAACGATATTCTTAGAAAATGA
>LSDV 01 00065 group 164
ATGGATCACAGCAAGTATCTATTAACGATATTCTTAGAAAATGATGACTCCTTTTTTAAA
TACTTATCAGAACAAGATGATGAAACAGCTATGTCTGATATCGAAACTATTGTAACATAT
TTAAATTTTTTATTGTCATTGTTAATTAGATCAAAGGATAAATTAGAGTCGATAGGTTAT
TATTATGAACCACTGTCTGAAGAATGTAAAACATTAGTTGATTTTTCCAATATGAAAAAT
TTTAGGATATTATTTAATAAGATTCCTATAAATATACTAAATAAACAAATAACTGTAAAT
AAAGGGTACTTATCAGATTTTGTTACGACATTGATGAGATTAAAAAAAGAACTTTTTTTA
GAATCACCAGAGCCGATAACATATATAGACCCTAGAAAAGATCCAACATTTTTAAACATT
TTATCAATATTGCACGAAAAATAA
>LSDV 01 00066 TK
ATGGACTATGGATATATACATTTAATTATAGGACCTATGTTTTCTGGCAAAAGTACTGAA
TTGATAAGAATAGTTAAAAGGTACCAAATAGCGCAGTATAAATGCTGTGTAGTAAAATAC
TTAAAAGATATCCGATATGGTAATTCTGTGTATACGCATGATAATAACCATGTATCTGCC
ATATCAACAACTTTATTATATGACGTCGTTGATAAAATTATGAATTTCGACATTATAGGT
ATAGATGAAGGCCAATTCTTTAAAGATATTGTATCTTTTTCTGAAAATATGGCAAATATG
GGAAAGATAATTATAATAGCTGCACTAGATAGCACGTTTCAACGAAAAGAATTTAATGAT
ATATTGAAATTAATACCGTTATCTGAAAAAGTAACAAAATTAAACGCTGTATGTATGGAA
TGTTATAAAGACGCCGCATTTTCTAAGAGGATCACTAAAGAAAAGGAAATAGAACTCATC
GGGGGTAAGGAAAAATATAAATCTGTTTGTAGGAAATGTTATTTTTTAGAATAA
>LSDV 01 00067 group 97
ATGGGTATCAGACACGAGTTAGATATTTTGCTTGTTTCTGAAAATCTCGCACTGAAGAAT
GTTGAACTTCTTAAAGGTGATAGTTATGGATGTACTATTAATATAAAAGTTAATCAACAA
AAAAAATTGGATTTTATTATTATATTACGGCCCGATTGGACAGAGGTAAGGAATGTTAAA
AAAATAAATATGGTATGTAACGGTGTTGTTATTGATACAACACTAATCAAAAAATCTTTT
TACGAAGAAGTATATTCATCATCTGTAACAGTTTTTCAAAATACTACCGTTGAATTTTTT
AGCGATACTAGTAAGAAATATAAAGAAGAATATCCCATTGTTAACATAAACACCATAAAG
CGTTATTACGAAATAAAAGATTCAAGAATGACATGTATAAACTTTGAATCACCTATAAGT
ATGGAAGAACTTGAATGTATGTCGATAAAAATAACTTCCGAAAATGGGAGCACCATTTCC
ATATACTGTGAATCCAAAACTAAAATATCATTAGATATAACAACAAAAAAAAATAACATA
AAACCTATTACAACAAAAAAATCTATTAAAAAAATAGAAAAAAATGATGATGATGATTTA
TTCACAGACCAAGAAAATGCAATGGAAATAGATGGGGATCATGCAACTGATAATAACAAA
CTATAG
>LSDV 01 00130 group 84
ATGATTAGGAAATATTATGAATTTAATAAAACTGTTTCAAAAGCGGTCTGTGTTTTGAAA
GGATATAAACTTCATGGTGTTATTAATTTTGATCAGTTACAAAATGGAATAGTAATTATA
TCAGGCGTTGTGCTCGGATTACCGGAAGGTAATCATGGATTACATGTACACGAGTTCGGT
GATGAAACTAATGGGTTTTTGAGTATGGGGAATCATTATAATCCTGAAAATAAAAAACAT
GGTAGCCCTTTTAATAATGAGAGGCATATTGGAGATCTGGGAAATATTTATTCAAACAAA
TATGGAATATCTTATATATATATATTAGATGGTAAAATCTCTCTTGTTGGAGATTATTCT
ATTATAGGAAGATCGTTAGTTATTAGTGAGAAAAATGATGATTTAGGCAAAGGATATAAC
TTTAAAAGTTTCATAGATGGAAACTCTGGAAACGGTGTTGCATATGGAATTATCGGAATT
GCATAA
>LSDV 01 00131 group 106
ATGACAAATACTTCCCTCCTTTTAAGCTACTTTTTCTTATTTTTGTACGGAAACGTGTTT
GTCAAATCTGACTATAACTATTTAATGTATAAGATAAATGTTTTTAAAAACAATGAAAGT
ACTATCAAGTGCTATCATAATAATGATATTATTTTTTTATCAGATGATTGCGTAAGCTTT
AACTCTACATATAATACAACTGTTTTAAACAATGATGATGTTAAAACTGAACTTGTTACA
TTGTGTGATGTATCTAAAGAAGTACAAATATTCTCTCTCGACAATTCTTATACTGGTCTA
TTTTTAACTTTTTTATGCAATAATAACGATAGTTATTGGTTTGTTGATATTTTAGAAAAT
GGAATAGGTAACAATGTTCCTACATGTATCGATGGAGAAGTTAATAATAAAAAAGTTAAA
AGTGAATTACAGGGCTCTTTAGATAAGGAAAAAGTGTTGTTGTCCACCTTTTGTAAAGTT
TCGCATGGAGATTGTAGAATGTGGTTTAAAAAACATATATTTTATAAATAA
>LSDV 01 00132 LIG
ATGTGCACATTTAAAAAATTTAGGTCGTTGTGTAAAAGCATTGCGTTAACGCCAAAATAT
TTAGAAAAAACAAAACTTATAAACAATTTCATAAGTTCACCTGATGTAGATAATAAGTAC
TTAGTAATAAAAATGTTATTGCCAATGGTCAACAAACGGGTTTATTATTTAAACGATTTA
CAAATCATTAAAATTTTTAGTAAAATATTTAAACATGATTATAAGACCATGTTAGAAGAT
TTAGAAAATGGATATGTATCTAATACAATAAAAAAGTTTTTTAAAATCAGCAATACTAAT
ATCGTTCCTATACAAAAAAGTATTTTATTACTTAACGATGTTGACATGTTTTTAAATCAT
TTAACTACATTAACAAAGGAAAAAGATAAACAAATGTTTTTAACAATGATATCCAGTATA
TGTACCGCCGACGATTTAAAATGTTTTATTTTATTTATCAAAAATGATTTGCAAATAAAA
GCAGGTGTAAAATGTGTACTAGATGCATTAGATAAACATGCATATAATCATTTTAAAAAT
TCTATAGATTTAAAAAAAATAGTATTTGGTATATCTTTGAATAAGTTGAACGATGATAAT
ATGGTACTTTTTACTCCTGTAAAACCTATGCTAGCAGATGTGTGTAAATCTAGCTATTAT
GCATTTAAAAAGTATAAAGATGGAATCTGTGCTGAAATAAAGTACGATGGCGAACGAATT
CAAATTCATAAAGACAATAATTTATATAAATATTTTAGTAGAAATCTAAAACCAGTTTTA
TTTCATAAAATAGAGGGATTTGATGAGTTTCTAACCAGTGCGTTCCCTTCGGCTAATAAC
TTTATTTTAGATGCTGAATTAATATTAATTGATTTGAATACTAACAAATTTGTACCGTTT
GGTTCATTAGGTATAAATAAAAAAAAATTTGTTTAAAAACTCTAGCACTTGTTTATTTGTC
TTTGATTGTTTATTTTACAATAATGTAAGTTTAATAAGTTTACCATTTGTTAATAGGAGA
AAAATTATTGTTGATAATATCAATGAAATAAAAAATAAGGTTATGGTGTCAGAAGCATTT
TATGTGTATAAAGAAGATGATTTAAATAAAATAATGGATGACGTTATTAAAAAAAAATTA
GAAGGGTTGATATTAAAAGGAATAAATATCGATTATGTACCTGGAAAAAGAGGTTGGTTA
AAGATGAAGAAGGATTACTTAGATGATGGATCCATGGCAGATTCTGCAGACTTAGTAGTT
TTAGGATCATATTACGGTAAAGGGTCTAAGGGTGGTGCGCCATCAATATTTTTAATGGGG
TGCTATGATAATGAAAGTAATAATTGGAAAACGGTAACTAAATGCTCTGGCCACGACGAT
GACACATTAAAAAAAGTACAAAAAGAAATAGAGTTTTTAAAAATTAGTAAAGATACAAAA
AAAATCCCAAAATGGTTAATAGTAGATAAAATATATTATCCAGATTTTGTAGTTTGTGAT
CCAAAAAAAATCACAGGTGTGGGAAATATCGGGATCTAGTTTTACAAAATCGATACATCAC
ACAGCAGATGGGATTTCGATAAGATTTCCTAGATTTAAAAAAATTAGATATGATAAAAAT
TGGTTATCGGCTACAAGTTTGAACGAACTTAAGAATTTATACAAAGAATCGTAA
>LSDV 01 00133 group 2
ATGAGATCACATTCATCGTTTGAATTGAATACCAAACGTCAAGACTTTGATGTTTTGAGG
AAAGCAAATGCTATGGCACTTGAACAAAAAATTAATTGGACAATGTTAAAAGAAGAAGTA
GATGATCTTTTTAAAAAAAATTGTACTGATTCTGATAATACAATTTATAACGGCATATTG
TCTGAATCCATAACTTTTGAAATAAATCATGGATTTTTTAATGTGGATACTGAAGTCAGT
ATATCAAAATCATTATTATATATGGATTTAGGAGTAGAAAATTATACTGATGTATTAGAA
AGTTACAACATTACAATATCAAACATGACAACGATGGCCACACCGACACCCGCTACAGTA
ATGACAACACCGTTATATTTAGAAAATAAAAATAACACCAATGATATTAAAGTAAAGCTT
TACATGGTTAATTCAAGTATTATTCTAACATTTGAAAATATAACAATTGTTATAAATAAT
ACATGTATTGGTACATCCATAAACAGTATTTATGCAAAAATAACAAGTGAGTTTATAACT
GTTAATGTCACAACAGATCCGTTGTCTACAACACCTCCATTTTTAACTAAGTCTATGTTT
GATAATTGTACCTTAACACTTCCTGTTACTATTTCTCAAGAAGGATATTATACAAAAGAA
TATAATGAATATACTGGTACGACTGAACCATTTACTACATATAGTTATTATTCAGATGAT
AATATGACCGAATCATTTACTACTGCATTTAGTTATTATTCGGATGATACCACCACGACT
GAACCATTCACTACATCTAATTATTTAAATGATACCACATCGGAACCATCATTTACTACA
TCGAGATACTATTTAAACAATGGTACATCTAATTATAAGAATATTTCATATGTATACAAT
AACAAAACAGGTATCGTATTTGTAAAACAACAAAATAACATTTTTAAAAATATAACAATA
CAAACAGAGTTTAAAAATTTATGTAACGAATCAAGTTTAGAAACAAAGGTATATGCTGTA
GGGGTTCCGGAAAAGTTTAATACAATTTTAAAGAATATTAGTGTTGAAAAATCTAATGAC
AGTGATTCATATTTTATTTGTAAAATGATTGACGATGGAGGGAATTGCGGAATTGATATC
TTCATGGATGCTGCAACTAATAATATTAATGATGAACTGGATAGTTCTAAAAAAGGAAGT
AGTACCAGACACGCTAGATCTGTTGATCATATGGAGTTAGATCCATTCTGTTTGCATATG
TATCATGGCATTGACGAAACCATAGATTGTTCACTAAAGAAAAACAATAAAGATATGAGT
AGCCGCACAAACAGAATCAGGAGATCTCCTCCTGATAAAGGTAAAAAACCACAAGTACCG
TTTTTTCTATTGGGAAACCATAAAGGTTTTTTTGAAAATGTTTTAGAAAAAATTAATAAA
TAA
>GTPV 07 00002 group 174
ATGTCACACTTCACATCTCCATATACTAAAGATTATTTTTATGAATTGATGCAAAGATAC
ACCAAACTAAAAAAAAAAAAACACCGGAAAAACTCCTGTTTCTTATTCATAA
>GTPV 07 00136 group 41
ATGGAAAATAATATTGTTGACGTTGATGAATATCGTATGTGTTTTATATACGATAAGGTT
GATTATATAAATATAGACGATCCCATTAAAAATATAATAGAAGAGTACTTTTTATGGAGG
GGGGTTAGTTGGTAG
>GTPV 07 00155 group 175
ATGTCACACTTCACATCTCCATATACTAAAGATTATTTTTATGAATTGATGCAAAGATAC
ACCAAACTAAAAAAAAAAAAACACCGGAAAAACTCCTGTTTCTTATTCATAA
>GTPV 08 00022 group 10
ATGGAAAAAGAAAATAATTGTTATATTCCTAACAACTTTGTTATTTATGATACTATTTTA
AATAAAATAATAACTTTTTGTATAGTTTTAAGAAAGTACAAGATTAACGATGATGAAACC
ATTTGGTAA
>GTPV 10 00003 group 176
ATGATTACCTATCAACAAAAAAAAATCTGTTTTTCGTTTAATATACACTCCTTTACAAAAA
GAAAAAAAGTTTTGGTTTTCTATTATATATAAATTCTTAGAAGAAAAAAGTAAGGTTTGT
TTTGAAGTTTTTTTAGTTAATAATAGAAAAAAAAAGTTCAGTGGTATCGAGGTGGTTTGTG
ACGATAATACGTCAAAACAACCAACCAATATGCCATCATTAA
>GTPV 10 00150 group 177
ATGATTACCTATCAACAAAAAAAAATCTGTTTTTCGTTTAATATACACTCCTTTACAAAAA
GAAAAAAAGTTTTGGTTTTCTATTATATATAAATTCTTAGAAGAAAAAAGTAAGGTTTGT
TTTGAAGTTTTTTTAGTTAATAATAGAAAAAAAAAGTTCAGTGGTATCGAGGTGGTTTGTG
ACGATAATACGTCAAAACAACCAACCAATATGCCATCATTAA
>SPPV 08 00131 group 4
ATGGTTATGAGGACCGACGAAGAGCCTAGTGAAAAACCTAATATGGCAACAAATACGTAT
AATAACAATGATAAAGCTAATAATAAAGATAAAAATAAAGGATTTTCTTACTTAAATAAT
GATATACAAGAAGATGAAAAAAATGTTAATAAAATTAAAAAAAGGAAGAACTAA
>LSDV 28 00019 group 17
ATGTATATAAGCAACGATTTAACTAAATTAAGAGTTAATGAATTAAGGCCCATATTAAAA
AGTAGTGATTTAGTTGTCGATGCCGAAGATTTTGTTCTTAATTTTATAATTAAGTGGGGA
ACTGTCAAAAAAACTAATAGGCTTAATACGATGAAGTTAGTAAATGACGCTGTAAGATTT
ACCTTTTTAACAGAAAAAGGAAAAAAAAAGGTTACAAGCGTGGTCGTCCAGATTTAA
>LSDV 29 00001 group 89
ATGAAAAAAGTATCCATTTACAGGTTTAATTGGCTTCTACTTAATTGGCTTCCACTTATT
AGGTTTAATTGGCTTCTACTTAATTGGCTTCCACTTATTAGGTTTAATTGGCTTCTACTT
AATTGGCTTCCACTTATTAGGTTTAATTGGCTTTTTATAATTAGGTTTAATTGGCTTCTA
CTTAATTGGCTTCAAAATATTTCACCACTCCACTTTTTTTAA
>LSDV 29 00159 group 88
ATGAAAAAAGTATCCATTTACAGGTTTAATTGGCTTCTACTTAATTGGCTTCCACTTATT
AGGTTTAATTGGCTTCTACTTAATTGGCTTCCACTTATTAGGTTTAATTGGCTTCTACTT
AATTGGCTTCCACTTATTAGGTTTAATTGGCTTTTTATAATTAGGTTTAATTGGCTTCTA
CTTAATTGGCTTCAAAATATTTCACCACTCCACTTTTTTTAA
>LSDV 31 00063 group 178
ATGGTGAGCAAGGGCGAGGAGCTGTTCACCGGGGTGGTGCCCATCCTGGTCGAGCTGGAC
GGCGACGTAAACGGCCACAAGTTCAGCGTGTCCGGCGAGGGCGAGGGCGATGCCACCTAC
GGCAAGCTGACCCTGAAGTTCATCTGCACCACCGGCAAGCTGCCCGTGCCCTGGCCCACC
CTCGTGACCACCCTGACCTACGGCGTGCAGTGCTTCAGCCGCTACCCCGACCACATGAAG
CAGCACGACTTCTTCAAGTCCGCCATGCCCGAAGGCTACGTCCAGGAGCGCACCATCTTC
TTCAAGGACGACGGCAACTACAAGACCCGCGCCGAGGTGAAGTTCGAGGGCGACACCCTG
GTGAACCGCATCGAGCTGAAGGGCATCGACTTCAAGGAGGACGGCAACATCCTGGGGCAC
AAGCTGGAGTACAACTACAACAGCCACAACGTCTATATCATGGCCGACAAGCAGAAGAAC
GGCATCAAGGTGAACTTCAAGATCCGCCACAACATCGAGGACGGCAGCGTGCAGCTCGCC
GACCACTACCAGCAGAACACCCCCATCGGCGACGGCCCCGTGCTGCTGCCCGACAACCAC
TACCTGAGCACCCAGTCCGCCCTGAGCAAAGACCCCAACGAGAAGCGCGATCACATGGTC
CTGCTGGAGTTCGTGACCGCCGCCGGGATCACTCTCGGCATGGACGAGCTGTACAAGTAA
>LSDV 31 00064 TK 2
ATGGCAAATATGGGAAAGATAATTATAATAGCTGCACTAGATAGCACGTTTCAACGAAAA
GAATTTAATGATATATTGAAATTAATACCGTTATCTGAAAAAGTAACAAAATTAAACGCT
GTATGTATGGAATGTTATAAAGACGCCGCATTTTCTAAGAGGATCACTAAAGAAAAGGAA
ATAGAACTCATCGGGGGTAAGGAAAAATATAAATCTGTTTGTAGGAAATGTTATTTTTTA
GAATAA
>SPPV 16 00131 group 179
ATGAATTCAGTAAAACTTTTCCCATATTACAATGTCTTATCAAAAACAACAGATAGATTG
TGTATTGCTTTATTTAGAGCAATTTATTTATTTGTATTAAATTTATATTTTTTAAAAAAG
AAAAAAAAAAAACAACATTTATTTTTACGATTCTTTGTATAAATTCTTAAGTTCGTTAAAA
CTTGTAGCAGATAACCAATTTTTATCATATCTAATTTTTTTAAATCTAGGAAATCTTATT
GAAATACCATCTGCTGTGTGA
>LSDV 33 00027 group 180
ATGACAACGCTATTACTAAATGTTGATTCGTTTAAAAAAAATGCATTTCCGTGTTTTAAT
TTAACCAAATACGATCCTATAACTGTAAAAAACTCTTCATTGAGTAAAAGTTTATAA
>LSDV 38 00115 group 103
ATGGATGAATTATTTACATTTTTACATAAAATAGAGAATGAGTATTCTAGAACTATATTT
AATTTTCATCTTATAAGTTATAAAGACAAGTACAAAATATATGATATAATGAAAGAAAAA
ATATCAATCGAAAACATTTTTAACAATATAGCATTAACTGATGAAATTAAGAATCATATT
AAAAAGTTGGTTTATTGTGATATCCATTTAACAAAGCATATTATAAACAATATAGTTTAT

```
GATTATGATCAAGTAAATTATTTAAAAGATTACATAAATATAAGTGATGATTATTATCTG
TATGACGCATGCGATGATTGTATCATTAGTAGCGACGACGATGATGATAATGATAATGCG
GACGATGACGAGGAAGACGACGACGAGGTTAATGATATAGAGGATGACTATGAATGA
>LSDV 01 00068 PAPS
ATGGATAAACCATTTATGTATTTTGACGAAATAGATAACGAATTAGAATATGACCCTAAA
ACTTCAGAAGAAAAGCCAAAAAAATTACCATATCAAGGTCAATTAAAATTACTTCTTTGT
GAATTATTTTTTCTGAGTAAGTTACAAAGACATGGAATATTGGATGGATGCACAATAGTA
TATGTTGGATCAGCTCCAGGAACACATATTAAATACTTAAGAGATCATTTTTTATCTATG
GGATTAGTTATAAGATGGATATTAATAGATGGACGACAACATGACACAATTTTAAATGGG
CTTAGAGACGTGACGTTAATAACTAAGTTTGTAGATGAAAGTTATATAAGAGTTTTAAAA
AAACAGCTATATCAATCAAAAATAGTTCTTATTTCTGATGTTAGATCCAAAAGAGGAGGA
AACGAACCTAGCACATTTGATTTATTAAGCAATTACGCATTACAAAATATAATGGTAAGT
ATATTAAAACCAGCAGCATCTAGTTTAAAATGGCGATGTCCATTCCCTGACCAATGGGTA
AAAGATTTCTATATTCCTCACGGAAATGAAATGCTTCAACCATTTGCGCCTAAATATTCT
GCAGAAATGAGGTTAATAAGTATTTATAGCGGTAATCCTATAAAGCTTAGATGCATCACA
AAGGATGATTCTATAAAATATGAAAAAAAGATGTTTTATTTTAATAAAATAATAAGGAAT
AGAATTATTATAAACTTTGATTATTCAAATCAAGAATATGACTTTTATCACATGTATAAT
ATGTTAAAAACTGTATATTCTAATAAAGATTTTTCATCTATAAAATCAAAAGTTTTATTT
TTTCACCATTCAATTTTCAAGTTTTTAAAAATACCTATATTCAACACAGAAAAAATAAAC
TATGAACCCGCACAACGTAAAATACTTAGCAAAAATATTATGTTTAAAAACCGAAATATT
AAAAAATCCATACGCTATAATAAGTAA
>LSDV 01 00069 group 150
ATGTTAGATGAAGATATATATTCTTTCTGTGATAAAAACGAAAACGATATAAGATGTAGG
TGTTTAAATCCTGATTCGAGTATAATAAAAATAGGAGAAGAAACAAGATTACCATACTAT
TGTTGGTATGAGCCATGTAAAAGATCAGATGCGCTTATTGTTAAATCGCTTAAAAAGAAC
ATATCACTATGTAATATTTCAGATTGTAGAGTAACATTAGGTAATATTAAAATAAAAAAT
GGATATATTGATGTTAAAAACGTTTGTGGTACTAATTCATCTTTTACAAATGAATATATT
CGAACTAAATATTTAAATCAAGAAATAGAAGAACCTATCATACATCCCATATGGTTACCA
ATATCTTTATTTATAATAACGTCTCTAATACTGATTACATAA
>LSDV 01 00070 rpoC
ATGGCAGTAATATCTAAAGTAACATATAGTTTATATAATCAGGAAGAAATTAATGCTACT
GATATATTTATTAATCATGTCAAAAACGATGATGATGTTGGAACGGTTAAAGATGGCAGG
TTAGGTGCCATGGATGGAGCATTATGTAAAACTTGTGGTAAAACAGAATTGCAATGTTTT
GGACATTGGGGAAAAGTAAGACTTTATGAGACACATATAATAAAACCAGAATACATAGGT
GAAGTAATAAGAATTCTTAACCATATATGTATAAGATGTGGATTTTTAAGATCTAGAGAA
CCATACATAGAAGACGTAACCAAAATGTCATCGTATGCACTTAGAAAGTTAAAGGATAAG
ATCCTTTCTAAAAAAAAAATCTTGTTGGAATAGTAAATGTATGCAACAATATCAAAAAATA
ACATTCTCAAAAAAAAAAAGTATGTTTCGTAAACAAATCAGACGATATCACAATCCCTAAT
GCGTTGATATATCAAAAAATAACGTCAATTTATAAACGTTTTTGGCCATTGTTAGAAATT
CATCAAAAACCGGAAAATTTATTTTATAAAAACTTTTTTCCTGTACCACCGTTGATAATT
AGACCAGCTATTAGTTTTTGGATAGATAGTATACCAAAAGAAACAAATGAACTAACTTAC
TTATTAGGTATGATTGTAAAGTACTGTAATATGAATGCGGACGAACAGGTGATACAAAAA
GCAATAATAGAATATGATGATATTAAAATTATATCAAATAATACTACTAGTATAAACTTA
TCATACATAACATCTGGAAAGAATAATATGATAAGAAGCTATATTGTTGCAAGACGAAAA
GATCAAACTGCTAGATCTGTTATTGGACCAGATATCACATTAACAATTAATGAAGTAGGT
GTTCCATTTTATATAAGGAATACTCTAACTGAAAAAATATTTGTAAATCCATTCACCATG
GATGAGGTACAGAGATTATTTACAAATAATGAGATTAAGTTTTACTTTAATAAACGACTA
AATCAATTAACTAGAATAAAGCAAGGAAAATTTATTAAAAACAAAATACATATGCTTCCT
GGGGATTGGGTAGAAGTACCATTAAAAGAAAACACGAGCATAATTTTTGGAAGGCAACCT
TCTTTACATAGATACAATGTTATTGCTTCTACAGTTAAATATATTGAAGGTGATACAATA
AAAATACCACCCGGTATAGCAAACTCACAAAATGCTGATTTTGACGGTGACGAAGAATGG
ATGATACTTGAACAAAACCCTAAAGCTGTTATAGAGCAAAGTATTTTAATGTATCCAACT
ACATTGTTAAAACATGACGTTAATGGATCTCCTGTATATGGTTCTATACAAGATGAAATA
GTGGCAGCGTATTCCCTTTTTAGGATGGAAAATTTAACACTTGATGAGGTTATGAATCTA
TTAGGAAAGTATGGTATTAATTTTTCATCCAATGGAAAAACAGTATTCTCAGGAAAGGAT
ATACTTGAATTTCTTATAAATGATAAAGTTAACTATCCTGGCATTTTAAGTAATGGTAGG
ATAGTCGCAAATGATATTATAAGCGATTTTATAGTTGCGATGCGTGGCATGTCACTTGCC
GGGTTTATATCTGACTATAAATCTAACGTAGAAGGTATTGAATTTATCATAAAATCATCT
TACGTCTTTAAGAGGTATTTAAGTATTTATGGATTTGGGGTCACATTTAAAGAATTACGT
CCCAATTCTGATTTTACAAACAAATTAGAAGCAATAAACTCAGAAAAAATTGAGTTAATT
AAAAATTCATATAAAAAATATCTAACAGATGTAAAAGATGGTAAAATATTACCTCTTTCA
AAATCTATGGAATCTGACGCTGTAGAATCAATGCTTTCCAACTTAACAAACCTTAATATA
AAAGAAATTGAAGAATATATGAAGACAACATTAATCAATAATCCAGATAATAATTTACTA
AAGATGGCAAAGGCTGGTTATAAAGTAAACCCAACTGAGTTAATGTATATATTAGGAACT
TACGGACAACAAAGGATTGATGGTGAGCCTACAGAAACAAGGGTGATGGGAAGGGTGTTA
CCGTATTATTTACCTGATTCAAAAGATCCAGAGGGGAGAGGATATATATTAAACTCATTG
ACGCAAGGGTTAACCGGATCACAATATTATTTTTCCATGTTAGTAGCACGATCACAATCA
ACAGATATTGTTTGTGAAACATCTAGAACAGGAACGTTGGCACGTAAAATTATAAAAAAG
ATGGAAGATATGGTTGTTGACGGATACGGGCAAGTTATTTACGGTAACATTTTGATAAAG
TATGCGGCTAATTATACAAAAATTTCAGCATCCGTTTGTAAACCAATTGATTTAATATTT
CCACGAGAGTCGATGGTTTGGTATTTTGAAATAAGCTCCATTTGGGAAAAAATCAAAGAT
GGTTTTATATATTCGCAAAAACAAAAAATATCAAAAAAAACATTATCACCATTTAATTTT
TTGGTGTTTTTAGAACCATCTGATGAAAAACATGCAATAAGTTCTAAAGATTTATACGAC
ATGATACAAAATGTAATCGATGACGTTAGTGAAAAATATTTCTTTACTATATCGAATATA
GATTTTATAAAGTATGTATTTCTAACTCACTTAAATCCTTCAATAATCAGAATAACAAAA
GAAACCGCTACAACAATTTTTAAAAAATTTTATGAAAAGTTAAATTATTCATTAGGTGGC
GGGACCCCTATAGGTATTATTGCTGCTCAGGTATTATCTGAAAAGTTTACTCAACAAGCG
TTATCTAGTTTTCACACCACTGAAAAGAGTGGAGCTATAAAACATAAACTTGGTTTTAAT
GCAAAAGGAAATTTACAACTAATGAGCGCTGAGGAAATGGGCGCTCGACCAAAAATAAGA
AAACAAACGCAAGATATACAAATTGGTGCTAGAGGGGTCGATGGCCCAGTTTCTGGATCA
GAAGAAATATATTCTCAAGTTAGGAAAGAACTATCTCAGAAGTTAAAATCTTTAGTTTTG
GACGATAATGGAAAATTGACAACGACAAGATTGCCTAAATCGACAAAAGCTTTAATTGGT
GGGTTGGTAGATTCTAAGATAGAGACATCTAGTGCTGCGTCTGATATAACAAGACAAATA
GTCCATCAACAATCAGGAGATATTTATTCGTTGCCTGTAAAGAATAAAGTATTCGGTGTA
GAATCAAATAGAAAGATCACATCTCCAATTATAGACGTCGATACATCATCTAATATATAC
GCAAACGTTTTACAAAAAAGAGATTTTAAAGTTACGTCTCCAAAAAATGTTGATGTATAT
GAAAAAACTAGAACTGATTCTTTTTCAGACGATTCGTATTTTTTGAAAAGATCATTTTCT
TCTAGTTCCGACGATACTTACTTTTTAAAAGAAACAGATGATAGCGATAGTGACATTTTT
AGGAGATCTTCAAAAAAGGATCAAACTTATTCACTAGCAGGAAAACCATTAAAAAAACAA
TCACTTAGAAGGTATTCATCTAGTGATTATGAAACAATAGGAGAAAATATATATGAATCT
ATTCGTGAACCAGAATATGCACTGCTTTCAAAACCAAGAGTTTTAAATCCACGAAGTCAT
ATACCCCTACCATCAGTACCTAAGGATGACATACCATTCACTCAACAAAAAAGAAAGGTG
ATTGACATGATTTGTGACTCAAGGTCTGCATCCTCTATATGTAATGCAAGAGGGTTGGAT
TCAGCAAATTATAGAGGTGATGGTAACATATATGAAACAACTGACGACGATTTTGTAAAA
CGTGAAAATTCTTTATATGCAAGATCAAAACTAGAACCAGAATTAAAGGATAATCCACTG
TATGAATCATCGTCTGATAGCGGCATTGTATCAAATCCATATAACAATCCTAAATTGTCG
AGAAGAAATGCTATTAAAAAGAAAGTATTAAACGATGGTTATGAGGAATTCGTTATAAGG
ACCGACGAAGAGCCTAGTGAAAAACCTAATATGGCAGCAAATACGTATAATAACAATGAT
AAAGCTAATAATAAAGATAAAAATAAAGGATTTTCTTACTTAAATAATGATATACAAAAA
GATGAAAAAAATGTTAATAAAATTAAAAAATCAAAAAAAGGAAGAACTAAACGTTTAACT
GAATTATCCACTGATAATAAAATGAATAATATGATAAAAACAATTGCTATTTCCAGTTAT
TTATCATCAACGAATAGTAGGATATCGTCCATAATGGCACAGGCTAATCACCAACCAAAA
GAATTGACAATTGTTAACATTGTAACGTCCGTATTATCGCAGATCGGTGGAACTTTAGCA
ATGGCTGGCTCTGGGTCTCCCAAAGCTGCAGCTGCAGGTTTAGTTATTCAAGGAATATCT
GGATTAATAGATGCGGCGACATCAATTTATTTTCTTCTTTCTGGTCAAGAACAACCTAAA
GATCCAGCTATTGAAAAGTTTTCCAACTACGCATCTTACGTTTCAAAAACAGATGCAGGA
GCAAGGGTTTGTATGATGCCAGATTCAGATATTACAATAACGTTAGCATATAGACATAGT
AACATGAATGTAGAAGCTGAAAAAACTAGAGGTGAGTACACAGATATAATTCCAAGTACC
GTATACTACTTAAAAAACAGTCAAATAAGTTATACGGTGAAAGTAACATTGGTGTGTCCA
ATCGGTCAGTTGAGGCTTTTGGAAGCAGATATAAATACATATGCTACACTTACTCGGGAA
GATAAAGATGGTGTAAAATTCTATCATGTGTTTGGGATTTTAGAACTTCTATCATATCAT
CCTAATGTTACATTTACTTGTGGAAATGAACCAGGTGTAATCTTCATCCCGTTTGAGCAA
AAACTAAGCGACATGCAATTATTAAGGATATCAACTCCTGGCGAACCTAGAGAAGCCGAA
GCAATGAGTTCTGATGTTTGTGATATTTATCCTCTTAAGAAGTTTTATGTATTAGCTGGA
AATTGCCCTTTTGATATGAGCAGAAAATCAGTAGCATATGTTACATGTAGTACGTTACTA
AGAATGTCAACGTATGAACATGAAAAGCAGAGGTGGATTTTAATGAATCCGTTTTCAAAC
GGCAATGAGGACAATATTCAATTGTTTACGTTTAAAAAATATGACTTTAGTGCAGAAAAA
GATAAAATAAAACTAAATAGTATTTCACATAGTGATACTATTTGTAGCCAATCTGATACT
AGTACATGTTTTTGGGCAGATGCTATGATTTTAGAAGATGTAACATCTTGCAATTCTAGG
ATAAGAAAATTGTCAGTTGAGATGTCTACAGTATCAGAAAAAGGATATAACAATTTTGTA
CTTACGTGTCCATATGGGTCTACACCGTTTTATATAAGTAATGGTTCAATAATAAGTATT
CCAATGAACACAAGAAGGACGTCGGTTAGATTTGCTTCTAAACATAACAATGTTGCACTA
ATATCATGTATACATAATTCAAACCCTGCATATAAATCGGATATCGTTGAAGTTATGTTT
AAACAATCAAATATGAGTGATATGTATTTGGATTTCAAATATTTTAAGGATAGAAAATAT
TTATTTGATTCATTTAGTGATATAATGCCTAAAAGATCAAAAACTTGTAAGAGAGCATCA
GAAAATAGTAATTGTAAAAACTATTATTATATAAAACATGTTCCTGAAATAGAGTTTAAA
GTGTATGTTGCAAAACTTCCAATGGTTAGATTAGGCACAAGTTATTCAGGTGTTTTGAAT
TATCAAACATTAGAAAAAGTTAATAAATATTTTTCAACACCTATAAGTATTTCTGTAGAT
GCTAGTACTCTATCCGATGTATACAAAAACCAAGAACATTTTTGGAAATTTGCAATGGAA
AAGAAAAGAACTTTTAGTTCTATTACAGCTACCATATTTGCGTGCTCTGTTGTCGCTGGG
AAGGTAAACGTAAACATGGGAGTTAAAGGTTCACACGATTATTATGGAAGAAGCGGTAAA
TATATTTACATGGGATCAAAAGATTTTGCATCTAATGATAAAATATATTTCAAATTTATA
CCTGACAAAGCAGAGTACCGTTTAAAGGATACATATGGCGAATGTGAAATTTATTTAGAT
TTAAAAACTAGGAGAGTGAATGTAAACTGTCCGGAATTGACTATACCACAACATCCATTT
GATTCACCAGATGTTAATAGTTTATGTGTGTTAGTTGCCACATCAAGAGATCATTGTGCT
ATATCTGAGGAAAATTGGAGAAGTTATGATAGAACTCATGGTGTTGGGTATAGCCACGAA
TACGTAGATGCGGAGTTTGATTCTTGTAGGAAGAGCCATGGTCCAACATATCCAGTTGAT
AACTTTTGCTTTTATTGGTGTGCTGGGATATATTGGCCACCTGACTATGACCCATGCGCT
TCATCGATGGTGTTAGGTTATTCTCCGATTTTTCCAGAAAATAGAATTGTGCACCCTCCA
TATATAAAGGAATTTGGTTATGAACCAGGAAAAAATGAATACGTGAAAAGAGAGTTATAT
AACAAATTACAAAATCTTTATGAAAAATATAATATGTTGGTTTTATATTCAATGAACCCG
GTTGTTGAAATGTCAAACGGATTAGCAAAATCTATGACGTCAGAAGCCCGTGAGATTTTT
AGGTTAATAGCAAATAGTAACGAAATGCAAAAAGCTAAAGAAGAAAATGATATGAAAGCT
GAGAAAGTTAAAATGGAAATAGAAGAAACATTAAATAATATTTACGTCGATACATTATCA
TACTCTGAATCAACTTCCCTTTTACGGTCTGCTATTTCCACAAGGTGTTGTGTTTTGGAC
GGTACTAGCGTATATAAGTATTTTGATTTAGAATATTATCTTTGTGGTAATTATTCTGAC
TACTTAATTAATATTAATAATGTAACATATGTAAAAATTAATGATAGCCTAATAGAAGAA
GATATCTATTTGGTAAGAAATATTCCACAGGTAACATGTTTTCATATAACGTTGGTGTCT
GTAAATAACGAAGAACAACAGAAAAAGTTTGAAACAGAAATTGTGACAATGGCATTTGAG
GATGTTTTAACTGAAATATTTGACGAGTATGATGATAGAATGGTTAGTTATTTTGACAAA
TATATAAGCGATGATAATAACAATAAAAATAGCAAATTTACCACCAATAACACAATTGCT
ATCATATTATCATTTGTTTTTACGGCAATAATTATTCTAATTTCTACTAAATTAATAATG
CGCTCTAGAAAAGGCAAATATACCATACATAATAATTTTGTTATCTTTAAAAATAATAAT
AGCAGTAGTGAAGATGATAATACAGTTTCATTATCTGATTTAGATTTAGATAATAATAGT
GAGTTTTTATATTTTTGA
>LSDV 01 00134 group 135
CCTAAATATGATAATTTAAATCAAAACAATAATTTAAAATTTTCTCAATTTTTTGATATC
AATAAAGATGATGACTGTATTAGTTTAAGAACTGCTGAAATATTTGATAATGATAAATCA
TCATTAATATCTTATATAAAAACCACCAATAAAAAAAAAAAAAGTTGATTATGGCGAGATA
AAAAAAACAGTGCATGGGAACACAAAAAATTATTCAAATTATTTTCCGGCAAAAAATCT
GACGATTATCTTGTTACGACGATAAATACTAAAGAATCACAGCCATGGATAAAAACAATT
TCTAAGCGTATGCGAATAGATATTAAAAAAAATGCAATCATAACAAAGGGAAAAAGTTCA
ATTTTACAAACAATAGAAATAGTATTTGTAAATAGAACATGTATAAAAATTTTTAAAGAT
TCGACGATGCATATTATATTATCAAAAGAAAAGGATGAAATTGGTTGTGTTGGAATTATA
AATAAAATATTTCAAGTATATAAAATATTGTTTTGTTTACTTTACGATATTTCAAAAAAT
GAAAATTTTAAAAATGCATTTGATAATTCTGTATCTATTTTAAACTTAACTAAGTTTGAT
GATAAGATTGCAAAAATAAAAACAGTAAAAAATGAATACGGGTTTGAAAACTTTAAAATT
GGAATGTTTAATTTAACATATAACAAACCTATTTTGCATACAGTATTTCCGTCGTTGTTA
GATTACGAAAACAAAATAAAATTTTTTAAAGGAAAAAAACTAAATATAGTTGCTCTTAGA
TCATTAGATGAATGTATTCAATATGTGTCGTTAGCAAATAAAATATTAAAAAAGATGGCA
GATCGGTCCGATATTCTAAATAATTTAAATATAGAATCTGAAAGTATAGAAATACTAAAA
AAAATATTGTTTAATTAA
>LSDV 38 00120 group 138
ATGTTCCGAGAAGAGAAGGTAATCGTTATTGATTTAAATCCAGATTTGGCAACATTTATA
AAACATGGATTTAACAATAGAGTTAGATGGCCGTTATTAACTACAGGTGTGGTTTTATCA
AATAATACAACTGCCGTCAATGAAGAATGGTTAACTTCTATAGAGCATATGCCTACAAGA
AAGATATTTTATAATTACACATCGAAAATTTTAAGAAAAGAAGTAGGTTTTTGTGTATAT
TTAAAAAAATCCCAAACAGAGAAAAAAAGAATTATTACATTGGGAGATTTCGACTATTAT
ATTATAGATTTAGAAAATTATAAATTTATAAAAATAGATAAACCTATTGAATTAAATGAC
ACACACTTTTCAAGATTATAG
>LSDV 38 00159 group 38
ATGGAGTTTTTGATTTTATTAAAATTCGCTAATCCATTTAAAAAACACTACGAATACTAC
TGTAATCAAAACACAATAAAGGAACCACCTAGAAAAGGATTAGTTGAAAGGATGATGAAT
ATGGTTGAATAA
>LSDV 47 00116 group 45
ATGGAAAATAATACACCACCTAATCACTTTGAAAAAGCTTCTGTTTATGCATGTAGGTTA
TTTAAACAGTTTTATAACGAAAAAAAAATATGATTTAGATAAATGTTTTTTTAAAAGTTAG
>LSDV 48 00058 group 77
ATGGATCCTGCAACTTTTATAAAAAAATTATGCGCCTAAAGGCGCTGTTATTTTTATTAAT
TATGAATTTTCCTTAACTGAATATTTTAATCCATCAGAAGATAAACACGCTGCTATATAT
ATCGGATCCGACTGTACATCTAACCATGTTAAAATGTTTTTATCGTGTAGCAAATTCGAT
GAAAAAAATGGCAATAGAAGCATCGTATAA
>LSDV 57 00065 group 181
ATGGCTGCAGAACAAGTAGAAGATTATTGCATCAGCTTTGTGGAAATGAAATTTATTAAC
AATACACTTTATTTTGTAGCTGAAAATGATGAAGACCTGGAATCAGATCACTTTGGCAAA
CTTGAACCTAAGCTCTCAATCATACGAAATTTGAATGACCAAGTTCTCTTCATTAACCAG
GGAAATCAACCTGTCTTTGAGGATATGCCTGATTCTGACTGTTCAGATAATGCACCCCAG
ACCATATTTATCATATATATGTATAAGGACAGCCTCACTAGAGGTCTGGCCGTAACCATC
TCTGTGCAGTGTAAGAAAATGTCTACTCTCTCCTGTGAGAACAAAATTGTTTCCTTTAAG
GAAATGAATCCTCCTGATAACATTGATAATGAAGAAAGTGACATCATATTCTTTCAAAGA
AGTGTTCCAGGACATGATGATAAGATACAATTTGAGTCTTCATTGTACAAAGGGTACTTT
CTAGCTTGTAAAAAAGAGAATGACCTTTTCAAACTCATTTTGAAAAAACAGAATGATAAT
AGAGATAAATCTGTAATGTTCACTGTTCAAAACCAGAACGCGGCCGCAGATCTAATTAAT
TAA
>LSDV 58 00133 group 3
ATGATTTGTGACTCAAGGTCTGCATCCTCTATATGTAATGCAAGAGGGTTGGATTCAGCA
AATTATAGAGGTGATGGTAACATATATGAAACAACTGACGACGATTTTGTAAAACGTGAA
AATTCTTTATATGCAAGATCAAAACTAGAACCAGAATTAAAGGATAATCCACTGTATGAA
TCATCGTCTGATAGCGGCATTGTATCAAATCCATATAACAATCCTAAATTGTCGAGAAGA
AATGCTATTAAAAAGAAAGTATTAAACGATGGTTATGAGGAATTCGTTATAAGGACCGAC
GAAGAGCCTAGTGAAAAACCTAATATGGCAGCAAATACGTATAATAACAATGATAAAGCT
AATAATAAAGATAAAAATAAAGGATTTTCTTACTTAAATAATGATATACAAAAAGATGAA
AAAAATGTTAATAAAATTAAAAAATCAAAAAAAAAGGAAGAACTAA
>LSDV 63 00049 group 94
ATGATATCATTAATAGACTTACCATCTAATGTTGAAATACGAACGTTGTCAAAAAATGGA
ATAGATTATATAACACATATCAACGATAAAAAATTATCAACAATTTTGATTATAACAAAA
GATAATTTTTTTAAAAATGTTTCATTTTCTGGCACGTTTAAAAAAGAAAATATTATATGG
AAAGGATACTATACATACAGAATAATAGAATCGACATTTGATGTGCCTAAATTAAAAATA
TCACCGAATGGTAAAAAAAGATACATAACTTGTAAAAGTGCCTTTAATAATTCAACTTTT
ACAACTAGGAGTGGACAATATATTGTTTAG
>IPFAONOG 00043 group 47
ATGACAAATTATATAACCTCTGTAATATTATGCAAGTACAAAAAAATATATGACAGAATA
AAAACATTAGAAAATTTAGTTAATATGAACTTAATTTGCAATTGTGAAAATTGCAACAAT
AGTAAAATAAATCACAAAAGTAAAAATATCTATCTTGGTAAAAAAACTTCAGATGATGAT
ATCATTAACTGTTTATATGATTTAGTAAATTATGCTATTCATGGATTAGTAAATCCATCA
CTAATTGGAATTAAGGGATGGGGGGCATTAATTTGTTTACTTAATGGAACAAAAAAAAAT
AAAATAGATATTAATATTTATAATACAATGGATTTTAATTTTGTATCCACCAATGGAGAT
ATCTTACTATCTGGATCTTACCTTGTAAATAAACTTATTCCAATAACAAAGACACCATTA
TCGTCATTTGTTCCATTTACATGTTTTTCACTAAATGATTATTTTTCAAAAACCGTTATT
TTATTTAATATAATTTTAGAATATATGATATCGTTTGTACTGTATCGAATTATTACAATT
CAAAGATATAATAATTTAAAGGAATATATTTCAAAAGTAGTTAATACATGTTTGGAAGCA
TCTGGTGTTTTCTTCGCACAAATAAATATAAGTAATATAATTGAAACAGAAATAAACGAA
ATCGTTATAAACGGTTCTTTTCCAGTTCGTTTACTTAATATATTTTTGAAAGTGATTACT
ACTATATTAGAGGAAATAAATGGAATTAGTTAA
```

GAATTTAATAACTTAACAAATTTAAGCAAAAATAAAACAGAAATTATTACATTAATATCA
GAAGATATAGAAAAACTTCAAAGTGTTAAAATAAACTTTGAGTTTGTATGTTTAGGAGAA
TTGAATCCAGATATTGTATTAAATAATGATAATGAAAAATACATAATCGATATCACTATC
AATCGTATTTATATAAAACGAGCAGATATAACTGAATTAGTCGTTGAACATATGATAGAA
AGATTTGTATCATTCAGTGTATTAGTTAAGGAATGGGGATTAGAAACATATATAGAGGAT
AATAATAATATAAAGTTTACAATACTTGTAAAATTTATCGAACCTGAAGAATTATCACTA
AATAAGTTTATGATGGTATTGCCAGGAGCTGCAAATAAAGGGAAAATTAGTAAATTTAAG
ATCCCTATAAGTAATTACAATTACTACGATGATCATAATAAAAGTAGGAAAATGAATAAA
ATGACAGTTGAACTGATGAATTTGAAAGAGTTGGGAACCTTTAATTTAGAAAATGTAATA
GTATATCCTGGTATATGGAATACATATGATATATTTGGTATAGAATCAGCCAGATCATAC
TTATGCGAAGCTTTATTAAATACATACGGTGAAGGATTTGATTATTTGTATCAACCATGT
GACTTATTGGCTAGCTTAATTTGTATGAGTTACGAACCCGAATCAGTTAATAAGTTTAAG
TTTGGTGTTACAAGTTCCCTTAAAAAGGCTACGTTTGGAGATAACAAAGCTCTATTAAAT
GCTGCATTACATAAAAAGACTGAACCTGTTAATGATAACAGCAGTTGTCATTTCTTTAGC
AAGGTTCCAAAAATAGGAACAGGGTACTACCGTTATTTTGTTAACCTAGAATTACTAACT
AGATTGGAAAAGAAACTTTCTGTTACAATATCTGATAGAAAGGTTGAAGAATTAATCGAA
AAATCCGACGAGTTTTAA
>LSDV 01 00071 group 206
ATGGATAAAAAAAGTCTTTACGAAAACGTTTTACTAAAGTCTACAGGTTCTCTACCCAAA
GCGAAGGCACCGACAAAGATGATGAGAGTAACGGATTATGTATATTTAGGAAATTATGAT
GATGCAATTAATGCAATATCTTCAAATGTAAATTTTAAATATATTTTAAATCTTACTACA
GAAAAATATTGTTTTAATGATTCACGTATAAATATTATTCATATGCCTCTTATTGATGAC
GAAAAAACAAATTTAAATGATCATTTTGATTATGTTACAAATTTTTTATCAAAATGTGAT
GAAGAACATTATCCTGTGTTAGTACATTGTGTTGCCGGCGTTAATAGAAGTGGTGCGATG
ATAATGGCATATTTAATGTCAAAGAGAAGTAAAGATATACCTGCGTTTATATATTTTTTA
TATATATACCACTCTATGAGAGAAAAAAGAGGCGCATTTATAGAAAATCCCTCTTTTAGA
AAACAACTAATTGATAAGTATATTATTAATGAATAA
>LSDV 01 00072 group 199
ATGGATAAAACAACATTAAATGTTAATGGTGTTGAATTAGAATACGCTAGAGAAAAAGAA
AATAAAACAATACAAACGGCTAAAACCTCTACTTTATGTTTCTTTATGTTAATTTTAGGT
ATTAGTATACTATTACTTTGGTTTCAAGTATCTGATAATTCTATTTTCTCAGAATTAATG
AAGTATATACGGATAAAAAAATCTATAAGAGGGTGGAGACCTTTAGTTGAAACCAAAACT
AACTTAGAAAGTGATAGAGGAAAAATGTTATCAATGGGAAGAGATGAGTATTTTTCTTTT
AATTGTGTAGATTTTGGTTCTTACTTTGTTCCTATTAGATTAGATAGAAAAACGTTTTTA
CCACAAGCTATTAGAAGGGGAAAAGGTGATGGATGGATGGTACAAAAAGCAGATAAAATA
GATGTATCCGCACAACAATTTTGTCAATATTTAATTAAACATAAATCAGAAAACATAATA
ACGTGCGGAACCCATATGATTAACGAAATAGGATATAGTGGCTATTTTACAAACTCTCAT
TGGTGTTCGGATTTTCATAATCTTTTAAACTAA
>LSDV 01 00073 H3L
ATGGCAGATATCCCATTATATGTTATACCAATCGTTGGTCGCGAAATTTCAGATGTAGTT
CCAGAATTAAAAAGTGACAATGATATATTTTATAAAAAAGTTGACACAGTAAAAGATTTT
AAAAATTCAGATGTAAATTTTTTTTTTAAAGATAAAAAAGATATCAGTTTATCATATAAG
TTCCTTATATGGGAAAAGGTAGAAAAATCAGGAGGTGTTGAAAATTTTACAGAATATTTT
TCTGGATTATGTAATGCTCTTTGTACAAAAGAGGCAAAAAGTTCTATTGCGAAACACTTT
AGTTTATGGAAATCGTATGCCGATGCGGATATAAAAAATTCTGAGAATAAGTTTATTGTT
GTTATAGAAGATGATAACACATTAAAAGATTTAATAACAATACATAACATTATAATTGAA
ATGCAAGAAAAAAAATATAGACATTTTCCAATTACGTGAAACTTTTCATAATAGTAATTCT
AGAATATTGTTCAATCAAGAAAATAATAATTTTATGTATTCGTACACAGGGGGATATGAT
TTTACCTTATCCGCATATGTAATTAGATTATCGTCTGCCATAAAAAATAATAAACGAAATT
ATAAAAAATAAAGGTATTTCTACCAGTTTAAGTTTTGAAATGTATAAGTTGGAAAAAGAA
TTAAAACTCAATAGACAAGTTTTAAATGACTCATCTAAGTATATACTTCACAATACTAAG
TATTTGTCAAAAAAAAAGAGCTAACGAAATGAAAAACGGTATATGGAATAGAGTTGGAAAA
TGGATGGCTCATAGATTTCCTGATTTTTCTTACTATGTATCCCATCCATTGGTTTCATTT
TTTGGTATATTTGATATTAGTATAATAGGAGCACTTATTATTTTATTTATTATAATAATG
ATAATTTTTGATTTGAATTCTAAATTACTATGGTTTTTAGCAGGTATGTTATTTACGTAT
ATAATTTAG
ATGTTACTTTTAACTTCTTTTGTTTTTTCATTGTTGTTTTCGCATTCGTTAGGAAAAAGA
AATTTGTCATATTTTGATACTGATGAATATAAAAATAGTATCAAAGATGTGTATGATTTT
AGTAAAAGATTATTGGACGACGATGTATTGTTTGCGCTAAATGATAACTGTACATTTAGA
GATGAAAGAGGTACACTCGCTACTCTTAAAGAACCACTTTCATTAAAATGTCCACTTTTA
AACGAATATGGGTTAAAATGGCCATATATGGAAAAGGATGATTATGAAATAAAATGGGAA
GTTGTACGTAATTACACTAAAAAAAGTATTAATAATAATACTAACAATTACTTAATTGAA
GATGGAAAATTACTAATTTTAAAAGCAAATCTTAATACGTTAAACAGTAAATATCTATGT
ACTATTACTAGAAAACGAGACAATGATTGCGACCAATCCATTGTGCGTCTCTCTAGATAT
AGAAATAAAAATTGTTATAAGTTAAACGGTTTAAAAGGCAGGAATATCGTGATTGAGTGT
GGGGTTCGTCACGTAGAATATGATACAGTTGAGTGGTATAAACAAAATAAAACATCAATA
ATAAAACTTAATAACGATAGTAAAAGAAGAATTATTATTCGTAATATCACTAAGGGTGAT
TCTGGAAAATATTATTGTAAAGGGCATTATAGTAAACTAAATATTTCATATACAATTAGT
AGGTGTACGTATCTATCAGTGTTTTCACTAAGTCATTACGAATATGAATTAGTTTATATG
CCAACTATTATTAACGTAACAATAGGAGAACCAATGACTGTAAACTGTAGCGTTCGTACA
AAGGCAAACGCATATGAATATATTGGCGCGATGTGGTTAGATGATAAAAAATTATATGTA
GGTATGGAAGAAAATTTATACCAAGACGGAAGTCGTAAAATTGAAGGAGATGATATCATT
AAAACTTCACATTTGGTATTTGTTAATGTTATGGAAAAAGATATTGGAAGAACATTTACA
TGTGAAGTAGGATCACGTTTTAAAGGCGAATATAGAACGGTTACTTTAAACTTAAAAAGT
TAA
>LSDV 01 00135 group 40
ATGGACAATAACATTGTTGACGTTGATGAATATCGTATGTGTTTTATATACGATAAGGTT
GATTATATAAATATAGACGATCCCATTAAAAATGTAATAGAAGAGTACTTTTTATGGAGG
GGGTTAGTTGGTAGAATAGGAAGAAAACCATCAAAGATAGGAAAATTATTTGTAGATTTT
ATTAACTTAGATTTAACAGCAAAAAAAATGTTAGGAGACTTGGATTCATTATTTACTAAT
ATTTTAAAGTTAGATGATATTAATGGTGCAAACGAAAGACTTTCTAACTTTGTGAAGTTT
GTCAATATTAATTTTAATAATAAACCTTTAGTGTTATTAGGATTGCTGGGGGCAGTTTCT
GAATTTTGGGGGAAAAAAAAGATTAGTGTTAATGGTATAATGTCAGTGTTCTTAAGCAAT
GTATCAAATGATACATTGCTTGAGATATGTAATCACATATAG
>LSDV 01 00136 group 136
ATGGATATTATACTTGGTAATACATTTTCTAACAATGATTGGGATATTTCAGAATTCTTT
AAAAATGTATTTGAAAATAATGTATTCGAAAATCCAAAAAATATATGCTCTGTTTTTGAT
AGTATAATTATTGGAAATAAGACAAAAAGTAAAATTATTGTGGCAGATACACACAAAATA
GATTTATCTATCTCTAACACATACAAAGAAAAAAAAAAAGGTAGATTTAATTAGAGTATCT
AGATTTTGTAAAGCCGTGGCTCTTAAAAGTAAAAAAGACTATGTTTACATTCCTGAAACA
AAAATTTCATTTGCCGTACTTACTGTTTCTACAATAGATGATGATTTAAAACATGAACTA
AGTTGTTGCTCGTTGCAAAACAAACAATATGATTTGTCGATGAATATGAATTCGTTTGCT
ATCGTTACTAAATGTAATGGTCTATCAATAAAAGGCACTAATATGTTAATAGTCATAACA
TTTTTTGAAGAGAAAAACTATCCACATATTCCATTGATACGTACTATATCTAGTAATGAT
GTGTTTATATCTAGACATAGTAGGTTGCATAAGGAAATACCTAATAAAGATTGGTTTAAA
TTTTACGTAGAGTTTAATCATAATTATTGTACGTCATTGACGGTTATAATAGATGGAAGC
ATCTTATATTCTAGATCAGATTATAAAACTCATTGTATAATTAGTAAGTATCAATCAAAA
AAAGACGAAATCAACGATGATTGTTGCTGTTGTTATAACACACCATCTGTTTATATACTT
AATAAAAAAGAAATTATTGAGTACGTGTCCTGTAATACAATAAGAGGAGGAATTCATATT
TCATTAAAAGGTGTCGGGGATTTTAGTTCCAGTTATATAGGGAAATGCCCTAACGTTGAA
CATTTAAAAGTTGTTATAGGTTCAACATATGACATGTTAACAAAACAAGATAGCATATCT
GGTAAAAAAATGTATTGTTCTTATATTTATGGAATTGCACATAGATAG
>LSDV 01 00137 group 25
ATGAATAAAATTAATAATATATTTAAGCTTATATGGATAGGTAATTATATTAATGTAATT
TTATCAGCTTCGTGTATCTCAAAAATATATAGCAGTTTTAAAGAAAACACTGTAAAGATT
AGTTGTAGCAAAACAAGCAAATTTAATTCTATTATTATAACATGGAAAAAGAATAATGAA
ACCATTGCTACGTATGGTCCACATGGTTCATACGTAGTAGATGATTATAAAAACAAAATA
GAGTATATATCTAAATCATTTAATTATTCAACTATTGTAATAAAAAATGCTACAATACAA
GATAATTCCTGTTATACATGCATATTTAATATTTTATTAAGTGAAAATGATAAGGGAACT
TTATGCTTAAACACAACAAATGATGAATATATAAACAATTTAATTCCAATAAAAATAAAT
GAAAATAATAATCACATAAAAAATAATATTATTGTTGATGATTACGGTACTACAGAATCA
TTGTTTTCTTTATATGGTACGTTATGTCTTTGTTTTTGGATTTATATGTACTACGTTGAC
ACTGTACGATTTTTCTTATAA
>LEFNJLPD 00001 group 182
TTGTTTTCGATTAATATTTTAACAACATCTAACATATGTTTATATTTTTTTATATTTGAT
AAAATTGTACATAAAGGTGTTGAGTATTCATTTTCTAATCCATTAACATTACCACCTAAA
TTAATTAATAATTTTACAATATCGACCGATGGCGAATCTCGTTGTAAGTATTTTTGGAAC
AATGAATACTCATTAACTATTTCGTTTGGATCACATGATTTAATTATACTTAATTTAGGA
TTGCCCCTAAAAGCTATATAA
>LEFNJLPD 00032 group 49
ATGTTAGTTGATATTCCAAAGAGTGGAACTGAAACAGATTATGATGAAAGTAATAATTTT
ACAGCATTCGCAGGTTCCACTATATACGGATATGGTTTAAAATCAAAAAAAAAATATAAAA
AAAAAGTAA
>LEFNJLPD 00085 group 126
ATGGCAGTAATATCTAAAGTAACATATAGTTTATATAATCAGGAAGAAATTAATGCTACT
GATATATTTATTAATCATGTCAAAAACGATGATGATGTTGGAACGGTTAAAGATGGCAGG
TTAGGTGCCATGGATGGAGCATTATGTAAAACTTGTGGTAAAACAGAATTGCAATGTTTT
GGACATTGGGGAAAAGTAAGACTTTATGAGACACATATAATAAAACCAGAATACATAGGT
GAAGTAATAAGAATTCTTAACCATATATGTATAAGATGTGGATTTTTAAGATCTAGAGAA
CCATACATAGAAGACGTAACCAAAATGTCATCGTATGCACTTAGAAAGTTAAAGGATAAG
ATCCTTTCTAAAAAAAAATCTTGTTGGAATAGTAAATGTATGCAACAATATCAAAAAATA
ACATTCTCAAAAAAAAAGTATGTTTCGTAA
>LEFNJLPD 00155 group 183
ATGGAATTAATCATAATTTTTGATTGTTCAAATCCAATTTTAGAATCCAAAAACATGTTT
TTGACAAAAGCTGTTAGATCATTTCCAAATACAAGTGAGGCATCCTTTTTGAAAGATTCA
AAAACTAAGAACACCTTTCCAGCAACCTCCCTGGAGGAAAATGCCAGTGATGACCAAAAC
AAATAA
>IAFEDADN 00091 group 80
ATGTTAATAGAAGATCTTCTAACAGATAGGTTTTATGAAAACATATTATTTTTTGGAAAA
ACGTTTCTAACGAGTAATGAGATAATACATAATTTTTTTTCTAATAAAGAAATAAAATCA
TTAGTTTTTTTCGATTCTTTATATAATGGAATAGAAGGAGATATAATAAGATTTGTGTTA
GATATTTCAAGATTAAAATGTTTTGGAAATAAGGGTTATGAATTATATAATAAAAACACT
TTTAAAAGTTTAAAAAGCTTTTTTTAA
>KLAIMAEJ 00149 group 36
GTGATATATGCAATAAAAGGATTTTGTCAACTACTCATTAATAGCTTAGCTAAAGTTTGT
ATGTTGTGTATTTTTTATATAGCTAGATTATGTAATTTAATTATATATAGTTTGTATTCA
TTATTAATGTTTCCAATGCAAAAGCTTATCTCCTTCATGTTTGGAAACCTAAATCCGTTC
GATTCTGTCTTGGATAAGGATGAAAAAATCCAAGACAATATAAACACTAATCACAAACCT
ATAGAAAGTAAAGAAATAAATAATGATCTACCATTAACTGTATTAGATAAAAAAGATACA
AGCGATATAAATATTAGAAATGATAATGGAGTTTTTGATTTTATTAAAATTCCTAATCCA
TTTAAAAAACACTACGAATACTACTGTAATCAAAACACAATAAAGGAACCACCTAGAAAA
GGATTAGTTGAAAGGATGATGAATATGGTTGAATAA
>KLAIMAEJ 00150 group 194
ATGTCTTCCGGCAACTATGTCTACCGAAACAACTTTTCAGATGATGATGATATCACAACC
GCCATCTCTGATTATTTGTTTTGGTCATCACTGGCATTTTCCTCCAGGGAGGTTGCTGGA
AAGGTGTTCTTAGTTTTTGAATCTTTCAAAAAGGATGCCTCACTTGTATTTGGAAATGAT
CTAACAGCTTTTGTCAAAAACATGTTTTTGGATTCTAAAATTGGATTTGAACAATCAAAA
ATTATGATTAATTCCATGTTAAAAAAAGAAAATTACATTAGGGAATCATGTGCAGTGATT
GGCATTTTAGCAAGAGCAGCAGAATATTGGGGTGGTGAATCATCTCCAACTTGTTCTTCT
GTGAAAGTGTTGGTATTGCTTCGAGACCTCGTTTCTGACAACGATATTTCGCTAGTGAAA
TCAGCACTAATAATTAGACTTAAAAGATTGAATGAAAAAAGTATCCATTTACAGGTTTAA
>PFHFNDGI 00100 group 184
ATGGCCGAATCTGATGATATTATCGACGATTATATTTCTGACGAAGATGCGAGCGATGAA
TTTNNGAAGAAGAAGAAGAAGAAGAATCGTTAG
